# Novel 2,6,9-Trisubstituted Purines as Potent CDK Inhibitors Alleviating Trastuzumab-Resistance of HER2-Positive Breast Cancers

**DOI:** 10.3390/ph15091041

**Published:** 2022-08-23

**Authors:** Ratnakar Reddy Kuchukulla, Injeoung Hwang, Sang Won Park, Sojeong Moon, Suhn Hyung Kim, Sumin Kim, Hwan Won Chung, Mi-Jung Ji, Hyun-Mee Park, Gu Kong, Wooyoung Hur

**Affiliations:** 1 HY-KIST Bioconvergence, Hanyang University, 222 Wangsimniro, Seongdong-gu, Seoul 04763, Korea; 2 Medicinal Materials Research Center, Korea Institute of Science and Technology (KIST), 5 Hwarangro 14 Gil, Seongbuk-gu, Seoul 02792, Korea; 3 Computational Science Research Center, Korea Institute of Science and Technology (KIST), 5 Hwarangro 14 Gil, Seongbuk-gu, Seoul 02792, Korea; 4 Advanced Analysis Data Center, Korea Institute of Science and Technology (KIST), 5 Hwarangro 14 Gil, Seongbuk-gu, Seoul 02792, Korea; 5 Department of Pathology, Hanyang University College of Medicine, 222 Wangsimniro, Seongdong-gu, Seoul 04763, Korea

**Keywords:** HER2-positive breast cancer, trastuzumab-resistance, CDK12 inhibitor, cyclinK degrader, 2,6,9-trisubstituted purine

## Abstract

HER2-positive (HER2+) breast cancer is defined by *HER2* oncogene amplification on chromosome 17q12 and accounts for 15–20% population of breast-cancer patients. Therapeutic anti-HER2 antibody such as trastuzumab is used as the first-line therapy for HER2-positive breast cancers. However, more than 50% of the patients respond poorly to trastuzumab, illustrating that novel therapy is warranted to overcome the resistance. We previously reported that in the majority of HER2+ breast-cancer patients, *CDK12* is co-amplified on 17q12 and involved in developing tumors and trastuzumab resistance, proposing CDK12 as a potential drug target for HER2+ breast cancers. Here, we designed and synthesized novel 2,6,9-trisubstituted purines as potent CDK12 inhibitors showing strong, equipotent antiproliferative activity against trastuzumab-sensitive HER2+ SK-Br3 cells and trastuzumab-resistant HER2+ HCC1954 cells (GI_50_ values < 50 nM) both of which express a high level of CDK12. Two potent analogue **30d** and **30e** at 40, 200 nM greatly downregulated the levels of cyclinK and Pol II p-CTD (Ser2), as well as the expression of CDK12 downstream genes (IRS1 and WNT1) in a dose-dependent manner. We also observed structure-property relationship for a subset of potent analogues, and found that **30e** is highly stable in liver microsomes with lack of CYP inhibition. In addition, **30d** exhibited a synergy with trastuzumab in the both cells, suggesting that our inhibitors could be applied to alleviate trastuzumab-resistance of HER2+ breast cancers and escalate the efficacy of trastuzumab as well. Our study may provide insight into developing a novel therapy for HER2+ breast cancers.

## 1. Introduction

Breast cancers are the most prevalent cancers in the world, and categorized into several subtypes based on genetic background and biomarker profiles [1]. So called HER2-positive (HER2+) breast cancers, in which human epidermal growth factor receptor 2 (*HER2*) oncogene is amplified in cancer genome, account for 15–20% of breast cancers. The HER2 overexpression is associated with the aggressiveness of breast cancers [2,3]. Accordingly, anti-HER2 monoclonal antibody such as trastuzumab is used as a first-line treatment for metastatic HER2+ breast cancers. Both progression-free survival and overall survival of HER2+ breast-cancer patients were significantly enhanced when trastuzumab was administered in combination with chemotherapy [4,5]. However, more than a half of HER2+ breast-cancer patients responded poorly to trastuzumab [6]. Studies revealed a number of molecular mechanisms associated with the trastuzumab resistance [7,8], including (i) HER2 mutation defective in binding with trastuzumab (i.e., HER2 truncation), (ii) upregulation of HER2 downstream signalings (i.e., PTEN loss), (iii) activation of alternative signaling pathways (i.e., IGF1R stimulation), and (iv) failure to trigger antibody-mediated anti-cancer immunity (i.e., FcγRIII F158 polymorphism). Thus, the development of a new drug offsetting the resistance mechanisms is warranted.

**Figure 1:**
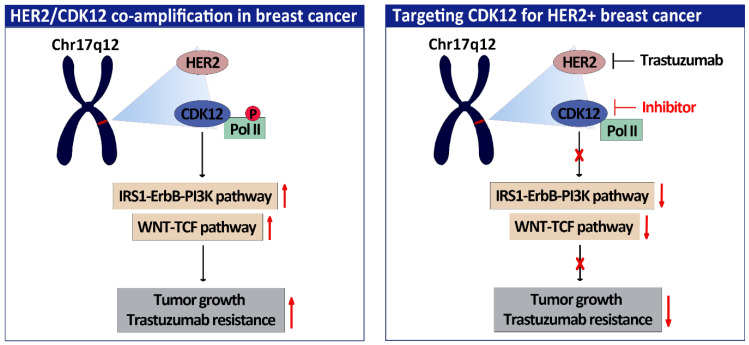
CDK12 is an attractive therapeutic target for HER2+ breast cancers. CDK12 and HER2 are co-amplified on chromosome 17q12 in HER2+ breast-cancer patients and involved in tumor growth and trastuzumab resistance through upregulation of IRS1-ErbB-PI3K and WNT-TCF pathways.

The *HER2* gene is located on chromosome 17q12. It was reported that multiple other genes at 17q12 are also amplified in HER2+ breast cancers, some of which are crucial for growth and survival of breast cancers [9]. Recently, we discovered that cyclin-dependent kinase 12 (*CDK12*) as a co-amplified gene on 17q12 is involved in tumorigenesis and trastuzumab resistance in HER2+ breast cancers, proposing CDK12 an attractive therapeutic target to escalate the therapeutic activity of trastuzumab and overcome the trastuzumab resistance (Figure 1) [10]. *CDK12* is a transcription-associated CDK-family kinase, and requires binding with cyclinK for activation. CDK12/cyclinK phosphorylates the *C*-terminal domain (CTD) of RNA PolII at Ser2, which, in turn, promotes transcriptional elongation, plays roles in RNA splicing, and regulates the expression of the genes involved in the DNA damage response and replication [11].

**Figure 2:**
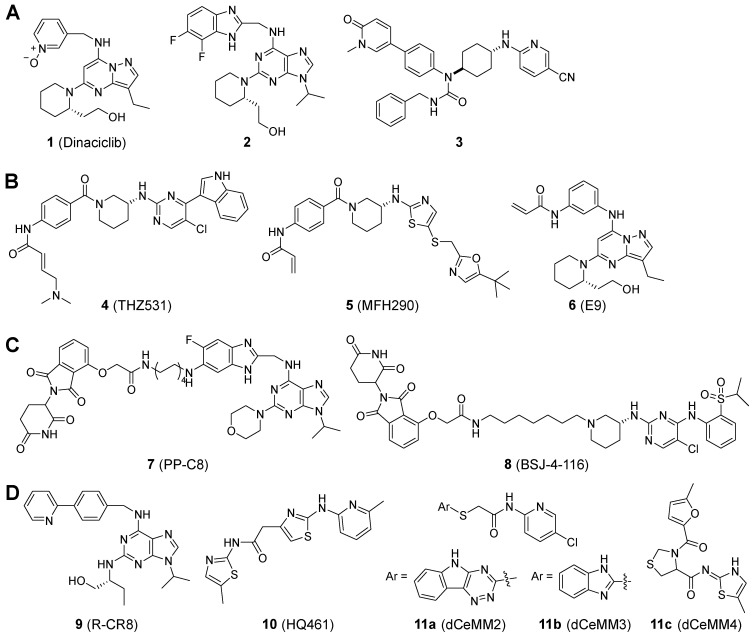
Structures of the published CDK12 inhibitors (**A**) non-covalent CDK12 inhibitors, (**B**) irreversible CDK12 inhibitors, (**C**) CDK12 inhibitor-based PROTACs, and (**D**) CDK12 inhibitors acting as cyclinK degraders.

Several CDK12 inhibitors and their anti-cancer activities have been reported (Figure 2) [12]. Dinaciclib (SCH 727965) was developed as a potent inhibitor of CDK-family kinases [13], and later it was revealed that dinaciclib shows a potent inhibitory activity against CDK12 as well [14]. In the clinical phase II trial for advanced breast cancers, dinaciclib alone showed only a marginal efficacy [15]. However, preclinical studies suggested that dinaciclib might be able to confer a significant efficacy for selected patient cohorts [10]. Additionally, pan-CDK inhibitors such as dinaciclib, selective CDK12 inhibitors (**2**, **3**) were unveiled from a rational designing approach [16,17]. Gray group reported irreversible CDK12 inhibitors such as THZ531 (**4**) [18], MFH290 (**5**) [19], and E9 (**6**) [20], whose electrophilic acrylamide moiety formed a covalent bond with the unique cysteine located in *C*-terminal region of CDK12/13 (Cys1039 for CDK12, Cys1017 for CDK13). These irreversible inhibitors exhibited prominent anti-cancer phenotypes in cancer cells owing to their highly selective, strong, persistent suppression of CDK12/13, but their in vivo activity could not be evaluated due to their poor stability in vivo. Recently two different CDK12 inhibitor-based PROTACs (PP-C8, BSJ-4-116) were reported, which were derived from **2** and THZ531 (**4**), respectively, [21,22]. Interestingly, unlike **2** and **4** showing a similar potency against CDK12 and CDK13, the both CDK12 degraders discriminated CDK12 over CDK13 for degradation in cells, providing an approach to achieve CDK12 selectivity.

Additionally, a new type of CDK12 inhibitors were reported, including R-CR8 (**9**) [23], HQ461 (**10**) [24], and dCeMM2/3/4 (**11a**/**b**/**c**) [25] which act as molecular glues promoting CDK12–DDB1 interaction. The inhibitor-bound CDK12 recruits DDB1, an adaptor protein of Cul4-uibiqutin ligase, through the terminal moiety of the inhibitors (i.e., the pyridyl group in R-CR8, 5-methylthiazol-2-amine group in HQ461). In the drug-induced complex, CDK12 behaves as a substrate binding protein of DDB1–Cul4 E3 ligase, provoking ubiquitination and the subsequent proteasomal degradation of cyclinK. CyclinK degradation significantly enhanced inhibitors’ activity to suppress the function of CDK12 in cancer cells. Herein, we wish to report novel potent purine scaffold CDK12 inhibitors acting as a cyclinK degrader that potently suppressed a growth of HER2+ breast cancers. Our inhibitors also deteriorated the growth of traszutumab-resistant HER2+ breast cancers with a similar potency. This study may provide an insight into designing potent CDK12 inhibitors for HER2+ breast cancers.

## 2. Results and Discussion

### 2.1. Inhibitor Design

**Figure 3:**
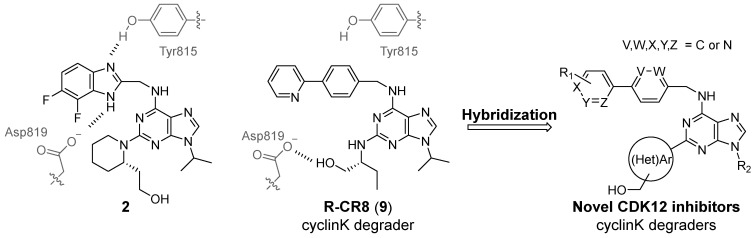
Design of novel purine-scaffold CDK12 inhibitors.

We designed novel 2,6,9-trisubstituted purine scaffold CDK12 inhibitors by hybridizing the reported purine-based CDK12 inhibitor (**2**) [17] and the cyclinK degrader R-CR8 (**9**) [23] (Figure 3). The X-ray co-crystal structures displayed two unique hydrogen bond interactions between the imidazole part of **2** and the side chains of Tyr815 and Asp819, which are positioned near the phenyl group of R-CR8. Thus, we replaced the phenyl group of R-CR8 with a pyridyl group, on which a nitrogen atom could be engaged with a hydrogen bond with Tyr815 or Asp819. We also introduced various heteroaryl or aryl moieties to 2 position, to which hydroxyalkyl moieties were attached to emulate the hydroxyalkyl group of CR-8. We expected that any heteroatom at 2 position might be involved in the interaction with nearby residues such as Asp819.

### 2.2. Structure and Activity Relationship

We synthesized a variety of 2,6,9-trisubstitued purine analogues, and measured their inhibitory activity against CDK12/cyclinK enzyme and growth-inhibitory activity against two different CDK12 overexpressing HER2+ breast-cancer cell lines (trastuzumab-sensitive SK-Br3 cells, trastuzumab-resistant HCC1954 cells).

**Scheme 1:**
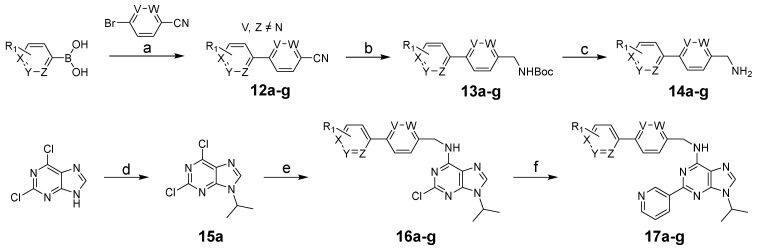
Synthetic scheme for bipyridyl methaneamines (**14a–g**) and **17a–g**. Reagents and conditions: (a) Pd(PPh_3_)_4_, K_2_CO_3,_ 1,4-dioxane, 100 °C, 55–96% yield. (b) NiCl_2_∙6H_2_O, Boc_2_O, NaBH_4_, MeOH, 0 °C to rt. (c) HCl, rt, 24–70% combined yield for (b) and (c). (d) isopropylbromide, K_2_CO_3_, DMSO, rt, 40% yield. (e) 14a-g, TEA, MeOH, 50 °C, 55–69% yield. (f) 3-pyridylboronic acid, Pd(PPh_3_)_4_, K_2_CO_3,_ 1,4-dioxane, 100 °C, 63–83% yield.

At first, we investigated the structure-activity relationship (SAR) for 6 position. A series of bipyridyl methaneamines were incorporated to 6 position, while 2 and 9 positions were fixed with 3-pyridyl and isopropyl group, respectively, (Scheme 1). A set of biaryl carbonitriles (**12a–g**) were prepared using Suzuki coupling reactions between respective pyridyl boronic acids and bromoaryl nitriles. The cyano group was then converted to aminomethylene group (**14a–g**) using NiCl_2_/Boc-mediated reduction and the subsequent Boc deprotection [26]. To synthesize the desired products **17a–g**, commercially available 2,6-dichloropurine was alkylated at 9 position with isopropyl group, and aminated with various bipyridyl methanamines (**14a–g**) at 6 position. The resulting intermediates (**16a–g**) were conjugated with 3-pyridyl group at 2 position through Suzuki coupling reactions.

Compounds **17a–e** showed similar IC_50_ values against CDK12/cyclinK, indicating that the terminal pyridyl group at 6 position is not involved in binding with CDK12 as in the case of CR-8 (Table 1). Interestingly, the N2 on the inner pyridine ring at 6 position increased CDK12 inhibitory activity 2–3 times (**17f** vs. **17b**, **17g** vs. **17d**), suggesting a potential hydrogen bond between the N2 and a nearby residue within CDK12 as we anticipated. In addition, although a terminal pyridyl group at 6 position made almost no contribution to the enzymatic activity, particular terminal groups present in **17a** and **17e** substantially augmented cell growth inhibitory activities against the both cells.

**Figure 4:**
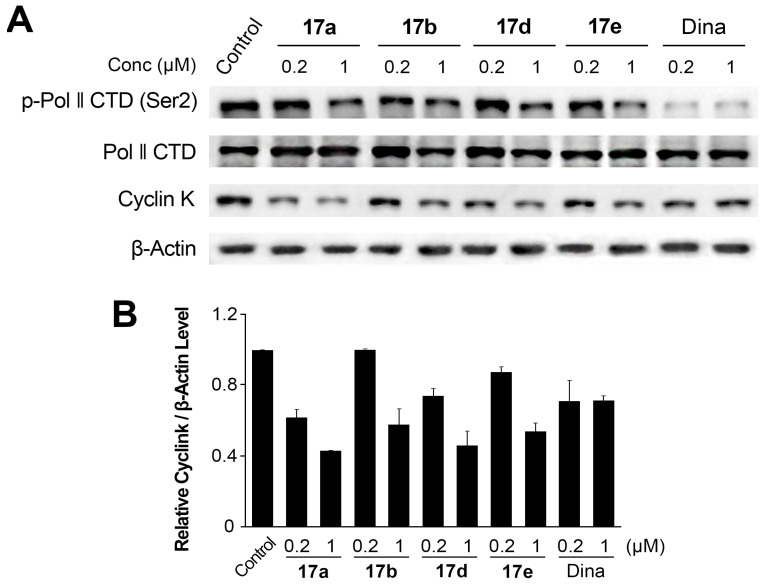
Four compounds (**17a**, **17b**, **17d**, **17e**), which contain different terminal pyridyl groups at C6 position but exhibit similar in vitro CDK12/cyclinK activity, downregulated Pol II p-CTD (Ser2) and cyclinK in a dose-dependent manner. Compounds at indicated concentrations were treated to SK-Br3 cells for 2 h. (**A**) Western blot analysis result. (**B**) Quantitation of relative cyclinK levels normalized to β-actin levels using ImageJ software. Control = DMSO. Dina = dinaciclib.

To test whether the significant improvement of cellular activity from **17a** and **17e** originated from cyclinK degradation, we selected four analogues (**17a**/**b**/**d**/**e**) showing similar in vitro CDK12 potency, and compared their ability in downregulating the levels of cyclinK and PolII p-CTD (Ser2) in SK-Br3 cells (Figure 4). All of the four compounds at 0.2, 1 μM downregulated cyclinK in a dose-dependent manner, and among them, **17a** induced the most prominent cyclinK degradation. However, all four compounds exhibited a similar level of PolII p-CTD (Ser2) suppression, suggesting that the higher growth inhibitory activity of **17a** and **17e** might originate from off-target effects irrespective of CDK12 or cyclinK. Conversely, dinaciclib at 0.2, 1 μM strongly suppressed PolII p-CTD, but showed a marginal activity in downrgulating cyclinK in cells.

**Scheme 2:**
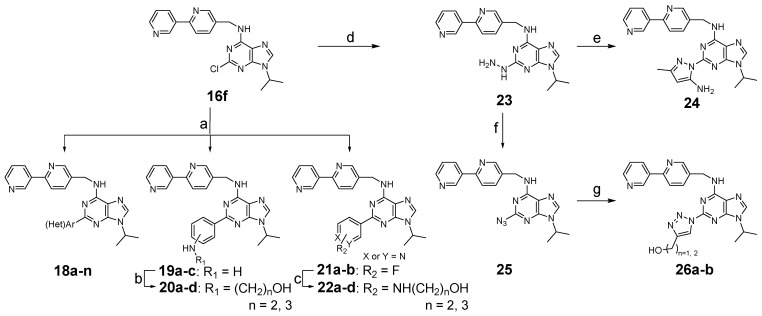
Derivatization at C2 position. Reagents and conditions: (a) Boronic acid, Pd(PPh_3_)_4_, K_2_CO_3,_ 1,4-dioxane, 100 °C, 12–78% yield. (b) Br(CH_2_)_n_OH, TEA, 110–120 °C, *n*-BuOH, 30–34% yield. (c) NH_2_(CH_2_)_n_OH, TEA, 110–120 °C, *n*-BuOH, 40–52% yield. (d) NH_2_NH_2_, *n*-BuOH, 150 °C, 48% yield. (e) 3-oxobutanenitrile, EtOH, reflux, 26% yield. (f) NaNO_2_/HCl, 0–5 °C, then NaN_3_, 40% yield. (g) CuSO_4_∙5H_2_O, sodium ascorbate, *n*-BuOH:H_2_O (1:1), rt, 30–40% yield.

Next, we investigated SAR for 2 position. A variety of aryl or heteroaryl groups were attached to 2 position of the intermediate **16f** to afford **18a–n**, **19a–c**, and **21a–b** (Scheme 2). The aniline NH_2_ group of **19a–c** was subjected to alkylation to produce *N*-hydroxyalkyl analogues **20a–d**. Additionally, α-fluoro pyridyl compounds **21a–b** reacted with hydroxyalkyl amines to generate **22a–d**. The chloro group of **16f** was also replaced with hydrazine group (**23**), which then reacted with 3-oxobutanenitrile to form an aminopyrazol-containing analogue **24**. Additionally, **23** was transformed to an azido intermediate (**25**) through sequential reactions using NaNO_2_/HCl and NaN_3_, which then underwent click chemistry to generate **26a–b** containing hydroxyalkyl triazoles at 2 position.

The SAR result in Table 2 showed that *m*-amino and *p*-amino group of the phenyl at 2 position augmented CDK12 inhibitory activity 3–4 times (**19b** vs. **18a**, **19c** vs. **18a**). A similar level of improvement was observed from their isosteres such as indole (**18d**) and indazole (**18e**, **18f**). *N*-hydroxypropyl modification of the aniline groups (**20b**, **20c**, **20d**) diminished the inhibitory activity, indicating that this variation might be sterically unfavorable for binding with CDK12.

Noticeably, the SAR result in Table 3 indicated that 6-membered heteroaryl moieties at 2 position significantly improved inhibitory activity. Compounds containing aminopyridyl (**18i**, **18j**), pyridyl (**18k**), or aminopyrimidyl (**18l**) group at 2 position showed a profound inhibition of CDK12/cyclinK with IC_50_ values < 100 nM. Their inhibitory activity was tolerated by *N*-hydroxyethyl group (**22a**, **22c**), but was compromised by *N*-hydroxypropyl modification (**22b**, **22d**). Unlike the 6-membered heteroaryl groups, 5-membered heteroaryl groups based on pyrazole (**18m**, **18n**, **24**) or triazole (**26a**, **26b**) did not improve CDK12 inhibition.

**Scheme 3:**
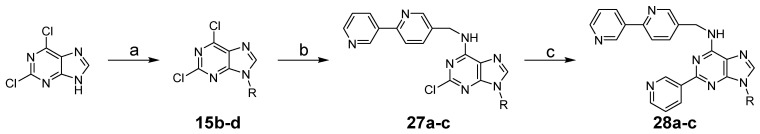
Derivatization at 9 position. Reagents and conditions: (a) R-Br, K_2_CO_3_, DMSO, rt, 40–70% yield or R-OH, PPh_3_, DIAD, THF, rt, 72% yield. (b) **14f**, TEA, MeOH, 50 °C, 40–60% yield. (c) 3-pyridylboronic acid, Pd(PPh_3_)_4_, K_2_CO_3,_ 1,4-dioxane, 100 °C, 23–48% yield.

In order to study SAR for 9 position, we prepared the analogues, which have 3-pyridyl at 2 position and **14g** at 6 position in common, but different alkyl groups at 9 position (Scheme 3). The SAR result in Table 4 showed that the ethyl group (**28a**) conferred a significantly better CDK12 inhibitory activity (CDK12/cyclinK IC_50_ = 16 nM) and cell growth inhibitory activity compared with isopropyl group (**17f**) (CDK12/cyclinK IC_50_ = 221 nM). However, replacement with a cyclopentyl group (**28b**) slightly decreased the activity and that with mopholinyl group (**28c**) eliminated inhibitory activity, suggesting that the pocket around the 9 position prefers a small-size moiety such as an ethyl group and does not accommodate the bulky groups.

**Scheme 4:**
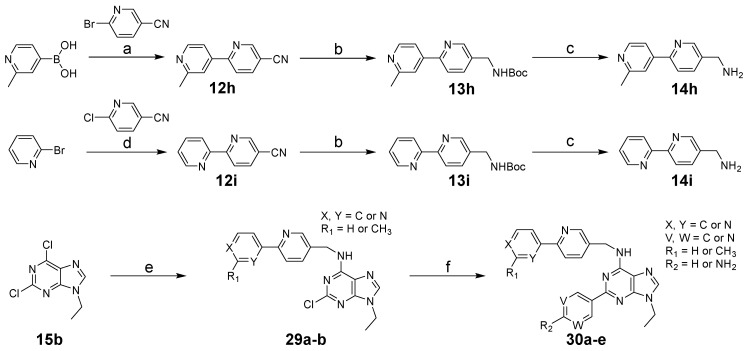
Synthesis of potent analogues. Reagents and conditions: (a) Pd(PPh_3_)_4_, K_2_CO_3,_ 1,4-dioxane, 100 °C, 50–60% yield. (b) NiCl_2_∙6H_2_O, Boc_2_O, NaBH_4_, MeOH, 0 °C to rt. (c) HCl, rt, 45–55% combined yield for (b) and (c). (d) *i*PrMgCl, ZnCl_2_, Pd_2_(dba)_3_, XPhos, THF, 65 °C, 65% yield. (e) **14h** or **14i**, TEA, MeOH, 50 °C, 50–72% yield. (f) boronic acid/ester, Pd(PPh_3_)_4_, K_2_CO_3,_ 1,4-dioxane, 100 °C, 20–55% yield.

Having the comprehensive SAR results in hands, we finally synthesized several analogues that were anticipated to have potent activities against both CDK12/cyclinK enzyme and cell growth inhibition. We fixed 9 position with the ethyl group and introduced the selected heteroaryl groups at 2 position, conferring a potent CDK12/cyclinK inhibition (Table 3 and Table 4). Additionally, two different bipyridyl methaneamines (**14h**, **14i**) were incorporated at 6 position that were expected to confer relatively more effective in CDK12 inhibition and cell-growth inhibition based on the results in Table 1. Synthesis was carried out using the same synthetic routes as before, but for 2,2′-bipyridyl carbonitrile (**12i**) that was prepared using a modified Negishi coupling reaction [27,28] (Scheme 4). Compounds **30d** (CDK12/cyclinK IC_50_ = 21 nM) and **30e** (CDK12/cyclinK IC_50_ = 85 nM) containing the 2′-pyridyl terminal moiety at 6 position showed less potent enzymatic activity compared with **28a**, **30a**, **30b** (IC_50_ values < 30 nM), but showed 2–3 fold higher activity in cancer cell-growth inhibition (GI_50_ values = 34–52 nM) (Table 5), which is consistent with the SAR trend shown in Table 1.

### 2.3. Docking Analysis

**Figure 5:**
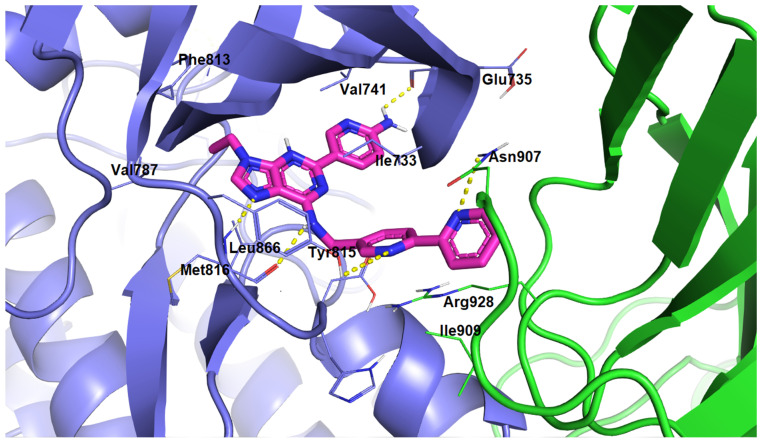
Docking result of **30d** in CDK12–DDB1 complex. Docking was performed based on the X-ray co-crystal structure of R-CR8 (pdb id: 6td3), and visualized using Pymol2.5 software (Schrödinger, New York, NY, USA). CDK12, DDB1, and **30d** are colored in cyan, green, and purple, respectively. The labeled residues are predicted to interact with **30d**. Predicted hydrogen bonds are highlighted in yellow dotted lines.

We performed a docking analysis for **30d** using the reported co-crystal structure of R-CR8-bound CDK12–DDB1 complex [23] (Figure 5). As shown in R-CR8, hydrogen bonds were expected between a pair of N7 and NH on the purine ring and the hinge Met816 backbone. The Ethyl group at 9 position sits in the small hydrophobic pocket created by three hydrophobic side chains of Val787, Phe813, and Leu866. The aminopyridine group at 2 position forms a hydrogen bond with the carbonyl backbone of Glu735, as well as hydrophobic interactions with Ile733 and Val741. The inner pyridine at 6 position is predicted to form a hydrogen bond with the side chain of Tyr815 and a hydrophobic contact with Ile733. In addition, the terminal pyridine at 6 position interacts with DDB1 through a hydrogen bond with Asn907 and hydrophobic interactions with the hydrophobic side chains of Ile909 and Arg928.

### 2.4. In Vitro Kinome-Wide Inhibition Profiling of **30d**

**Figure 6:**
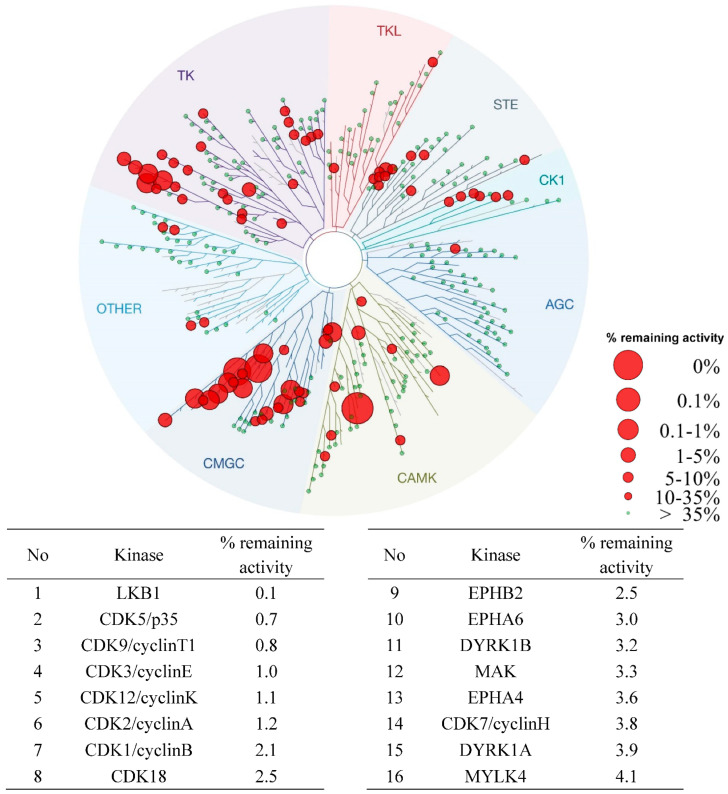
Kinome–wide inhibition profiling of **30d** at 10 μM against a panel of 359 human wild-type kinases. (**Top**) percent remaining activity of each kinase illustrated using a web–based TREE*spot*^TM^ visualization tool (https://www.discoverx.com/services/drug–discovery–development–services/treespot–data–analysis, accessed on 1 March 2022). (**Bottom**) the list of top-ranking kinases showing >95% inhibition by 10 μM of **30d**.

We also investigated the kinome-wide selectivity of **30d** at 10 μM against a panel of human kinases (Figure 6 and Supplementary Table S1). The profiling result suggested that **30d** has a similar kinome-wide inhibition profile to that of dinaciclib [29] (Supplementary Figure S1). Among 359 human wild-type kinases examined, 16 kinases were strongly inhibited (more than 95%) by 10 μM of **30d**, including CDK1/cyclinB, CDK2/cyclinA, CDK3/cyclinE, CDK5/p35, CDK7/cyclinH, CDK9/cyclinT1, CDK12/cyclinK, CDK18, EPHA4, EPHA6, EPHB2, DYRK1A, DYRK1B, LKB1, MAK, and MYLK4. This result indicated that **30d** is a pan-CDK inhibitor capable of potently inhibiting multiple other CDKs, as well as CDK12/cyclinK. Interestingly, **30d** showed only a marginal inhibition against CDK4/6 (Supplementary Table S1), which on the contrary are highly sensitive to dinaciclib [29,30]. In addition, **30d** potently inhibited several EPH-family tyrosine kinases, none of which is targeted by dinaciclib (Supplementary Figure S1). Unlike a selective CDK12 inhibitor THZ531, which exhibited a selective activity against SK-Br3 (GI_50_ = 30 nM) over HCC1954 cells (GI_50_ = 195 nM), the equipotent growth-inhibitory activity of **30d** (SK-Br3 GI_50_ = 46 nM, HCC1954 GI_50_ = 36 nM) and dinaciclib (SK-Br3 GI_50_ = 12 nM, HCC1954 GI_50_ = 12 nM) against both breast-cancer cell lines might be owing to their ability to simultaneously inhibit multiple CDK-family kinases. Thus, targeting multiple CDKs along with CDK12/cyclinK may be advantageous to overcoming trastuzumab resistance. Moreover, this kinome-wide inhibition result suggested that our inhibitors could be expanded to a drug discovery campaign against other important kinases including EPH-family tyrosine kinases.

### 2.5. Liver Microsomal Stability and CYP Inhibition

We then examined the five potent analogues in Table 4 for in vitro metabolic stability in liver microsomes from three different species (human, dog, mouse) and for inhibitory activity against the 5 representative cytochrome P450 enzymes (CYPs) (Table 6). We found that for liver microsomal stability, the 2′-pyridyl group is more suitable than α-methyl-4′-pyridyl group as the terminal aromatic group at 6 position (**30d** vs. **30a**, **30e** vs. **30b**), and the aminopyrimidyl group is superior to the aminopyridyl group as a substituent at 2 position (**30b** vs. **30a**, **30e** vs. **30d**). The five analogues showed a similar, desirable CYP inhibition profile except for CYP3A4, suggesting that they could be readily used in combination with other agents. Among them, the analogues containing aminopyrimidine group at 2 position (**30b**, **30e**) exhibited only a slight inhibition against all 5 CYPs, implying that the aminopyrimidine is the most suitable substituent at 2 position to avoid the inhibition of CYPs. Together, **30e** was the best analogue in terms of in vitro liver metabolic stability and CYPs’ activity conservation.

### 2.6. Suppression of Cyclink and PolII CTD Phosphorylation

**Figure 7:**
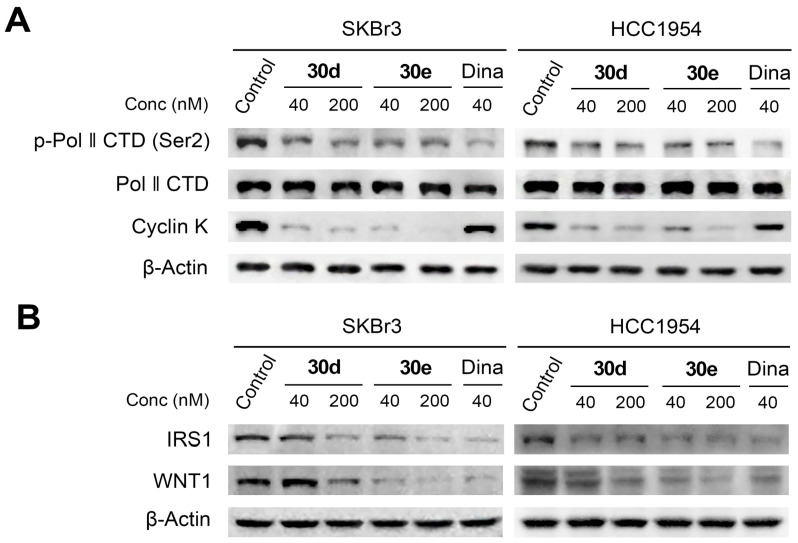
Both **30d** and **30e** showed a potent, dose-dependent downregulation of (**A**) cyclinK and p-Pol II CTD (Ser2), and (**B**) the expressions of CDK12 downstream genes (IRS1 and WNT1) in SK-Br3 and HCC1954 cells. Compounds were treated at 40 and 200 nM for (**A**) 2 h or (**B**) 24 h; then, Western blot analysis was performed. Control = DMSO, Dina = dinaciclib.

We examined the intracellular target inhibition in SK-Br3 and HCC1954 cells after treatment of potent analogues **30d** and **30e** at 40 and 200 nM for 2 h (Figure 7A). In both cells, cyclinK was greatly downregulated in a dose-dependent manner, suggesting that both compounds act as a potent cyclinK degrader. Additionally, **30d** and **30e** showed a strong, dose-dependent suppression of the levels of PolII p-CTD (Ser2). Compared with **30d** and **30e**, dinaciclib at 40 nM showed only a marginal downregulation of cyclinK, but exhibited a more potent inhibition of PolII CTD phosphorylation in both cells. We also examined the expression levels of CDK12 downstream genes (IRS1 and WNT1) [10] following treatment of the same doses of compounds for 24 h (Figure 7B). Both **30d** and **30e** showed a strong suppression of IRS1 and WNT1, but **30e** was more prominent downregulation as comparable to dinaciclib.

### 2.7. Synergism between **30d** and Trastuzumab

**Figure 8:**
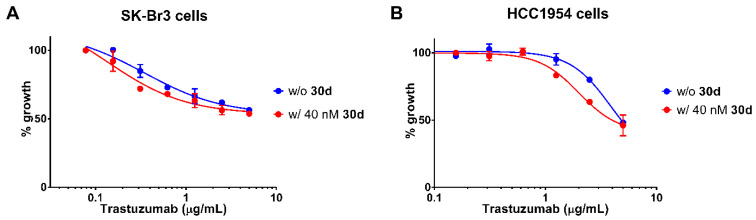
Synergism between **30d** and trastuzumab in (**A**) SK-Br3 cells and (**B**) HCC1954 cells. Antiproliferative activity of trastuzumab was slightly enhanced in both cells by co-treatment of 40 nM of **30d**. The blue line indicates the titration of trastuzumab only, and the red line indicates the titration of trastuzumab in the presence of 40 nM **30d**.

Additionally, we examined a combination effect in both SK-Br3 and HCC1954 cells. We treated multiple doses of trastuzumab for 72 h in the absence or presence of **30d** at a single dose (40 nM) around its GI_50_ value (Figure 8). We observed a slight gain in the inhibitory activity of trastuzumab when it was co-treated with **30d** in both cell lines, demonstrating a modest level of synergism between **30d** and trastuzumab in growth inhibition of HER2+ breast cancer cells, regardless of their trastuzumab sensitivity.

## 3. Materials and Methods

### 3.1. Chemistry

All reagents and solvents purchased from commercial sources were used without further purification. Nuclear magnetic resonance (NMR) solvents were purchased from Cambridge Isotope Laboratories Inc. All reactions were monitored under UV light (254 nm) using a thin layer chromatography on pre-coated silica gel glass plates from Merck. Flash column chromatography was performed using silica gel (Kieselgel 60 Art. 9385, 230–400 mesh) from Merck. ^1^H and ^13^C NMR spectra were recorded on Bruker 400 MHz FT-NMR. Chemical shifts are reported in parts per million (ppm, δ) using peaks from an NMR solvent (CDCl_3_, CD_3_OD, or DMSO-*d*_6_) as a reference. Coupling constants (*J*) are reported in Hertz (Hz), and the multiplicities of peaks are abbreviated as s: singlet, br: broad singlet, d: doublet, t: triplet, q: quartet, dd: doublet of doublet, dt: doublet of triplet, and m: multiplet. High-resolution mass-spectral results were obtained using Orbitrap Eclipse™ Tribrid™ Mass Spectrometer (ThermoFisher Scientific, Waltham, MA, USA).

#### 3.1.1. General Procedure for Synthesis of **12a–h**

To a stirred solution of corresponding aryl bromide (1 eq.), pyridyl boronic acid/ester (1.2 eq.), and Pd(PPh_3_)_4_ (0.05 eq.) in 1,4-dioxane (45 mL) under N_2_ atmosphere, was added 5 mL of a 2 M aq. K_2_CO_3_ solution. The vigorously stirred mixture was heated to 100 °C for 12 h. After cooling, the mixture was combined with EtOAc, and washed with water and brine. The organic layer was dried over MgSO_4_, filtered, and concentrated under a reduced pressure. The residue was purified by column chromatography (*n*-hexane:EtOAc = 9:1) to afford bipyridine carbonitriles as white solids.

##### [2,3′-Bipyridine]-6′-carbonitrile (**12a**)

Yield 96%; ^1^H NMR (400 MHz, CDCl_3_) δ 9.31 (d, *J* = 1.7 Hz, 1H), 8.77 (d, *J* = 4.5 Hz, 1H), 8.50 (dd, *J* = 8.1, 2.2 Hz, 1H), 7.91–7.78 (m, 3H), 7.38 (ddd, *J* = 7.1, 4.8, 1.3 Hz, 1H); ^13^C NMR (101 MHz, DMSO-*d*_6_) δ 151.86, 150.12, 149.19, 137.70, 137.13, 135.19, 132.39, 129.07, 124.33, 121.72, 117.53, 40.15, 39.94, 39.73, 39.52, 39.52, 39.31, 39.10, 38.89; HRMS (ESI+) *m*/*z* calcd. for C_11_H_7_N_3_ [M + H]^+^ 182.0718, found 182.0721.

##### [3,3′-Bipyridine]-6-carbonitrile (**12b**)

Yield 59%; ^1^H NMR (400 MHz, DMSO-*d_6_*) δ 9.17 (d, *J* = 1.9 Hz, 1H), 9.05 (d, *J* = 2.0 Hz, 1H), 8.69 (dd, *J* = 4.8, 1.4 Hz, 1H), 8.45 (dd, *J* = 8.1, 2.3 Hz, 1H), 8.30–8.24 (m, 1H), 8.18 (d, *J* = 8.2 Hz, 1H), 7.59–7.54 (m, 1H); ^13^C NMR (101 MHz, DMSO-*d*_6_) δ 150.18, 149.42, 148.20, 136.27, 135.77, 134.95, 131.80, 131.08, 129.13, 124.07, 117.51, 40.15, 39.94, 39.73, 39.52, 39.52, 39.31, 39.10, 38.90.; HRMS (ESI+) *m*/*z* calcd. for C_11_H_7_N_3_ [M + H]^+^ 182.0718, found 182.0721.

##### 6′-Methyl-[3,3′-bipyridine]-6-carbonitrile (**12c**)

Yield 50%; ^1^H NMR (400 MHz, CDCl_3_) δ 8.86 (dd, *J* = 2.2, 0.7 Hz, 1H), 8.68 (d, *J* = 2.1 Hz, 1H), 7.94 (dd, *J* = 8.1, 2.3 Hz, 1H), 7.74 (ddd, *J* = 8.0, 5.1, 1.6 Hz, 2H), 7.26 (d, *J* = 8.1 Hz, 1H), 2.58 (s, 3H). ^13^C NMR (101 MHz, CDCl_3_ + CD_3_OD) δ 159.55, 149.14, 147.01, 136.77, 135.27, 135.03, 132.56, 132.23, 131.99, 131.89, 128.96, 128.70, 128.66, 128.54, 124.06, 116.96, 24.48; HRMS (ESI+) *m*/*z* calcd. for C_12_H_9_N_3_ [M + H]^+^: 196.0869, found: 196.0881.

##### [3,4′-Bipyridine]-6-carbonitrile (**12d**)

Yield 55%; ^1^H NMR (400 MHz, CDCl_3_) δ 8.99 (d, *J* = 1.6 Hz, 1H), 8.79 (d, *J* = 5.1 Hz, 2H), 8.07 (dd, *J* = 8.0, 2.1 Hz, 1H), 7.84 (d, *J* = 8.0 Hz, 1H), 7.53 (d, *J* = 5.3 Hz, 2H); ^13^C NMR (101 MHz, DMSO-*d*_6_) δ 150.54, 149.51, 142.45, 136.04, 132.70, 129.22, 121.71, 117.39, 40.15, 39.94, 39.73, 39.52, 39.52, 39.31, 39.10, 38.89; HRMS (ESI+): *m*/*z* calcd. for C_11_H_7_N_3_ [M + H]^+^ 182.0718, found 182.0721.

##### 2′-Methyl-[3,4′-bipyridine]-6-carbonitrile (**12e**)

Yield 60%; ^1^H NMR (400 MHz, CDCl_3_) δ 8.89 (d, *J* = 1.6 Hz, 1H), 8.65–8.52 (m, 1H), 8.05–7.92 (m, 1H), 7.76 (dd, *J* = 8.1, 0.7 Hz, 1H), 7.36–7.24 (m, 2H), 2.60 (s, 3H). ^13^C NMR (101 MHz, CDCl_3_) δ 159.87, 150.23, 149.47, 143.57, 137.38, 135.26, 133.79, 132.13, 132.03, 128.57, 128.45, 118.77, 116.97, 24.63.

##### [2,3′-Bipyridine]-5-carbonitrile (**12f**)

Yield 66%; ^1^H NMR (400 MHz, DMSO-*d*_6_) δ 9.34 (d, *J* = 1.7 Hz, 1H), 9.14 (d, *J* = 1.5 Hz, 1H), 8.71 (dd, *J* = 4.7, 1.4 Hz, 1H), 8.52 (dd, *J* = 8.0, 1.5 Hz, 1H), 8.46 (dd, *J* = 8.3, 2.1 Hz, 1H), 8.30 (d, *J* = 8.3 Hz, 1H), 7.58 (dd, *J* = 8.0, 4.8 Hz, 1H); ^13^C NMR (101 MHz, DMSO-*d*_6_) δ 157.04, 152.72, 151.15, 148.35, 141.21, 134.67, 132.50, 123.98, 120.68, 117.08, 108.12, 40.15, 40.15, 39.94, 39.94, 39.73, 39.73, 39.52, 39.52, 39.52, 39.31, 39.31, 39.10, 39.10, 38.89, 38.89.

##### [2,4′-Bipyridine]-6-carbonitrile (**12g**)

Yield 67%; ^1^H NMR (400 MHz, CDCl_3_) δ 9.01 (d, *J* = 1.9 Hz, 1H), 8.81 (s, 2H), 8.11 (dd, *J* = 8.0, 1.7 Hz, 1H), 7.95 (d, *J* = 7.8 Hz, 3H); ^13^C NMR (101 MHz, CDCl_3_) δ 157.87, 152.81, 150.79, 144.54, 140.51, 121.37, 120.64, 116.51, 109.92, 77.48, 77.36, 77.16, 77.16, 76.84; HRMS (ESI+) *m*/*z* calcd. for C_11_H_7_N_3_ [M + H]^+^ 182.0718, found 182.0725.

##### 2′-Methyl-[2,4′-bipyridine]-5-carbonitrile (**12h**)

Yield 60%; ^1^H NMR (400 MHz, CDCl_3_) δ 8.90 (d, *J* = 1.6 Hz, 1H), 8.58 (dd, *J* = 11.2, 3.3 Hz, 1H), 8.01–7.93 (m, 1H), 7.75 (dd, *J* = 8.1, 0.7 Hz, 1H), 7.34–7.22 (m, 2H), 2.59 (d, *J* = 8.7 Hz, 3H). ^13^C NMR (101 MHz, CDCl_3_) δ 159.67, 158.14, 152.64, 150.02, 144.76, 140.32, 120.87, 120.60, 118.36, 116.45, 109.66, 24.57; HRMS (ESI+) *m*/*z* calcd. for C_12_H_9_N_3_ [M + H]^+^: 196.0869, found: 196.0881.

##### [2,2′-Bipyridine]-5-carbonitrile (**12i**) 

**12i** was obtained following the literature procedure [28].

#### 3.1.2. General Procedure for Synthesis of **14a–i**

Intermediate **12a–i** (ca. 20 g) was added to methanol (120 mL) and cooled to 0 °C. Di-*tert*-butyl dicarbonate (2 eq.) was added and the suspension was stirred for 15 m. Then, NiCl_2_∙6H_2_O (0.3 eq.) was added and stirred for 5 m. Next, NaBH_4_ (3.5 eq.) was added in portion-wise for 30 m. After the addition was completed (ca. 30 m), the ice bath was removed and the mixture was stirred with warming to rt overnight. After the reaction was completed, diethylenetriamine (1 eq.) was added to the stirring mixture. After 15 min, methanol was evaporated and we added 100 mL of aq. NaHCO_3_. After the extraction, using EtOAc (3 × 80 mL), the organic layer was dried over MgSO_4_, evaporated under a reduced pressure, and subjected to flash chromatography (2% methanol in dichloromethane) to afford a Boc-protected intermediate (**13a–i**). Then, the intermediate was dissolved in 50 mL of dichloromethane and cooled to 4 °C, to which we slowly added 10 mL of 4*N* HCl in 1,4-dioxane and stirred for 1 h at rt. The eluted solid in dichloromethane was filtered and dried to afford a light brown salt as a pure product (**14a–i**).

##### [2,3′-Bipyridin]-6′-ylmethanamium (**14a**)

Yield 48%; ^1^H NMR (400 MHz, DMSO-*d*_6_) δ 9.33 (d, *J* = 2.0 Hz, 1H), 8.81 (d, *J* = 5.0 Hz, 1H), 8.72 (s, 2H), 8.61 (dd, *J* = 8.2, 2.3 Hz, 1H), 8.23 (ddd, *J* = 13.7, 9.4, 4.8 Hz, 2H), 7.77 (d, *J* = 8.2 Hz, 1H), 7.67 (dd, *J* = 8.9, 3.6 Hz, 1H), 4.29 (q, *J* = 5.7 Hz, 2H); ^13^C NMR (101 MHz, DMSO-*d*_6_) δ 154.79, 150.14, 146.87, 145.77, 142.79, 137.21, 129.93, 125.36, 124.00, 123.31, 42.14, 40.15, 39.94, 39.73, 39.52, 39.52, 39.31, 39.10, 38.89.

##### [3,3′-Bipyridine]-6-ylmethanamium (**14b**)

Yield 44%; ^1^H NMR (400 MHz, DMSO-*d*_6_) δ 9.40 (d, *J* = 1.9 Hz, 1H), 9.14 (d, *J* = 2.1 Hz, 1H), 8.95 (t, *J* = 6.5 Hz, 2H), 8.73 (s, 3H), 8.43 (dd, *J* = 8.2, 2.4 Hz, 1H), 8.15 (dd, *J* = 8.1, 5.6 Hz, 1H), 7.76 (d, *J* = 8.2 Hz, 1H), 4.27 (q, *J* = 5.8 Hz, 2H); ^13^C NMR (101 MHz, DMSO-*d*_6_) δ 154.42, 147.29, 142.82, 141.69, 140.70, 136.14, 135.27, 129.47, 127.10, 122.94, 42.45, 40.15, 39.94, 39.73, 39.52, 39.52, 39.31, 39.10, 38.89.

##### (6′-Methyl-[3,3′-bipyridin]-6-yl)methanamine (**14c**)

Yield 56%; ^1^H NMR (400 MHz, DMSO) δ 9.28 (s, 1H), 9.18 (s, 1H), 8.99–8.84 (m, 4H), 8.55–8.47 (m, 1H), 8.09 (d, *J* = 8.4 Hz, 1H), 7.85 (d, *J* = 8.2 Hz, 1H), 4.29 (q, *J* = 5.3 Hz, 2H), 2.86 (s, 3H). ^13^C NMR (101 MHz, DMSO-*d_6_*) δ 154.40, 153.55, 146.99, 143.90, 139.15, 137.05, 133.14, 129.66, 128.62, 123.77, 42.62, 19.15.

##### [3,4′-Bipyridine]-6-ylmethanamium (**14d**)

Yield 24%; ^1^H NMR (400 MHz, DMSO-*d*_6_) δ 9.19 (s, 1H), 8.90 (d, *J* = 5.7 Hz, 2H), 8.45 (d, *J* = 6.0 Hz, 4H), 8.22 (d, *J* = 5.5 Hz, 2H), 7.71 (d, *J* = 8.0 Hz, 1H), 4.32 (q, J = 5.9 Hz, 2H); ^13^C NMR (101 MHz, DMSO-*d_6_*) δ 156.30, 152.35, 148.04, 142.38, 136.56, 129.75, 124.26, 123.05, 42.59, 40.15, 39.94, 39.73, 39.52, 39.52, 39.31, 39.10, 38.89.

##### (2′-Methyl-[3,4′-bipyridin]-6-yl)methanamine (**14e**)

Yield 60%; ^1^H NMR (400 MHz, DMSO-*d_6_*) δ 9.26 (d, *J* = 2.1 Hz, 1H), 8.90 (d, *J* = 6.3 Hz, 1H), 8.70 (bs, 3H), 8.54 (dd, *J* = 8.2, 2.1 Hz, 1H), 8.50 (s, 1H), 8.37 (d, *J* = 5.0 Hz, 1H), 7.80 (d, *J* = 8.2 Hz, 1H), 4.32 (q, *J* = 11.3, 5.5 Hz, 2H), 2.84 (s, 3H).

##### [2,3′-Bipyridine]-5-ylmethanamium (**14f**)

Yield 47%; ^1^H NMR (400 MHz, DMSO-*d*_6_) δ 9.51 (d, *J* = 2.0 Hz, 1H), 9.09 (d, *J* = 8.3 Hz, 1H), 8.92 (dd, *J* = 5.5, 1.3 Hz, 1H), 8.89 (d, *J* = 1.7 Hz, 1H), 8.68 (s, 3H), 8.31 (d, *J* = 8.2 Hz, 1H), 8.20 (dd, *J* = 8.2, 2.3 Hz, 1H), 8.07 (dd, *J* = 8.1, 5.5 Hz, 1H), 4.17 (q, *J* = 5.8 Hz, 2H); ^13^C NMR (101 MHz, DMSO-*d*_6_) δ 150.66, 149.87, 142.90, 141.76, 139.82, 139.02, 136.71, 131.30, 127.54, 121.34, 40.15, 39.94, 39.73, 39.52, 39.52, 39.31, 39.10, 38.89.

##### [2,4′-Bipyridine]-6-ylmethanamium (**14g**)

Yield 45%; ^1^H NMR (400 MHz, DMSO-*d*_6_) δ 9.05 (d, *J* = 6.8 Hz, 2H), 8.98 (d, *J* = 1.8 Hz, 1H), 8.93 (s, 3H), 8.74 (d, *J* = 6.8 Hz, 2H), 8.49 (d, *J* = 8.2 Hz, 1H), 8.31 (dd, *J* = 8.2, 2.1 Hz, 1H), 4.19 (q, J = 5.8 Hz, 2H); ^13^C NMR (101 MHz, DMSO-*d*_6_) δ 150.66, 149.87, 142.90, 141.76, 139.82, 139.02, 136.71, 131.30, 127.54, 121.34, 40.15, 39.94, 39.73, 39.52, 39.52, 39.31, 39.10, 38.89.

##### (2′-Methyl-[2,4′-bipyridin]-5-yl)methanamine (**14h**)

Yield 70%; ^1^H NMR (400 MHz, DMSO-*d*_6_) δ 8.98 (d, *J* = 1.8 Hz, 1H), 8.90 (d, *J* = 6.3 Hz, 1H), 8.81 (bs, 3H), 8.67 (s, 1H), 8.55 (dd, *J* = 6.3, 1.5 Hz, 1H), 8.47 (d, *J* = 8.2 Hz, 1H), 8.29 (dd, *J* = 8.2, 2.2 Hz, 1H), 4.21 (q, *J* = 5.6 Hz, 2H), 2.85 (d, *J* = 6.9 Hz, 3H).

#### 3.1.3. General Procedure for Synthesis of **15a–c**

Both 2,6-dichloro-9*H*-purine (10.0 g, 53.2 mmol) and K_2_CO_3_ (21.9 g, 159 mmol) were dissolved in 70 mL anhydrous DMSO. Alkylbromide (133 mmol) was added dropwise to the reaction mixture at rt, and stirred overnight. Upon completion of the reaction, the reaction mixture was poured into ice water and extracted with EtOAc, and dried over MgSO_4_. The concentrated mixture was subjected to a column chromatography using *n*-hexane/EtOAc (3:1) as eluent. Pure products were obtained with yield 40–70% based on alkyl groups.

##### Procedure for Synthesis of 2,6-Dichloro-9-(tetrahydro-2*H*-pyran-4-yl)-9*H*-purine (**15d**)

A mixture of 2,6-dichloro-9*H*-purine (0.56 g, 3 mmol), 4-hydroxytetrahydropyran (0.455g, 4.5mmol), and Ph_3_P (1.18 g, 4.5 mmol) in THF (20 mL) was stirred at rt for 1 h under N_2_ atmosphere, then was added DIAD (0.909 g, 4.5 mmol) dropwise under ice-bath. The resulting mixture was stirred at rt over 2 days. The solvent was evaporated and the residue was purified by column chromatography (*n*-hexane:EtOAc:CH_2_Cl_2_ = 1:1:0.4) to give 15d (0.505 g, 72% yield). 

#### 3.1.4. General Procedure for Synthesis of **16a–g**, **27a–c**, **29a–b**

To solution of 2,6-dichloro-9-alkylpurines (**15a–d**) (37.7 mmol) in methanol (20 mL), we added *n*,*n*′-bipyridin-5-ylmethanaminiums (**14a–j**) (1.2 eq.) and Et_3_N (3 eq.) under N_2_ atmosphere at rt. The reaction mixture was heated at 50 °C for 12 h. After the reaction was completed, methanol was evaporated. The mixture was diluted with EtOAc and was then washed with saturated aq. NaHCO_3_, water, and brine, sequentially. The organic layer was dried over MgSO_4_, filtered, and concentrated. The residue was purified by a column chromatography (2–3% methanol in dichloromethane) to provide the light brown solid products.

##### *N*-([2,3′-Bipyridin]-6′-ylmethyl)-2-chloro-9-isopropyl-9*H*-purin-6-amine (**16a**)

Yield 69%; ^1^H NMR (400 MHz, CDCl_3_) δ 9.09 (d, *J* = 1.7 Hz, 1H), 8.67 (d, *J* = 4.2 Hz, 1H), 8.26 (dd, *J* = 8.1, 2.2 Hz, 1H), 7.85 (s, 1H), 7.81 (s, 1H), 7.74 (td, *J* = 7.7, 1.7 Hz, 1H), 7.69 (d, *J* = 7.9 Hz, 1H), 7.46 (d, *J* = 8.2 Hz, 1H), 7.26–7.18 (m, 1H), 4.97 (s, 2H), 4.78 (dt, *J* = 13.5, 6.8 Hz, 1H), 1.54 (d, *J* = 6.8 Hz, 6H); ^13^C NMR (101 MHz, CDCl_3_) δ 157.02, 155.15, 154.55, 154.22, 150.11, 147.50, 138.03, 137.04, 135.19, 133.82, 122.88, 122.14, 120.53, 119.18, 77.48, 77.36, 77.16, 77.16, 76.84, 47.01, 45.45, 22.86; HRMS (ESI+) *m*/*z* calcd. for C_19_H_18_ClN_7_ [M + H]^+^ 380.1390, found 380.1391.

##### *N*-([3,3′-Bipyridin]-6-ylmethyl)-2-chloro-9-isopropyl-9*H*-purin-6-amine (**16b**)

Yield 65%; ^1^H NMR (400 MHz, CDCl_3_) δ 8.79 (d, *J* = 1.8 Hz, 1H), 8.75 (d, *J* = 1.8 Hz, 1H), 8.62 (dd, *J* = 4.8, 1.3 Hz, 1H), 7.87 (s, 1H), 7.83 (dt, *J* = 8.1, 2.5 Hz, 2H), 7.74 (s, 1H), 7.49 (d, *J* = 8.1 Hz, 1H), 7.38 (dd, *J* = 7.7, 4.9 Hz, 1H), 4.97 (s, 2H), 4.84–4.75 (dt, J = 13.5, 6.8 Hz, 1H), 1.55 (d, *J* = 6.8 Hz, 6H); ^13^C NMR (101 MHz, CDCl_3_) δ 156.59, 155.13, 154.22, 149.35, 148.15, 147.44, 138.05, 135.28, 134.44, 133.28, 132.38, 123.88, 122.42, 119.20, 77.48, 77.36, 77.16, 77.16, 76.84, 47.05, 45.38, 22.87, 22.70; HRMS (ESI+) *m*/*z* calcd. for C_19_H_18_ClN_7_ [M + H]^+^ 380.1390, found 380.1392.

##### 2-Chloro-9-isopropyl-*N*-((6′-methyl-[3,3′-bipyridin]-6-yl)methyl)-9*H*-purin-6-amine (**16c**)

^1^H NMR (400 MHz, CDCl_3_) δ 8.80 (d, *J* = 1.7 Hz, 1H), 8.74 (d, *J* = 2.1 Hz, 1H), 7.91–7.84 (m, 2H), 7.81 (dd, *J* = 8.0, 2.4 Hz, 1H), 7.50 (d, *J* = 8.1 Hz, 1H), 7.38 (s, 1H), 7.30 (d, *J* = 7.9 Hz, 1H), 5.01 (s, 1H), 4.85 (dq, *J* = 13.3, 6.7 Hz, 1H), 2.66 (s, 3H), 1.61 (d, *J* = 6.8 Hz, 6H). ^13^C NMR (101 MHz, CDCl_3_) δ 158.22, 147.29, 147.21, 137.84, 135.05, 134.84, 132.40, 130.38, 123.53, 46.98, 24.11, 22.83; HRMS (ESI+) *m*/*z* calcd for C_20_H_20_ClN_7_ [M + H]^+^: 394.1547, found: 394.1551.

##### *N*-([3,4′-Bipyridin]-6-ylmethyl)-2-chloro-9-isopropyl-9*H*-purin-6-amine (**16d**)

Yield 63%; ^1^H NMR (400 MHz, DMSO-*d_6_*) δ 8.92 (s, 1H), 8.74 (s, 1H), 8.70–8.65 (m, 2H), 8.31 (s, 1H), 8.08 (d, *J* = 8.2 Hz, 1H), 8.02 (d, *J* = 6.1 Hz, 2H), 7.91 (d, *J* = 8.1 Hz, 1H), 4.71 (d, *J* = 5.5 Hz, 2H), 4.67 (d, *J* = 6.7 Hz, 2H), 1.49 (d, *J* = 6.7 Hz, 6H); ^13^C NMR (101 MHz, CDCl_3_) δ 155.59, 155.14, 148.08, 139.38, 137.44, 124.03, 77.48, 77.36, 77.16, 76.84, 47.69, 22.85; HRMS (ESI+) *m*/*z* calcd. for C_19_H_18_ClN_7_ [M + H]^+^ 380.1390, found 380.1394.

##### 2-Chloro-9-isopropyl-*N*-((2′-methyl-[3,4′-bipyridin]-6-yl)methyl)-9*H*-purin-6-amine (**16e**)

^1^H NMR (400 MHz, CDCl_3_) δ 8.75 (d, *J* = 1.7 Hz, 1H), 8.60 (d, *J* = 4.8 Hz, 1H), 7.85 (dd, *J* = 8.1, 2.2 Hz, 1H), 7.76 (dd, *J* = 10.6, 8.4 Hz, 3H), 7.66 (d, *J* = 5.1 Hz, 1H), 6.84 (s, 1H), 4.92 (s, 1H), 4.81 (dq, *J* = 13.5, 6.8 Hz, 1H), 2.66 (s, 3H), 1.58 (d, *J* = 6.8 Hz, 6H). ^13^C NMR (101 MHz, CDCl_3_) δ 159.10, 154.14, 149.64, 149.57, 146.43, 137.98, 136.69, 120.74, 118.20, 47.12, 24.49, 22.75; HRMS (ESI+) *m*/*z* calcd. for C_20_H_20_ClN_7_ [M + H]^+^: 394.1547, found: 394.1547.

##### *N*-([2,3′-Bipyridin]-5-ylmethyl)-2-chloro-9-isopropyl-9*H*-purin-6-amine (**16f**)

Yield 50%; ^1^H NMR (400 MHz, CDCl_3_) δ 9.15 (s, 1H), 8.70 (d, *J* = 1.6 Hz, 1H), 8.63 (s, 1H), 8.27 (d, *J* = 8.0 Hz, 1H), 7.79 (dd, *J* = 8.1, 2.2 Hz, 1H), 7.70 (s, 1H), 7.67 (d, *J* = 8.1 Hz, 1H), 7.37 (dd, *J* = 7.7, 4.8 Hz, 1H), 6.93 (s, 1H), 4.85 (d, *J* = 18.7 Hz, 2H), 4.78 (dt, *J* = 13.6, 6.8 Hz, 1H), 1.53 (d, *J* = 6.8 Hz, 6H); ^13^C NMR (101 MHz, CDCl_3_) δ 155.13, 154.14, 150.00, 149.76, 148.22, 138.03, 136.79, 134.38, 133.27, 123.72, 120.40, 119.02, 77.48, 77.36, 77.16, 77.16, 76.84, 47.18, 41.87, 22.84; HRMS (ESI+) *m*/*z* calcd. for C_19_H_18_ClN_7_ [M + H]^+^ 380.1390, found 380.1394.

##### *N*-([2,4′-Bipyridin]-5-ylmethyl)-2-chloro-9-isopropyl-9*H*-purin-6-amine (**16g**)

Yield 55%; ^1^H NMR (400 MHz, CDCl_3_) δ 8.80 (s, 1H), 8.23 (s, 1H), 7.87 (dd, *J* = 31.8, 7.4 Hz, 3H), 6.84 (s, 1H), 4.93 (s, 2H), 4.84 (dt, *J* = 13.4, 6.6 Hz, 1H), 1.58 (d, *J* = 6.7 Hz, 6H); ^13^C NMR (101 MHz, CDCl_3_) δ 155.03, 152.41, 150.22, 137.15, 135.46, 121.31, 121.31, 77.48, 77.48, 77.36, 77.16, 77.16, 77.16, 76.84, 47.43, 22.88; HRMS (ESI+) *m*/*z* calcd. for C_19_H_18_ClN_7_ [M + H]^+^ 380.1390, found 380.1395.

##### *N*-([2,3′-Bipyridin]-5-ylmethyl)-2-chloro-9-ethyl-9*H*-purin-6-amine (**27a**)

^1^H NMR (400 MHz, CDCl_3_) δ 9.18 (s, 1H), 8.74 (d, *J* = 1.7 Hz, 1H), 8.67 (d, *J* = 3.5 Hz, 1H), 8.32 (d, *J* = 8.0 Hz, 1H), 7.84 (dd, *J* = 8.1, 2.2 Hz, 1H), 7.76–7.66 (m, 2H), 7.42 (dd, *J* = 7.8, 4.8 Hz, 1H), 6.81 (s, 1H), 4.91 (s, 2H), 4.22 (q, *J* = 7.3 Hz, 2H), 1.52 (t, *J* = 7.3 Hz, 3H). ^13^C NMR (101 MHz, CDCl_3_) δ 154.08, 149.90, 149.69, 148.11, 139.90, 136.72, 134.34, 123.65, 120.34, 38.99, 15.57; HRMS (ESI+) *m*/*z* calcd. for C_18_H_16_ClN_7_ [M + H]^+^: 366.1234, found: 366.1232.

##### *N*-([2,3′-Bipyridin]-5-ylmethyl)-2-chloro-9-cyclopentyl-9*H*-purin-6-amine (**27b**)

^1^H NMR (400 MHz, CDCl_3_) δ 9.16 (s, 1H), 8.71 (d, *J* = 1.1 Hz, 1H), 8.65 (d, *J* = 3.9 Hz, 1H), 8.34–8.24 (m, 1H), 7.80 (dd, *J* = 8.1, 2.2 Hz, 1H), 7.72–7.63 (m, 2H), 7.39 (dd, *J* = 7.9, 4.8 Hz, 1H), 7.20 (t, *J* = 5.4 Hz, 1H), 4.86 (dt, *J* = 14.5, 7.2 Hz, 3H), 2.33–2.17 (m, 2H), 1.85 (d, *J* = 15.1 Hz, 4H), 1.79–1.69 (m, 2H). ^13^C NMR (101 MHz, CDCl_3_) δ 153.95, 149.87, 149.62, 148.08, 138.44, 136.66, 134.42, 134.30, 123.62, 120.26, 55.83, 32.88, 23.76; HRMS (ESI+) *m*/*z* calcd. for C_21_H_20_ClN_7_ [M + H]^+^: 406.1543, found: 406.1543.

##### *N*-([2,3′-Bipyridin]-5-ylmethyl)-2-chloro-9-(tetrahydro-2*H*-pyran-4-yl)-9*H*-purin-6-amine (**27c**)

^1^H NMR (400 MHz, CDCl_3_) δ 9.11 (s, 1H), 8.69 (d, *J* = 1.7 Hz, 1H), 8.59 (s, 1H), 8.25 (d, *J* = 8.0 Hz, 1H), 7.79 (dd, *J* = 8.1, 2.0 Hz, 1H), 7.73 (s, 1H), 7.67 (d, *J* = 8.1 Hz, 1H), 7.34 (dd, *J* = 7.9, 4.8 Hz, 1H), 6.39 (s, 1H), 4.84 (s, 2H), 4.69–4.57 (m, 1H), 4.06 (dd, *J* = 8.7, 5.7 Hz, 2H), 3.59–3.48 (m, 2H), 2.02 (dt, *J* = 18.8, 6.6 Hz, 4H). ^13^C NMR (101 MHz, CDCl_3_) δ 154.18, 149.87, 148.09, 137.89, 134.42, 120.42, 66.93, 51.19, 33.22; HRMS (ESI+) *m*/*z* calcd. for C_21_H_20_ClN_7_O [M + H]^+^: 422.1496, found: 422.1500.

##### 2-Chloro-9-ethyl-*N*-((2′-methyl-[2,4′-bipyridin]-5-yl)methyl)-9*H*-purin-6-amine (**29a**)

^1^H NMR (400 MHz, CDCl_3_) δ 8.74 (d, *J* = 1.3 Hz, 1H), 8.60 (d, *J* = 5.2 Hz, 1H), 7.83 (dd, *J* = 8.1, 2.1 Hz, 1H), 7.77 (s, 1H), 7.74 (s, 1H), 7.71 (d, *J* = 8.4 Hz, 1H), 7.64 (d, *J* = 5.2 Hz, 1H), 6.90 (t, *J* = 5.9 Hz, 1H), 4.91 (s, 1H), 4.20 (q, *J* = 7.3 Hz, 2H), 2.65 (s, 3H), 1.50 (t, *J* = 7.3 Hz, 3H). ^13^C NMR (101 MHz, CDCl_3_) δ 159.19, 154.21, 149.73, 149.61, 146.27, 139.92, 136.68, 120.70, 120.56, 118.15, 38.99, 24.57, 15.55.; HRMS (ESI+) *m*/*z* calcd. for C_19_H_18_ClN_7_ [M + H]^+^: 380.1390, found: 380.1396.

##### *N*-([2,2′-Bipyridin]-5-ylmethyl)-2-chloro-9-ethyl-9*H*-purin-6-amine (**29b**)

Yield 72%; ^1^H NMR (400 MHz, DMSO-*d_6_*) δ 8.74 (d, *J* = 1.7 Hz, 1H), 8.67 (dd, *J* = 4.7, 0.7 Hz, 1H), 8.43–8.29 (m, 3H), 8.04–7.86 (m, 3H), 7.43 (ddd, *J* = 7.5, 4.8, 1.0 Hz, 1H), 4.79 (d, *J* = 5.7 Hz, 2H), 4.49 (q, *J* = 7.1 Hz, 2H), 1.39 (t, *J* = 7.2 Hz, 3H). ^13^C NMR (101 MHz, DMSO-*d_6_*) δ 161.41, 155.60, 154.49, 152.89, 151.19, 149.71, 149.17, 146.44, 137.72, 136.87, 135.68, 124.51, 120.81, 120.56, 110.59, 42.30, 17.73. HRMS (ESI+) *m*/*z* calcd. for C_18_H_16_ClN_7_ [M + H]^+^: 366.1234, found: 366.1222.

#### 3.1.5. General Procedure for Synthesis of **17a–g**, **18a–n**, **19a–c**, **21a–b**, **28a–c**, **30a–e**

To a stirred solution of 2,6,9-trisubstituted purine intermediates (**16a–h**, **27a–c**, **29a–b**) (0.236 mmol), respective aryl boronic acids/esters (1.2 eq.), and Pd(PPh_3_)_4_ (0.05 eq.) in 1,4-dioxane (2 mL) under a N_2_ atmosphere, we added 0.5 mL of a 2 M aq. K_2_CO_3_ solution. The vigorously stirred mixture was warmed to 100 °C for 12 h. After cooling, the mixture was diluted with EtOAc and washed with water and brine. The organic layer was dried over MgSO_4_, and concentrated. The residue was purified by silica column chromatography using 4~5% methanol in dichloromethane as an eluent to afford desired products.

##### *N*-([2,3′-Bipyridin]-6′-ylmethyl)-9-isopropyl-2-(pyridin-3-yl)-9*H*-purin-6-amine] (**17a**)

Yield 83%; ^1^H NMR (400 MHz, CDCl_3_) δ 9.66 (s, 1H), 9.13 (d, *J* = 1.9 Hz, 1H), 8.69 (d, *J* = 8.0 Hz, 1H), 8.66 (d, *J* = 4.3 Hz, 1H), 8.61 (d, *J* = 3.7 Hz, 1H), 8.23 (dd, *J* = 8.2, 2.2 Hz, 1H), 7.87 (s, 1H), 7.77–7.69 (m, 1H), 7.67 (d, *J* = 7.9 Hz, 1H), 7.51 (d, *J* = 8.2 Hz, 1H), 7.45 (s, 1H), 7.34 (dd, *J* = 7.8, 4.8 Hz, 1H), 7.22 (ddd, *J* = 7.1, 4.8, 1.2 Hz, 1H), 5.14 (s, 2H), 4.88 (dt, *J* = 13.5, 6.8 Hz, 1H), 1.63 (d, *J* = 6.8 Hz, 6H); ^13^C NMR (101 MHz, CDCl_3_) δ 158.43, 156.51, 154.67, 154.44, 150.06, 149.91, 149.76, 147.60, 138.43, 137.00, 135.71, 135.10, 134.57, 133.58, 123.21, 122.79, 121.76, 120.51, 119.74, 77.48, 77.36, 77.16, 77.16, 76.84, 47.27, 45.78, 22.77; HRMS (ESI+) *m*/*z* calcd. for C_24_H_22_N_8_ [M + H]^+^ 423.2046, found 423.2046.

##### *N*-([3,3′-Bipyridin]-6-ylmethyl)-9-isopropyl-2-(pyridin-3-yl)-9*H*-purin-6-amine] (**17b**)

Yield 80%; ^1^H NMR (400 MHz, CDCl_3_) δ 9.68 (s, 1H), 8.83 (dd, *J* = 5.1, 2.0 Hz, 2H), 8.73 (d, *J* = 8.0 Hz, 1H), 8.68–8.60 (m, 2H), 7.92–7.82 (m, 3H), 7.55 (d, *J* = 8.1 Hz, 1H), 7.43–7.35 (m, 2H), 6.93 (s, 1H), 5.16 (s, 2H), 4.99–4.88 (m, 1H), 1.67 (d, *J* = 6.8 Hz, 6H); ^13^C NMR (101 MHz, CDCl_3_) δ 158.14, 156.55, 154.37, 150.15, 149.92, 149.16, 148.04, 147.38, 138.40, 135.35, 135.07, 134.27, 133.25, 131.98, 123.75, 123.07, 121.97, 119.61, 77.48, 77.16, 76.84, 47.19, 45.64, 22.69; HRMS (ESI+) *m*/*z* calcd. for C_24_H_22_N_8_ [M + H]^+^ 423.2046, found 423.2046.

##### 9-Isopropyl-*N*-((6′-methyl-[3,3′-bipyridin]-6-yl)methyl)-2-(pyridin-3-yl)-9*H*-purin-6-amine (**17c**)

Yield: 63%; ^1^H NMR (400 MHz, CDCl_3_) δ 9.61 (s, 1H), 8.72 (d, *J* = 1.9 Hz, 1H), 8.68–8.61 (m, 2H), 8.56 (d, *J* = 3.6 Hz, 1H), 7.80 (s, 1H), 7.75 (dd, *J* = 8.1, 2.2 Hz, 1H), 7.69 (dd, *J* = 8.0, 2.3 Hz, 1H), 7.46 (d, *J* = 8.1 Hz, 1H), 7.29 (dd, *J* = 7.9, 4.8 Hz, 1H), 7.17 (s, 1H), 6.94 (s, 1H), 5.08 (s, 2H), 4.91–4.80 (m, 1H), 2.54 (s, 3H), 1.60 (d, *J* = 6.8 Hz, 6H). ^13^C NMR (101 MHz, CDCl_3_) δ 158.20, 157.28, 156.64, 150.23, 150.01, 147.41, 147.38, 138.25, 135.40, 134.97, 134.67, 132.24, 130.40, 123.40, 123.07, 121.90, 47.23, 24.18, 22.74. HRMS (ESI+) *m*/*z* calcd. for C_25_H_24_N_8_ [M + H]^+^: 437.2202, found: 437.2206.

##### *N*-([3,4′-Bipyridin]-6-ylmethyl)-9-isopropyl-2-(pyridin-3-yl)-9*H*-purin-6-amine] (**17d**)

Yield 67%; ^1^H NMR (400 MHz, CDCl_3_) δ 9.67 (s, 1H), 8.83 (s, 1H), 8.77 (d, *J* = 7.8 Hz, 1H), 8.68 (d, *J* = 20.1 Hz, 3H), 7.89 (d, *J* = 13.8 Hz, 4H), 7.78 (d, *J* = 8.0 Hz, 1H), 7.44 (s, 1H), 6.40 (s, 1H), 5.07 (s, 2H), 4.94 (dd, *J* = 13.3, 6.4 Hz, 1H), 1.67 (d, *J* = 6.7 Hz, 6H); ^13^C NMR (101 MHz, CDCl_3_) δ 158.73, 154.24, 153.84, 149.90, 149.66, 148.25, 138.82, 138.09, 136.56, 134.67, 134.45, 134.31, 129.81, 128.33, 128.22, 123.64, 120.39, 119.21, 77.48, 77.36, 77.16, 76.84, 47.17, 22.82; HRMS (ESI+) *m*/*z* calcd. for C_24_H_22_N_8_ [M + H]^+^ 423.2046, found 423.2043.

##### 9-Isopropyl-*N*-((2′-methyl-[3,4′-bipyridin]-6-yl)methyl)-2-(pyridin-3-yl)-9*H*-purin-6-amine (**17e**)

Yield 67%; ^1^H NMR (400 MHz, CDCl_3_) δ 9.61 (d, *J* = 1.4 Hz, 1H), 8.79 (d, *J* = 1.8 Hz, 1H), 8.64 (dt, *J* = 7.9, 1.9 Hz, 1H), 8.57 (dd, *J* = 4.8, 1.7 Hz, 1H), 8.51 (d, *J* = 5.2 Hz, 1H), 7.84–7.79 (m, 2H), 7.47 (t, *J* = 6.5 Hz, 1H), 7.33–7.27 (m, 2H), 7.24 (d, *J* = 5.2 Hz, 1H), 6.78 (s, 1H), 5.09 (s, 2H), 4.86 (dd, *J* = 13.6, 6.8 Hz, 1H), 2.57 (s, 3H), 1.60 (t, *J* = 7.1 Hz, 6H). ^13^C NMR (101 MHz, CDCl_3_) δ 159.26, 158.49, 156.59, 154.31, 150.17, 149.94, 149.82, 147.51, 145.39, 138.32, 135.43, 135.06, 134.36, 132.69, 123.10, 121.90, 121.05, 119.69, 118.69, 47.25, 24.56, 22.73. HRMS (ESI+) *m*/*z* calcd. for C_25_H_24_N_8_ [M + H]^+^: 437.2202, found: 437.2193.

##### *N*-([2,3′-Bipyridin]-5-ylmethyl)-9-isopropyl-2-(pyridin-3-yl)-9*H*-purin-6-amine (**17f**)

Yield 66%; ^1^H NMR (400 MHz, CDCl_3_) δ 9.66 (s, 1H), 9.15 (s, 1H), 8.76 (d, *J* = 1.3 Hz, 1H), 8.73–8.48 (m, 3H), 8.25 (d, *J* = 7.9 Hz, 1H), 7.84 (dd, *J* = 8.1, 1.9 Hz, 1H), 7.80 (s, 1H), 7.66 (d, *J* = 8.1 Hz, 1H), 7.40 (d, *J* = 36.2 Hz, 2H), 6.76 (s, 1H), 5.02 (s, 2H), 4.89 (dt, *J* = 13.5, 6.8 Hz, 1H), 1.62 (t, *J* = 10.5 Hz, 6H); ^13^C NMR (101 MHz, CDCl_3_) δ 156.72, 154.35, 153.96, 150.34, 149.96, 149.63, 148.26, 138.46, 136.54, 135.50, 134.62, 134.33, 134.16, 123.67, 123.24, 120.42, 119.60, 77.48, 77.16, 76.84, 47.41, 41.87, 22.80; HRMS (ESI+) *m*/*z* calcd. for C_24_H_22_N_8_ [M + H]^+^ 423.2046, found 423.2042.

##### *N*-([2,4′-Bipyridin]-5-ylmethyl)-9-isopropyl-2-(pyridin-3-yl)-9*H*-purin-6-amine] (**17g**)

Yield 82%; ^1^H NMR (400 MHz, CDCl_3_) δ 9.66 (s, 1H), 8.82 (s, 1H), 8.77–8.67 (m, 3H), 8.65 (s, 1H), 7.87 (d, *J* = 12.6 Hz, 4H), 7.76 (d, *J* = 8.0 Hz, 1H), 7.41 (s, 1H), 6.55 (s, 1H), 5.05 (s, 2H), 4.96–4.88 (m, 1H), 1.66 (d, *J* = 6.6 Hz, 6H); ^13^C NMR (101 MHz, CDCl_3_) δ 156.45, 154.30, 153.69, 150.19, 149.74, 149.38, 146.51, 138.64, 136.63, 136.09, 135.29, 123.51, 121.20, 120.87, 77.48, 77.36, 77.16, 76.84, 47.55, 22.85; HRMS (ESI+) *m*/*z* calcd. for C_24_H_22_N_8_ [M + H]^+^ 423.2046, found 423.2041.

##### *N*-([2,3′-Bipyridin]-5-ylmethyl)-9-isopropyl-2-phenyl-9*H*-purin-6-amine (**18a**)

Yield: 30%; ^1^H NMR (400 MHz, CDCl_3_) δ 9.20 (d, *J* = 1.7 Hz, 1H), 8.86 (d, *J* = 1.8 Hz, 1H), 8.67 (dd, *J* = 4.8, 1.5 Hz, 1H), 8.52 (dd, *J* = 7.8, 1.8 Hz, 2H), 8.39–8.28 (m, 1H), 8.01–7.92 (m, 1H), 7.89 (s, 1H), 7.75 (d, *J* = 8.1 Hz, 1H), 7.55–7.36 (m, 4H), 5.13 (s, 2H), 4.98 (dd, *J* = 13.6, 6.8 Hz, 1H), 1.70 (d, *J* = 6.8 Hz, 6H). ^13^C NMR (101 MHz, CDCl_3_) δ 158.66, 154.13, 153.85, 149.86, 149.63, 148.21, 138.76, 138.05, 136.52, 134.32, 134.24, 129.72, 128.25, 128.13, 123.57, 120.36, 47.09, 22.76.; HRMS (ESI+) *m*/*z* calcd. for C_25_H_23_N_7_ [M + H]^+^: 422.2093, found: 422.2097.

##### 3-(6-(([2,3′-Bipyridin]-5-ylmethyl)amino)-9-isopropyl-9*H*-purin-2-yl)benzamide (**18b**)

Yield 37%; ^1^H NMR (400 MHz, DMSO-*d*_6_) δ 9.21 (s, 1H), 8.90 (s, 1H), 8.83 (s, 1H), 8.57 (dd, *J* = 26.9, 6.3 Hz, 3H), 8.38 (d, *J* = 8.1 Hz, 1H), 8.33 (s, 1H), 8.10 (s, 1H), 8.01 (s, 2H), 7.93 (d, *J* = 7.7 Hz, 1H), 7.56 (t, *J* = 7.8 Hz, 1H), 7.49 (dd, *J* = 8.0, 4.7 Hz, 1H), 7.45 (s, 1H), 4.88 (dd, *J* = 13.5, 6.8 Hz, 3H), 1.60 (d, *J* = 6.8 Hz, 6H). ^13^C NMR (101 MHz, DMSO-*d*_6_) δ 168.55, 165.52, 152.81, 150.16, 148.13, 140.08, 135.07, 134.40, 134.26, 124.21, 121.13, 120.82, 116.88, 46.93, 22.80; HRMS (ESI+) *m*/*z* calcd. for C_26_H_24_N_8_O [M + H]^+^: 465.2151, found: 465.2157.

##### 4-(6-(([2,3′-Bipyridin]-5-ylmethyl)amino)-9-isopropyl-9*H*-purin-2-yl)benzamide (**18c**)

Yield 41%; ^1^H NMR (400 MHz, DMSO-*d*_6_) δ 9.22 (d, *J* = 1.6 Hz, 1H), 8.84 (s, 1H), 8.60 (d, *J* = 4.6 Hz, 2H), 8.45 (d, *J* = 8.3 Hz, 2H), 8.39 (d, *J* = 8.1 Hz, 1H), 8.34 (s, 1H), 8.03 (d, *J* = 5.5 Hz, 1H), 7.99 (dd, *J* = 10.8, 6.9 Hz, 4H), 7.49 (dd, *J* = 7.9, 4.8 Hz, 1H), 7.43 (s, 1H), 4.87 (dd, *J* = 13.6, 6.8 Hz, 3H), 1.60 (d, *J* = 6.7 Hz, 6H). ^13^C NMR (101 MHz, DMSO-*d*_6_) δ 168.10, 156.73, 152.81, 150.17, 149.81, 148.13, 140.28, 137.04, 135.50, 134.39, 134.24, 128.00, 127.79, 124.21, 120.80, 47.06, 22.75; HRMS (ESI+) *m*/*z* calcd. for C_26_H_24_N_8_O [M + H]^+^: 465.2151, found: 465.2161.

##### *N*-([2,3′-Bipyridin]-5-ylmethyl)-2-(1*H*-indol-5-yl)-9-isopropyl-9*H*-purin-6-amine (**18d**)

Yield 48%; ^1^H NMR (400 MHz, CDCl_3_) δ 9.19 (s, 1H), 8.85 (s, 2H), 8.65 (s, 1H), 8.50–8.25 (m, 3H), 7.93 (d, *J* = 7.3 Hz, 1H), 7.82 (s, 1H), 7.70 (d, *J* = 7.6 Hz, 1H), 7.54–7.34 (m, 2H), 7.27 (d, *J* = 15.0 Hz, 2H), 6.68 (s, 1H), 6.40 (s, 1H), 5.13 (s, 2H), 5.00 (m, 1H), 1.69 (d, *J* = 6.0 Hz, 6H). ^13^C NMR (101 MHz, CDCl_3_) δ 159.96, 154.03, 153.73, 149.76, 149.64, 148.18, 137.57, 137.15, 136.60, 134.70, 134.52, 134.28, 128.02, 124.77, 123.57, 122.67, 121.27, 120.41, 110.63, 103.69, 46.92, 22.81; HRMS (ESI+) *m*/*z* calcd. for C_27_H_24_N_8_ [M + H]^+^: 461.2202, found: 461.2203.

##### *N*-([2,3′-Bipyridin]-5-ylmethyl)-2-(1*H*-indazol-6-yl)-9-isopropyl-9*H*-purin-6-amine (**18e**)

Yield 40%; ^1^H NMR (400 MHz, CDCl_3_) δ 9.20 (d, *J* = 1.7 Hz, 1H), 8.91 (d, *J* = 1.8 Hz, 1H), 8.75–8.62 (m, 2H), 8.34 (ddd, *J* = 11.4, 8.0, 5.3 Hz, 2H), 8.13 (s, 1H), 7.99–7.90 (m, 2H), 7.86 (t, *J* = 9.7 Hz, 1H), 7.74 (d, *J* = 8.3 Hz, 1H), 7.42 (dd, *J* = 7.9, 4.8 Hz, 1H), 6.53 (s, 1H), 5.14 (s, 2H), 5.01 (dt, *J* = 13.5, 6.8 Hz, 1H), 1.72 (d, *J* = 6.8 Hz, 6H). ^13^C NMR (101 MHz, CDCl_3_+ CD_3_OD) δ 163.00, 157.18, 153.39, 153.00, 151.43, 142.03, 141.43, 141.03, 139.29, 139.04, 138.93, 127.99, 127.73, 125.17, 125.07, 124.08, 122.47, 51.22, 26.39; HRMS (ESI+) *m*/*z* calcd. for C_26_H_23_N_9_ [M + H]^+^: 462.2155, found: 462.2149.

##### *N*-([2,3′-Bipyridin]-5-ylmethyl)-2-(1*H*-indazol-5-yl)-9-isopropyl-9*H*-purin-6-amine (**18f**)

12%; ^1^H NMR (400 MHz, DMSO-*d_6_*) δ 13.14 (s, 1H), 9.22 (s, 1H), 8.84 (d, *J* = 9.8 Hz, 2H), 8.60 (d, *J* = 4.9 Hz, 1H), 8.48 (d, *J* = 8.9 Hz, 2H), 8.38 (d, *J* = 7.8 Hz, 1H), 8.26 (d, *J* = 7.4 Hz, 1H), 8.21 (s, 1H), 8.02 (s, 2H), 7.59 (d, *J* = 8.9 Hz, 1H), 7.52–7.45 (m, 1H), 4.88 (dd, *J* = 13.6, 6.8 Hz, 3H), 1.61 (d, *J* = 6.7 Hz, 7H). ^13^C NMR (101 MHz, CDCl_3_ + CD_3_OD) δ 163.26, 157.87, 157.14, 153.30, 152.92, 151.39, 141.75, 140.94, 139.16, 139.03, 138.98, 135.88, 131.21, 127.98, 127.10, 125.12, 124.95, 122.12, 51.14, 26.41; HRMS (ESI+) *m*/*z* calcd. for C_26_H_23_N_9_ [M + H]^+^: 462.2155, found: 462.2154.

##### *N*-([2,3′-Bipyridin]-5-ylmethyl)-9-isopropyl-2-(quinolin-3-yl)-9*H*-purin-6-amine (**18g**)

Yield 38%; ^1^H NMR (400 MHz, CDCl_3_) δ 10.06 (d, *J* = 1.9 Hz, 1H), 9.35 (s, 1H), 9.22 (d, *J* = 2.0 Hz, 1H), 8.90 (s, 1H), 8.67 (d, *J* = 3.3 Hz, 1H), 8.37 (d, *J* = 8.1 Hz, 2H), 8.07 (d, *J* = 8.5 Hz, 1H), 7.98 (dd, *J* = 8.1, 2.1 Hz, 1H), 7.94 (s, 1H), 7.90–7.83 (m, 1H), 7.80 (d, *J* = 8.2 Hz, 1H), 7.71 (d, *J* = 7.5 Hz, 1H), 7.44 (dd, *J* = 7.9, 4.8 Hz, 1H), 6.48 (s, 1H), 5.15 (s, 2H), 5.02 (dd, *J* = 13.6, 7.0 Hz, 1H), 1.74 (d, *J* = 6.8 Hz, 6H). ^13^C NMR (101 MHz, CDCl_3_) δ 156.78, 153.96, 150.91, 149.89, 149.60, 148.62, 148.20, 138.42, 136.50, 135.24, 134.56, 134.26, 134.05, 131.39, 129.97, 129.28, 128.77, 127.79, 126.73, 123.58, 120.39, 47.33, 22.78; HRMS (ESI+) *m*/*z* calcd. for C_28_H_24_N_8_ [M + H]^+^: 473.2202, found: 473.2204.

##### *N*-([2,3′-Bipyridin]-5-ylmethyl)-9-isopropyl-2-(6-methylpyridin-3-yl)-9*H*-purin-6-amine (**18h**)

Yield 78%; ^1^H NMR (400 MHz, CDCl_3_) δ 9.53 (s, 1H), 9.15 (s, 1H), 8.78 (d, *J* = 1.6 Hz, 1H), 8.62 (s, 1H), 8.59 (dd, *J* = 8.1, 2.0 Hz, 1H), 8.27 (d, *J* = 8.0 Hz, 1H), 7.86 (dd, *J* = 8.1, 2.1 Hz, 1H), 7.81 (s, 1H), 7.68 (d, *J* = 8.1 Hz, 1H), 7.37 (dd, *J* = 7.6, 4.7 Hz, 1H), 7.23 (d, *J* = 8.1 Hz, 1H), 6.54 (s, 1H), 5.02 (s, 2H), 4.90 (dt, *J* = 13.6, 6.8 Hz, 1H), 2.62 (s, 3H), 1.64 (d, *J* = 6.8 Hz, 6H); ^13^C NMR (101 MHz, CDCl_3_) δ 159.38, 156.99, 154.30, 153.94, 149.98, 149.63, 149.38, 148.30, 138.33, 136.56, 135.88, 134.65, 134.32, 134.22, 131.64, 123.66, 122.84, 120.40, 119.49, 77.48, 77.16, 76.84, 47.33, 41.94, 24.48, 22.80; HRMS (ESI+) *m*/*z* calcd. for C_25_H_24_N_8_ [M + H]^+^ 437.2202, found 437.2199.

##### *N*-([2,3′-Bipyridin]-5-ylmethyl)-2-(6-aminopyridin-3-yl)-9-isopropyl-9*H*-purin-6-amine] (**18i**)

Yield 39%; ^1^H NMR (400 MHz, CDCl_3_) δ 9.13 (dd, *J* = 6.1, 1.8 Hz, 2H), 8.76 (d, *J* = 1.7 Hz, 1H), 8.59 (dd, *J* = 4.8, 1.6 Hz, 1H), 8.44 (dd, *J* = 8.6, 2.2 Hz, 1H), 8.28–8.21 (m, 1H), 7.83 (dd, *J* = 8.1, 2.2 Hz, 1H), 7.75 (s, *J* = 13.0 Hz, 1H), 7.63 (d, *J* = 8.1 Hz, 1H), 7.34 (dd, *J* = 8.0, 4.8 Hz, 1H), 6.78 (s, 1H), 6.53 (d, *J* = 8.6 Hz, 1H), 4.97 (d, *J* = 4.0 Hz, 2H), 4.86 (dt, *J* = 13.4, 6.7 Hz, 3H), 1.60 (t, *J* = 6.0 Hz, 6H); ^13^C NMR (101 MHz, CDCl_3_) δ 159.03, 157.38, 153.95, 149.97, 149.69, 148.55, 148.33, 138.02, 137.85, 136.61, 134.75, 134.38, 125.43, 123.70, 120.49, 119.04, 108.08, 77.48, 77.16, 76.84, 47.19, 22.85; HRMS (ESI+) *m*/*z* calcd. for C_24_H_23_N_9_ [M + H]^+^ 438.2155, found 438.2151.

##### *N*-([2,3′-Bipyridin]-5-ylmethyl)-2-(2-aminopyridin-4-yl)-9-isopropyl-9*H*-purin-6-amine] (**18j**)

Yield 38%; ^1^H NMR (400 MHz, CDCl_3_) δ 9.15 (d, *J* = 1.7 Hz, 1H), 8.78 (d, *J* = 1.7 Hz, 1H), 8.62 (dd, *J* = 4.8, 1.5 Hz, 1H), 8.30–8.23 (m, 1H), 8.13 (d, *J* = 5.4 Hz, 1H), 7.84 (s, 1H), 7.82 (d, *J* = 2.2 Hz, 1H), 7.68 (s, 1H), 7.67–7.65 (m, 1H), 7.55 (s, 1H), 7.36 (dd, *J* = 7.7, 5.1 Hz, 1H), 6.56 (s, 1H), 5.01 (s, 2H), 4.91 (dt, *J* = 13.6, 6.8 Hz, 1H), 4.77 (s, 2H), 1.64 (d, *J* = 6.8 Hz, 6H); ^13^C NMR (101 MHz, CDCl_3_) δ 158.93, 156.67, 154.28, 154.04, 150.04, 149.75, 148.41, 148.30, 147.65, 138.80, 136.55, 134.62, 134.34, 134.16, 123.71, 120.50, 113.11, 107.70, 77.48, 77.16, 76.84, 47.39, 22.88; HRMS (ESI+) *m*/*z* calcd. for C_24_H_23_N_9_ [M + H]^+^ 438.2155, found 438.2163.

##### *N*-([2,3′-Bipyridin]-5-ylmethyl)-9-isopropyl-2-(pyrimidin-5-yl)-9*H*-purin-6-amine (**18k**)

Yield: 15%; ^1^H NMR (400 MHz, CDCl_3_) δ 9.71 (s, 2H), 9.28 (s, 1H), 9.21 (d, *J* = 1.6 Hz, 1H), 8.83 (d, *J* = 1.7 Hz, 1H), 8.67 (dd, *J* = 4.9, 1.6 Hz, 1H), 8.43–8.36 (m, 1H), 7.96–7.86 (m, 2H), 7.76 (d, *J* = 8.1 Hz, 1H), 7.51–7.43 (m, 1H), 6.52 (s, 1H), 5.07 (s, 2H), 4.94 (dq, *J* = 13.6, 6.8 Hz, 1H), 1.71 (t, *J* = 5.6 Hz, 6H). ^13^C NMR (101 MHz, DMSO-*d*_6_) δ 159.24, 156.22, 150.10, 149.80, 148.07, 140.60, 137.01, 135.69, 134.38, 134.30, 124.22, 120.80, 47.27, 22.68. HRMS (ESI) calcd. for C_23_H_21_N_9_ [M + H]^+^: 424.1998, found: 424.2002.

##### *N*-([2,3′-Bipyridin]-5-ylmethyl)-2-(2-aminopyrimidin-5-yl)-9-isopropyl-9*H*-purin-6-amine] (**18l**)

Yield 56%; ^1^H NMR (400 MHz, DMSO*-d_6_*) δ 9.28 (s, 1H), 9.10 (s, 2H), 8.79 (s, 1H), 8.66 (s, 1H), 8.50 (s, 1H), 8.39 (d, *J* = 7.5 Hz, 1H), 8.24 (s, 1H), 7.99 (d, *J* = 8.1 Hz, 1H), 7.95 (d, *J* = 7.7 Hz, 1H), 7.51 (s, 1H), 7.00 (s, 2H), 5.00–4.70 (m, 3H), 1.56 (d, *J* = 6.7 Hz, 7H); ^13^C NMR (101 MHz, DMSO-*d_6_*) δ 163.83, 157.59, 154.87, 152.26, 149.24, 147.49, 138.92, 136.44, 135.39, 133.74, 124.01, 120.69, 120.25, 46.38, 40.14, 39.94, 39.73, 39.52, 39.52, 39.31, 39.10, 38.90, 22.19; HRMS (ESI+) *m*/*z* calcd. for C_23_H_22_N_10_ [M + H]^+^ 439.2107, found 439.2113.

##### *N*-([2,3′-Bipyridin]-5-ylmethyl)-9-isopropyl-2-(1*H*-pyrazol-5-yl)-9*H*-purin-6-amine (**18m**)

Yield 30%; ^1^H NMR (400 MHz, CDCl_3_) δ 9.09 (d, *J* = 1.6 Hz, 1H), 8.71 (d, *J* = 1.7 Hz, 1H), 8.57 (d, *J* = 3.5 Hz, 1H), 8.28–8.15 (m, 1H), 7.82–7.74 (m, 2H), 7.66–7.51 (m, 2H), 7.32 (dd, *J* = 7.9, 4.8 Hz, 1H), 6.92 (d, *J* = 1.4 Hz, 1H), 6.59 (s, 1H), 4.92 (s, 1H), 4.82 (dd, *J* = 13.6, 6.8 Hz, 1H), 1.56 (d, *J* = 6.8 Hz, 5H). ^13^C NMR (101 MHz, CDCl_3_) δ 153.98, 149.92, 149.59, 148.19, 138.14, 136.47, 134.52, 134.27, 133.81, 123.60, 120.37, 105.52, 47.08, 22.79; HRMS (ESI+) *m*/*z* calcd. for C_22_H_21_N_9_ [M + H]^+^: 412.1998, found: 412.2005.

##### *N*-([2,3′-Bipyridin]-5-ylmethyl)-9-isopropyl-2-(1*H*-pyrazol-4-yl)-9*H*-purin-6-amine (**18n**)

Yield 45%; ^1^H NMR (400 MHz, CDCl_3_) δ 9.02 (d, *J* = 6.3 Hz, 1H), 8.67 (d, *J* = 6.6 Hz, 1H), 8.48 (d, *J* = 4.8 Hz, 1H), 8.20–7.97 (m, 3H), 7.79 (d, *J* = 8.0 Hz, 1H), 7.68 (s, 1H), 7.57 (t, *J* = 7.9 Hz, 1H), 7.28–7.22 (m, 2H), 6.77 (s, 1H), 4.85 (s, 2H), 4.74 (dd, *J* = 13.9, 6.8 Hz, 1H), 1.48 (t, *J* = 7.6 Hz, 6H). ^13^C NMR (101 MHz, CDCl_3_ + CD_3_OD) δ 155.57, 153.33, 149.54, 149.27, 147.69, 137.13, 136.78, 134.87, 134.78, 123.86, 123.48, 120.71, 117.87, 46.85, 22.68; HRMS (ESI+) *m*/*z* calcd. for C_22_H_21_N_9_ [M + H]^+^: 412.1998, found: 412.2001.

##### N-([2,3′-Bipyridin]-5-ylmethyl)-2-(2-aminophenyl)-9-isopropyl-9H-purin-6-amine (**19a**)

Yield 52%; ^1^H NMR (400 MHz, CDCl_3_) δ 9.20 (d, *J* = 1.7 Hz, 1H), 8.83 (s, 1H), 8.70–8.64 (m, 1H), 8.47 (dd, *J* = 8.0, 1.4 Hz, 1H), 8.34 (d, *J* = 8.0 Hz, 1H), 7.92 (d, *J* = 8.1 Hz, 1H), 7.86 (s, 1H), 7.74 (d, *J* = 8.1 Hz, 1H), 7.43 (dd, *J* = 7.9, 4.8 Hz, 1H), 7.24–7.17 (m, 1H), 6.80 (t, *J* = 7.2 Hz, 1H), 6.75 (d, *J* = 8.0 Hz, 1H), 6.45 (s, 1H), 5.05 (s, 2H), 4.89 (dt, *J* = 13.5, 6.8 Hz, 1H), 1.68 (d, *J* = 6.8 Hz, 6H). ^13^C NMR (101 MHz, CDCl_3_) δ 160.21, 153.95, 153.62, 149.91, 149.44, 148.22, 147.90, 137.85, 136.34, 134.55, 134.24, 133.93, 131.22, 130.75, 123.58, 120.44, 120.39, 117.13, 117.03, 47.17, 22.69; HRMS (ESI+) *m*/*z* calcd. for C_25_H_24_N_8_ [M + H]^+^: 437.2202, found: 437.2204.

##### *N*-([2,3′-Bipyridin]-5-ylmethyl)-2-(3-aminophenyl)-9-isopropyl-9*H*-purin-6-amine (**19b**)

Yield: 50%; ^1^H NMR (600 MHz, CDCl_3_) δ 9.16 (s, 1H), 8.82 (s, 1H), 8.63 (d, *J* = 4.2 Hz, 1H), 8.29 (d, *J* = 7.8 Hz, 1H), 7.96–7.78 (m, 3H), 7.70 (d, *J* = 8.2 Hz, 1H), 7.45–7.35 (m, 1H), 7.24 (d, *J* = 12.2 Hz, 2H), 6.77 (d, *J* = 6.9 Hz, 1H), 6.36 (s, 1H), 5.17–4.88 (m, 3H), 1.65 (d, *J* = 6.7 Hz, 6H). ^13^C NMR (101 MHz, DMSO*-d_6_*) δ 158.26, 152.71, 149.91, 148.90, 147.91, 139.71, 137.15, 136.08, 134.44, 129.07, 124.30, 122.58, 120.81, 120.59, 116.37, 115.88, 113.92, 46.94, 22.73; HRMS (ESI+) *m*/*z* calcd. for C_25_H_24_N_8_ [M + H]^+^: 437.2202, found: 437.2205.

##### *N*-([2,3′-Bipyridin]-5-ylmethyl)-2-(4-aminophenyl)-9-isopropyl-9*H*-purin-6-amine (**19c**)

Yield: 55%; ^1^H NMR (400 MHz, CDCl_3_) δ 9.19 (d, *J* = 2.0 Hz, 1H), 8.84 (d, *J* = 1.7 Hz, 1H), 8.66 (dd, *J* = 4.8, 1.5 Hz, 1H), 8.37–8.28 (m, 3H), 7.92 (dd, *J* = 8.1, 2.1 Hz, 1H), 7.80 (s, 1H), 7.71 (d, *J* = 8.2 Hz, 1H), 7.41 (dd, *J* = 8.0, 4.8 Hz, 1H), 6.76 (d, *J* = 8.6 Hz, 2H), 6.37 (s, 1H), 5.08 (s, 2H), 4.95 (dq, *J* = 13.5, 6.6 Hz, 1H), 3.87 (s, 2H), 1.67 (d, *J* = 6.8 Hz, 6H). ^13^C NMR (101 MHz, CDCl_3_) δ 153.81, 149.82, 149.65, 148.21, 137.53, 136.56, 134.69, 134.40, 134.27, 129.64, 123.57, 120.41, 114.55, 46.91, 22.76.; HRMS (ESI+) *m*/*z* calcd. for C_25_H_24_N_8_ [M + H]^+^: 437.2202, found: 437.2199.

##### *N*-([2,3′-Bipyridin]-5-ylmethyl)-2-(6-fluoropyridin-3-yl)-9-isopropyl-9*H*-purin-6-amine (**21a**)

Yield 72%; ^1^H NMR (400 MHz, CDCl_3_) δ 9.26 (d, *J* = 2.3 Hz, 1H), 9.15 (s, 1H), 8.78 (dd, *J* = 8.3, 2.4 Hz, 1H), 8.75 (d, *J* = 2.3 Hz, 1H), 8.62 (s, 1H), 8.29–8.23 (m, 1H), 7.82 (dd, *J* = 8.2, 2.3 Hz, 1H), 7.79 (s, 1H), 7.66 (d, *J* = 8.0 Hz, 1H), 7.35 (dd, *J* = 7.8, 4.8 Hz, 1H), 6.96 (dd, *J* = 8.6, 2.7 Hz, 1H), 6.71 (s, 1H), 5.00 (s, 2H), 4.88 (dt, *J* = 13.5, 6.8 Hz, 1H), 1.63 (d, *J* = 6.8 Hz, 6H); ^13^C NMR (101 MHz, CDCl_3_) δ 165.72, 163.33, 155.86, 154.36, 154.02, 150.02, 149.59, 148.38, 148.27, 148.23, 140.96, 140.88, 138.50, 136.47, 134.29, 134.05, 132.63, 132.59, 123.67, 120.40, 119.55, 109.06, 108.69, 77.48, 77.16, 76.84, 47.41, 41.92, 22.80; HRMS (ESI+) *m*/*z* calcd. for C_24_H_21_FN_8_ [M + H]^+^ 441.1951, found 441.1944.

##### *N*-([2,3′-Bipyridin]-5-ylmethyl)-2-(2-fluoropyridin-4-yl)-9-isopropyl-9*H*-purin-6-amine] (**21b**)

Yield 57%; ^1^H NMR (400 MHz, CDCl_3_) δ 9.15 (d, *J* = 1.8 Hz, 1H), 8.78 (d, *J* = 1.7 Hz, 1H), 8.63 (dd, *J* = 4.8, 1.4 Hz, 1H), 8.28 (d, *J* = 4.6 Hz, 1H), 8.28–8.25 (m, 1H), 8.18 (d, *J* = 5.2 Hz, 1H), 7.92 (s, 1H), 7.86 (s, 1H), 7.86–7.81 (m, 1H), 7.70 (d, *J* = 8.1 Hz, 1H), 7.37 (dd, *J* = 8.0, 4.8 Hz, 1H), 6.62 (s, 1H), 5.03 (s, 2H), 4.91 (dt, *J* = 13.6, 6.8 Hz, 1H), 1.66 (d, *J* = 6.8 Hz, 6H); ^13^C NMR (101 MHz, CDCl_3_) δ 165.95, 163.60, 155.33, 154.37, 154.18, 152.12, 150.10, 149.66, 148.32, 147.88, 147.73, 139.23, 136.53, 134.57, 134.34, 133.89, 123.70, 120.48, 120.12, 120.08, 108.31, 107.93, 77.48, 77.16, 76.84, 47.60, 22.85.; HRMS (ESI+) *m*/*z* calcd. for C_24_H_21_FN_8_ [M + H]^+^ 441.1951, found 441.1954.

##### *N*-([2,3′-Bipyridin]-5-ylmethyl)-9-ethyl-2-(pyridin-3-yl)-9*H*-purin-6-amine (**28a**)

Yield 48%; ^1^H NMR (400 MHz, CDCl_3_) δ 9.71 (d, *J* = 1.5 Hz, 1H), 9.20 (d, *J* = 1.8 Hz, 1H), 8.88–8.76 (m, 2H), 8.67 (ddd, *J* = 7.8, 4.8, 1.4 Hz, 2H), 8.33 (dt, *J* = 8.0, 1.8 Hz, 1H), 7.91 (dd, *J* = 8.1, 2.1 Hz, 1H), 7.85 (s, 1H), 7.74 (d, *J* = 8.1 Hz, 1H), 7.44 (ddd, *J* = 17.0, 7.9, 4.9 Hz, 2H), 6.49 (t, *J* = 5.6 Hz, 1H), 5.08 (s, 2H), 4.35 (q, *J* = 7.3 Hz, 2H), 1.62 (t, *J* = 7.3 Hz, 3H). ^13^C NMR (101 MHz, CDCl_3_) δ 156.89, 154.25, 153.81, 150.24, 149.83, 149.50, 148.12, 140.03, 136.42, 135.45, 134.50, 134.24, 134.18, 134.04, 123.58, 123.17, 120.30, 38.89, 15.52; HRMS (ESI+) *m*/*z* calcd. for C_23_H_20_N_8_ [M + H]^+^: 409.1889, found: 409.1882.

##### *N*-([2,3′-Bipyridin]-5-ylmethyl)-9-cyclopentyl-2-(pyridin-3-yl)-9*H*-purin-6-amine (**28b**)

Yield 23%; ^1^H NMR (400 MHz, CDCl_3_) δ 9.72 (s, 1H), 9.21 (s, 1H), 8.92–8.79 (m, 2H), 8.68 (s, 2H), 8.36 (d, *J* = 7.9 Hz, 1H), 7.98–7.85 (m, 2H), 7.76 (d, *J* = 8.2 Hz, 1H), 7.59–7.40 (m, 2H), 6.45 (s, 1H), 5.17–4.90 (m, 3H), 2.38 (d, *J* = 7.8 Hz, 2H), 2.08 (ddd, *J* = 27.0, 13.6, 6.9 Hz, 4H), 1.89 (d, *J* = 6.7 Hz, 2H). ^13^C NMR (101 MHz, CDCl_3_) δ 154.24, 153.90, 150.10, 149.86, 149.77, 149.55, 148.16, 139.20, 136.46, 135.53, 134.25, 134.03, 123.60, 123.21, 120.34, 56.30, 32.77, 24.17; HRMS (ESI+) *m*/*z* calcd. for C_26_H_24_N_8_ [M + H]^+^: 449.2202, found: 449.2213.

##### *N*-([2,3′-Bipyridin]-5-ylmethyl)-2-(pyridin-3-yl)-9-(tetrahydro-2*H*-pyran-4-yl)-9*H*-purin-6-amine (**28c**)

Yield 24%; ^1^H NMR (400 MHz, CDCl_3_) δ 9.71 (d, *J* = 1.6 Hz, 1H), 9.21 (d, *J* = 1.7 Hz, 1H), 8.84 (d, *J* = 7.6 Hz, 2H), 8.74–8.63 (m, 2H), 8.41–8.32 (m, 1H), 7.97–7.88 (m, 2H), 7.77 (d, *J* = 8.1 Hz, 1H), 7.51 (dd, *J* = 7.8, 4.9 Hz, 1H), 7.45 (dd, *J* = 8.0, 4.4 Hz, 1H), 6.43 (s, 1H), 5.09 (s, 2H), 4.81 (ddd, *J* = 16.1, 11.9, 4.3 Hz, 1H), 4.23 (dd, *J* = 11.4, 4.0 Hz, 2H), 3.71 (dd, *J* = 11.9, 10.0 Hz, 2H), 2.31 (dd, *J* = 12.2, 4.2 Hz, 2H), 2.24–2.18 (m, 2H). ^13^C NMR (101 MHz, CDCl_3_) δ 156.80, 153.82, 150.20, 149.74, 149.52, 148.03, 138.27, 136.46, 135.60, 134.55, 134.34, 134.16, 133.98, 123.64, 123.51, 123.26, 120.33, 67.07, 51.77, 33.03; HRMS (ESI+) *m*/*z* calcd. for C_26_H_24_N_8_O [M + H]^+^: 465.2151, found: 465.2149.

##### 2-(6-Aminopyridin-3-yl)-9-ethyl-*N*-((2′-methyl-[2,4′-bipyridin]-5-yl)methyl)-9*H*-purin-6-amine (**30a**)

Yield 30%; ^1^H NMR (400 MHz, CDCl_3_) δ 9.20 (d, *J* = 1.9 Hz, 1H), 8.83 (d, *J* = 1.8 Hz, 1H), 8.61 (d, *J* = 5.2 Hz, 1H), 8.50 (dd, *J* = 8.6, 2.2 Hz, 1H), 7.91 (dd, *J* = 8.1, 2.2 Hz, 1H), 7.76 (dd, *J* = 12.1, 5.7 Hz, 3H), 7.68–7.62 (m, 1H), 6.59 (t, *J* = 7.7 Hz, 1H), 6.26 (s, 1H), 5.06 (d, *J* = 4.6 Hz, 2H), 4.70 (s, 2H), 4.32 (q, *J* = 7.3 Hz, 2H), 2.66 (s, 3H), 1.60 (t, *J* = 7.3 Hz, 3H). ^13^C NMR (101 MHz, CDCl_3_+ CD_3_OD) δ 159.17, 158.89, 157.77, 153.57, 149.41, 149.26, 148.46, 146.80, 139.19, 137.87, 136.56, 135.51, 124.91, 120.92, 120.87, 118.38, 118.05, 108.03, 38.79, 24.13, 15.44; HRMS (ESI+) *m*/*z* calcd. for C_24_H_23_N_9_ [M + H]^+^: 438.2155, found: 438.2152.

##### 2-(2-Aminopyrimidin-5-yl)-9-ethyl-*N*-((2′-methyl-[2,4′-bipyridin]-5-yl)methyl)-9*H*-purin-6-amine (**30b**)

Yield 34%; ^1^H NMR (400 MHz, DMSO-*d*_6_) δ 9.09 (s, 2H), 8.81 (s, 1H), 8.52 (t, *J* = 7.2 Hz, 2H), 8.18 (s, 1H), 8.05 (d, *J* = 8.1 Hz, 1H), 7.97 (dd, *J* = 8.2, 1.9 Hz, 1H), 7.90 (s, 1H), 7.02 (s, 2H), 4.84 (s, 2H), 4.23 (q, *J* = 7.2 Hz, 2H), 2.54 (s, 3H), 1.45 (t, *J* = 7.2 Hz, 3H). ^13^C NMR (101 MHz, CDCl_3_+ CD_3_OD) δ 166.78, 162.68, 162.39, 159.71, 157.94, 157.41, 153.20, 152.93, 150.90, 143.47, 140.57, 139.46, 126.30, 125.02, 124.99, 122.47, 122.13, 42.82, 27.76, 19.29. HRMS (ESI) calcd. for C_23_H_22_N_10_ [M + H]^+^: 439.2102, found: 439.2122; HRMS (ESI+) *m*/*z* calcd. for C_24_H_22_N_10_ [M + H]^+^: 439.2102, found: 439.2122. 

##### 9-Ethyl-*N*-((2′-methyl-[2,4′-bipyridin]-5-yl)methyl)-2-(pyrimidin-5-yl)-9*H*-purin-6-amine (**30c**)

Yield 20%; ^1^H NMR (400 MHz, CDCl_3_) δ 9.71 (s, 2H), 9.28 (s, 1H), 8.83 (d, *J* = 1.9 Hz, 1H), 8.61 (d, *J* = 5.3 Hz, 1H), 7.91 (dd, *J* = 8.1, 2.2 Hz, 1H), 7.86 (s, 1H), 7.78 (d, *J* = 7.7 Hz, 2H), 7.66 (d, *J* = 5.2 Hz, 1H), 6.31 (s, 1H), 5.08 (s, 2H), 4.36 (q, *J* = 7.3 Hz, 2H), 2.66 (s, 3H), 1.63 (t, *J* = 7.3 Hz, 3H). ^13^C NMR (101 MHz, CDCl_3_) δ 159.16, 158.94, 156.47, 154.68, 154.35, 154.07, 149.71, 149.38, 146.25, 140.37, 136.33, 134.67, 131.73, 120.67, 120.51, 118.10, 39.01, 24.55, 15.51; HRMS (ESI+) *m*/*z* calcd. for C_23_H_21_N_9_ [M + H]^+^: 424.1998, found: 424.2007.

##### *N*-([2,2′-Bipyridin]-5-ylmethyl)-2-(6-aminopyridin-3-yl)-9-ethyl-9*H*-purin-6-amine (**30d**)

Yield 50%; ^1^H NMR (400 MHz, CDCl_3_) δ 9.22 (d, *J* = 1.8 Hz, 1H), 8.79 (d, *J* = 1.7 Hz, 1H), 8.69 (d, *J* = 3.9 Hz, 1H), 8.51 (dd, *J* = 8.6, 2.2 Hz, 1H), 8.39 (dd, *J* = 7.8, 6.3 Hz, 2H), 7.92 (dd, *J* = 8.2, 2.2 Hz, 1H), 7.83 (td, *J* = 7.8, 1.8 Hz, 1H), 7.77 (s, 1H), 7.35–7.30 (m, 1H), 6.58 (d, *J* = 8.6 Hz, 1H), 6.11 (s, 1H), 5.08 (s, 2H), 4.64 (s, 2H), 4.32 (q, *J* = 7.3 Hz, 2H), 1.61 (t, *J* = 7.3 Hz, 3H). ^13^C NMR (101 MHz, DMSO*-d*_6_) δ 161.02, 157.39, 155.70, 154.34, 149.60, 149.11, 148.96, 140.65, 137.57, 136.92, 136.74, 124.34, 123.08, 120.76, 120.55, 107.52, 38.42, 15.79; HRMS (ESI+) *m*/*z* calcd. for C_23_H_21_N_9_ [M + H]^+^: 424.1998, found: 424.2008.

##### *N*-([2,2′-Bipyridin]-5-ylmethyl)-2-(2-aminopyrimidin-5-yl)-9-ethyl-9*H*-purin-6-amine (**30e**)

Yield 55%;^1^H NMR (400 MHz, DMSO*-d*_6_) δ 9.09 (s, 2H), 8.77 (s, 1H), 8.69–8.63 (m, 1H), 8.50 (s, 1H), 8.35 (t, *J* = 7.8 Hz, 2H), 8.18 (s, 1H), 8.00–7.88 (m, 2H), 7.43 (ddd, *J* = 7.5, 4.8, 1.1 Hz, 1H), 7.01 (s, 2H), 4.85 (s, 2H), 4.23 (q, *J* = 7.2 Hz, 2H), 1.46 (t, *J* = 7.3 Hz, 3H). ^13^C NMR (101 MHz, DMSO*-d_6_*) δ 164.33, 158.16, 155.65, 154.34, 149.67, 149.13, 141.02, 137.69, 136.85, 136.76, 124.45, 120.78, 120.57, 38.50, 15.76; HRMS (ESI+) *m*/*z* calcd. for C_22_H_20_N_10_ [M + H]^+^: 425.1951, found: 425.1672.

#### 3.1.6. General Procedure for Synthesis of **20a–d**

The anilino compound **19a–c** (70 mg, 1.0 eq.) and trimethylamine (2.0 eq.) were added to a solution of respective bromoalkylalcohol (1.5 eq) in *n*-butanol (1.0 mL) at rt. The reaction mixture was heated with stirring at 110 °C for 12 h. After the reaction was completed, the mixture was cooled to rt and the solvent was evaporated. The residue was diluted with water and extracted with EtOAc (3 × 50 mL). The combined organic extracts were dried over MgSO_4_, filtered, and concentrated under a reduced pressure. The desired product was obtained by a column chromatography using 5% methanol in dichloromethane as eluent.

##### 2-((2-(6-(([2,3′-Bipyridin]-5-ylmethyl)amino)-9-isopropyl-9*H*-purin-2-yl)phenyl)amino)ethan-1-ol (**20a**)

Yield: 30%; ^1^H NMR (600 MHz, CDCl_3_) δ 9.09 (d, *J* = 1.5 Hz, 1H), 8.67 (s, 1H), 8.58 (d, *J* = 3.8 Hz, 1H), 8.44 (t, *J* = 12.6 Hz, 1H), 8.21 (d, *J* = 8.0 Hz, 1H), 7.74 (d, *J* = 8.6 Hz, 1H), 7.71 (s, 1H), 7.56 (d, *J* = 8.1 Hz, 1H), 7.32 (dd, *J* = 7.9, 4.8 Hz, 1H), 7.28–7.23 (m, 2H), 6.76–6.70 (m, 2H), 6.64 (d, *J* = 34.1 Hz, 1H), 4.91 (s, 2H), 4.86–4.81 (m, 1H), 3.89 (t, *J* = 5.0 Hz, 2H), 3.41 (t, *J* = 5.0 Hz, 2H), 1.59 (t, *J* = 11.7 Hz, 6H). ^13^C NMR (101 MHz, CDCl_3_) δ 160.14, 153.76, 153.48, 149.76, 149.44, 148.72, 148.12, 137.58, 136.35, 134.54, 134.26, 133.94, 131.54, 131.17, 123.57, 120.31, 115.76, 111.48, 61.31, 47.07, 45.91, 22.68; HRMS (ESI+) *m*/*z* calcd. for C_27_H_28_N_8_O [M + H]^+^: 481.2464, found: 481.2467.

##### 3-((2-(6-(([2,3′-Bipyridin]-5-ylmethyl)amino)-9-isopropyl-9H-purin-2-yl)phenyl)amino)propan-1-ol (**20b**)

Yield: 34%; ^1^H NMR (600 MHz, CDCl_3_) δ 9.14 (s, 1H), 8.81 (d, *J* = 52.8 Hz, 2H), 8.61 (s, 1H), 8.50 (d, *J* = 7.0 Hz, 1H), 8.26 (d, *J* = 7.5 Hz, 1H), 7.91–7.75 (m, 2H), 7.66 (d, *J* = 7.6 Hz, 1H), 7.36 (s, 1H), 7.26 (d, *J* = 11.8 Hz, 1H), 6.74 (dd, *J* = 20.9, 7.4 Hz, 2H), 6.51 (s, 1H), 5.01 (s, 2H), 4.84 (m, 1H), 3.79 (t, 2H), 3.37 (t, 2H), 1.93 (m, 2H), 1.63 (d, *J* = 6.3 Hz, 6H). ^13^C NMR (101 MHz, CDCl_3_) δ 153.51, 149.79, 149.43, 148.11, 137.65, 136.39, 134.24, 134.00, 131.44, 131.25, 120.35, 77.36, 77.04, 76.73, 61.17, 47.11, 46.03, 22.68, 8.60.; HRMS (ESI+) *m*/*z* calcd. for C_28_H_30_N_8_O [M + H]^+^: 495.2621, found: 495.2626.

##### 3-((3-(6-(([2,3′-Bipyridin]-5-ylmethyl)amino)-9-isopropyl-9H-purin-2-yl)phenyl)amino)propan-1-ol (**20c**)

Yield 30%; ^1^H NMR (600 MHz, CDCl_3_) δ 9.12 (s, 1H), 8.82 (d, *J* = 18.5 Hz, 1H), 8.61 (d, *J* = 4.2 Hz, 1H), 8.25 (t, *J* = 18.7 Hz, 1H), 7.83 (dd, *J* = 27.7, 7.8 Hz, 3H), 7.70–7.57 (m, 2H), 7.37 (dd, *J* = 7.7, 4.9 Hz, 1H), 7.28–7.14 (m, 2H), 6.69 (d, *J* = 7.6 Hz, 1H), 6.63 (s, 1H), 4.97 (s, 2H), 4.91 (dt, *J* = 13.4, 6.7 Hz, 1H), 3.79 (t, *J* = 5.7 Hz, 2H), 3.33 (t, *J* = 6.5 Hz, 2H), 1.94–1.85 (m, 2H), 1.63 (t, *J* = 9.0 Hz, 6H). ^13^C NMR (201 MHz, CDCl_3_) δ 149.85, 149.62, 148.23, 136.50, 134.40, 120.75, 117.76, 96.16, 61.43, 42.39, 32.23, 22.81; HRMS (ESI+) *m*/*z* calcd. for C_28_H_30_N_8_O [M + H]^+^: 495.2621, found: 495.2625.

##### 3-((4-(6-(([2,3′-Bipyridin]-5-ylmethyl)amino)-9-isopropyl-9*H*-purin-2-yl)phenyl)amino)propan-1-ol (**20d**)

Yield: 31%; ^1^H NMR (400 MHz, CDCl_3_) δ 9.18 (d, *J* = 1.8 Hz, 1H), 8.84 (s, 1H), 8.65 (dd, *J* = 4.7, 1.3 Hz, 1H), 8.39–8.26 (m, 3H), 7.92 (dd, *J* = 8.2, 1.9 Hz, 1H), 7.79 (s, 1H), 7.70 (d, *J* = 8.1 Hz, 1H), 7.40 (dd, *J* = 7.9, 4.8 Hz, 1H), 6.69 (d, *J* = 8.7 Hz, 2H), 6.42 (s, 1H), 5.07 (s, 2H), 4.94 (dt, *J* = 13.5, 6.7 Hz, 1H), 3.85 (t, *J* = 5.9 Hz, 2H), 3.38 (t, *J* = 6.5 Hz, 2H), 1.93 (dt, *J* = 12.3, 6.2 Hz, 2H), 1.67 (t, *J* = 9.1 Hz, 6H). ^13^C NMR (101 MHz, CDCl_3_) δ 153.78, 149.80, 149.66, 148.22, 136.56, 134.28, 129.59, 123.58, 120.42, 112.31, 61.36, 46.74, 41.44, 31.93, 22.76; HRMS (ESI+) *m*/*z* calcd. for C_28_H_30_N_8_O [M + H]^+^: 495.2621, found: 495.2630.

#### 3.1.7. General Procedure for Synthesis of **22a–d**

Respective aminoalkylalcohol (1.2 eq.) and triethylamine (3 eq.) were added to a solution of **21a** or **21b** (0.05 mmol) in *n*-butanol (1 mL) under N_2_ atmosphere at rt. The reaction mixture was reacted in microwave at 120 °C for 2 h. After the reaction was completed, the mixture was diluted with EtOAc, then washed with saturated aq. NaHCO_3_, water, and brine sequentially. The organic layer was dried over MgSO_4_, filtered, and concentrated under a reduced pressure. The residue was purified by a column chromatography using 5% methanol in dichloromethane to provide the desired products as white solids.

##### 2-((5-(6-(([2,3′-Bipyridin]-5-ylmethyl)amino)-9-isopropyl-9*H*-purin-2-yl)pyridin-2-yl)amino)ethan-1-ol (**22a**)

Yield 52%; ^1^H NMR (400 MHz, CDCl_3_) δ 9.14 (dd, *J* = 6.8, 1.8 Hz, 2H), 8.76 (d, *J* = 1.8 Hz, 1H), 8.61 (dd, *J* = 4.7, 1.3 Hz, 1H), 8.42 (dd, *J* = 8.8, 2.2 Hz, 1H), 8.29–8.23 (m, 1H), 7.82 (dd, *J* = 8.1, 2.2 Hz, 1H), 7.76 (s, 1H), 7.66 (d, *J* = 8.1 Hz, 1H), 7.36 (dd, *J* = 7.6, 4.8 Hz, 1H), 6.48 (d, *J* = 8.7 Hz, 1H), 6.40 (s, 1H), 5.22 (s, 1H), 4.99 (d, *J* = 4.7 Hz, 2H), 4.86 (dt, *J* = 13.6, 6.8 Hz, 1H), 3.88–3.82 (m, 2H), 3.58 (dd, *J* = 9.4, 5.1 Hz, 2H), 1.63 (d, *J* = 6.8 Hz, 6H); ^13^C NMR (101 MHz, CDCl_3_) δ 159.46, 157.51, 154.15, 153.87, 149.90, 149.65, 148.41, 148.28, 137.77, 137.48, 136.57, 134.75, 134.39, 124.54, 123.70, 120.48, 118.90, 107.73, 77.48, 77.16, 76.84, 63.44, 47.23, 45.59, 22.80; HRMS (ESI+) *m*/*z* calcd. for C_26_H_27_N_9_O [M + H]^+^ 482.2417, found 482.2431.

##### 3-((5-(6-(([2,3′-Bipyridin]-5-ylmethyl)amino)-9-isopropyl-9*H*-purin-2-yl)pyridin-2-yl)amino)propan-1-ol (**22b**)

Yield 40%; ^1^H NMR (400 MHz, CDCl_3_) δ 9.08 (s, 2H), 8.71 (d, *J* = 1.8 Hz, 1H), 8.55 (dd, *J* = 4.8, 1.5 Hz, 1H), 8.33 (dd, *J* = 8.8, 2.2 Hz, 1H), 8.24–8.17 (m, 1H), 7.78 (dd, *J* = 8.1, 2.2 Hz, 1H), 7.69 (s, 1H), 7.61 (d, *J* = 8.1 Hz, 1H), 7.30 (dd, *J* = 8.0, 4.8 Hz, 1H), 6.37 (d, *J* = 8.8 Hz, 2H), 4.91 (dd, *J* = 16.3, 5.8 Hz, 3H), 4.78 (dt, *J* = 13.7, 6.8 Hz, 1H), 3.59 (t, *J* = 5.6 Hz, 2H), 3.56–3.51 (m, 2H), 1.75–1.67 (m, 2H), 1.56 (d, *J* = 6.8 Hz, 6H). ^13^C NMR (101 MHz, CDCl_3_) δ 163.42, 161.69, 157.13, 153.23, 152.96, 151.98, 151.44, 141.40, 140.84, 138.97, 127.94, 127.46, 124.85, 111.06, 63.04, 51.07, 42.42, 36.17, 26.40; HRMS (ESI+) *m*/*z* calcd. for C_27_H_29_N_9_O [M + H]^+^: 496.2573, found: 496.2570.

##### 2-((4-(6-(([2,3′-Bipyridin]-5-ylmethyl)amino)-9-isopropyl-9*H*-purin-2-yl)pyridin-2-yl)amino)ethan-1-ol] (**22c**)

Yield 47%; ^1^H NMR (400 MHz, CDCl_3_) δ 9.15 (d, *J* = 1.7 Hz, 1H), 8.82 (d, *J* = 1.5 Hz, 1H), 8.63 (d, *J* = 3.4 Hz, 1H), 8.28 (d, *J* = 8.0 Hz, 1H), 8.10 (d, *J* = 5.5 Hz, 1H), 7.89 (s, 1H), 7.86 (dd, *J* = 8.2, 2.1 Hz, 1H), 7.69 (d, *J* = 8.1 Hz, 1H), 7.62 (d, *J* = 4.6 Hz, 1H), 7.49 (s, 1H), 7.38 (dd, *J* = 7.9, 4.8 Hz, 1H), 6.49 (s, 1H), 4.99 (s, 2H), 4.91 (dt, *J* = 13.4, 6.8 Hz, 1H), 3.83 (t, *J* = 4.8 Hz, 2H), 3.57 (d, *J* = 3.8 Hz, 2H), 1.65 (d, *J* = 6.8 Hz, 6H); ^13^C NMR (101 MHz, CDCl_3_) δ 159.45, 156.70, 154.09, 153.88, 149.93, 149.61, 148.17, 147.74, 147.40, 138.67, 136.43, 134.45, 134.23, 134.14, 123.60, 120.46, 112.11, 107.23, 77.35, 77.03, 76.71, 63.55, 47.28, 45.72, 22.74; HRMS (ESI+) *m*/*z* calcd. for C_26_H_27_N_9_O [M + H]^+^ 482.2417, found 482.2420.

##### 3-((4-(6-(([2,3′-Bipyridin]-5-ylmethyl)amino)-9-isopropyl-9*H*-purin-2-yl)pyridin-2-yl)amino)propan-1-ol] (**22d**)

Yield 49%; ^1^H NMR (400 MHz, CDCl_3_) δ 9.13 (s, 1H), 8.78 (s, 1H), 8.61 (d, *J* = 4.1 Hz, 1H), 8.23 (d, *J* = 7.9 Hz, 1H), 8.09 (d, *J* = 5.4 Hz, 1H), 7.83 (s, 1H), 7.81 (d, *J* = 8.1 Hz, 1H), 7.64 (d, *J* = 8.1 Hz, 1H), 7.54 (d, *J* = 5.4 Hz, 1H), 7.39 (s, 1H), 7.35 (dd, *J* = 7.8, 4.8 Hz, 1H), 6.76 (s, 1H), 4.96 (s, 2H), 4.89 (dt, *J* = 13.4, 6.9 Hz, 1H), 3.67–3.63 (m, 2H), 3.56 (d, *J* = 5.5 Hz, 2H), 1.80–1.73 (m, 2H), 1.62 (d, *J* = 6.8 Hz, 6H); ^13^C NMR (101 MHz, CDCl_3_) δ 159.21, 156.56, 154.20, 154.01, 150.06, 149.70, 148.29, 146.31, 138.87, 136.50, 134.60, 134.37, 134.30, 123.74, 120.64, 111.58, 107.36, 77.48, 77.16, 76.84, 58.93, 47.43, 38.60, 33.48, 22.88.

#### 3.1.8. *N*-([2,3′-Bipyridin]-5-ylmethyl)-2-hydrazineyl-9-isopropyl-9H-purin-6-amine (**23**)

Intermediate **16f** (600 mg) and NH_2_NH_2_∙H_2_O (0.5 mL) were mixed in *n*-butanol (1 mL) with stirring at rt, then heated to 150 °C with stirring overnight. After the mixture was cooled to rt, water (10 mL) was added, and the solid (300 mg) was filtered (48% yield). ^1^H NMR (400 MHz, DMSO-*d_6_*) δ 9.23 (d, *J* = 1.6 Hz, 1H), 8.76 (s, 1H), 8.62 (dd, *J* = 4.7, 1.6 Hz, 1H), 8.45–8.36 (m, 1H), 8.11–7.83 (m, 4H), 7.57–7.46 (m, 1H), 7.40 (s, 1H), 4.80–4.49 (m, 3H), 4.05 (s, 2H), 1.48 (d, *J* = 6.8 Hz, 6H). ^13^C NMR (101 MHz, DMSO-*d*_6_) δ 161.99, 152.64, 150.13, 148.14, 134.48, 134.23, 124.22, 120.67, 46.05, 22.64.

#### 3.1.9. *N*-([2,3′-Bipyridin]-5-ylmethyl)-2-(5-amino-3-methyl-1H-pyrazol-1-yl)-9-isopropyl-9H-purin-6-amine (**24**)

Intermediate **23** (40 mg, 1 eq.) and 3-oxobutanenitrile (1.5 eq) were mixed in ethanol (2 mL) at rt, then refluxed with stirring. After the reaction was completed, ethanol was evaporated and subjected to a column chromatography using 2% methanol in dichloromethane as eluent. Yield 26%; ^1^H NMR (400 MHz, CDCl_3_) δ 9.20 (s, 1H), 8.79 (s, 1H), 8.67 (d, *J* = 4.8 Hz, 1H), 8.40–8.28 (m, 1H), 7.90 (d, *J* = 6.1 Hz, 1H), 7.83 (s, 1H), 7.74 (d, *J* = 8.1 Hz, 1H), 7.43 (dd, *J* = 7.8, 4.8 Hz, 1H), 6.54 (s, 1H), 5.40 (s, 1H), 4.98 (s, 3H), 2.30 (d, *J* = 6.1 Hz, 3H), 1.93 (d, *J* = 70.3 Hz, 2H), 1.66–1.56 (m, 6H). ^13^C NMR (101 MHz, CDCl_3_) δ 154.10, 151.38, 149.96, 149.39, 149.25, 148.20, 137.55, 136.34, 134.22, 123.54, 120.32, 90.29, 46.44, 23.05, 14.42; HRMS (ESI+) *m*/*z* calcd. for C_23_H_24_N_10_ [M + H]^+^: 441.2264, found: 441.2270.

#### 3.1.10. *N*-([2,3′-Bipyridin]-5-ylmethyl)-2-azido-9-isopropyl-9H-purin-6-amine (**25**)

Compound **23** (300 mg) and NaNO_2_ (250 mg, 1.5 eq.)/HCl (1 mL) were mixed in water (4 mL) at 4 °C and stirred for 30 m, then we added, dropwise, NaN_3_ (150 mg, 1.2 eq.) dissolved in 2 mL of water and stirred for 12 h at rt. The reaction mixture was diluted with water (100 mL) and extracted with EtOAc (100 mL). The organic layer was washed with brine, dried over MgSO_4_, and concentrated under a reduced pressure. The crude mixture was purified by a column chromatography (1% methanol in dichloromethane) to afford **25** (200 mg, yield: 40%). ^1^H NMR (400 MHz, CDCl_3_) δ 9.10 (s, 1H), 8.66 (d, *J* = 1.5 Hz, 1H), 8.57 (s, 1H), 8.29–8.14 (m, 1H), 7.75 (dd, *J* = 8.1, 2.2 Hz, 1H), 7.65–7.54 (m, 2H), 7.32 (dd, *J* = 7.8, 4.8 Hz, 1H), 6.95 (d, *J* = 6.6 Hz, 1H), 4.80 (s, 2H), 4.72–4.56 (m, 1H), 1.48 (d, *J* = 6.7, 6H). ^13^C NMR (101 MHz, CDCl_3_) δ 156.38, 153.95, 149.91, 149.68, 148.18, 137.38, 136.65, 134.52, 134.27, 133.55, 123.61, 120.30, 77.39, 77.27, 77.07, 76.75, 47.06, 22.75, 22.62.

#### 3.1.11. General Procedure for Synthesis of **26a–b**

Either 2-propyn-1-ol or 3-butyn-1-ol (0.24 mmol) was added to a solution of compound **25** (0.171 mmol) in a mixture of *t*-butanol (2 mL) and water (2 mL). Subsequently, freshly prepared 1 M sodium ascorbate solution (174 μL, 0.15 mmol) and 7.5% solution of CuSO_4_∙5H_2_O (288 μL, 0.06 mmol) were added to the reaction mixture and stirred at rt overnight. The solvent was evaporated and the residue was purified on flash chromatography using 2% methanol in dichloromethane.

##### (1-(6-(([2,3′-Bipyridin]-5-ylmethyl)amino)-9-isopropyl-9*H*-purin-2-yl)-1*H*-1,2,3-triazol-4-yl)methanol (**26a**)

Yield 30%; ^1^H NMR (400 MHz, DMSO-*d*_6_) δ 9.23 (s, 1H), 9.03 (s, 1H), 8.83 (s, 1H), 8.65 (d, *J* = 24.8 Hz, 2H), 8.40 (s, 2H), 8.01 (s, 2H), 7.51 (s, 1H), 5.33 (t, *J* = 5.7 Hz, 1H), 4.92–4.75 (m, 3H), 4.64 (d, *J* = 5.7 Hz, 2H), 1.57 (d, *J* = 6.7 Hz, 6H). ^13^C NMR (201 MHz, DMSO-*d_6_*) δ 153.02, 150.24, 150.07, 148.75, 148.18, 140.67, 137.42, 134.31, 121.99, 120.87, 55.37, 47.35, 41.34, 22.68.; HRMS (ESI+) *m*/*z* calcd. for C_22_H_22_N_10_O [M + H]^+^: 443.2056, found: 443.2052.

##### 2-(1-(6-(([2,3′-Bipyridin]-5-ylmethyl)amino)-9-isopropyl-9*H*-purin-2-yl)-1*H*-1,2,3-triazol-4-yl)ethan-1-ol (**26b**)

Yield: 40%; ^1^H NMR (400 MHz, CDCl_3_) δ 9.07 (d, *J* = 1.7 Hz, 1H), 8.72 (d, *J* = 1.8 Hz, 1H), 8.56 (dd, *J* = 4.8, 1.5 Hz, 1H), 8.29 (s, 1H), 8.22 (d, *J* = 8.0 Hz, 1H), 7.88–7.77 (m, 2H), 7.64 (d, *J* = 8.2 Hz, 1H), 7.32 (dd, *J* = 7.7, 4.9 Hz, 1H), 6.70 (s, 1H), 4.96–4.76 (m, 3H), 3.95 (t, *J* = 5.8 Hz, 2H), 2.99 (t, *J* = 5.8 Hz, 2H), 1.54 (d, *J* = 6.8 Hz,, 6H). ^13^C NMR (101 MHz, CDCl_3_) δ 153.62, 149.62, 149.19, 147.82, 145.39, 138.49, 136.82, 134.33, 133.39, 123.63, 121.17, 120.28, 118.95, 61.29, 47.21, 29.01, 22.76; HRMS (ESI+) *m*/*z* calcd. for C_23_H_24_N_10_O [M + H]^+^: 457.2213, found: 457.2219.

### 3.2. Docking Analysis

Compound **30d** was drawn using ChemDraw 20 and converted to 3D-structure via Open Babel 2.3.1 with subsequent energy minimization by means of MMFF94 force field. Molecular docking was conducted through our in-house code, VnsDock, based on Autodock4 scoring function [31] and variable neighborhood search enhanced with L-BFGS-B refinement. In the co-crystal structure of the complex of CDK12, DDB2, and R-CR8 (PDB ID: 6td3), the center of R-CR8 with the coordinates of −62.49(x), 24.44(y), −4.64(z) was defined as the center of grid box with its width of 30 Å each in x, y, z direction. Then R-CR8, cofactor, and water molecules were extracted from the complex. Chimera [32] was used to generate all hydrogens and to assign Gasteiger partial charges for each atom. Grid maps per each ligand atom type were generated with a spacing of 0.375 Å between the grid points. Docking simulation was performed with a stop condition of 3 million number of energy evaluations during 10 iterative runs. The best energy pose was analyzed about its specific intermolecular interactions by virtue of PLIP [33] and visualized using Pymol 2.5 software. 

### 3.3. Bioassays

#### 3.3.1. Antibodies

For Western blot analysis, the following antibodies were used: anti-Pol II CTD (2629S, Cell Signaling Technology, Danvers, MA, USA), anti-Pol II p-CTD (Ser2) (13499S, Cell Signaling Technology), anti-cyclinK (A301-939A-M, Bethyl Laboratories), anti-IRS1 (3407S, Cell Signaling Technology), anti-WNT1 (SC-514531, Santa Cruz Biotechnology, Dallas, TX, USA), anti-β-actin (MAB1501R, Millipore, Burlington, MA, USA), and horseradish peroxidase (HRP)-conjugated secondary antibodies (anti-mouse GTX213112-01; anti-rabbit GTX213110-01) (Genetex, Irvine, CA, USA). 

#### 3.3.2. Cell Culture

Breast-cancer cell lines (SK-Br3, HCC-1954) were purchased from Korea Cell Line Bank (Seoul, Korea) and were cultured in RPMI medium supplemented with 10% FBS and penicillin/streptomycin (Welgene, Seoul, Korea). All cell lines were maintained at 37 °C in a cell incubator in the presence of 5% CO_2_.

#### 3.3.3. In Vitro Kinase Assay

In vitro CDK12/cyclinK assays for all compounds and in vitro kinome-wide inhibition profiling of **30d** at 10 μM were performed by a CRO company (Reaction Biology Corp., San Diego, CA, USA).

#### 3.3.4. Antiproliferation Assay

Breast-cancer cells in culture media (100 μL volume/well, 5000 SK-Br3 cells/well, 1000 HCC-1954 cells/well) were seeded in tissue culture-treated 96-well plates, and incubated at a cell incubator for 24 h. Serially diluted compounds (3-fold, 10 point from 10 mM, duplicated) were pre-plated and 500 nL of compound solutions were pin-transferred to the cells in the assay plates using pintool system (JANUS liquid handler, PerkinElmer, Waltham, MA, USA) and incubated at a cell incubator for 72 h. Cell-titer Glo^TM^ reagent (50 μL, Promega Corp., Madison, WI, USA) was added to each well, and the luminescence signal was read using an Envision^TM^ plate reader (PerkinElmer, Waltham, MA, USA). The titration curve fitting and IC_50_ values were generated using Prism 7.0 s/w (GraphPad, San Diego, CA, USA).

#### 3.3.5. Western Blot

Cells were lysed with lysis buffer containing 50 mM Tris pH 7.4, 150 mM NaCl, 1 mM EDTA, 1% NP-40, and 0.25% sodium deoxycholate supplemented with protease-inhibitor and phosphatase-inhibitor cocktails. The lysate samples (25 μg/lane) were loaded onto 6–12% sodium dodecyl sulfate polyacrylamide gel and separated by electrophoresis (SDS-PAGE). The proteins were transferred to nitrocellulose membrane (Gelman Sciences, Ann Arbor, MI, USA) by electroblotting. The membranes were blocked with 5% non-fat dry milk (MB Cell, Seoul, Korea), incubated with primary antibodies for overnight at 4 °C. After the washing steps were performed with TBST solution, the membranes were incubated with HRP-conjugated secondary antibodies for 1 h at rt. After the secondary washing steps using TBST solution, protein bands on the membranes were visualized using enhanced chemiluminescence ECL detection reagent (Biorad Laboratories, Hercules, CA, USA).

#### 3.3.6. In Vitro Liver Microsomal Stability Assay

Each compound (final 1 μM, duplicated) was added to each 0.1 M PBS solution (pH 7.4) containing liver microsome of three different species (human, dog, mouse). After a brief (5 m) incubation at 37 °C, the NADPH regeneration system (Promega Corp., Madison, WI, USA) was added, then the mixture was incubated at 37 °C for 30 m. The reaction was terminated by adding acetonitrile containing an internal standard (chlorpropamide), then the mixture was centrifuged at 15,000 rpm, 4 °C for 5 m. The supernatant solution was injected to LC/MS/MS system (TSQ Vantage Triple-Stage Quadrupole Mass Spectrometer, ThermoFisher Scientific, Waltham, MA, USA). The remaining substrates were analyzed with Xcalibur 4.4 s/w using an MRM (multiple reaction monitoring) quantitation mode.

## 4. Conclusions

We designed novel 2,6,9-trisubstituted purine CDK12 inhibitors based on the X-ray co-crystal structures of a purine-based CDK12 inhibitor (**2**) and cyclinK degrader R-CR8. We provided comprehensive SAR results with regard to in vitro CDK12/cyclinK inhibition and growth inhibition of trastuzumab-sensitive HER2+ SK-Br3 cells, as well as trastuzumab-resistant HER2+ HCC1954 cells. We found **30d** and **30e,** which showed a potent in vitro activity (CDK12/cyclinK IC_50_ = 21 nM and 85 nM, respectively), and a potent growth-inhibitory activity against the both HER2+ breast-cancer cell lines (SK-Br3 GI_50_ = 46–52 nM, HCC1954 GI_50_ = 34–36 nM). We also observed the structure-property relationship for a subset of potent analogues, and found that **30e** was most suitable analogue in terms of in vitro metabolic stability and CYP activity conservation. In the both cells, **30d** and **30e** at 40, 200 nM potently and dose-dependently downregulated the levels of cyclinK, PolII p-CTD (Ser2), and CDK12 downstream gene expressions. Kinome-wide inhibition profiling result revealed that **30d** also potently inhibits multiple other CDKs, including CDK1/2/3/5/7/9/18 and other family kinases such as EPH receptors. Our SAR analysis suggested that targeting multiple CDKs along with CDK12/cyclinK might be advantageous in overcoming trastuzumab resistance. Compound **30d** also showed a modest level of synergism with trastuzumab in both HER2+ breast-cancer cells. Thus, our CDK12 inhibitors could be developed to treat trastuzumab-resistant HER2+ breast cancers and escalate the efficacy of trastuzumab, as well. Our potent CDK12 inhibitors may serve as a good starting point in developing novel therapy for HER2+ breast cancers.

## Figures and Tables

**Table 1:** Structure-activity relationship for the bi-pyridyl part at 6 position.

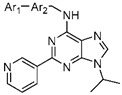				
Compound	Ar_1_-Ar_2_	CDK12/CyclinK (IC_50_/μM)	SK-Br3 (GI_50_/μM) *	HCC1954 (GI_50_/μM) *
**17a**	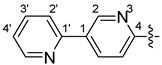	0.433	0.151 ± 0.025	0.106 ± 0.011
**17b**	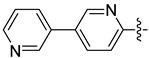	0.582	0.862 ± 0.178	0.627 ± 0.128
**17c**	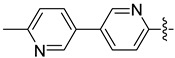	1.109	24.560 ± 4.992	13.580 ± 1.160
**17d**	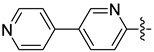	0.627	0.741 ± 0.034	0.887 ± 0.040
**17e**	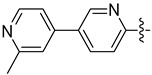	0.481	0.277 ± 0.038	0.230 ± 0.044
**17f**	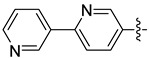	0.221	0.410 ± 0.034	0.247 ± 0.030
**17g**	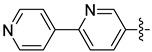	0.153	0.804 ± 0.095	0.494 ± 0.048

* These values are means and standard deviations from three independent assays.

**Table 2:** Structure-activity relationship for aryl substituents at 2 position.

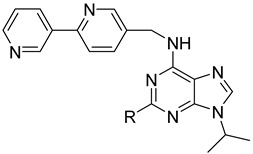				
Compound	R	CDK12/CyclinK (IC_50_/μM)	SK-Br3 (GI_50_/μM) *	HCC1954 (GI_50_/μM) *
**18a**	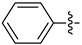	0.623	1.692 ± 0.243	1.116 ± 0.260
**19a**	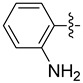	0.619	2.607 ± 0.899	2.634 ± 0.448
**20a**	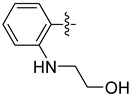	0.799	2.304 ± 0.318	1.557 ± 0.116
**20b**	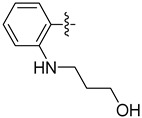	3.012	11.240 ± 1.117	6.051 ± 0.351
**19b**	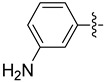	0.170	0.614 ± 0.055	0.341 ± 0.059
**20c**	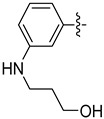	0.257	0.732 ± 0.177	0.502 ± 0.025
**19c**	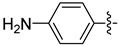	0.150	0.795 ± 0.063	0.497 ± 0.088
**20d**	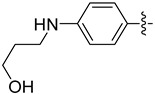	0.499	1.093 ± 0.127	0.907 ± 0.068
**18b**	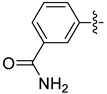	0.504	1.625 ± 0.248	1.137 ± 0.304
**18c**	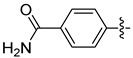	0.484	3.711 ± 0.450	3.141 ± 0.201
**18d**	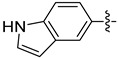	0.155	1.496 ± 0.112	1.351 ± 0.243
**18e**	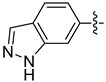	0.179	0.608 ± 0.115	0.399 ± 0.051
**18f**	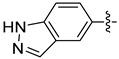	0.104	2.580 ± 0.167	1.865 ± 0.148

* These values are means and standard deviations from three independent assays.

**Table 3:** Structure-activity relationship for heteroaryl substituents at 2 position.

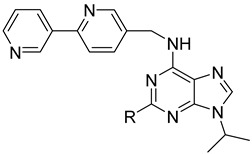				
Compound	R	CDK12/CyclinK (IC_50_/μM)	SK-Br3 (GI_50_/μM) *	HCC1954 (GI_50_/μM) *
**18g**	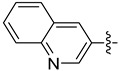	0.486	3.715 ± 0.557	4.230 ± 0.214
**17f**	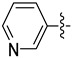	0.221	0.410 ± 0.034	0.247 ± 0.030
**21a**	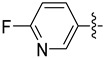	0.147	1.696 ± 0.165	1.335 ± 0.393
**18h**	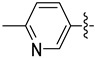	0.090	1.270 ± 0.330	0.942 ± 0.125
**18i**	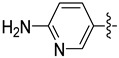	0.065	0.376 ± 0.067	0.278 ± 0.007
**22a**	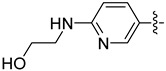	0.053	0.262 ± 0.021	0.174 ± 0.019
**22b**	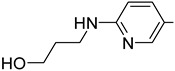	0.304	1.183 ± 0.118	0.977 ± 0.070
**21b**	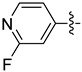	0.164	1.679 ± 0.267	1.274 ± 0.021
**18j**	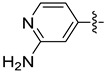	0.094	0.710 ± 0.024	0.354 ± 0.060
**22c**	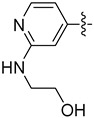	0.086	0.473 ± 0.087	0.433 ± 0.052
**22d**	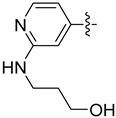	0.113	0.480 ± 0.026	0.387 ± 0.037
**18k**	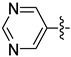	0.051	0.318 ± 0.149	0.310 ± 0.041
**18l**	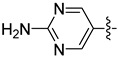	0.087	0.242 ± 0.042	0.162 ± 0.011
**18m**	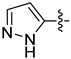	0.501	1.717 ± 0.336	1.132 ± 0.050
**18n**	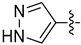	1.433	1.993 ± 0.120	1.172 ± 0.027
**24**	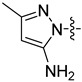	2.345	5.160 ± 0.365	3.436 ± 0.177
**26a**	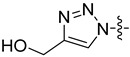	2.051	13.260 ± 3.154	9.967 ± 1.857
**26b**	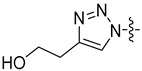	0.539	8.303 ± 0.882	6.158 ± 0.429

* These values are means and standard deviations from three independent assays.

**Table 4:** Structure-activity relationship for derivatization at 9 position.

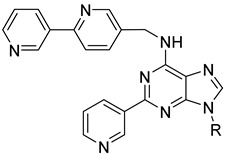				
Compound	R	CDK12/CyclinK (IC_50_/μM)	SK-Br3 (GI_50_/μM) *	HCC1954 (GI_50_/μM) *
**17f**	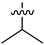	0.221	0.410 ± 0.034	0.247 ± 0.030
**28a**	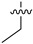	0.016	0.215 ± 0.053	0.142 ± 0.014
**28b**	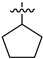	0.293	2.597 ± 0.310	2.140 ± 0.176
**28c**	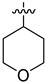	>10	>100	44.360 ± 2.461

* These values are means and standard deviations from three independent assays.

**Table 5:** Structure-activity relationship of potent analogues.

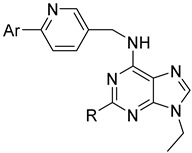					
Compound	Ar	R	CDK12/CyclinK (IC_50_/μM) *	SK-Br3 (GI_50_/μM) *	HCC1954 (GI_50_/μM) *
**30a**	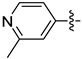	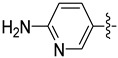	0.029 ± 0.015	0.136 ± 0.016	0.104 ± 0.003
**30b**	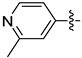	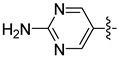	0.028 ± 0.008	0.090 ± 0.008	0.080 ± 0.007
**30c**	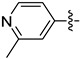	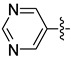	0.064 ± 0.012	0.189 ± 0.057	0.157 ± 0.022
**30d**	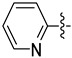	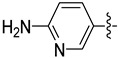	0.021 ± 0.007	0.046 ± 0.007	0.036 ± 0.005
**30e**	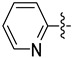	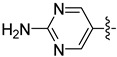	0.085 ± 0.011	0.052 ± 0.003	0.034 ± 0.002
THZ531			0.051 ± 0.007	0.030 ± 0.006	0.195 ± 0.050
Dinaciclib			0.0066 ± 0.0002	0.012 ± 0.001	0.012 ± 0.001
**2**			0.0086 ± 0.0017	0.094 ± 0.012	0.069 ± 0.006
R-CR8 (**9**)			0.287 ± 0.036	0.101 ± 0.014	0.083 ± 0.011

* These values are means and standard deviations from three independent assays.

**Table 6:** In vitro liver metabolic stability and activity against CYPs of potent analogues.

Compound	Liver Microsomal Stability (% Remaining)			CYP % Activity at 10 μM				
	Human	Dog	Mouse	1A2	2C9	2C19	2D6	3A4
**30a**	32.2	45.1	35.3	59.3	73.0	76.8	89.6	38.9
**30b**	40.6	53.6	45.0	74.9	82.9	75.4	85.7	72.4
**30c**	27.3	79.5	34.0	68.6	67.5	73.9	88.8	31.8
**30d**	64.8	46.2	49.9	74.7	57.9	68.1	81.0	43.0
**30e**	100	67.6	85.0	87.6	81.6	72.6	92.1	92.8

## Data Availability

Data is contained within the article and Supplementary Materials.

## Supplementary Materials

The following supporting information can be downloaded at: https://www.mdpi.com/article/10.3390/ph15091041/s1. Figure S1: KinomeScan^TM^ profiling data for 10 μM dinaciclib against a panel of 456 human wild type kinases available in HMS LINCS database (https://lincs.hms.harvard.edu/db/datasets/20128/results accessed on 8 August 2022); Table S1: Kinome-wide inhibition profiling data (% remaining activity) of 10 μM **30d**; Copies of ^1^H and ^13^C spectral data for key intermediates and final compounds.
