# Supplementary material for: Novel 2,6,9-Trisubstituted Purines as Potent CDK Inhibitors Alleviating Trastuzumab-Resistance of HER2-Positive Breast Cancers
Source: Pharmaceuticals (Basel). 2022 Aug 23;15(9):1041. doi: 10.3390/ph15091041 (PMC9506414; doi:10.3390/ph15091041)
Supplement: Supplementary file 1 [file pharmaceuticals-15-01041-s001.zip › pharmaceuticals-1884414-supplementary.pdf]

Supplementary Data

Novel 2,6,9-trisubstituted purines as potent CDK inhibitors alleviating trastuzumab-resistance of HER2-positive breast cancers

Ratnakar Reddy Kuchukulla,[1,¶] Injeoung Hwang,[1,2,¶] Sang Won Park,[1] Sojeong Moon,[1] Suhn Hyung Kim,[2] Sumin Kim,[1] Hwan Won Chung,[3] Mi-Jung Ji,[4] Hyun-Mee Park,[4] Gu Kong,[1,2,5,*] and Wooyoung Hur,[1,2,*]

1. HY-KIST Bioconvergence, Hanyang University, 222 Wangsimniro, Seongdong-gu, Seoul 04763, South Korea
2. Medicinal Materials Research Center, Korea Institute of Science and Technology (KIST), 5 Hwarangro 14 gil, Seongbuk-gu, Seoul 02792, South Korea
3. Computational Science Research Center, Korea Institute of Science and Technology (KIST), 5 Hwarangro 14 gil, Seongbuk-gu, Seoul 02792, South Korea
4. Advanced Analysis and Data Center, Korea Institute of Science and Technology (KIST), 5 Hwarangro 14 gil, Seongbuk-gu, Seoul 02792, South Korea
5. Department of Pathology, Hanyang University College of Medicine, 222 Wangsimniro, Seongdong-gu, Seoul 04763, South Korea

¶These authors are equal contributors.

*Corresponding authors: Wooyoung Hur (whur@kist.re.kr) and Gu Kong (gkong@hanyang.ac.kr)

Contents:

1. Supplementary Figure S1 -- 2
2. Supplementary Table S1 -- 4
3. Copies of ^{1}H and ^{13}C spectral data for key intermediates ---------------------------------- 8
4. Copies of ^{1}H and ^{13}C spectral data for final compounds --------------------------------- 22

Dinaciclib

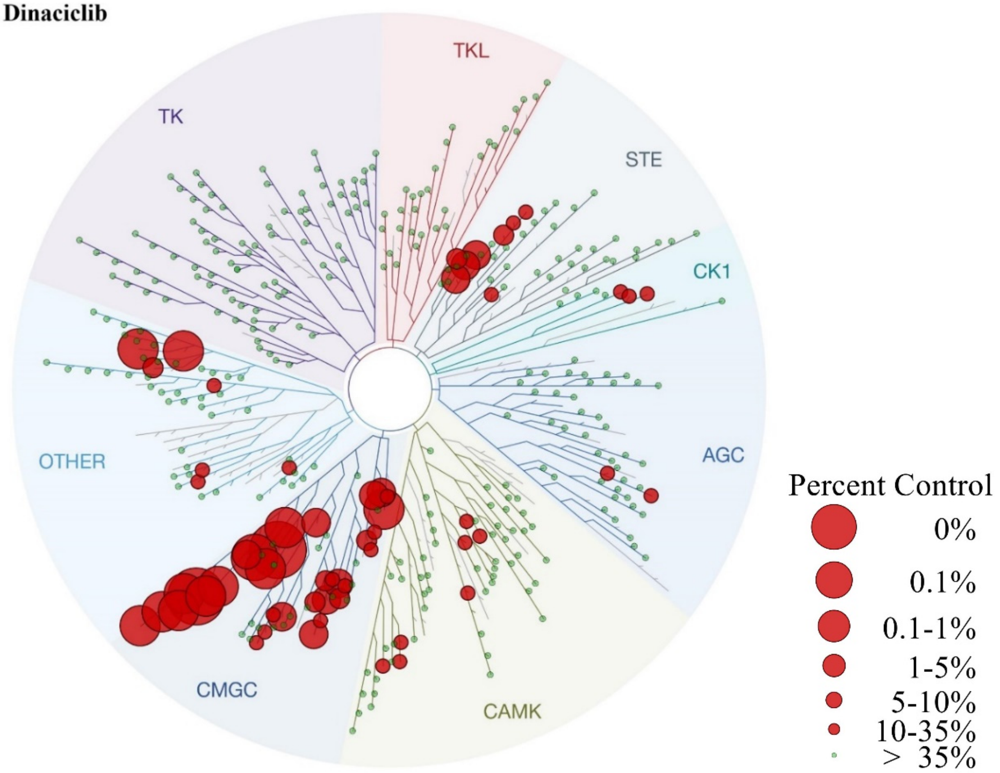

No	Kinase	% control	No	Kinase	% control
1	CDK9	0	19	CDK4-cyclinD3	2.0
2	PFTK1	0	20	ERK5	2.9
3	CDK4-cyclinD1	0.1	21	GSK3A	3.0
4	PCTK3	0.2	22	TAOK1	3.1
5	CDC2L5	0.3	23	HIPK2	3.6
6	CDK2	0.3	24	TAOK3	3.8
7	CDK3	0.3	25	CLK2	4.2
8	PCTK1	0.3	26	HIPK3	4.4
9	CDK7	0.4	27	TAOK2	4.8
10	PFTAIRE2	0.4	28	CLK4	5.1
11	TLK2	0.5	29	DYRK1A	6.0
12	ICK	0.9	30	DYRK1B	6.0
13	PCTK2	0.9	31	PAK3	7.8
14	TLK1	0.9	32	STK36	7.8
15	ERK8	1.0	33	CDKL1	9.1
16	PFPK5(P.falciparum)	1.2	34	YSK1	9.2
17	CDKL5	1.6	35	CDKL2	10
18	CDK5	1.8			

Supplementary Figure S1. KinomeScan™ profiling data for 10 μM dinaciclib against a panel of 456 human wild type kinases available in HMS LINCS database (https://lincs.hms.harvard.edu/db/datasets/20128/results). (Top) Percent binding of each kinase to immobilized ligands, illustrated using a web-based TREE*spot*™ visualization tool (https://www.discoverx.com/services/drug-discovery-devel1opment-services/treespot-data-analysis). (Bottom) The list of top-ranking kinases that are tightly bound to 10 μM of dinaciclib.

Supplementary Table S1. Kinome-wide inhibition profiling data (% remaining activity) of 10 μM **30d**.

No	Kinase	% remaining
1	ABL1	61.2
2	ABL2	56.1
3	ACK1	13.7
4	AKT1	94.9
5	AKT2	100.1
6	AKT3	96.1
7	ALK	59.7
8	ACVRL1	105.2
9	ACVR1	117.9
10	BMPR1A	125.6
11	ACVR1B	132.4
12	TGFBR1	111.9
13	BMPR1B	115.3
14	ARAF	79.5
15	ARK5	20.8
16	ASK1	18.4
17	AurkA	33.4
18	AurkB	73
19	AurkC	25.5
20	AXL	44.2
21	BLK	66.8
22	BMPR2	52.4
23	BMX	141.4
24	BRAF	60.8
25	BRK	46.6
26	BRSK1	92.8
27	BRSK2	97.5
28	BTK	102.6
29	Kit	14.2
30	MERTK	71
31	MET	96.4
32	Src	60.1
33	CAMK1a	34
34	CAMK1b	66.8
35	CAMK1d	58
36	CAMK1g	54.3
37	CAMK2a	64
38	CAMK2b	65.2
39	CAMK2d	61.7
40	CAMK2g	105.1
41	CAMK4	95.2
42	CAMKK1	18.7
43	CAMKK2	10.6
44	CDC7/DBF4	98.2
45	CDK1/cyclinB	2.1
46	PFTK1	14
47	CDK16	17.5
48	PCTK2	7.3
49	PCTK3	2.5
50	CDK2/cyclinA	1.2
51	CDK3/cyclinE	1
52	CDK4/cyclinD1	33.5
53	CDK4/cyclinD3	28.6
54	CDK5/p35	0.7
55	CDK6/cyclinD1	24.2
56	CDK7/cyclinH	3.8
57	CDK9/cyclinT1	0.8
58	CDK12/cyclinK	1.1
59	CHEK1	79.5
60	CHEK2	7.4
61	CSNK1A1	26.5
62	CSNK1A1L	53.1
63	CSNK1D	10.2
64	CSNK1G1	29.8
65	CSNK1E	22.9
66	CSNK1G2	27
67	CSNK1G3	24.4
68	CSNK2A1	84.2
69	CSNK2A2	91.3
70	CLK1	13.7
71	CLK2	11.7
72	CLK3	94.6
73	CLK4	28.7
74	MAP3K8	102.3
75	CSK	54.8
76	CTK	87.6
77	DAPK1	99.7
78	DAPK2	85.7
79	DCAMKL1	72.6
80	DCAMKL2	55
81	DDR1	50.2
82	DDR2	87.2
83	DMPK	90.4
84	DMPK2	92.4
85	DRAK1	79.3
86	DYRK1A	3.9
87	DYRK1B	3.2
88	DYRK2	22.3
89	DYRK3	58.6
90	DYRK4	89.3
91	EGFR	83.7
92	EPHA1	13.2
93	EPHA2	42.3
94	EPHA3	7.8
95	EPHA4	3.6
96	EPHA5	5.9

No	Kinase	% remaining
97	EPHA6	3
98	EPHA7	31.3
99	EPHA8	94
100	EPHB1	13.6
101	EPHB2	2.5
102	EPHB3	66
103	EPHB4	23.2
104	ERBB2	74.2
105	ERBB4	73
106	ERK1	9.4
107	ERK2	10.6
108	ERK5	97.7
109	ERK7	13.5
110	ERN1	50.8
111	ERN2	65.4
112	FAK	52.5
113	FER	52.3
114	FES	6.4
115	FGFR1	28.8
116	FGFR2	33.7
117	FGFR3	30.6
118	FGFR4	87.4
119	FGR	54.6
120	FLT1	36.3
121	FLT3	40.2
122	FLT4	10
123	FMS	35.4
124	FRK	43.5
125	FYN	81
126	MAP4K2	58.4
127	MAP4K3	49.6
128	GRK1	76.3
129	GRK2	99.1
130	GRK3	101.5
131	GRK4	88.3
132	GRK5	91.5
133	GRK6	80.6
134	GRK7	49.7
135	GSK3a	10.6
136	GSK3b	5.8
137	Haspin	79.4
138	HCK	46.5
139	MAP4K4	102.7
140	HIPK1	93
141	HIPK2	81.3
142	HIPK3	94.1
143	HIPK4	43.9
144	HPK1	30.2
145	IGF1R	58
146	IKK-alpha	71.5
147	IKK-beta	83.7
148	IKK-epsilon	80.1
149	INSR	65.1
150	IRAK1	79
151	IRAK2	97.5
152	IRAK4	94.8
153	INSRR	31.1
154	ITK	81.7
155	JAK1	92.1
156	JAK2	87
157	JAK3	99.7
158	JNK1	41.6
159	JNK2	61.7
160	JNK3	48.7
161	VEGFR2	88.6
162	MAP4K5	88.3
163	KSR1	100.3
164	KSR2	88.4
165	LATS1	85
166	LATS2	77.4
167	LCK	83.6
168	ICK	39.6
169	LIMK1	59.9
170	LIMK2	76.5
171	LKB1	0.1
172	LOK	41.4
173	LRRK2	17.7
174	LYN	65.7
175	LYN B	54.4
176	MAK	3.3
177	MAPKAPK2	102.4
178	MAPKAPK3	100.5
179	MAPKAPK5	103.4
180	MARK1	81.5
181	MARK2	74.3
182	MARK3	83.1
183	MARK4	66.9
184	MAST3	39.3
185	MASTL	102.9
186	MEK1	149.4
187	MEK2	176
188	MEK3	73.5
189	MEK5	84.1
190	MAP3K1	87
191	MAP3K2	90
192	MAP3K3	94

No	Kinase	% remaining
193	MEKK6	109
194	MELK	21.2
195	MINK	101.3
196	MKK4	96.4
197	MKK6	95.4
198	MKK7	96.3
199	MLCK	63.4
200	MYLK2	60
201	MLK1	27.9
202	MLK2	62.3
203	MLK3	51.4
204	MLK4	114.4
205	MNK1	89.3
206	MNK2	91.2
207	MRCKa	96.6
208	MRCKb	99.3
209	RPS6KA5(Kin.Dom.1-N-term)	80.5
210	RPS6KA4(Kin.Dom.1-N-term)	115
211	STK23	82.8
212	MST1	62.2
213	MST2	53.5
214	MST3	47
215	MST4	45.8
216	MUSK	10.2
217	MYLK3	105.4
218	MYLK4	4.1
219	MYO3A	71.3
220	MYO3b	68.1
221	NEK1	92.3
222	NEK11	94.3
223	NEK2	91.1
224	NEK3	86.6
225	NEK4	95.8
226	NEK5	83.8
227	NEK6	92.1
228	NEK7	93.2
229	NEK9	90
230	NIM1	76
231	NLK	24.8
232	OSR1	50.8
233	P38-alpha	84.2
234	P38-beta	64.7
235	P38-delta	102.4
236	P38-gamma	118.2
237	S6K1	92.1
238	p70S6Kb/RPS6KB2	92.2
239	PAK1	24.5
240	PAK2	46.7

No	Kinase	% remaining
241	PAK3	29.1
242	PAK4	12.9
243	PAK5	7.6
244	PAK6	57.2
245	PASK	96.3
246	PBK/TOPK	93.5
247	PDGFRa	52.8
248	PDGFRb	34.7
249	PDPK1	105.1
250	PHKg1	16.5
251	PHKg2	56.9
252	PIM1	97.8
253	PIM2	93.8
254	PIM3	86.7
255	PKA	93.1
256	PKAC-beta	104.2
257	PKAC-gamma	88.4
258	PKAC-alpha	62.4
259	PKCb1	89.9
260	PKCb2	39.6
261	PRKCD	95.2
262	PRKCE	94.1
263	PRKCH	87.9
264	PRKCG	49.6
265	PRKCI	86.1
266	PRKD1	70.3
267	PRKD3	54.3
268	PRKCQ	79.8
269	PKCzeta	90.2
270	PRKD2	72.3
271	PRKG1	107.7
272	PRKG1b	97.6
273	PRKG2	99.5
274	PKN1	85.4
275	PKN2	85.8
276	PKN3	81.6
277	PLK1	68.3
278	PLK2	106.8
279	PLK3	94.4
280	PLK4	83.4
281	PRKX	79.2
282	PYK2	47.5
283	RAF1	68.6
284	RET	79.4
285	RIPK2	66.1
286	RIPK4	97.8
287	RIPK5	67.8
288	ROCK1	97.2

No	Kinase	% remaining
289	ROCK2	92.3
290	MST1R	86.8
291	ROS1	27.8
292	RSK1	59.1
293	RSK2	69.4
294	RSK3	58.1
295	RSK4	54.2
296	SBK1	94.5
297	SGK1	84.9
298	SGK2	84.7
299	SGK3	92.6
300	SIK	56.3
301	SIK2	35.9
302	QSK	30.2
303	SLK	69.5
304	SNARK	59
305	SNRK	93
306	SRMS	96.8
307	SRPK1	54.3
308	SRPK2	60.7
309	TSSK6	86.9
310	STK16	35.9
311	CIT	97.9
312	TSSK1B	38
313	YSK1	23.1
314	YANK2	57.6
315	YANK3	86.6
316	STK33	33.5
317	NDR1	28.3
318	NDR2	36.9
319	STK39	29.6
320	SYK	48.4
321	TAK1	98.2
322	TAOK1	28.1
323	TAOK2	23.8
324	TAOK3	23.1
325	TBK1	55
326	TEC	71.5
327	TESK1	93.1
328	TESK2	83.7
329	TGFBR2	94.5
330	TIE2	34.8
331	TLK1	67.8
332	TLK2	72.2
333	TNIK	69
334	TNK1	91.3
335	TRKA	28.7
336	TRKB	16.5

No	Kinase	% remaining
337	TRKC	54.9
338	TSSK2	85.3
339	TSSK3	91.5
340	TTBK1	107.7
341	TTBK2	97.3
342	TXK	34.9
343	LTK	73.6
344	TYK2	90.7
345	TYRO3	22.4
346	ULK1	70.3
347	ULK2	87.3
348	ULK3	91.8
349	VRK1	87.3
350	VRK2	96.6
351	WEE1	90.2
352	WNK1	82.5
353	WNK2	107.8
354	WNK3	74.5
355	YES	20.6
356	YSK4	96.8
357	ZAK	97.5
358	ZAP70	100.1
359	DAPK3	80.8

^1H and ^{13}C NMR spectra for key intermediates

16a

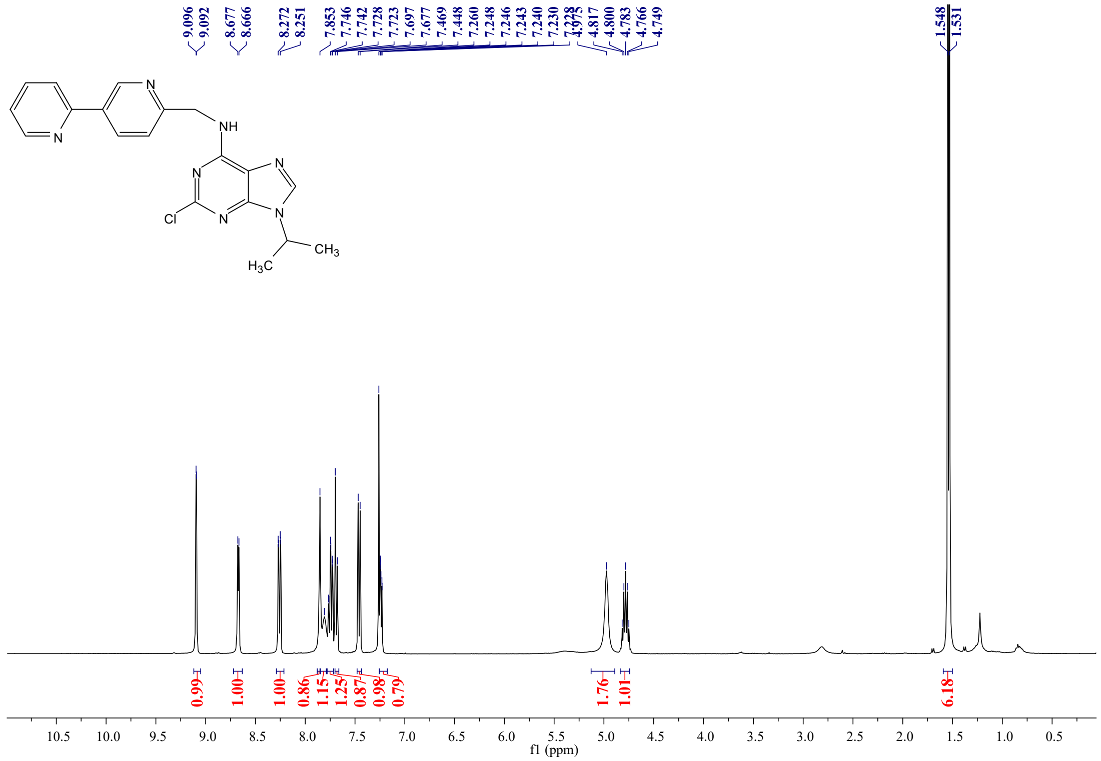

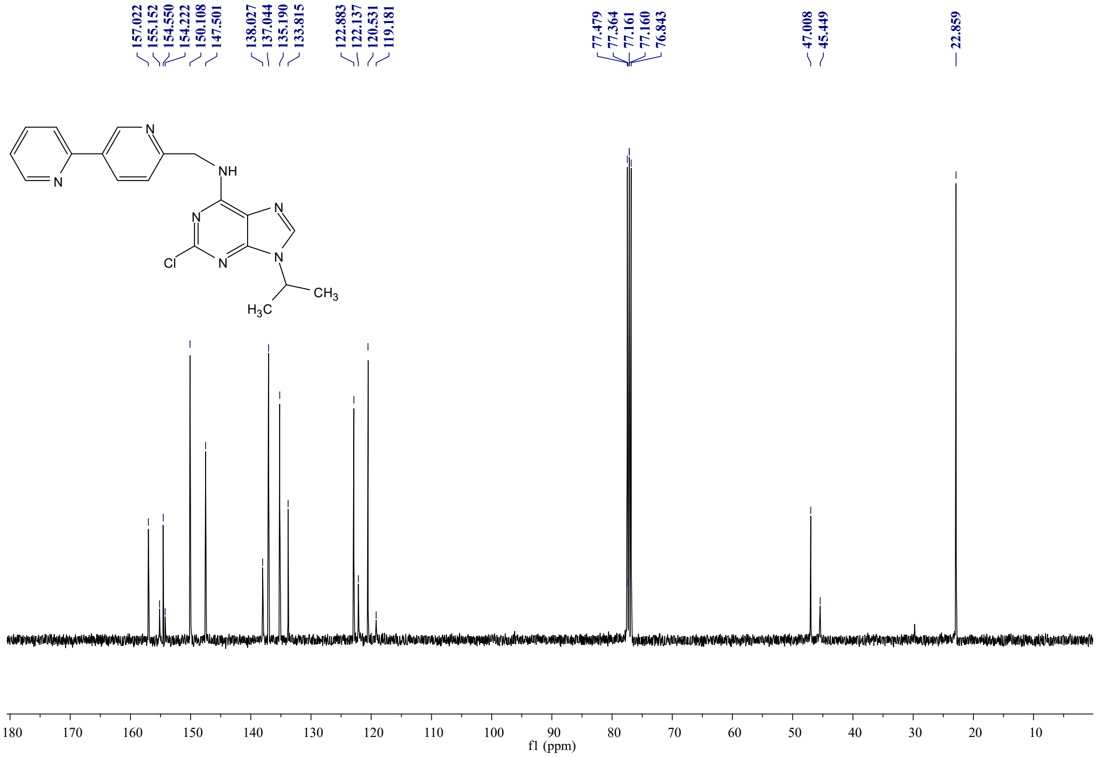

16b

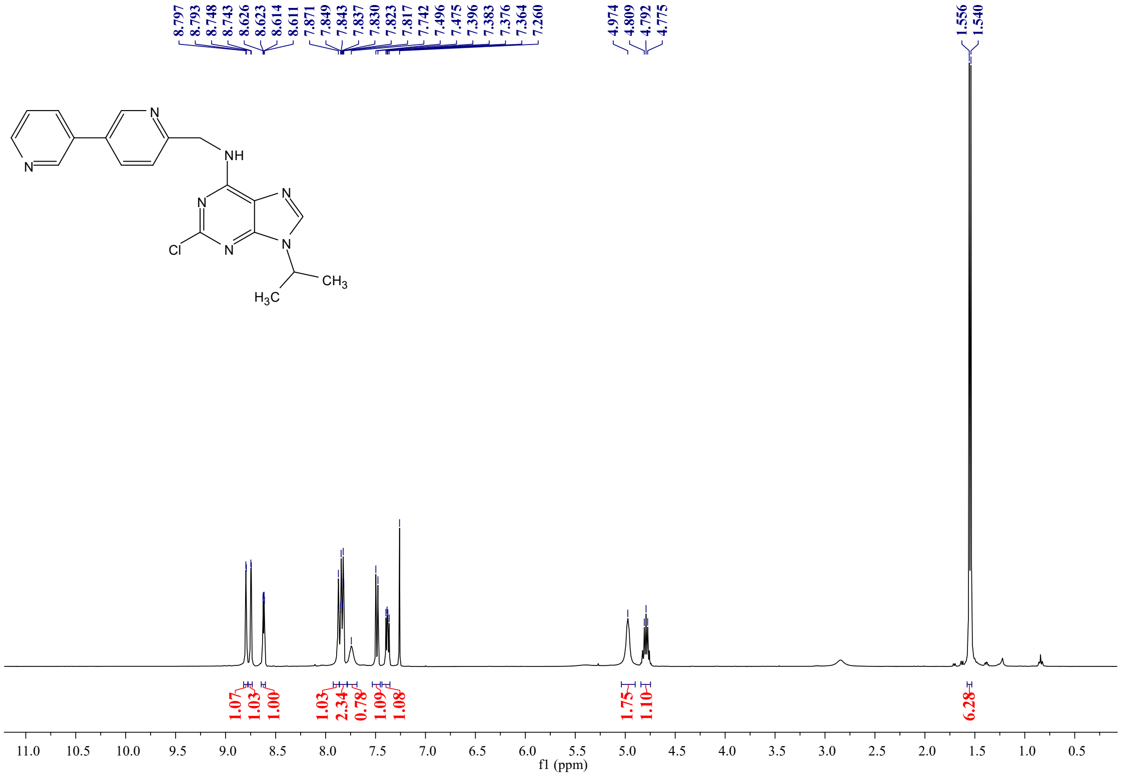
8.797
8.793
8.748
8.743
8.626
8.623
8.614
8.611
7.871
7.849
7.843
7.837
7.830
7.823
7.817
7.742
7.496
7.475
7.396
7.383
7.376
7.364
7.260
4.974
4.809
4.792
4.775
1.556
1.540
NH
N
N
N
N
N
N
Cl
CH3
H3C
1.07
1.03
1.00
1.03
2.34
0.78
1.09
1.08
1.75
1.10
6.28
11.0 10.5 10.0 9.5 9.0 8.5 8.0 7.5 7.0 6.5 6.0 5.5 5.0 4.5 4.0 3.5 3.0 2.5 2.0 1.5 1.0 0.5
f1 (ppm)

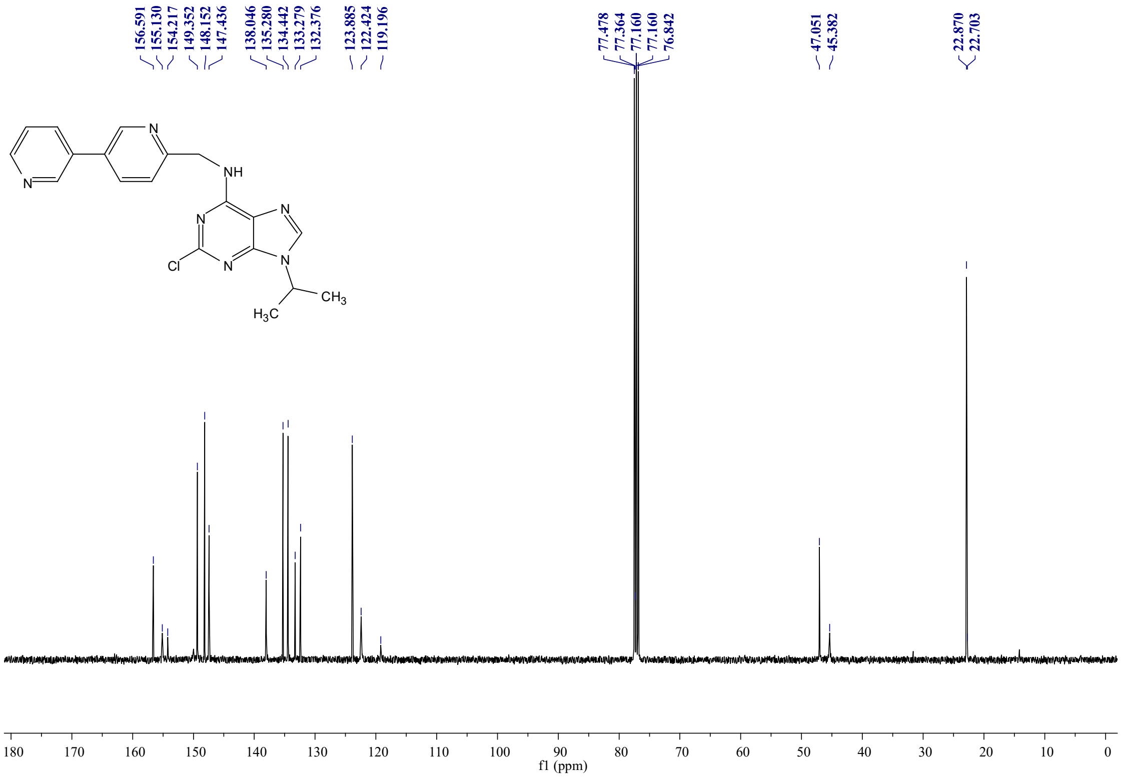
156.591
155.130
154.217
149.352
148.152
147.436
138.046
135.280
134.442
133.279
132.376
123.885
122.424
119.196
77.478
77.364
77.160
77.160
76.842
47.051
45.382
22.870
22.703
NH
N
N
N
N
N
N
Cl
CH3
H3C
180 170 160 150 140 130 120 110 100 90 80 70 60 50 40 30 20 10 0
f1 (ppm)

16c

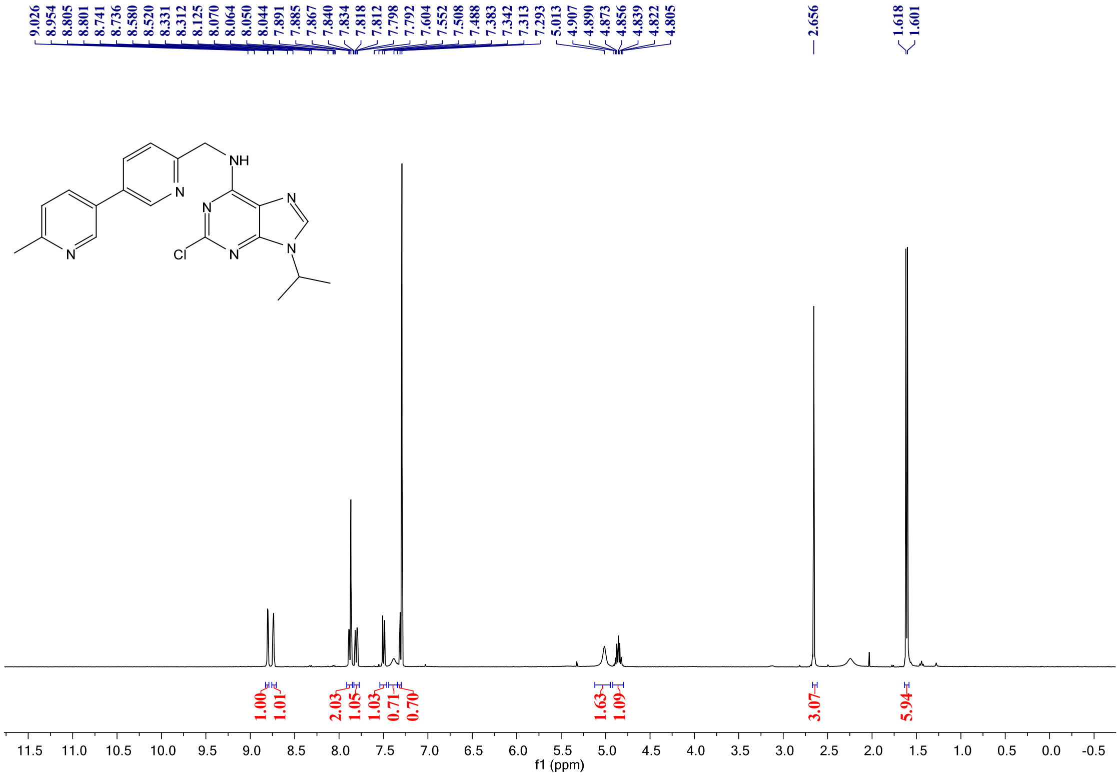
9.026 8.954 8.805 8.801 8.741 8.736 8.580 8.520 8.331 8.312 8.125 8.070 8.064 8.050 8.044 7.891 7.885 7.867 7.840 7.834 7.818 7.812 7.798 7.792 7.604 7.552 7.508 7.488 7.383 7.342 7.313 7.293 5.013 4.907 4.890 4.873 4.856 4.839 4.822 4.805
2.656
1.618 1.601
NH
N
N
N
N
N
Cl
N
1.00 1.01 2.03 1.05 1.03 0.71 0.70 1.63 1.09 3.07 5.94
11.5 11.0 10.5 10.0 9.5 9.0 8.5 8.0 7.5 7.0 6.5 6.0 5.5 5.0 4.5 4.0 3.5 3.0 2.5 2.0 1.5 1.0 0.5 0.0 -0.5
f1 (ppm)

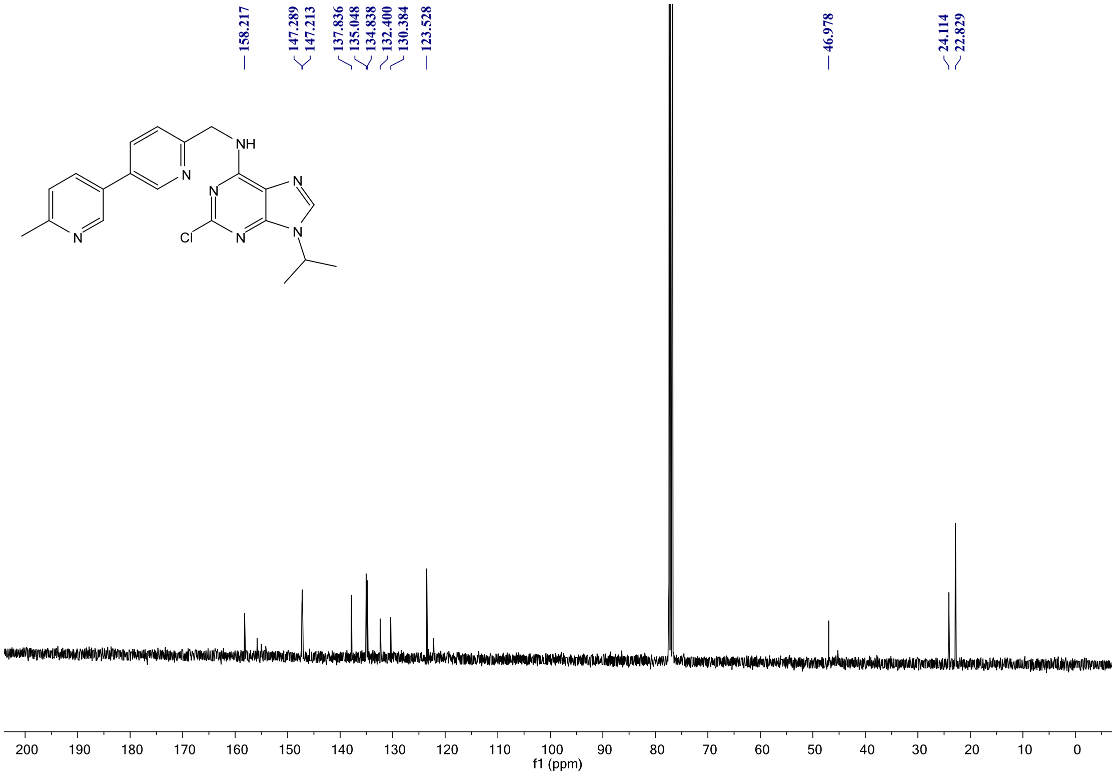
158.217
147.289 147.213
137.836 135.048 134.838 132.400 130.384
123.528
46.978
24.114 22.829
NH
N
N
N
N
N
Cl
N
200 190 180 170 160 150 140 130 120 110 100 90 80 70 60 50 40 30 20 10 0
f1 (ppm)

16d

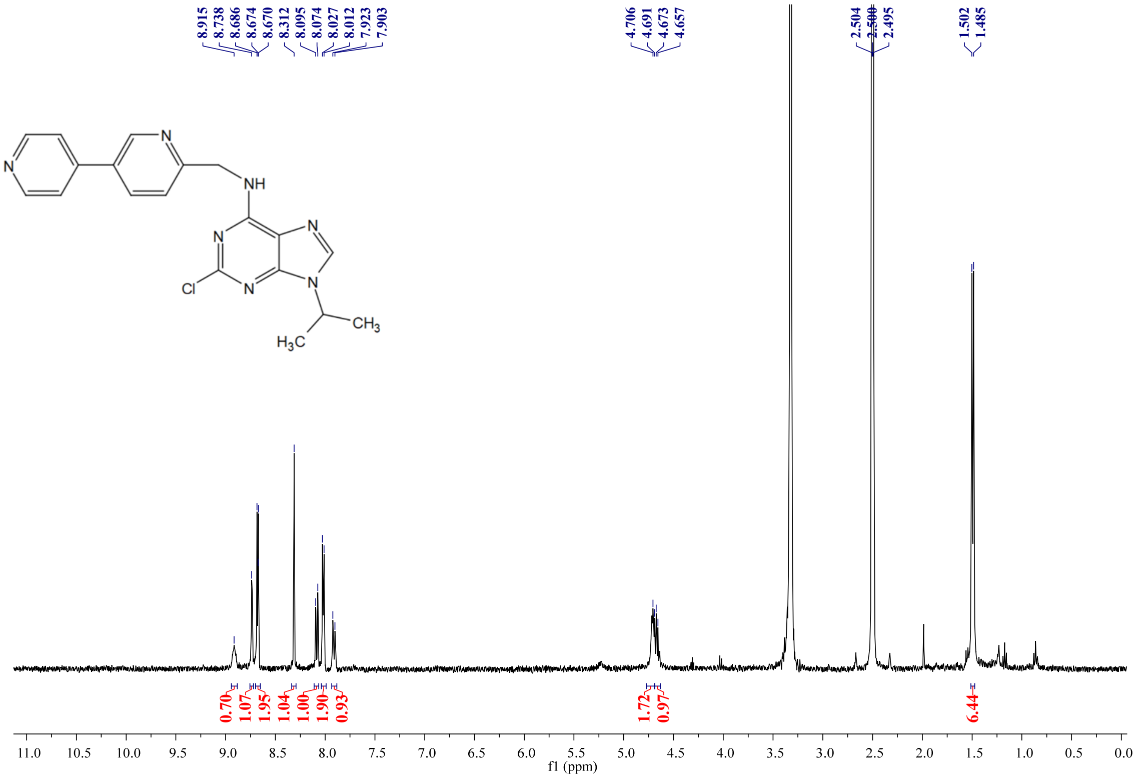

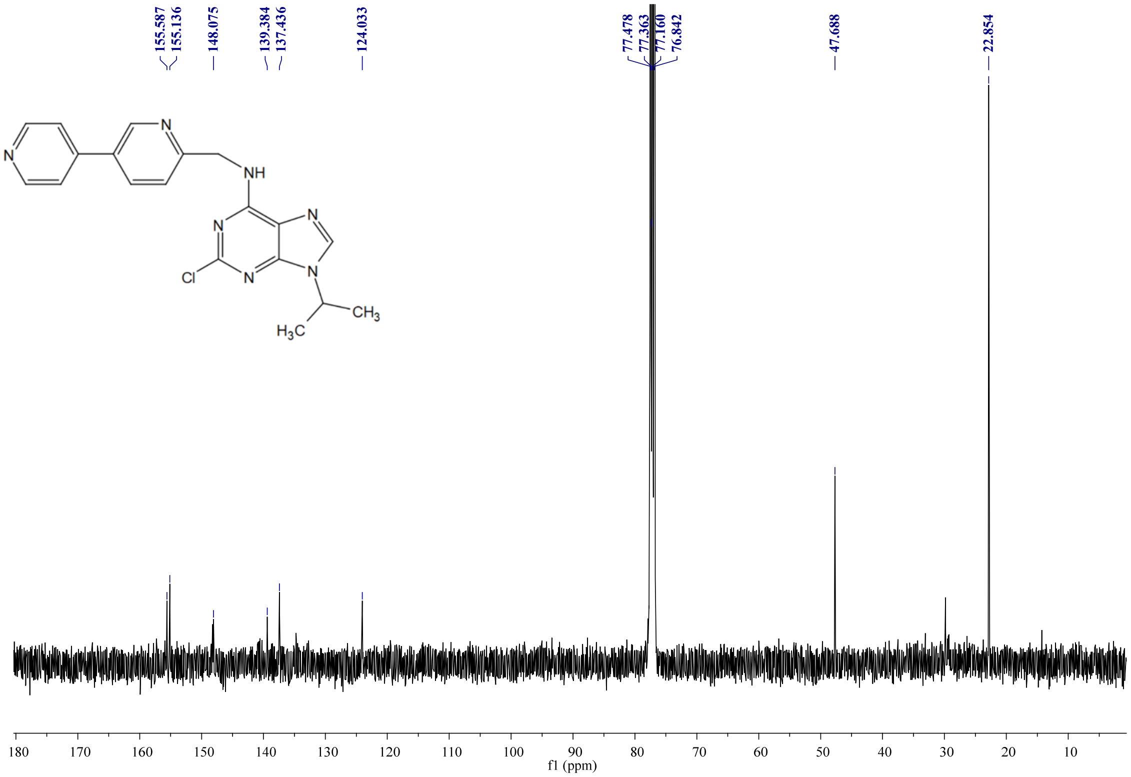

16e

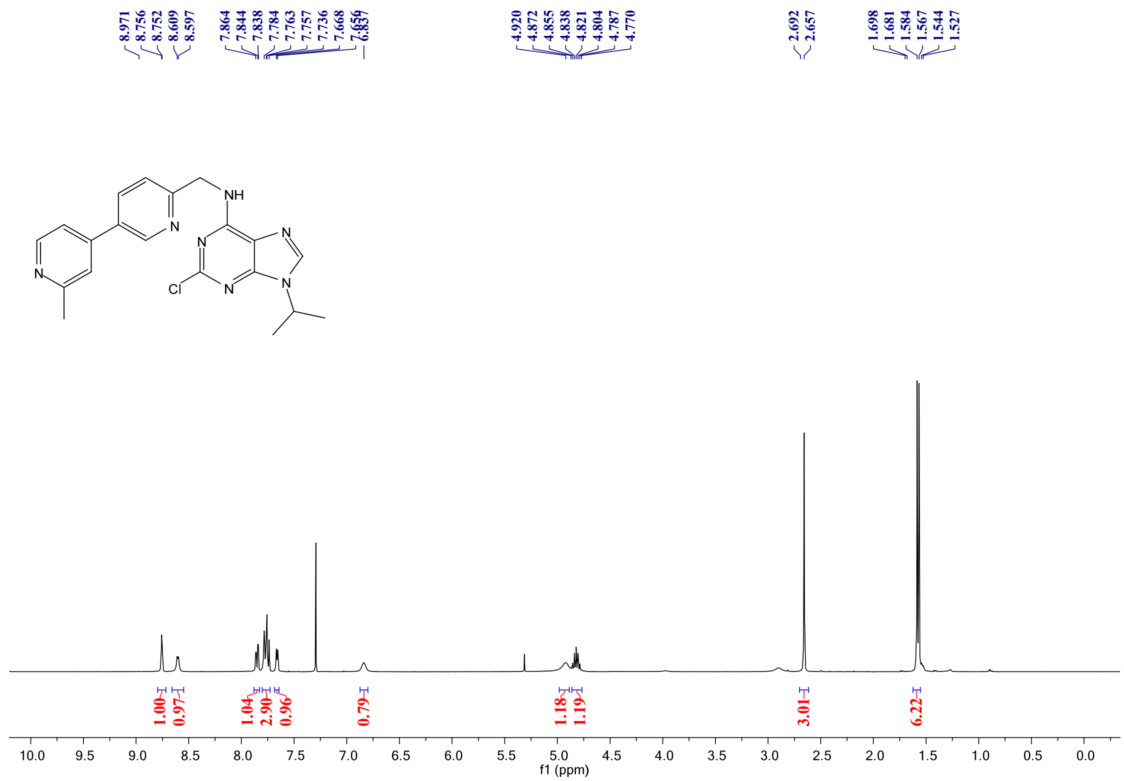

8.971
8.756
8.752
8.609
8.597
7.864
7.844
7.838
7.784
7.763
7.757
7.736
7.668
7.656
6.837
4.920
4.872
4.855
4.838
4.821
4.804
4.787
4.770
2.692
2.657
1.698
1.681
1.584
1.567
1.544
1.527
NH
N
N
N
N
N
N
Cl
1.00
0.97
1.04
2.90
0.96
0.79
1.18
1.19
3.01
6.22
f1 (ppm)

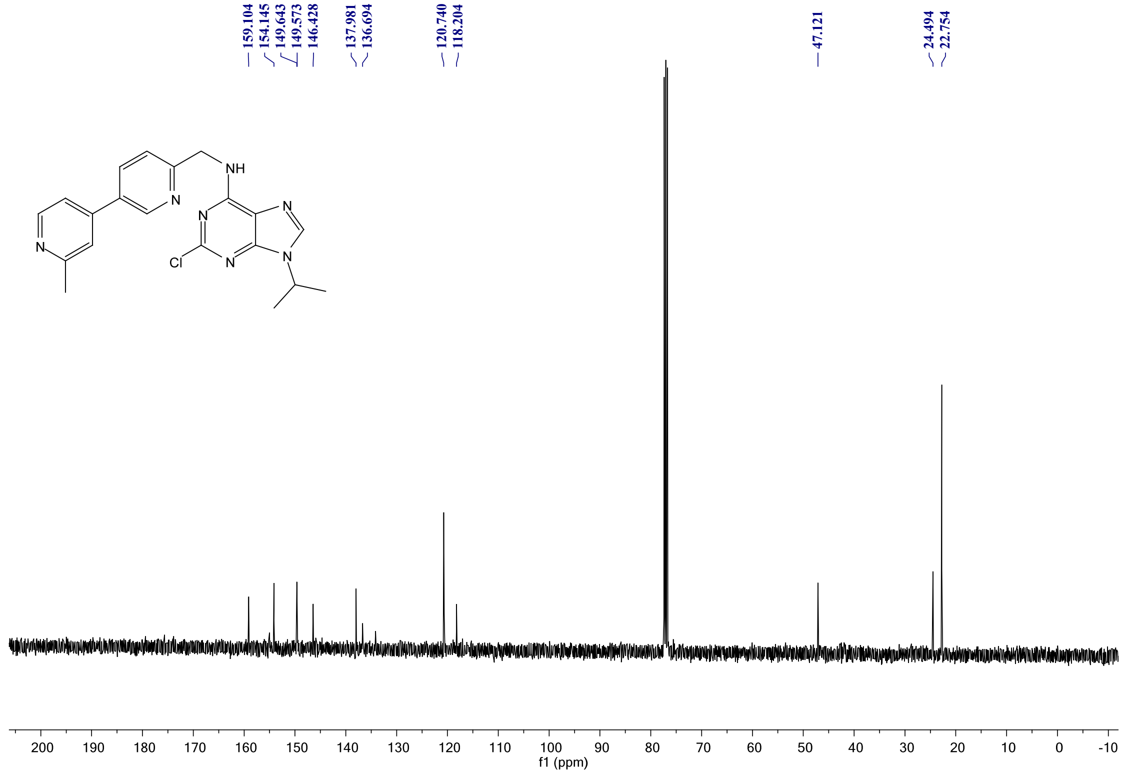

159.104
154.145
149.643
149.573
146.428
137.981
136.694
120.740
118.204
47.121
24.494
22.754
NH
N
N
N
N
N
N
Cl
f1 (ppm)

16f

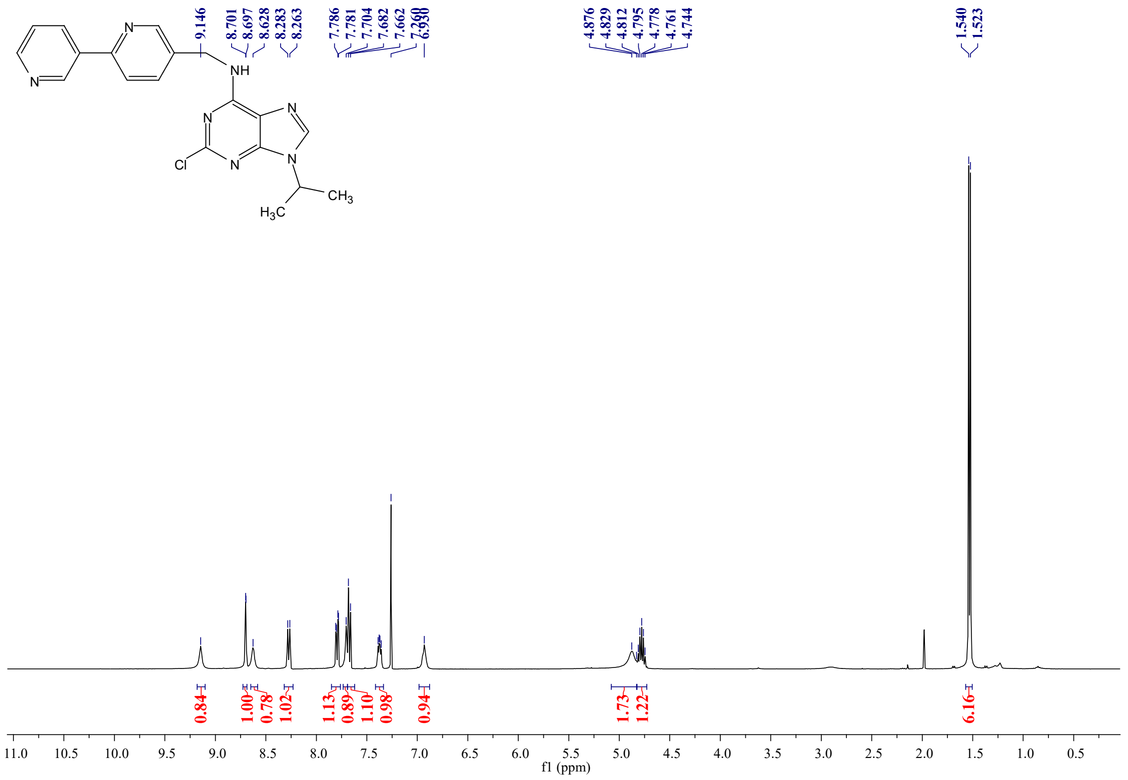
9.146
8.701
8.697
8.628
8.283
8.263
7.786
7.781
7.704
7.682
7.662
7.260
6.930
4.876
4.829
4.812
4.795
4.778
4.761
4.744
1.540
1.523
NH
N
Cl
CH3
H3C
0.84
1.00
0.78
1.02
1.13
0.89
1.10
0.98
0.94
1.73
1.22
6.16
11.0 10.5 10.0 9.5 9.0 8.5 8.0 7.5 7.0 6.5 6.0 5.5 5.0 4.5 4.0 3.5 3.0 2.5 2.0 1.5 1.0 0.5
f1 (ppm)

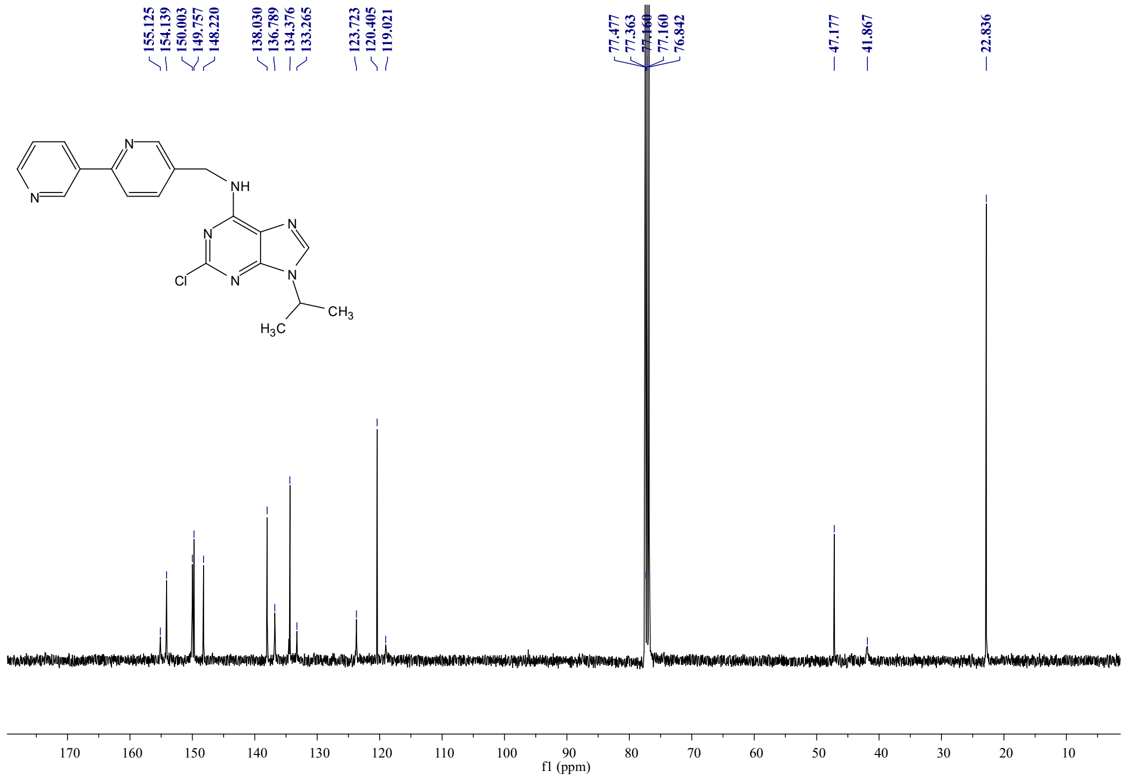
155.125
154.139
150.003
149.757
148.220
138.030
136.789
134.376
133.265
123.723
120.405
119.021
77.477
77.363
77.160
77.160
76.842
47.177
41.867
22.836
NH
N
Cl
CH3
H3C
170 160 150 140 130 120 110 100 90 80 70 60 50 40 30 20 10
f1 (ppm)

16g

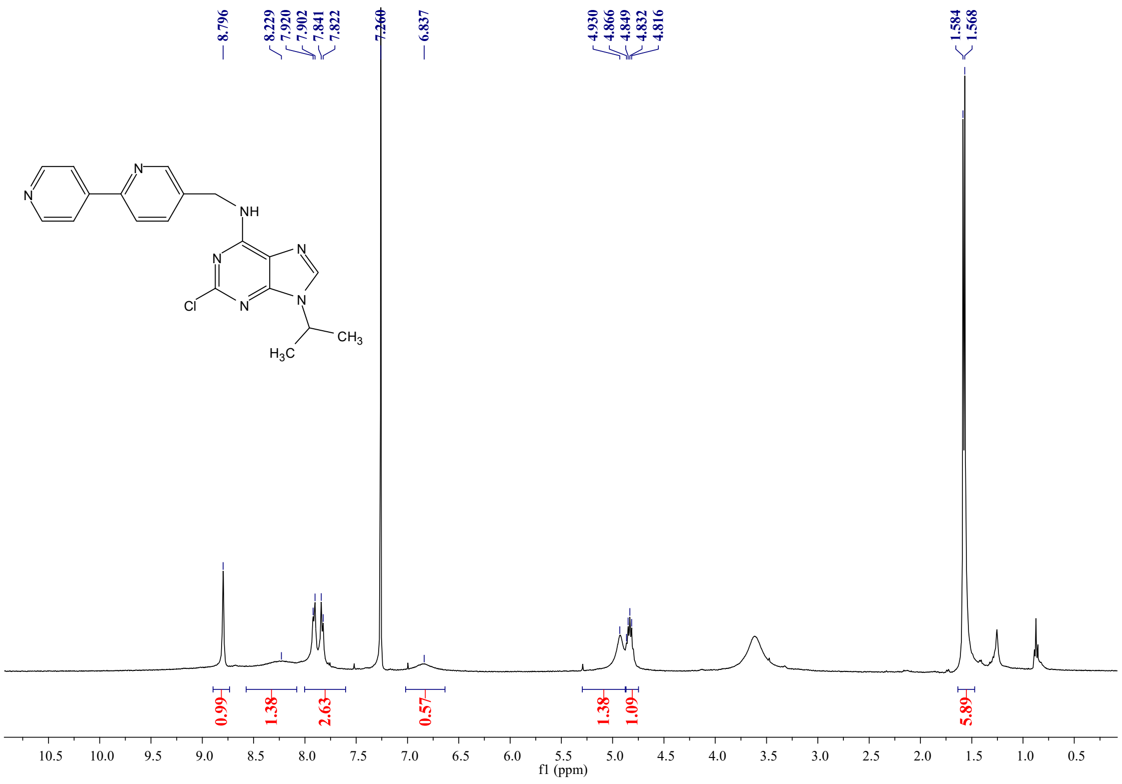

8.796
8.229
7.920
7.902
7.841
7.822
7.260
6.837
4.930
4.866
4.849
4.832
4.816
1.584
1.568
0.99
1.38
2.63
0.57
1.38
1.09
5.89
f1 (ppm)

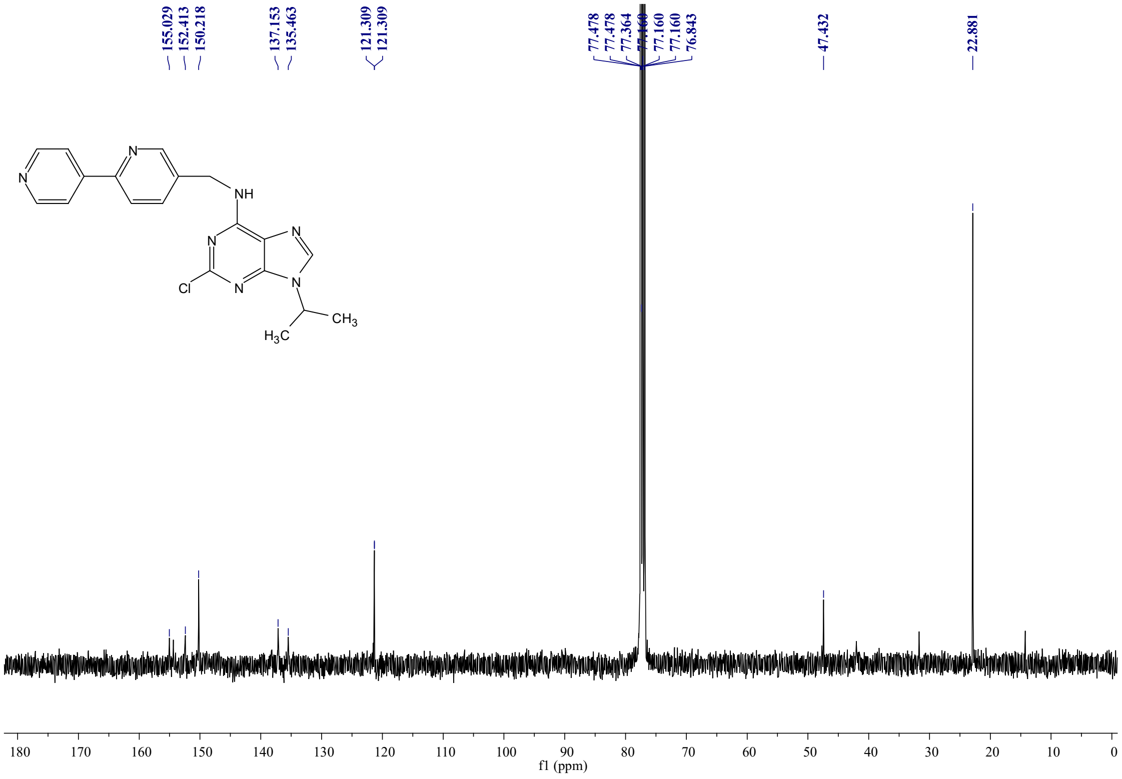

155.029
152.413
150.218
137.153
135.463
121.309
121.309
77.478
77.478
77.364
77.160
77.160
76.843
47.432
22.881
f1 (ppm)

23

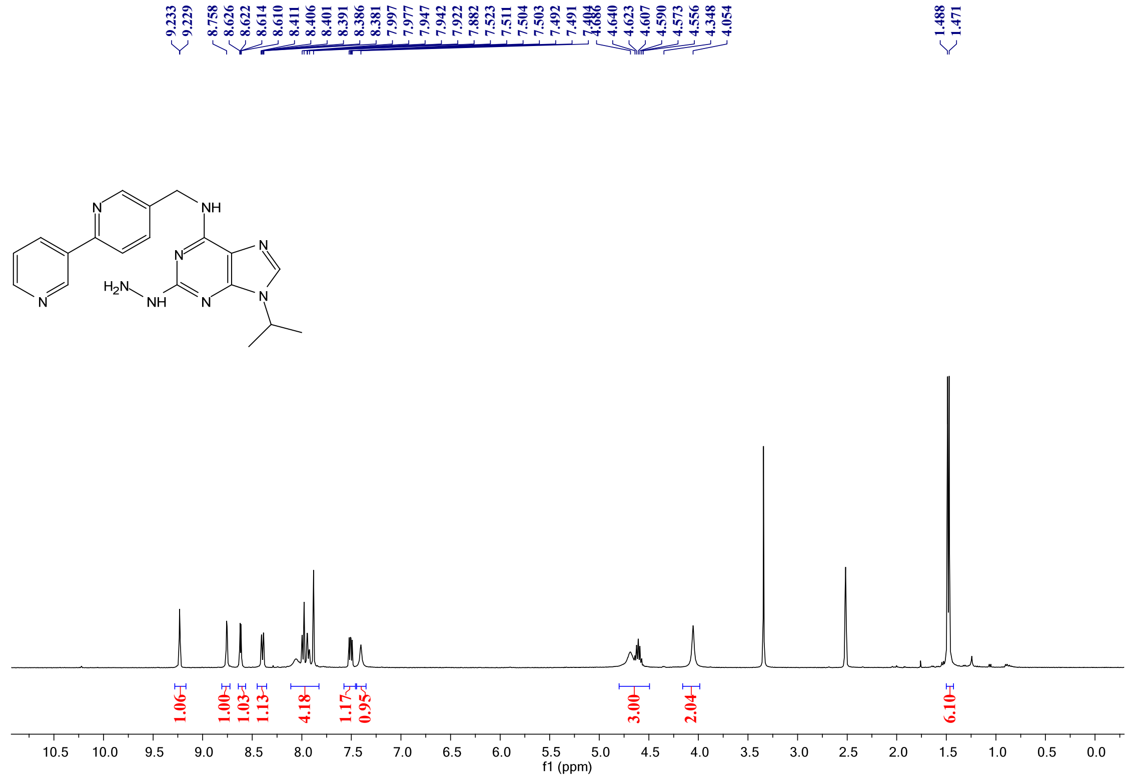
9.233
9.229
8.758
8.626
8.622
8.614
8.610
8.411
8.406
8.401
8.391
8.386
8.381
7.997
7.977
7.947
7.942
7.922
7.882
7.523
7.511
7.504
7.503
7.492
7.491
4.640
4.623
4.607
4.590
4.573
4.556
4.348
4.054
1.488
1.471
N
NH
N
N
N
N
H_2N
NH
N
1.06
1.00
1.03
1.13
4.18
1.17
0.95
3.00
2.04
6.10
10.5 10.0 9.5 9.0 8.5 8.0 7.5 7.0 6.5 6.0 5.5 5.0 4.5 4.0 3.5 3.0 2.5 2.0 1.5 1.0 0.5 0.0
f1 (ppm)

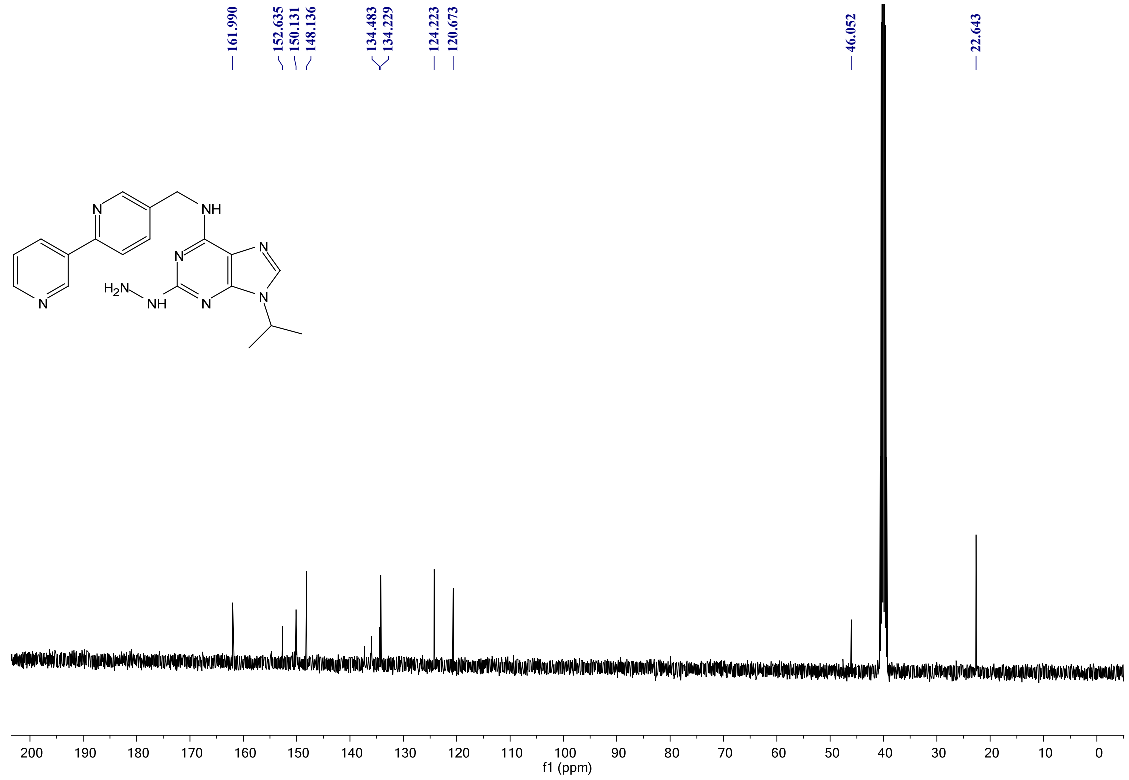
161.990
152.635
150.131
148.136
134.483
134.229
124.223
120.673
46.052
22.643
N
NH
N
N
N
N
H_2N
NH
N
200 190 180 170 160 150 140 130 120 110 100 90 80 70 60 50 40 30 20 10 0
f1 (ppm)

25

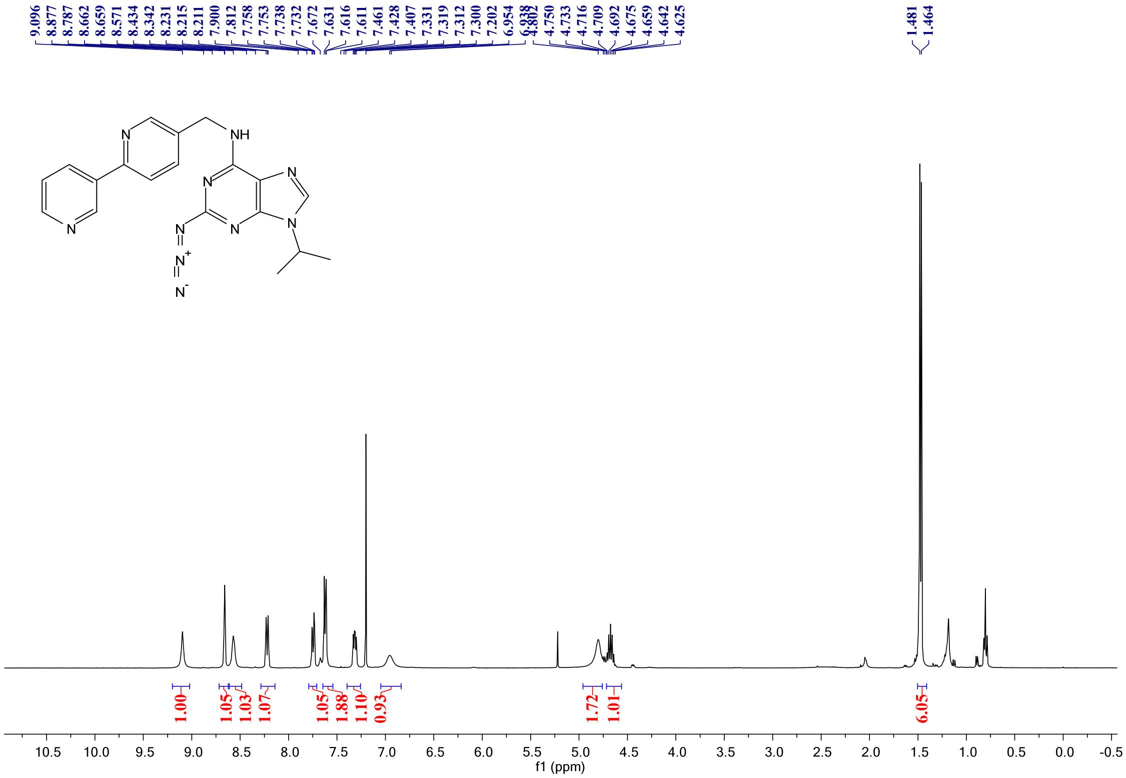
9.096 8.877 8.787 8.662 8.659 8.571 8.434 8.342 8.231 8.215 8.211 7.900 7.812 7.758 7.753 7.738 7.732 7.672 7.631 7.616 7.611 7.461 7.428 7.407 7.331 7.319 7.312 7.300 7.202 6.954 6.938 4.802 4.750 4.733 4.716 4.709 4.692 4.675 4.659 4.642 4.625
1.481 1.464
NH
N
N
N
N
N
N
N
N+
N-
1.00 1.05 1.03 1.07 1.05 1.88 1.10 0.93 1.72 1.01 6.05
f1 (ppm)

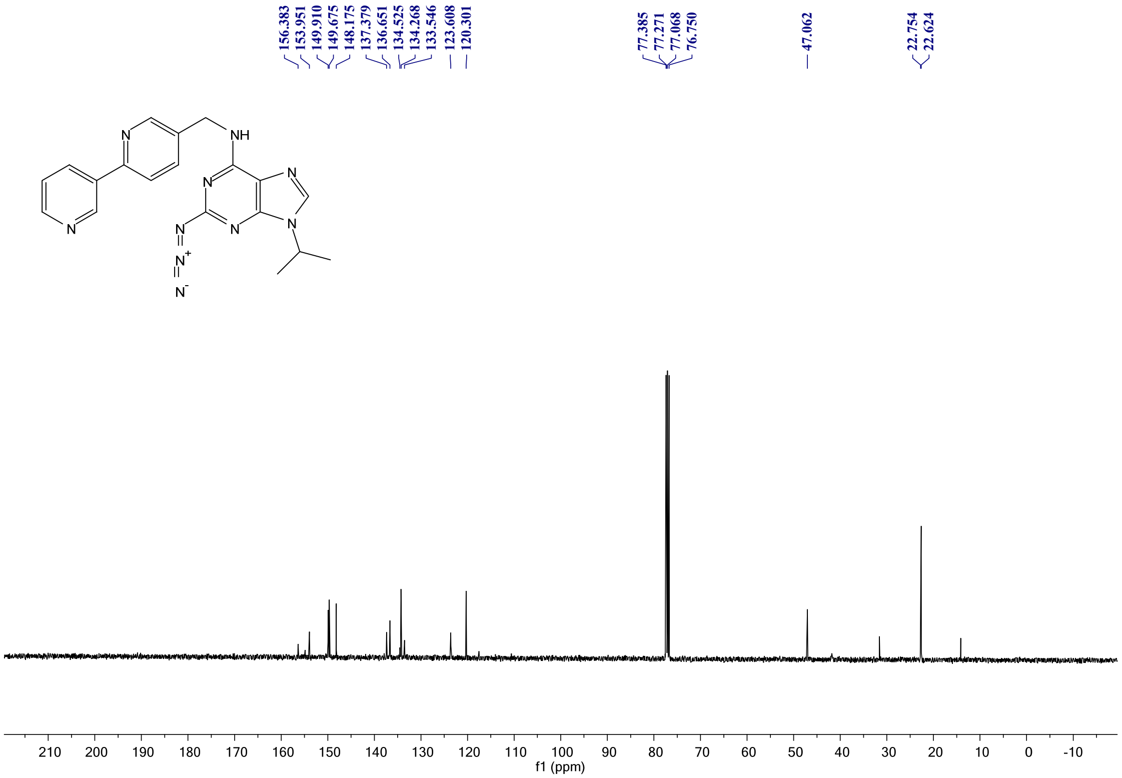
156.383 153.951 149.910 149.675 148.175 137.379 136.651 134.525 134.268 133.546 123.608 120.301
77.385 77.271 77.068 76.750
47.062
22.754 22.624
NH
N
N
N
N
N
N
N
N+
N-
f1 (ppm)

27a

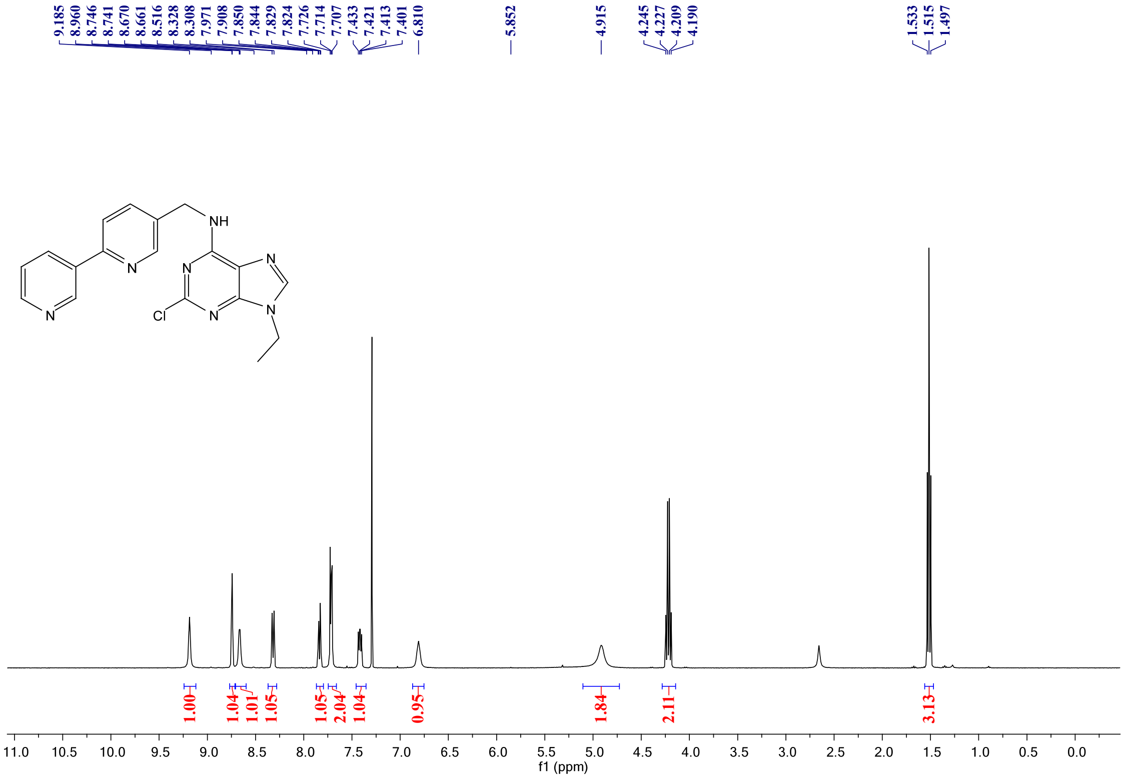
9.185
8.960
8.746
8.741
8.670
8.661
8.516
8.328
8.308
7.971
7.908
7.850
7.844
7.829
7.824
7.726
7.714
7.707
7.433
7.421
7.413
7.401
6.810
5.852
4.915
4.245
4.227
4.209
4.190
1.533
1.515
1.497
NH
N
N
N
N
N
Cl
N
1.00
1.04
1.01
1.05
1.05
2.04
1.04
0.95
1.84
2.11
3.13
f1 (ppm)

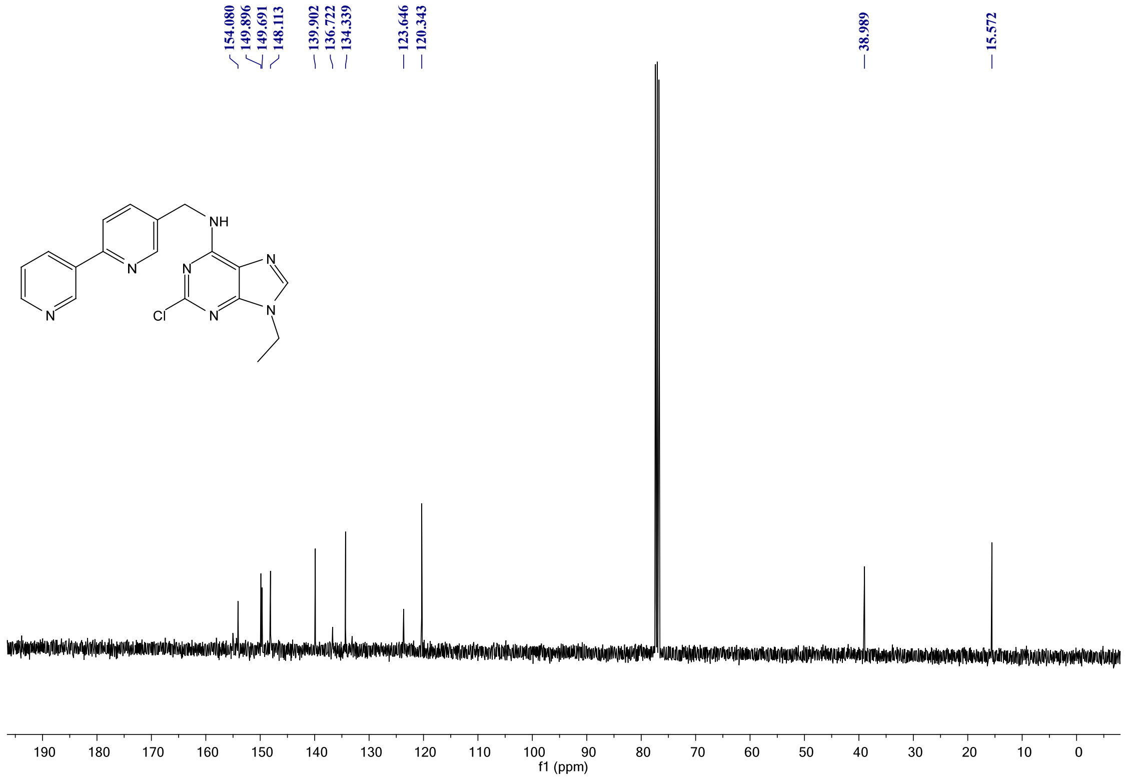
154.080
149.896
149.691
148.113
139.902
136.722
134.339
123.646
120.343
38.989
15.572
NH
N
N
N
N
N
Cl
N
f1 (ppm)

27b

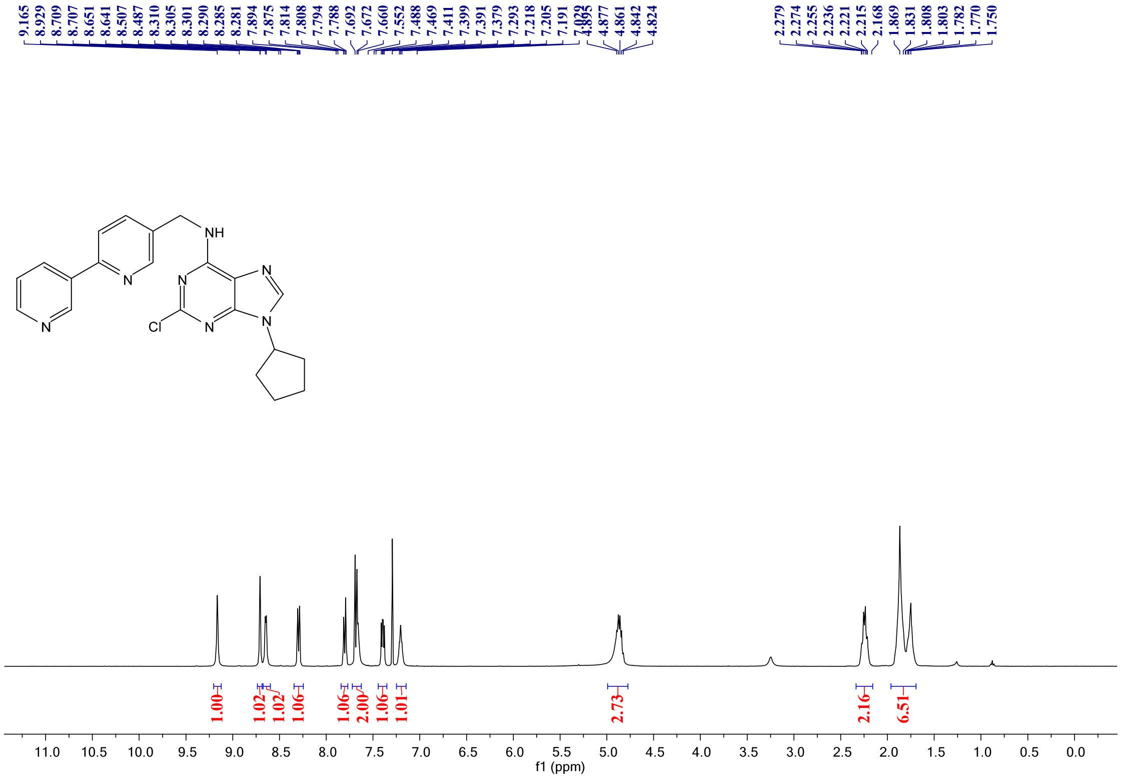
9.165
8.929
8.709
8.707
8.651
8.641
8.507
8.487
8.310
8.305
8.301
8.290
8.285
8.281
7.894
7.875
7.814
7.808
7.794
7.788
7.692
7.672
7.660
7.552
7.488
7.469
7.411
7.399
7.391
7.379
7.293
7.218
7.205
7.191
4.877
4.861
4.842
4.824
2.279
2.274
2.255
2.236
2.221
2.215
2.168
1.869
1.831
1.808
1.803
1.782
1.770
1.750
NH
N
Cl
1.00
1.02
1.02
1.06
1.06
2.00
1.06
1.01
2.73
2.16
6.51
f1 (ppm)

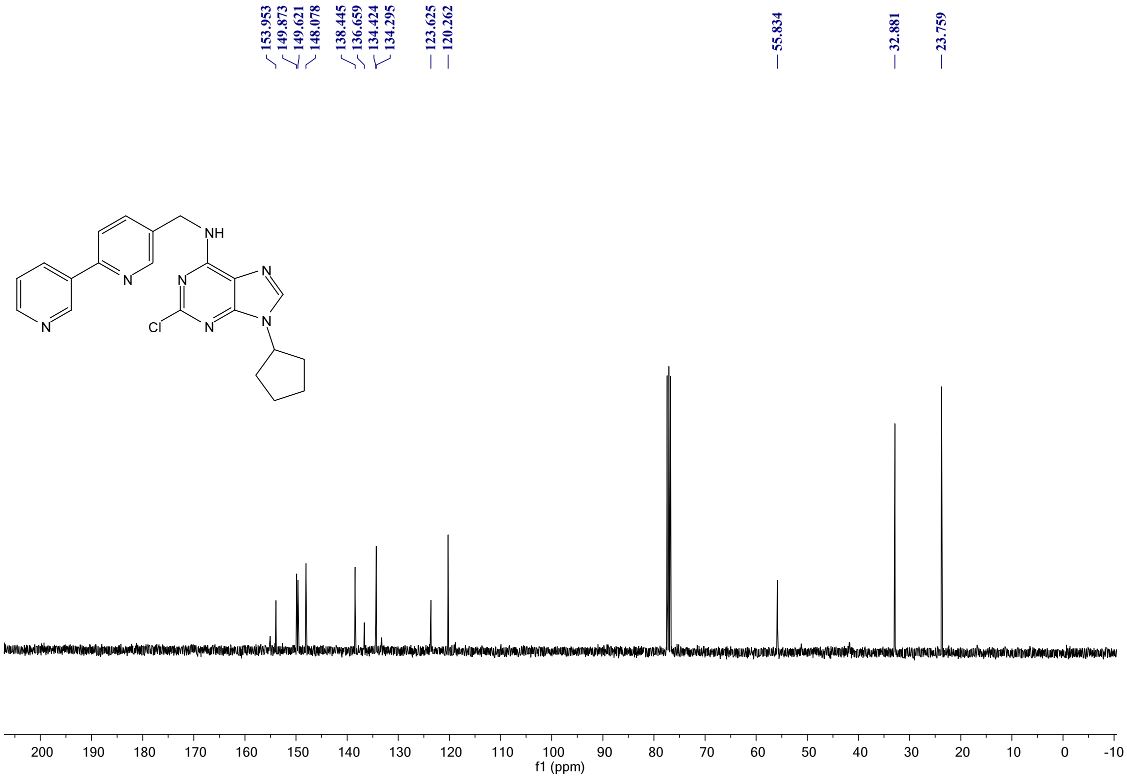
153.953
149.873
149.621
148.078
138.445
136.659
134.424
134.295
123.625
120.262
55.834
32.881
23.759
NH
N
Cl
f1 (ppm)

27c

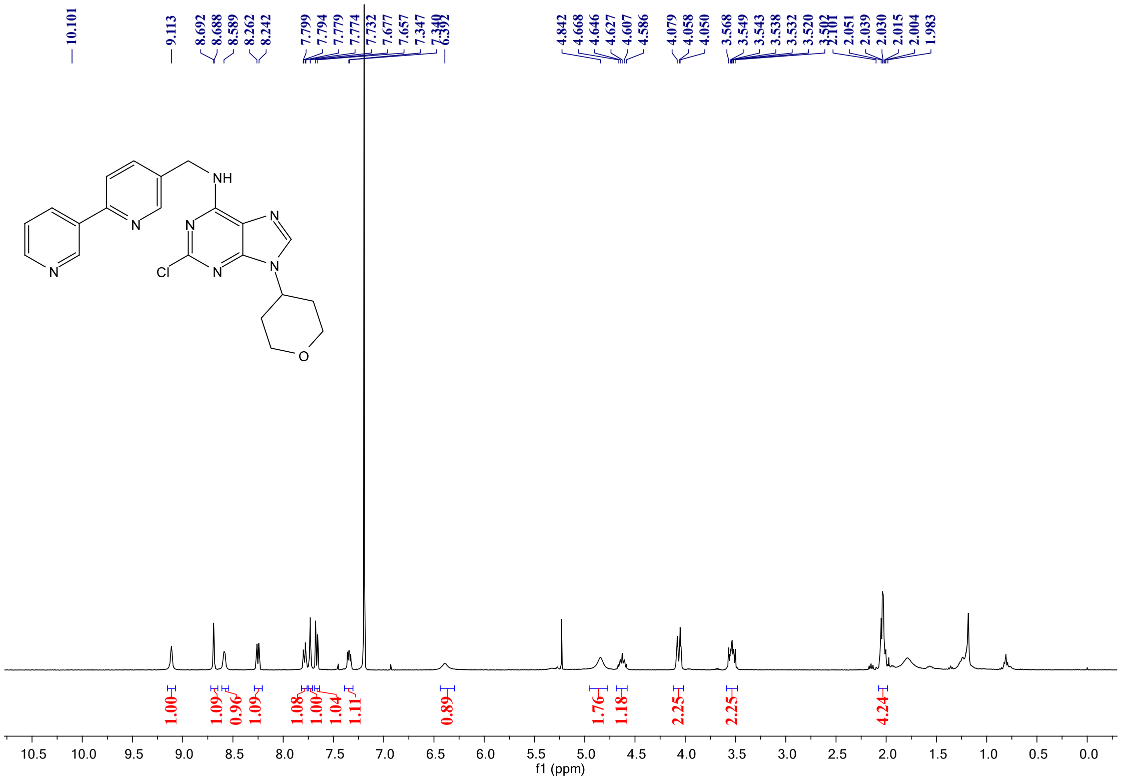
10.101
9.113
8.692
8.688
8.589
8.262
8.242
7.799
7.794
7.779
7.774
7.732
7.677
7.657
7.347
4.842
4.668
4.646
4.627
4.607
4.586
4.079
4.058
4.050
3.568
3.549
3.543
3.538
3.532
3.520
2.051
2.039
2.030
2.015
2.004
1.983
NH
N
Cl
O
1.00
1.09
0.96
1.09
1.08
1.00
1.04
1.11
0.89
1.76
1.18
2.25
2.25
4.24
f1 (ppm)

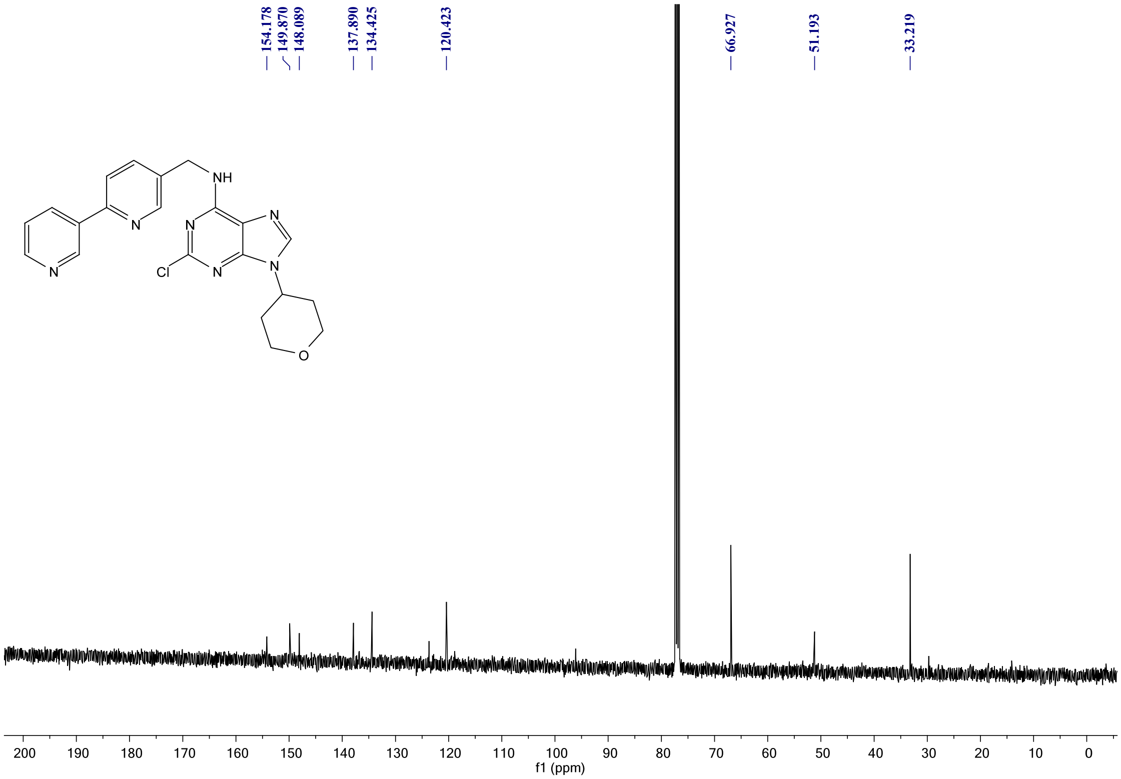
154.178
149.870
148.089
137.890
134.425
120.423
66.927
51.193
33.219
NH
N
Cl
O
f1 (ppm)

29a

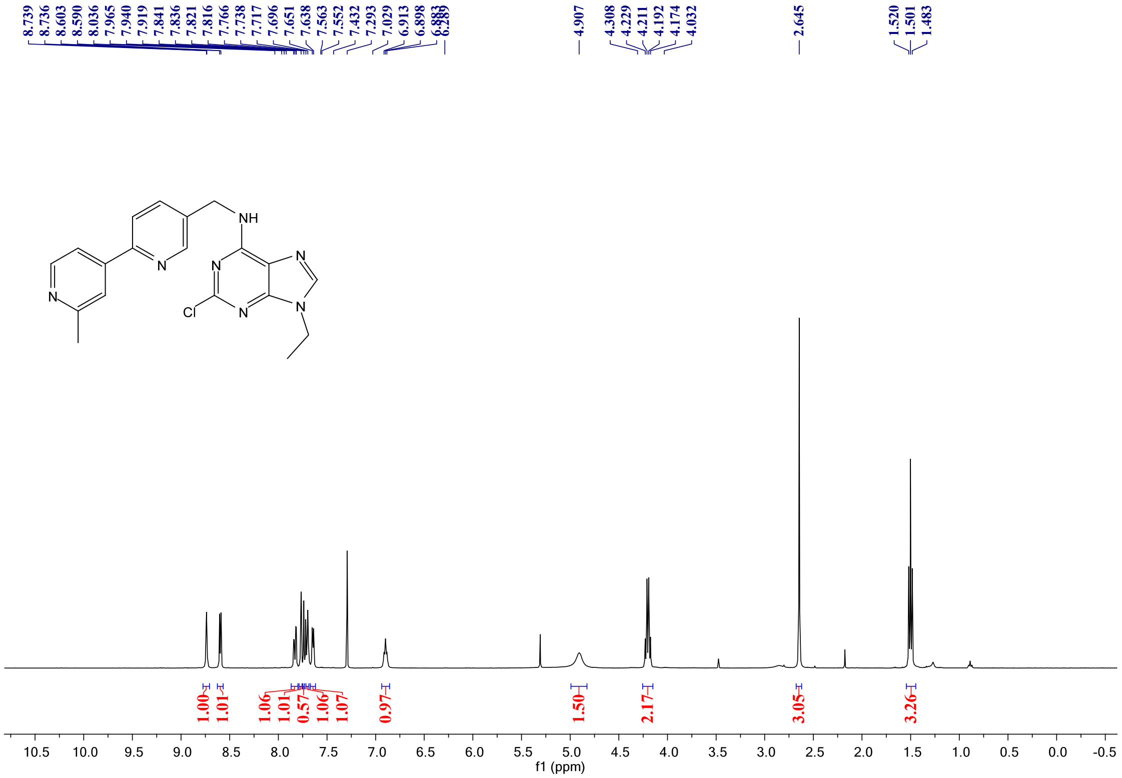
8.739
8.736
8.603
8.590
8.036
7.965
7.940
7.919
7.841
7.836
7.821
7.816
7.766
7.738
7.717
7.696
7.651
7.638
7.563
7.552
7.432
7.293
7.029
6.913
6.898
4.907
4.308
4.229
4.211
4.192
4.174
4.032
2.645
1.520
1.501
1.483
NH
N
N
N
N
Cl
N
N
N
1.00
1.01
1.06
1.01
0.57
1.06
1.07
0.97
1.50
2.17
3.05
3.26
f1 (ppm)

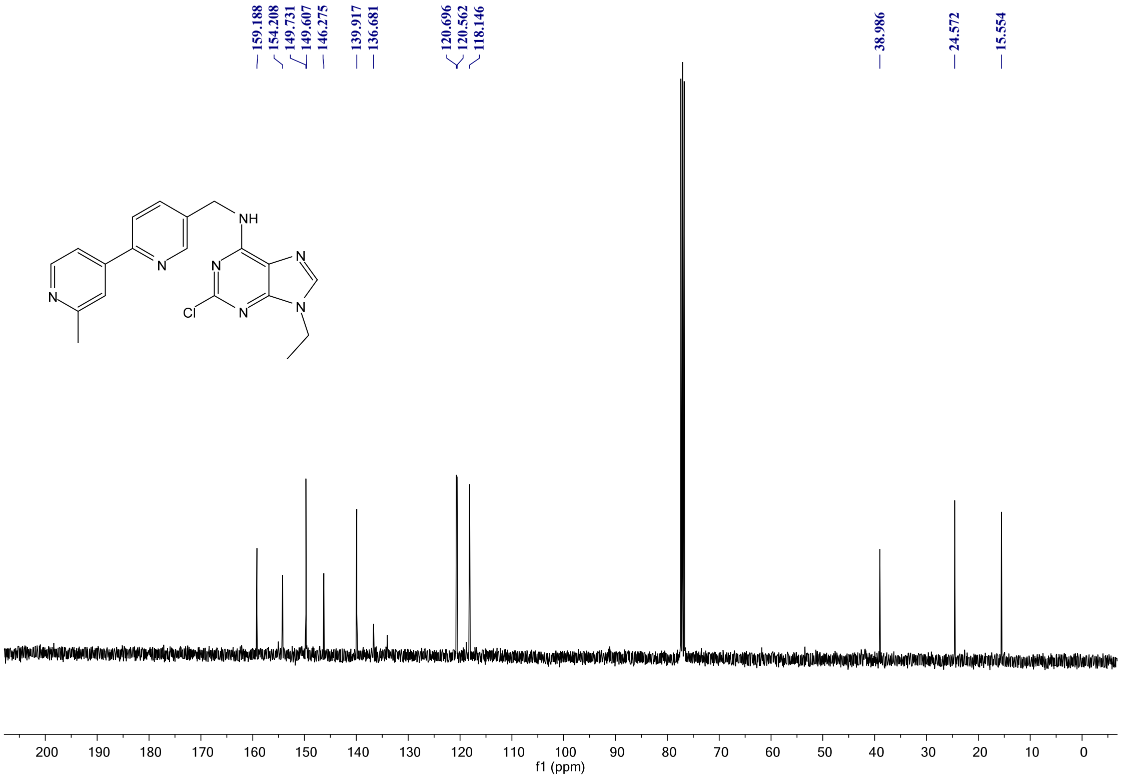
159.188
154.208
149.731
149.607
146.275
139.917
136.681
120.696
120.562
118.146
38.986
24.572
15.554
NH
N
N
N
N
Cl
N
N
N
f1 (ppm)

29b

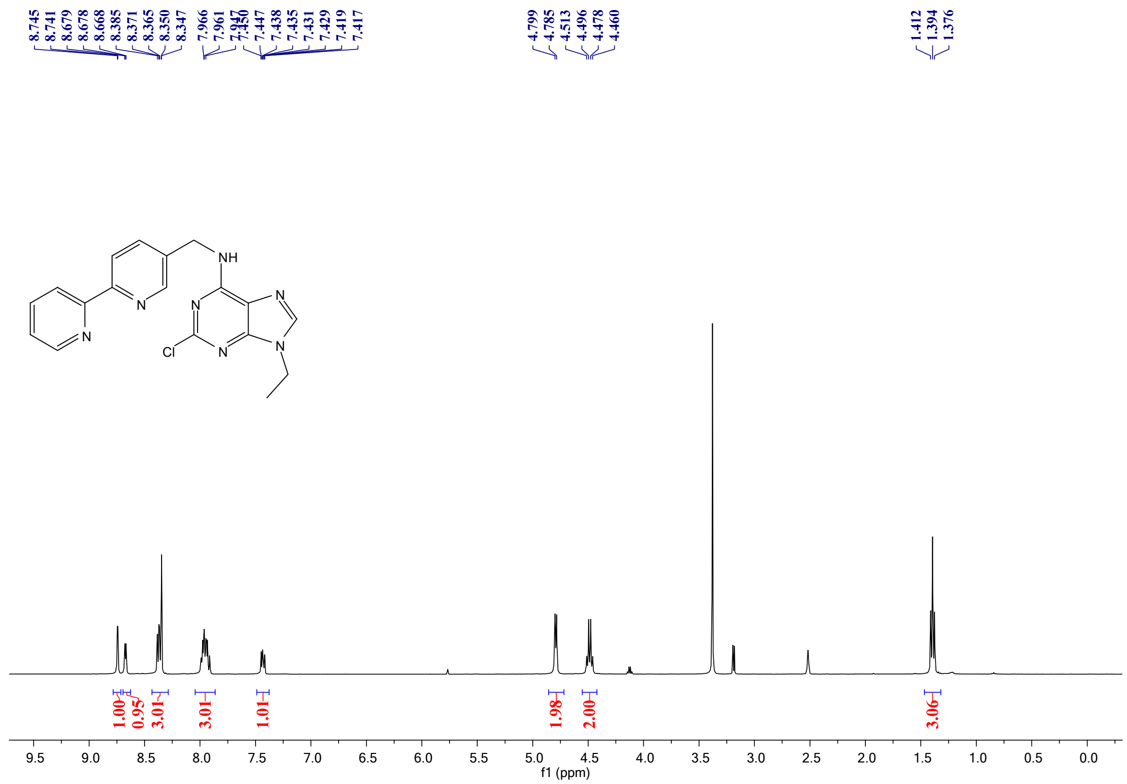
8.745
8.741
8.679
8.678
8.668
8.385
8.371
8.365
8.350
8.347
7.966
7.961
7.947
7.450
7.447
7.438
7.435
7.431
7.429
7.419
7.417
4.799
4.785
4.513
4.496
4.478
4.460
1.412
1.394
1.376
NH
N
N
N
N
N
N
Cl
1.00
0.95
3.01
3.01
1.01
1.98
2.00
3.06
9.5 9.0 8.5 8.0 7.5 7.0 6.5 6.0 5.5 5.0 4.5 4.0 3.5 3.0 2.5 2.0 1.5 1.0 0.5 0.0
f1 (ppm)

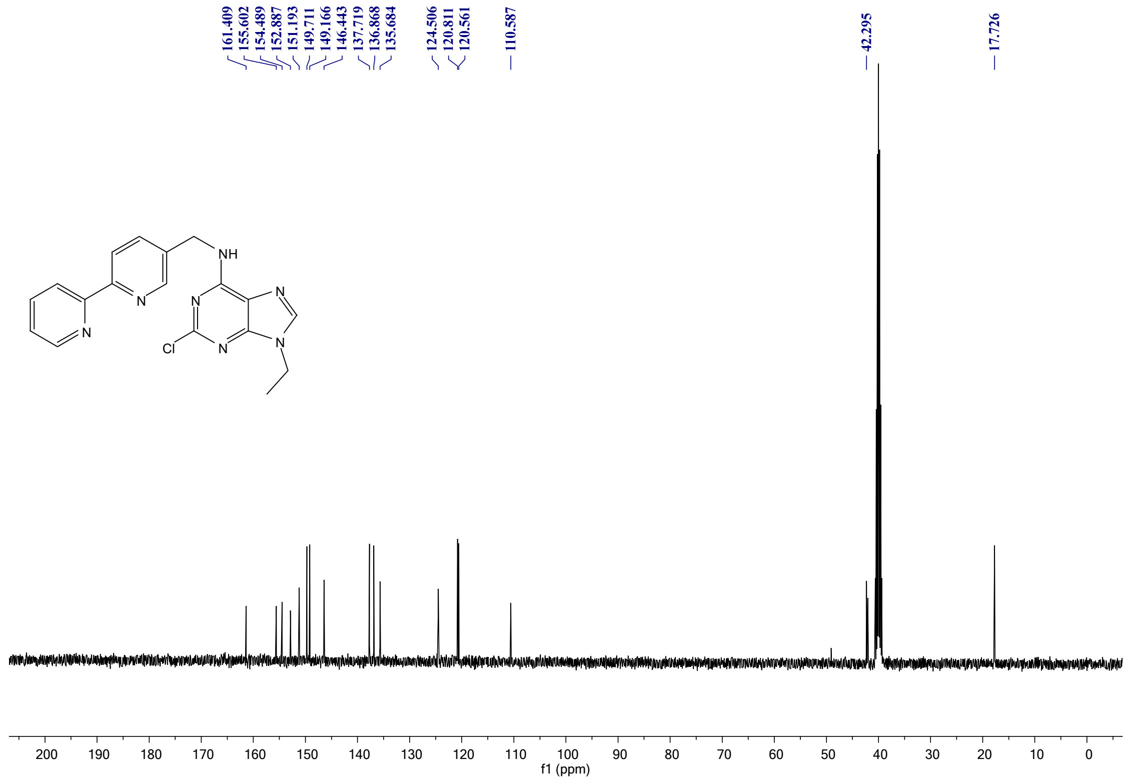
161.409
155.602
154.489
152.887
151.193
149.711
149.166
146.443
137.719
136.868
135.684
124.506
120.811
120.561
110.587
42.295
17.726
NH
N
N
N
N
N
N
Cl
200 190 180 170 160 150 140 130 120 110 100 90 80 70 60 50 40 30 20 10 0
f1 (ppm)

^{1}H and ^{13}C NMR spectra for final compounds

17a

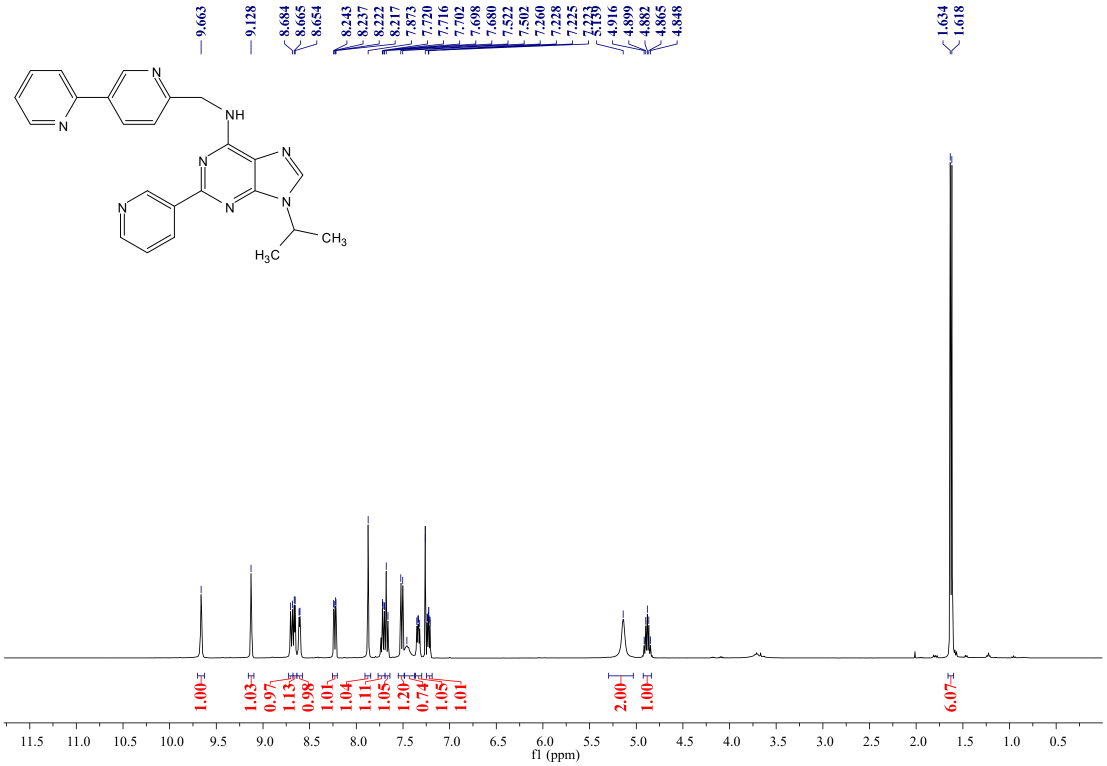

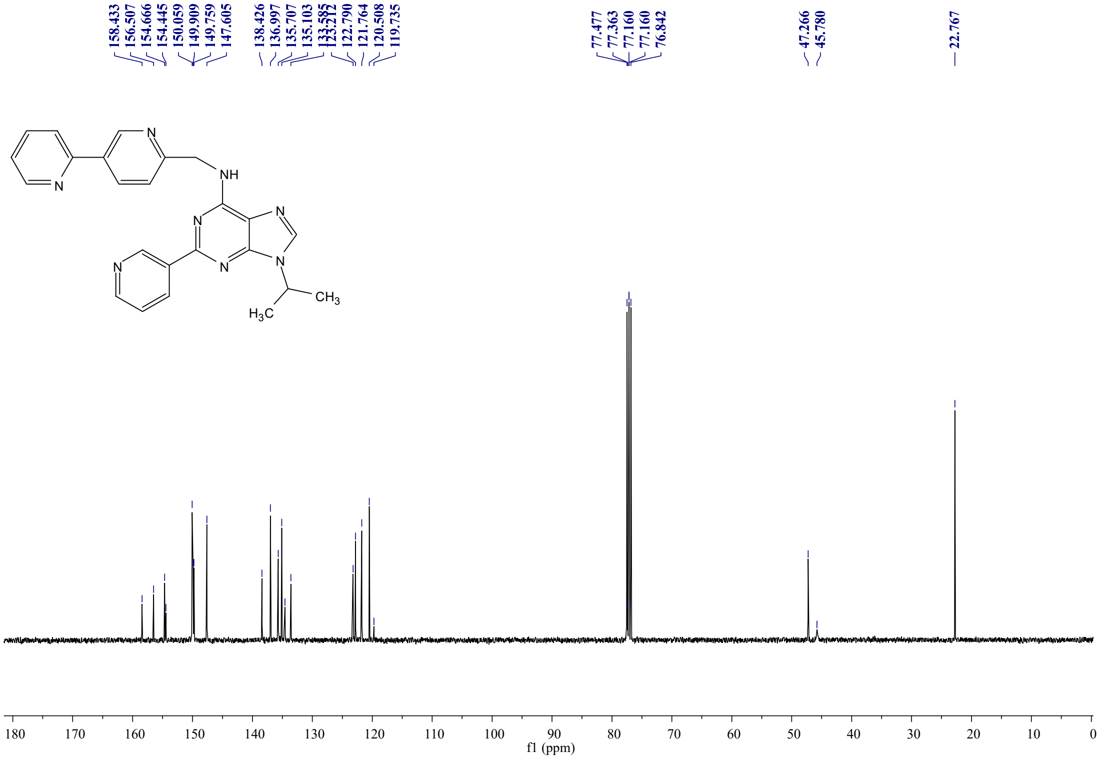

17b

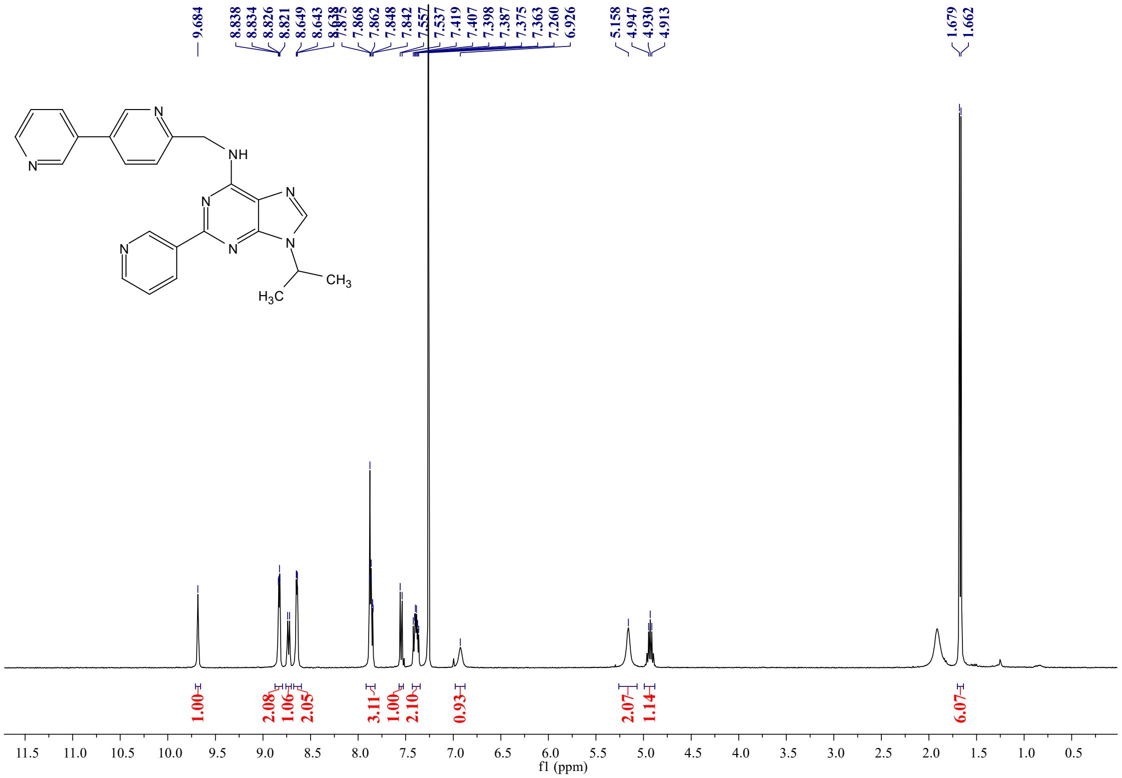

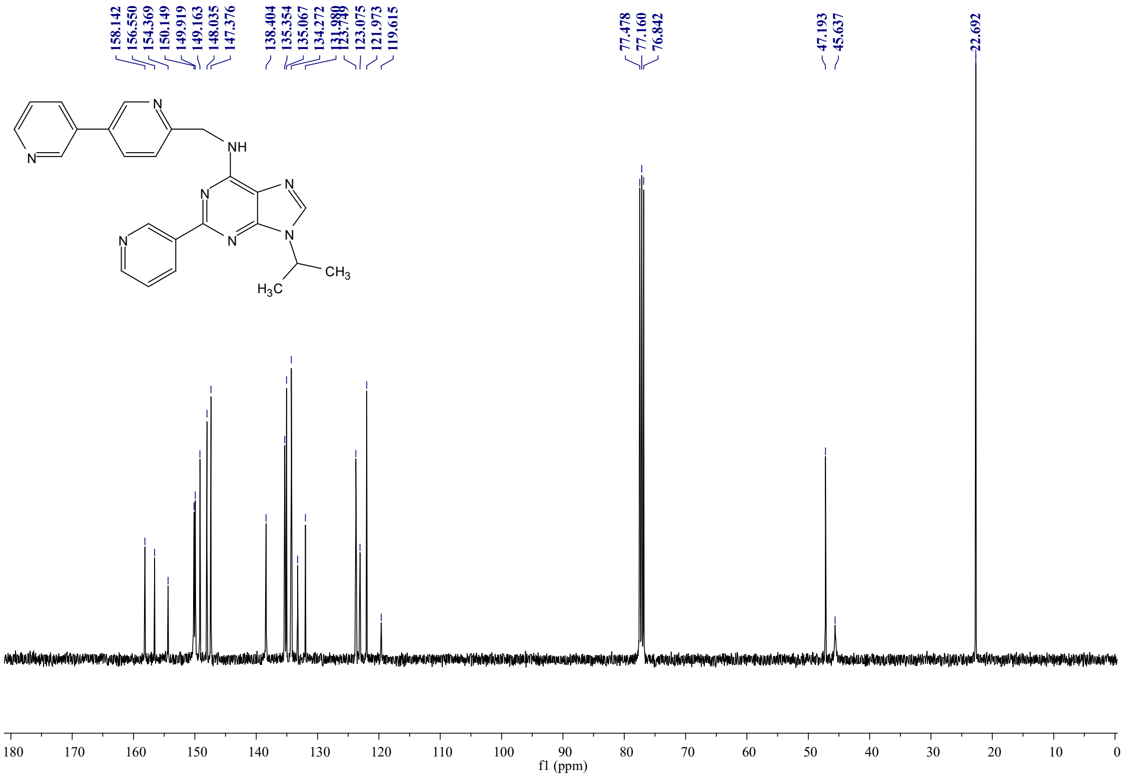

17c

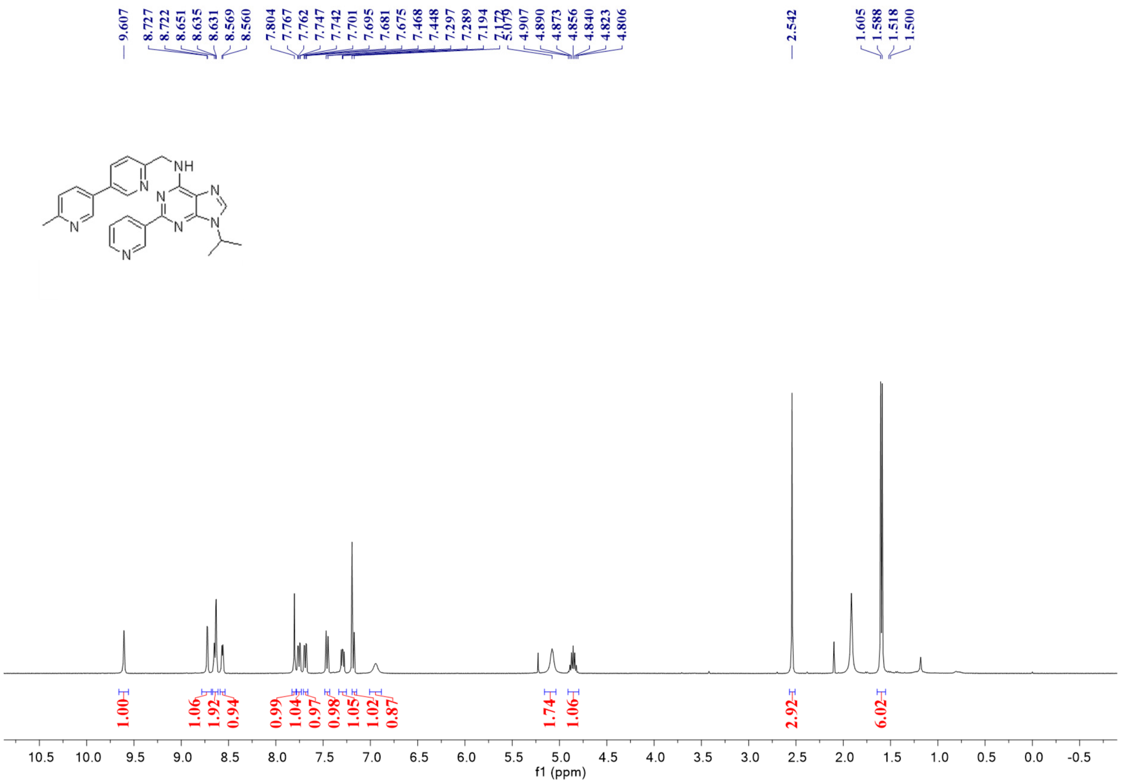

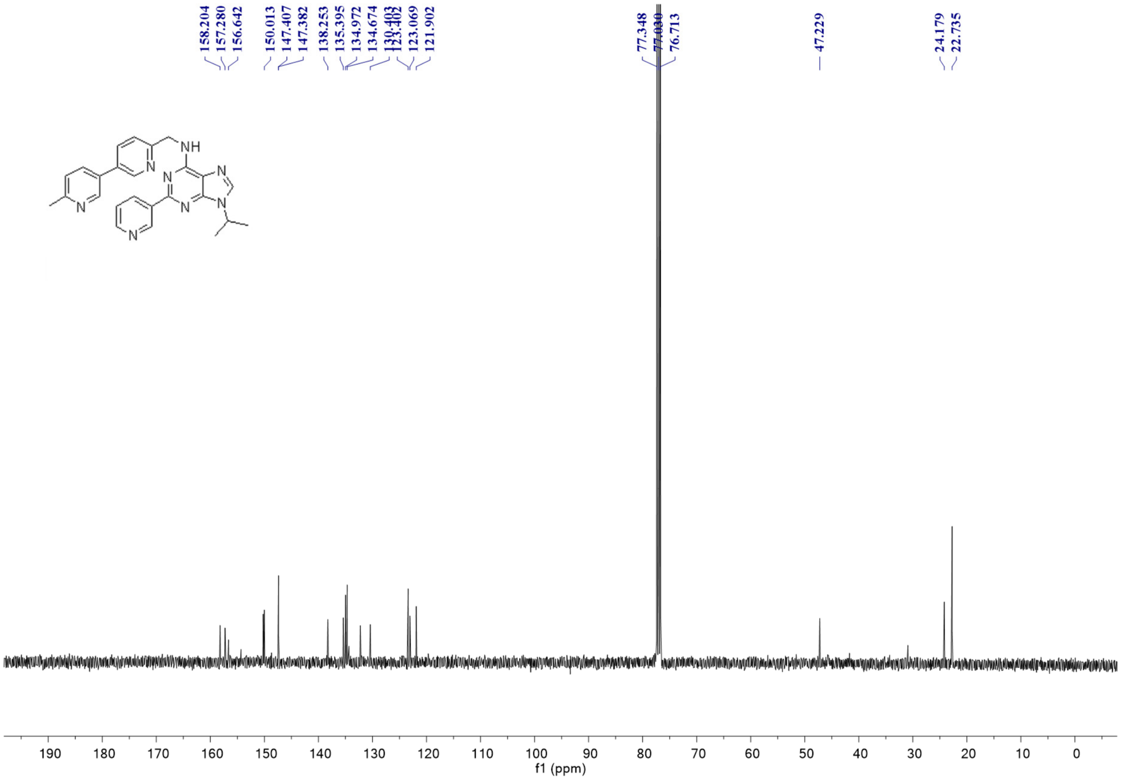

17d

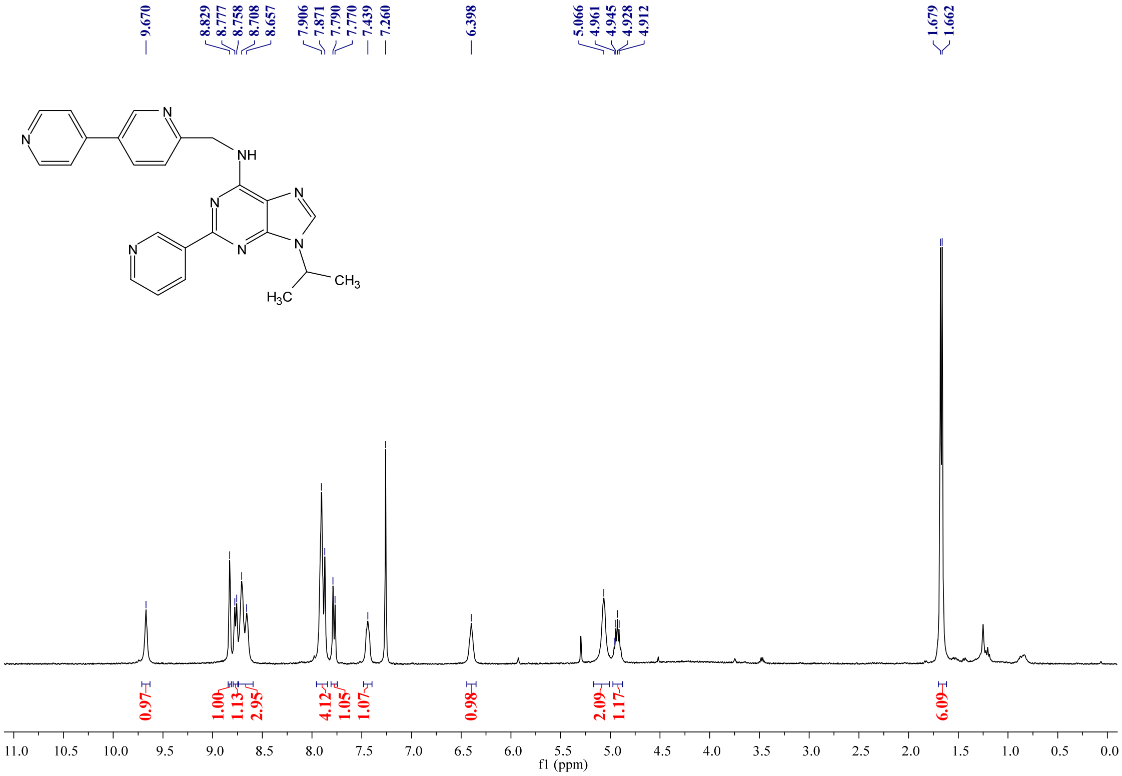

9.670
8.829
8.777
8.758
8.708
8.657
7.906
7.871
7.790
7.770
7.439
7.260
6.398
5.066
4.961
4.945
4.928
4.912
1.679
1.662
NH
N
CH3
H3C
0.97
1.00
1.13
2.95
4.12
1.05
1.07
0.98
2.09
1.17
6.09
f1 (ppm)

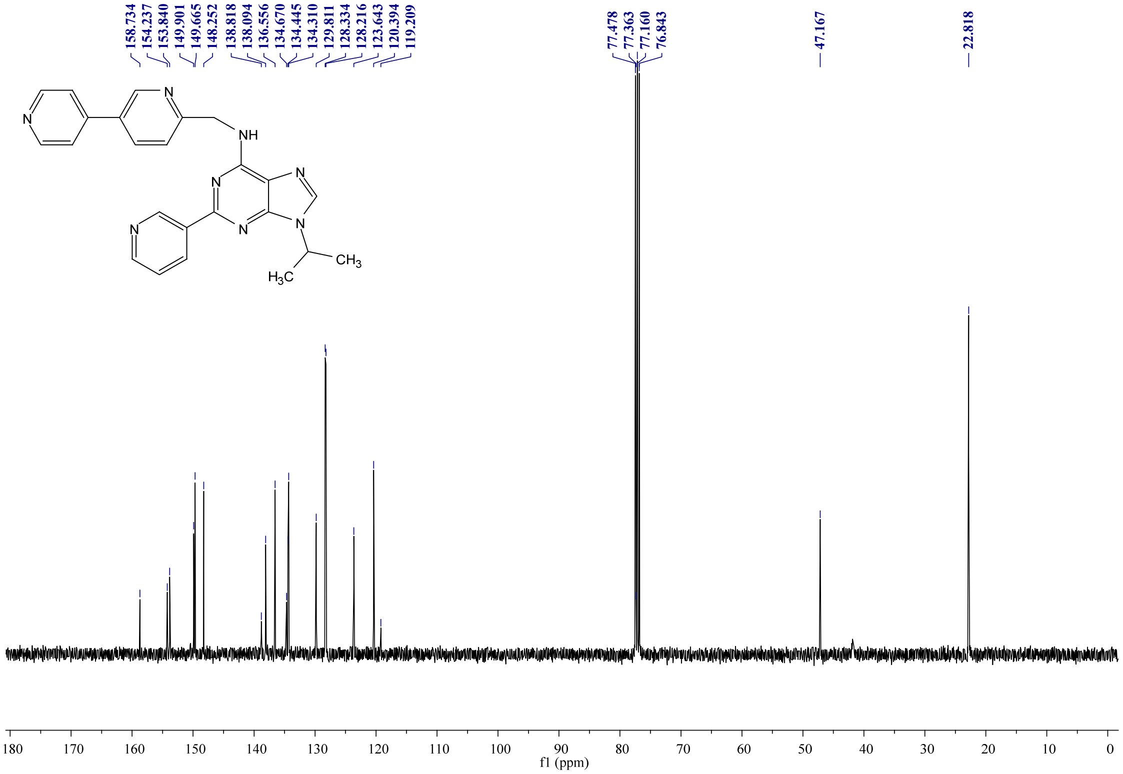

158.734
154.237
153.840
149.901
149.665
148.252
138.818
138.094
136.556
134.670
134.445
134.310
129.811
128.334
128.216
123.643
120.394
119.209
77.478
77.363
77.160
76.843
47.167
22.818
NH
N
CH3
H3C
f1 (ppm)

17e

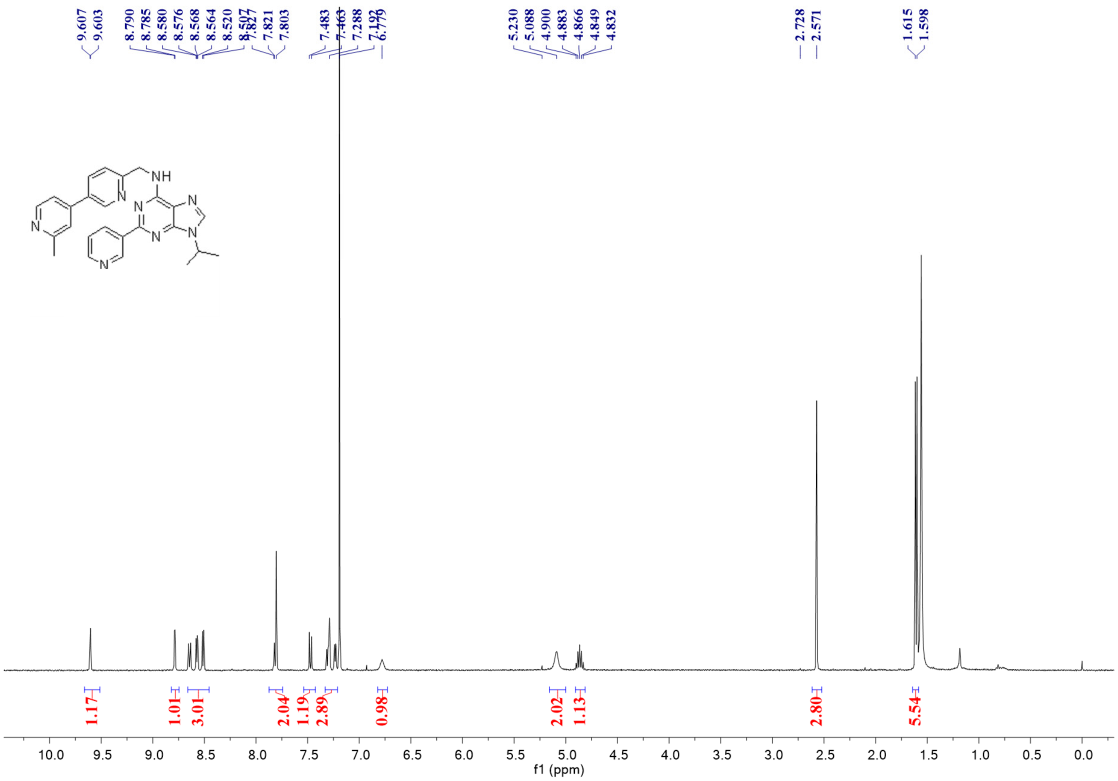

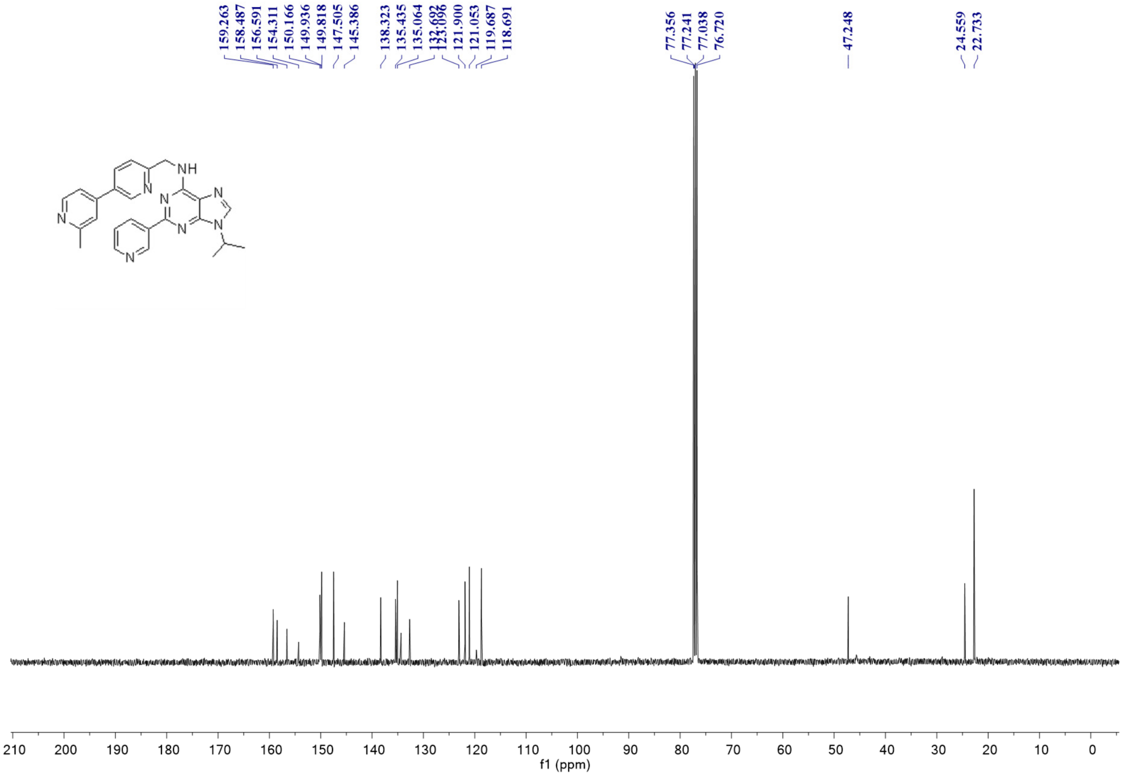

17f

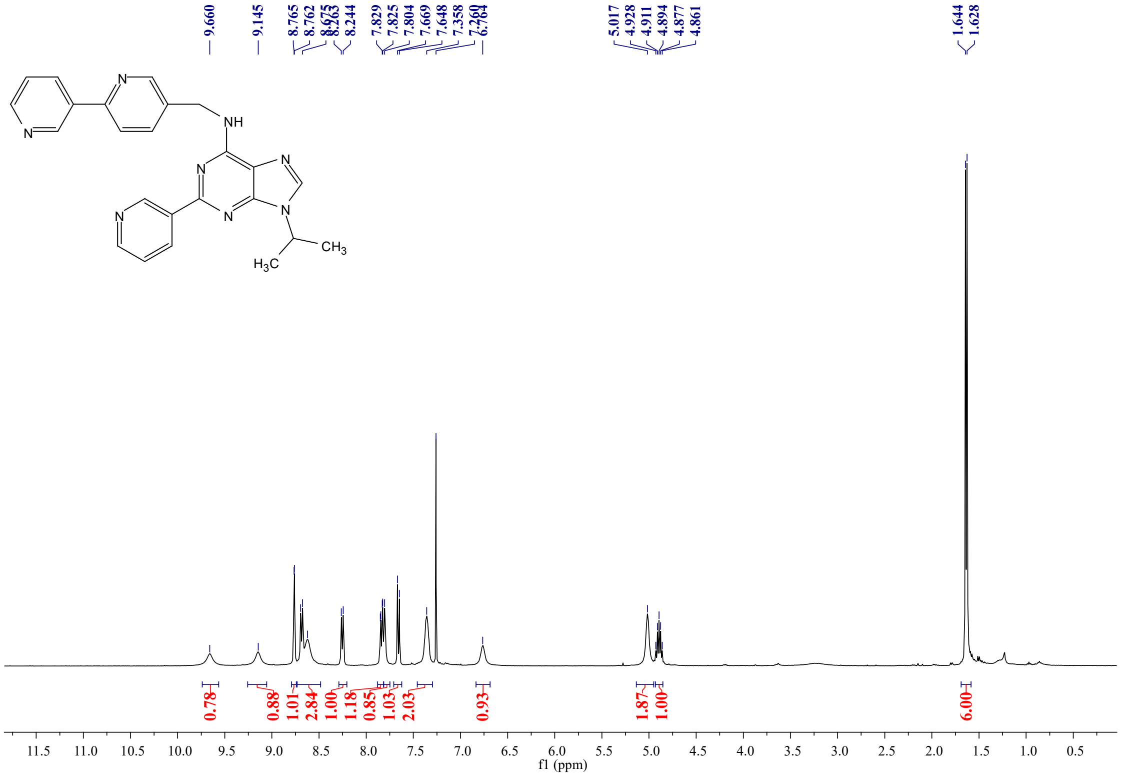
9.660
9.145
8.765
8.762
8.675
8.263
8.244
7.829
7.825
7.804
7.669
7.648
7.358
7.260
6.764
5.017
4.928
4.911
4.894
4.877
4.861
1.644
1.628
0.78
0.88
1.01
2.84
1.00
1.18
0.85
1.03
2.03
0.93
1.87
1.00
6.00
f1 (ppm)

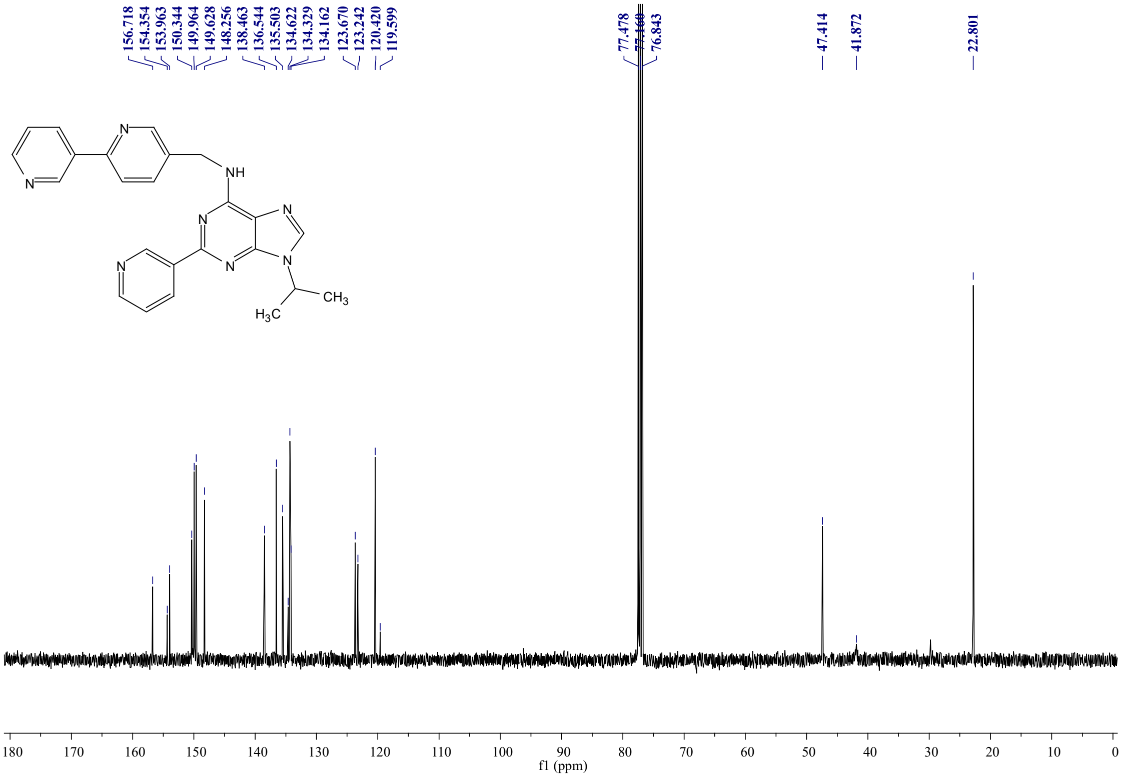
156.718
154.354
153.963
150.344
149.964
149.628
148.256
138.463
136.544
135.503
134.622
134.329
134.162
123.670
123.242
120.420
119.599
77.478
77.160
76.843
47.414
41.872
22.801
f1 (ppm)

17g

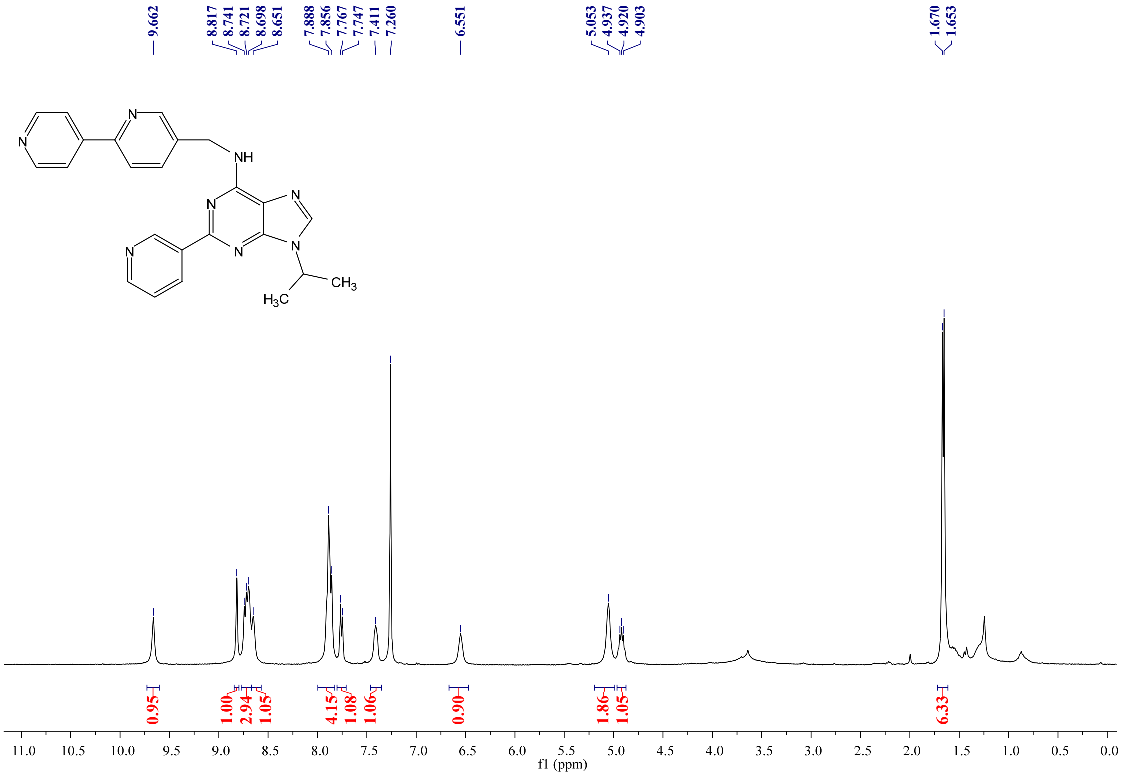
9.662
8.817
8.741
8.721
8.698
8.651
7.888
7.856
7.767
7.747
7.411
7.260
6.551
5.053
4.937
4.920
4.903
1.670
1.653
N
N
NH
N
N
N
N
N
CH3
H3C
0.95
1.00
2.94
1.05
4.15
1.08
1.06
0.90
1.86
1.05
6.33
11.0 10.5 10.0 9.5 9.0 8.5 8.0 7.5 7.0 6.5 6.0 5.5 5.0 4.5 4.0 3.5 3.0 2.5 2.0 1.5 1.0 0.5 0.0
f1 (ppm)

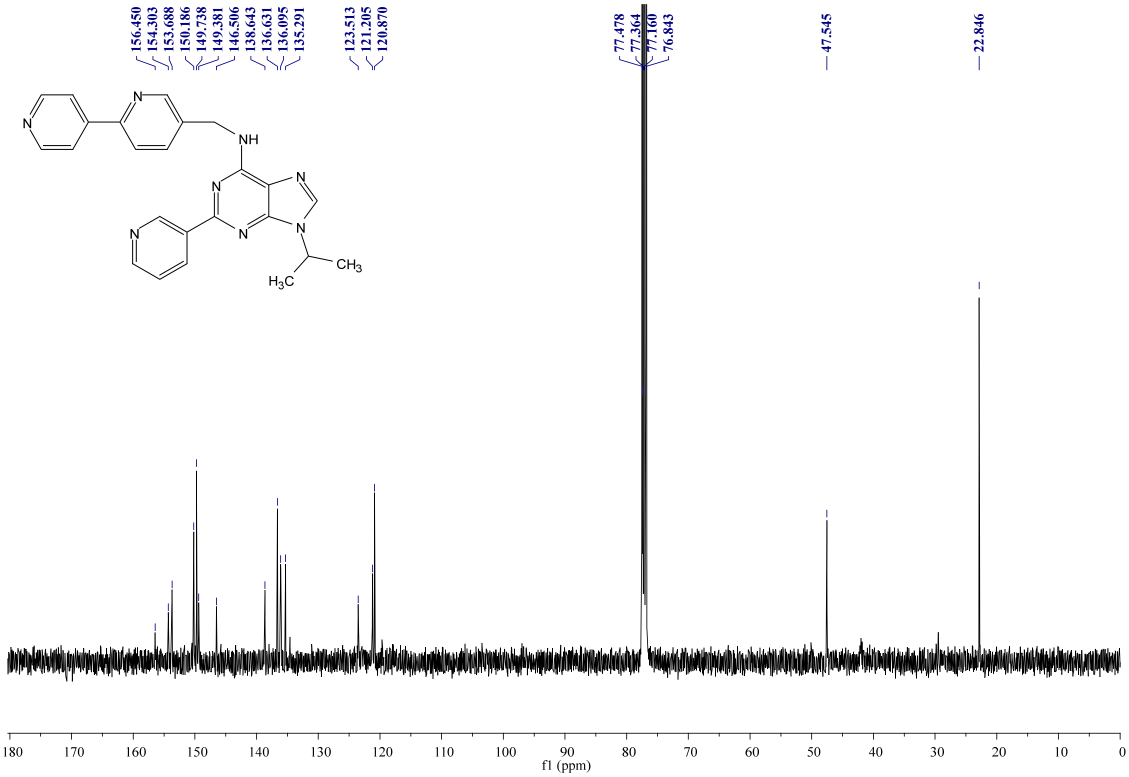
156.450
154.303
153.688
150.186
149.738
149.381
146.506
138.643
136.631
136.095
135.291
123.513
121.205
120.870
77.478
77.364
77.160
76.843
47.545
22.846
N
N
NH
N
N
N
N
N
CH3
H3C
180 170 160 150 140 130 120 110 100 90 80 70 60 50 40 30 20 10 0
f1 (ppm)

18a

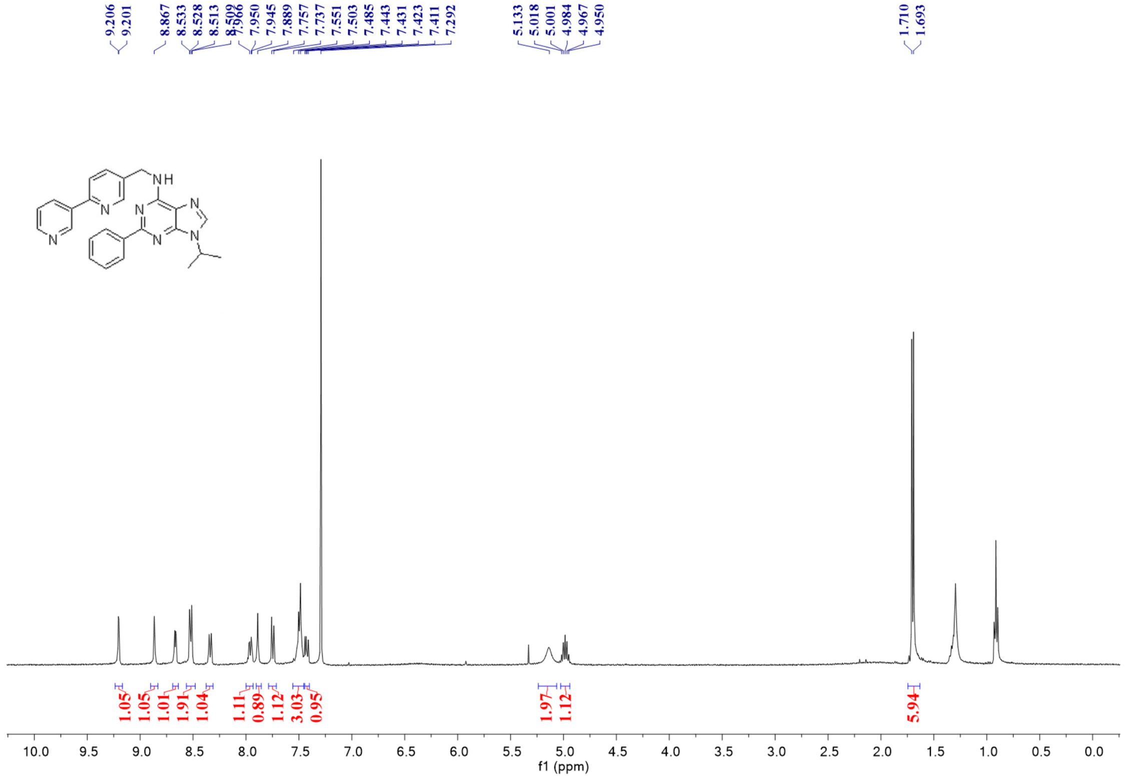
9.206
9.201
8.867
8.533
8.528
8.513
8.509
7.966
7.950
7.945
7.889
7.757
7.737
7.551
7.503
7.485
7.443
7.431
7.423
7.411
7.292
5.133
5.018
5.001
4.984
4.967
4.950
1.710
1.693
NH
N
N
N
N
N
N
1.05
1.05
1.01
1.91
1.04
1.11
0.89
1.12
3.03
0.95
1.97
1.12
5.94
10.0 9.5 9.0 8.5 8.0 7.5 7.0 6.5 6.0 5.5 5.0 4.5 4.0 3.5 3.0 2.5 2.0 1.5 1.0 0.5 0.0
f1 (ppm)

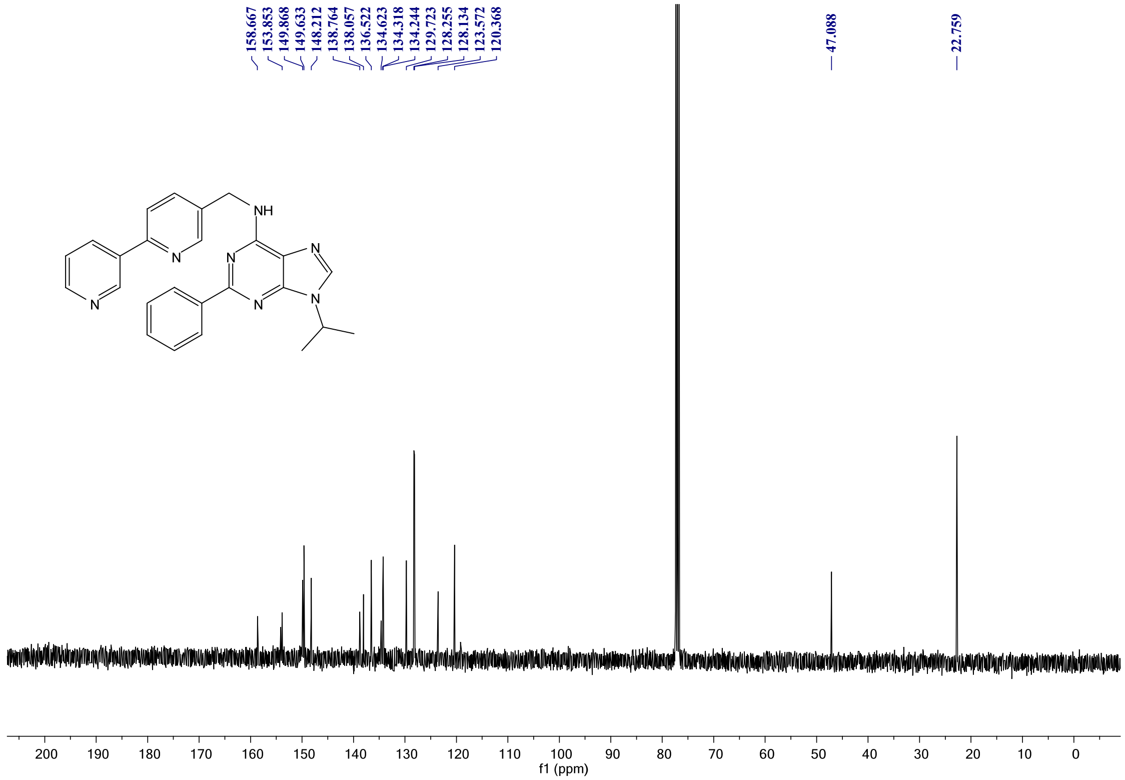
158.667
153.853
149.868
149.633
148.212
138.764
138.057
136.522
134.623
134.318
134.244
129.723
128.255
128.134
123.572
120.368
47.088
22.759
NH
N
N
N
N
N
N
200 190 180 170 160 150 140 130 120 110 100 90 80 70 60 50 40 30 20 10 0
f1 (ppm)

18b

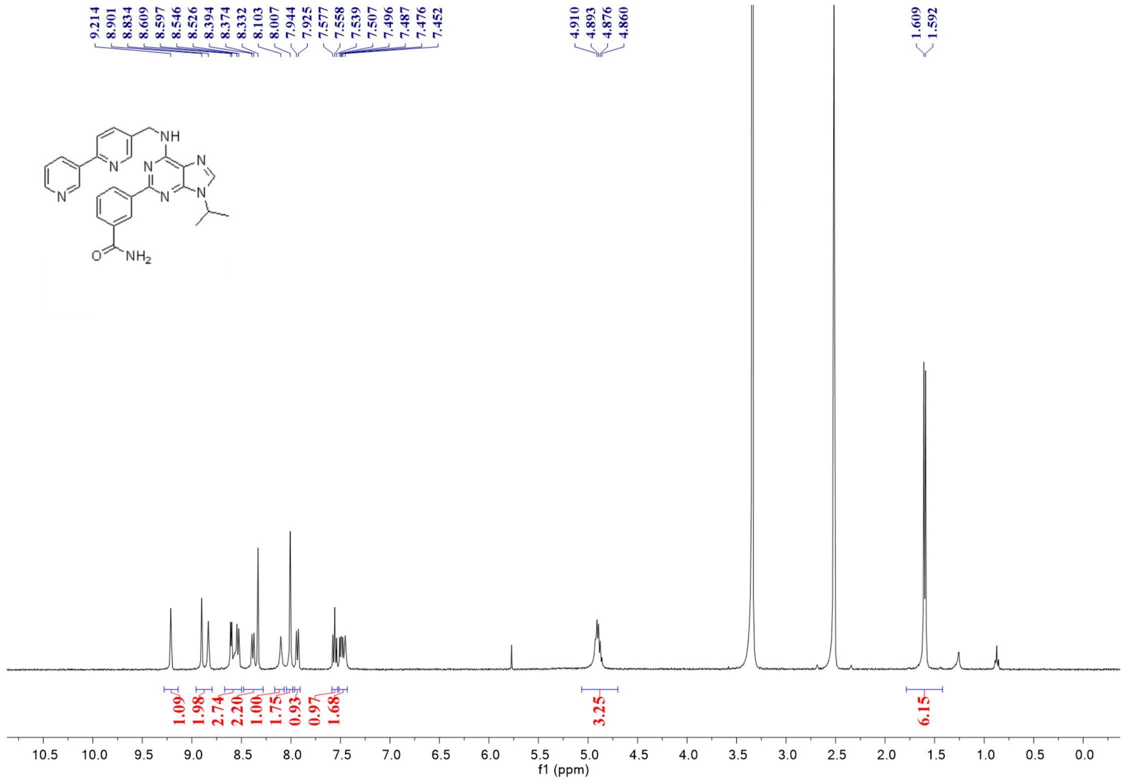
9.214 8.901 8.834 8.609 8.597 8.546 8.526 8.394 8.374 8.332 8.103 8.007 7.944 7.925 7.577 7.558 7.539 7.507 7.496 7.487 7.476 7.452
4.910 4.893 4.876 4.860
1.609 1.592
NH
N
N
N
N
N
O
NH2
1.09 1.98 2.74 2.20 1.00 1.75 0.93 0.97 1.68
3.25
6.15
10.5 10.0 9.5 9.0 8.5 8.0 7.5 7.0 6.5 6.0 5.5 5.0 4.5 4.0 3.5 3.0 2.5 2.0 1.5 1.0 0.5 0.0
f1 (ppm)

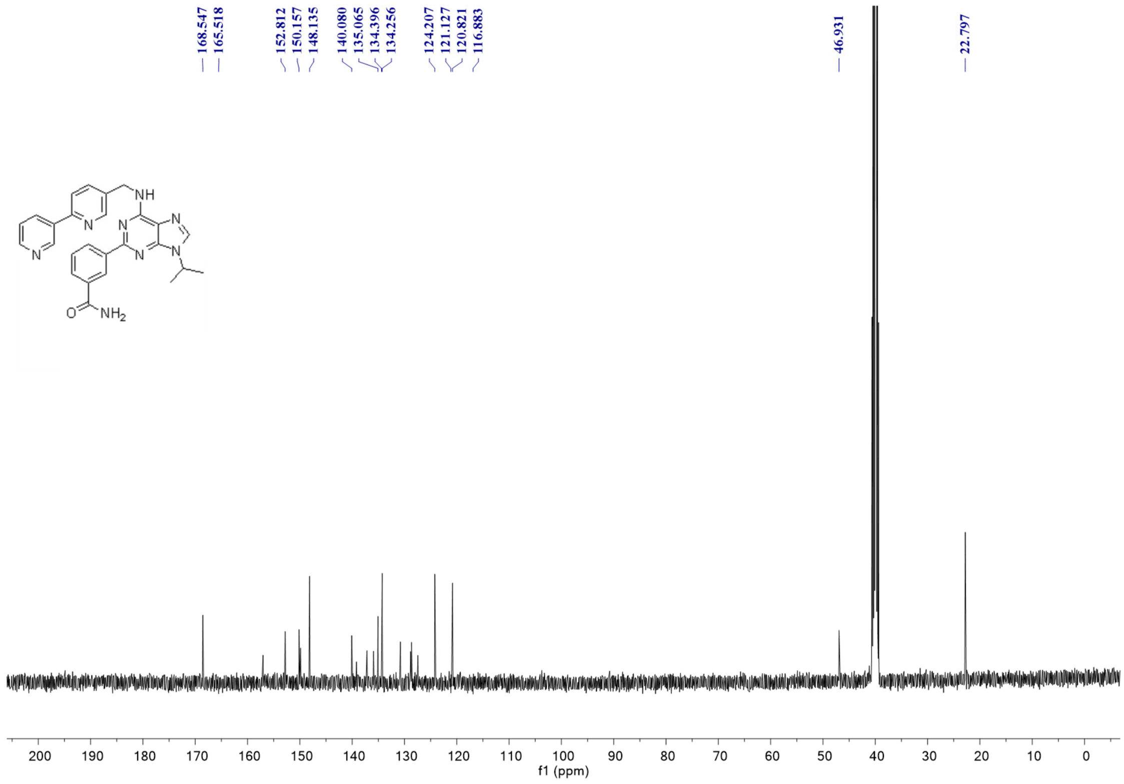
168.547 165.518
152.812 150.157 148.135
140.080 135.065 134.396 134.256
124.207 121.127 120.821 116.883
46.931
22.797
NH
N
N
N
N
N
O
NH2
200 190 180 170 160 150 140 130 120 110 100 90 80 70 60 50 40 30 20 10 0
f1 (ppm)

18c

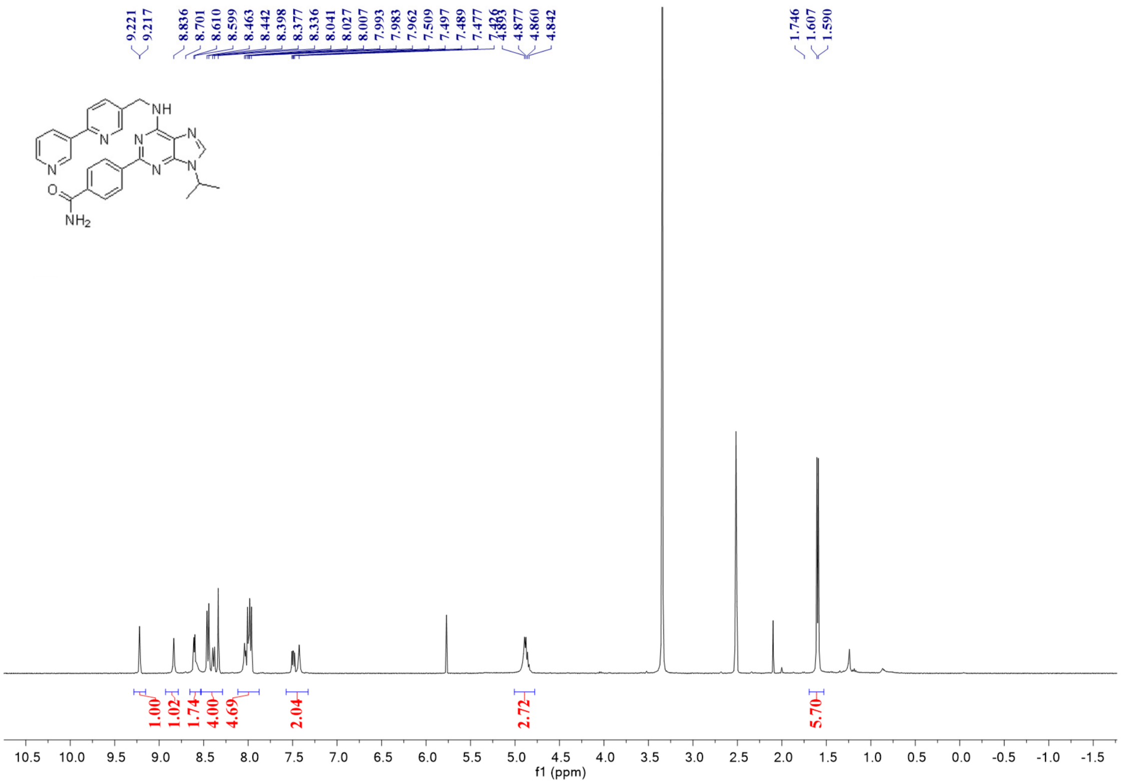
9.221
9.217
8.836
8.701
8.610
8.599
8.463
8.442
8.398
8.377
8.336
8.041
8.027
8.007
7.993
7.983
7.962
7.509
7.497
7.489
7.477
4.877
4.860
4.842
1.746
1.607
1.590
1.00
1.02
1.74
4.00
4.69
2.04
2.72
5.70
f1 (ppm)

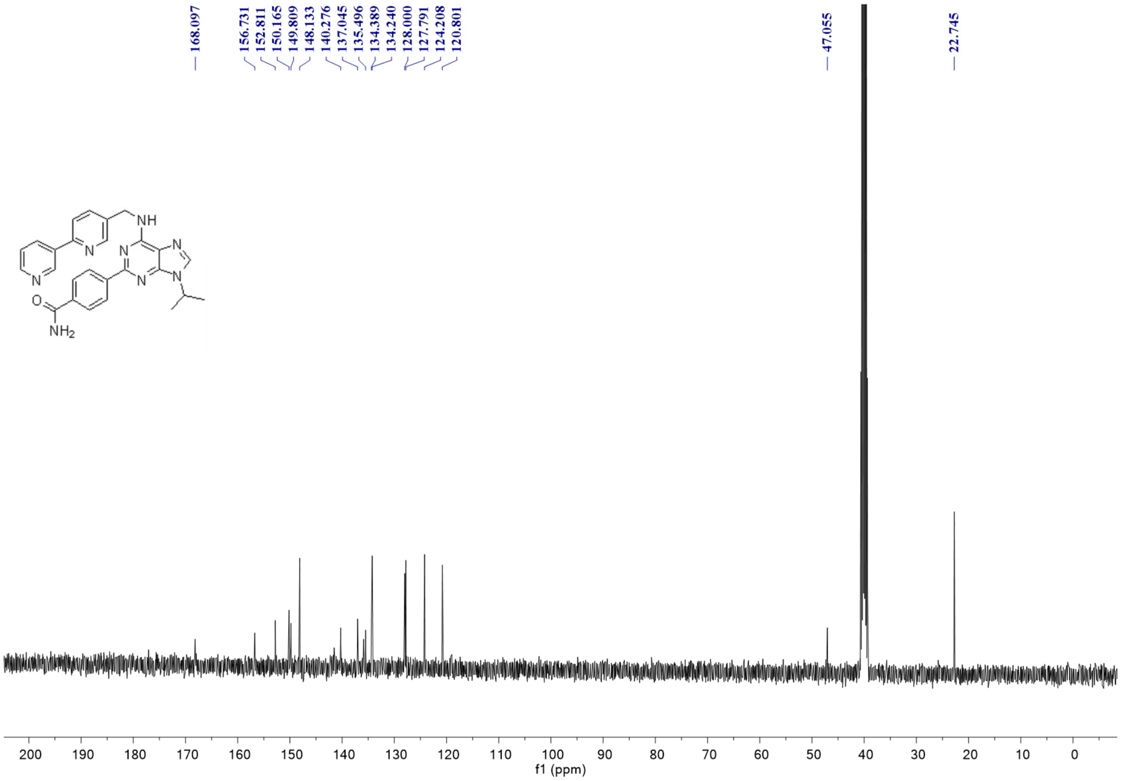
168.097
156.731
152.811
150.165
149.809
148.133
140.276
137.045
135.496
134.389
134.240
128.000
127.791
124.208
120.801
47.055
22.745
f1 (ppm)

18d

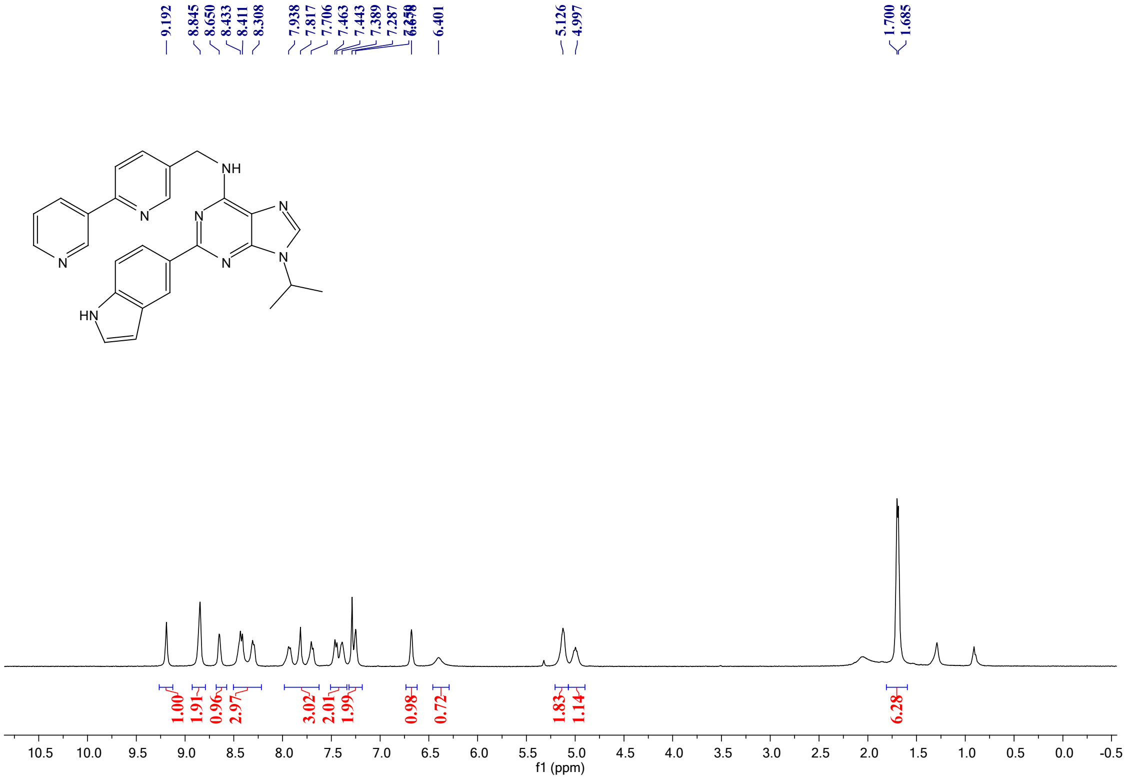

9.192
8.845
8.650
8.433
8.411
8.308
7.938
7.817
7.706
7.463
7.443
7.389
7.287
6.401
5.126
4.997
1.700
1.685
1.00
1.91
0.96
2.97
3.02
2.01
1.99
0.98
0.72
1.83
1.14
6.28
f1 (ppm)

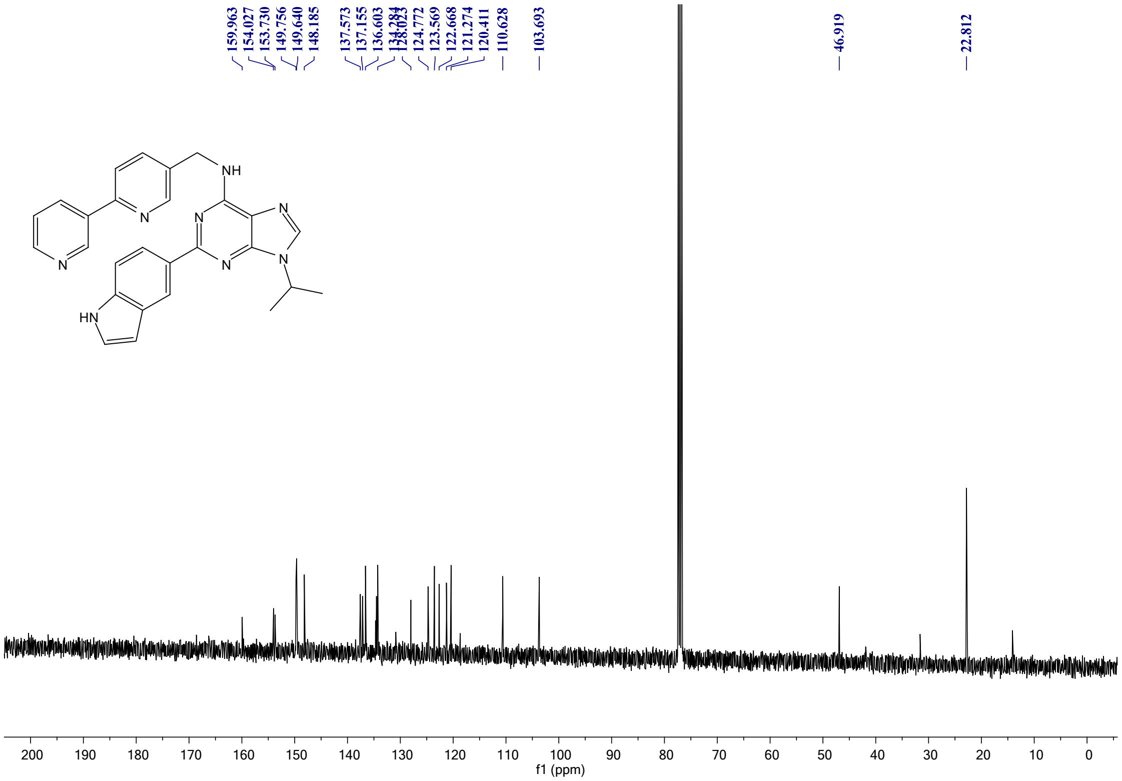

159.963
154.027
153.730
149.756
149.640
148.185
137.573
137.155
136.603
124.772
123.569
122.668
121.274
120.411
110.628
103.693
46.919
22.812
f1 (ppm)

18e

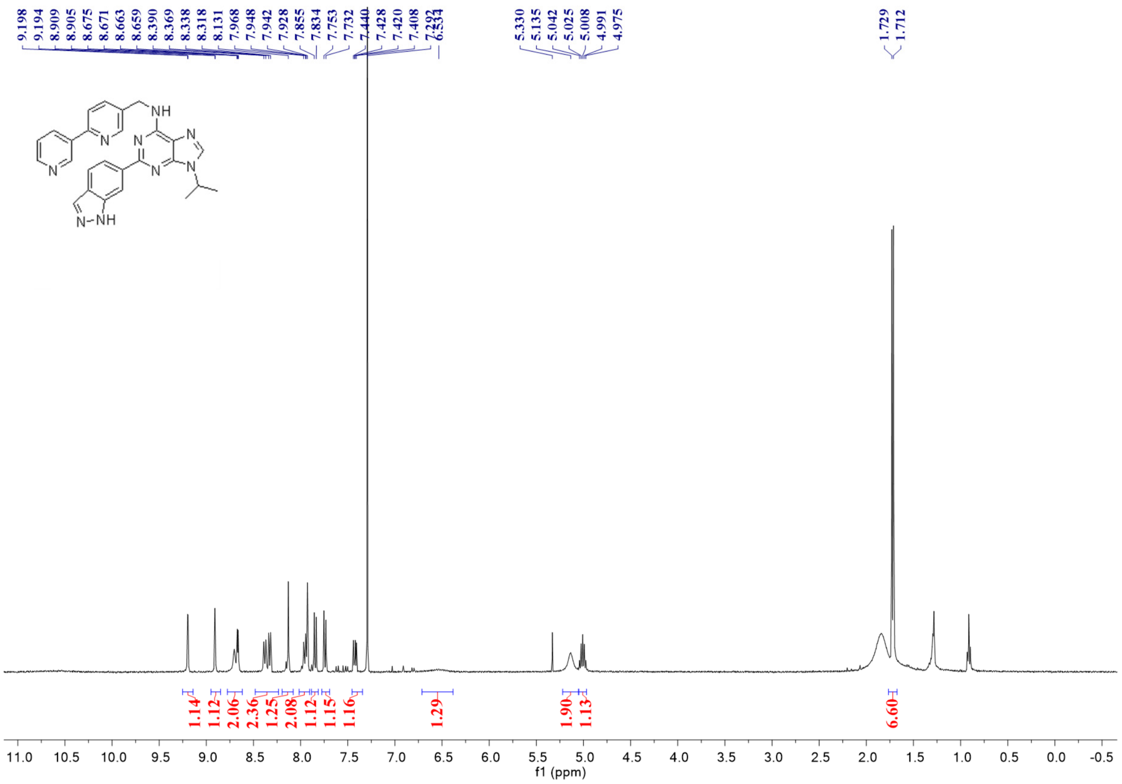

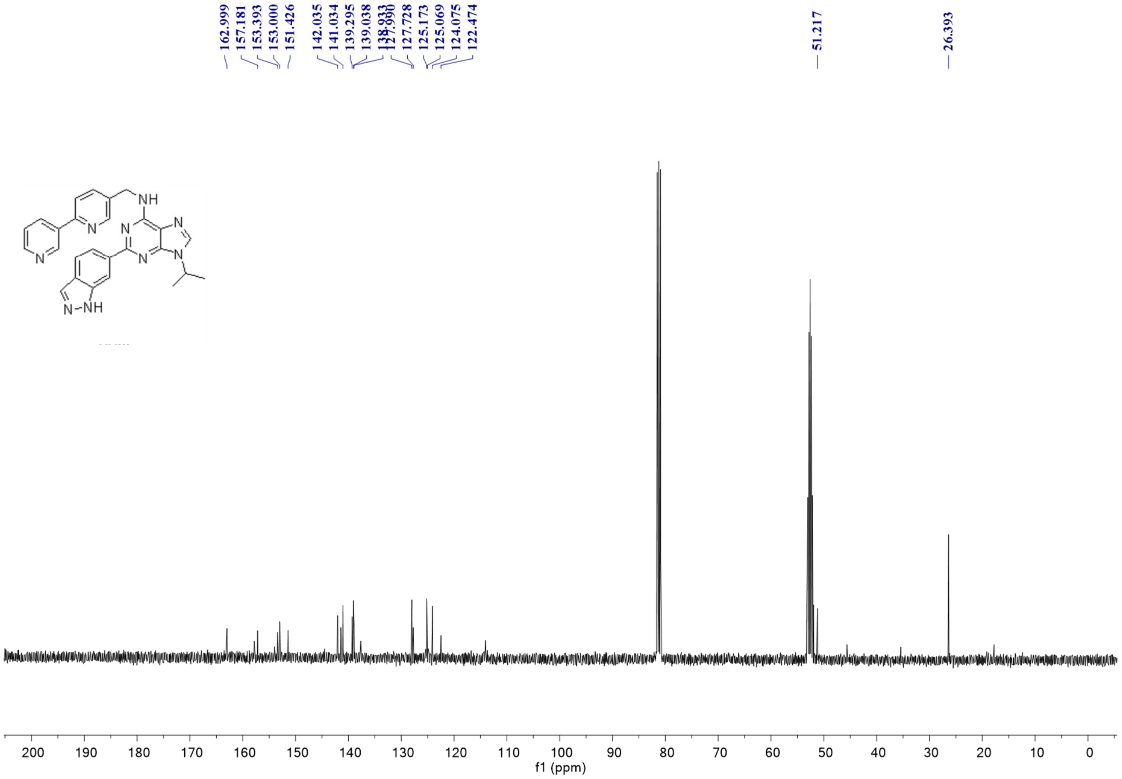

18f

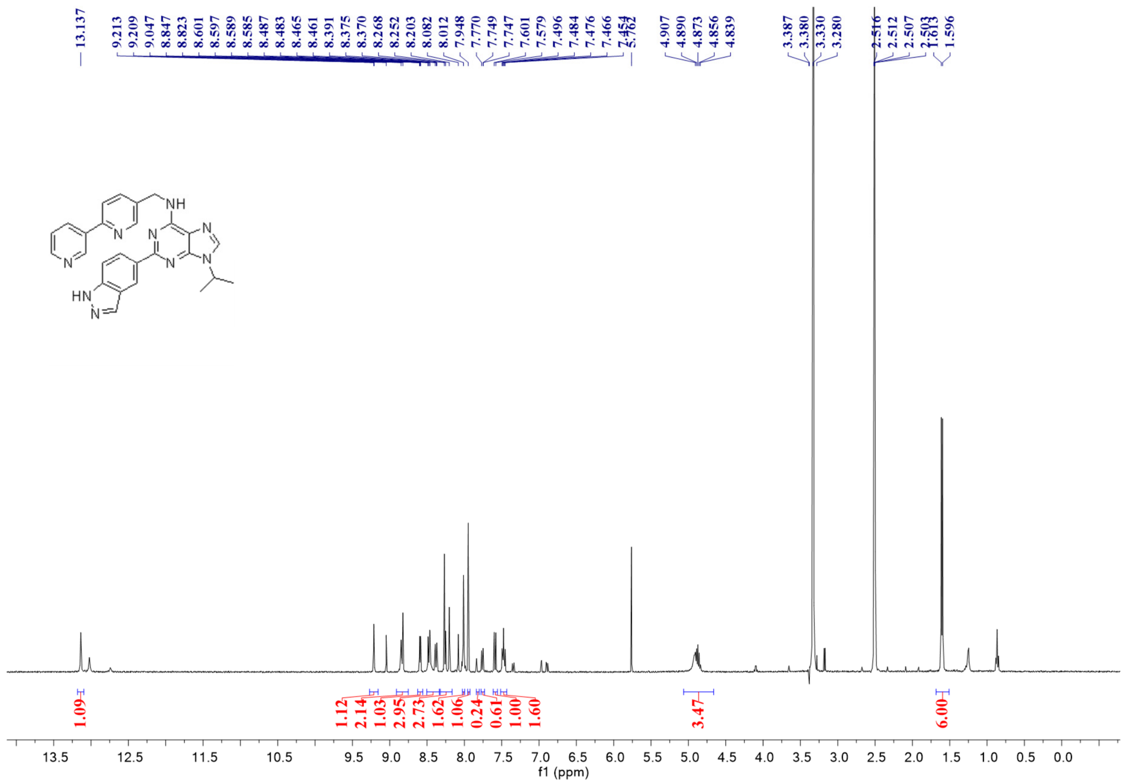
13.137
9.213
9.209
9.047
8.847
8.823
8.601
8.597
8.589
8.585
8.487
8.483
8.465
8.461
8.391
8.375
8.370
8.268
8.252
8.203
8.082
8.012
7.948
7.770
7.749
7.747
7.601
7.579
7.496
7.484
7.476
7.466
7.454
5.762
4.907
4.890
4.873
4.856
4.839
3.387
3.380
3.330
3.280
2.516
2.512
2.507
2.503
1.613
1.596
NH
N
N
N
N
N
HN
N
1.09
1.12
2.14
1.03
2.95
2.73
1.62
1.06
0.24
0.61
1.00
1.60
3.47
6.00
13.5 12.5 11.5 10.5 9.5 9.0 8.5 8.0 7.5 7.0 6.5 6.0 5.5 5.0 4.5 4.0 3.5 3.0 2.5 2.0 1.5 1.0 0.5 0.0
f1 (ppm)

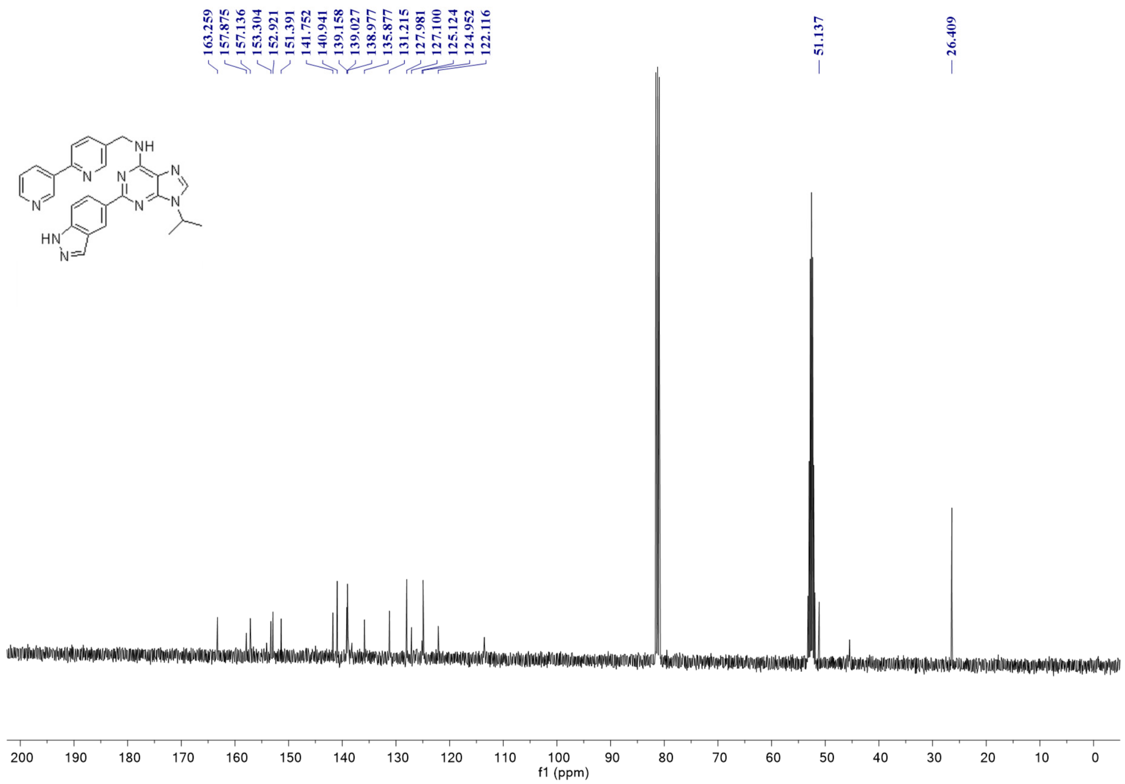
163.259
157.875
157.136
153.304
152.921
151.391
141.752
140.941
139.158
139.027
138.977
135.877
131.215
127.981
127.100
125.124
124.952
122.116
51.137
26.409
NH
N
N
N
N
N
HN
N
200 190 180 170 160 150 140 130 120 110 100 90 80 70 60 50 40 30 20 10 0
f1 (ppm)

18g

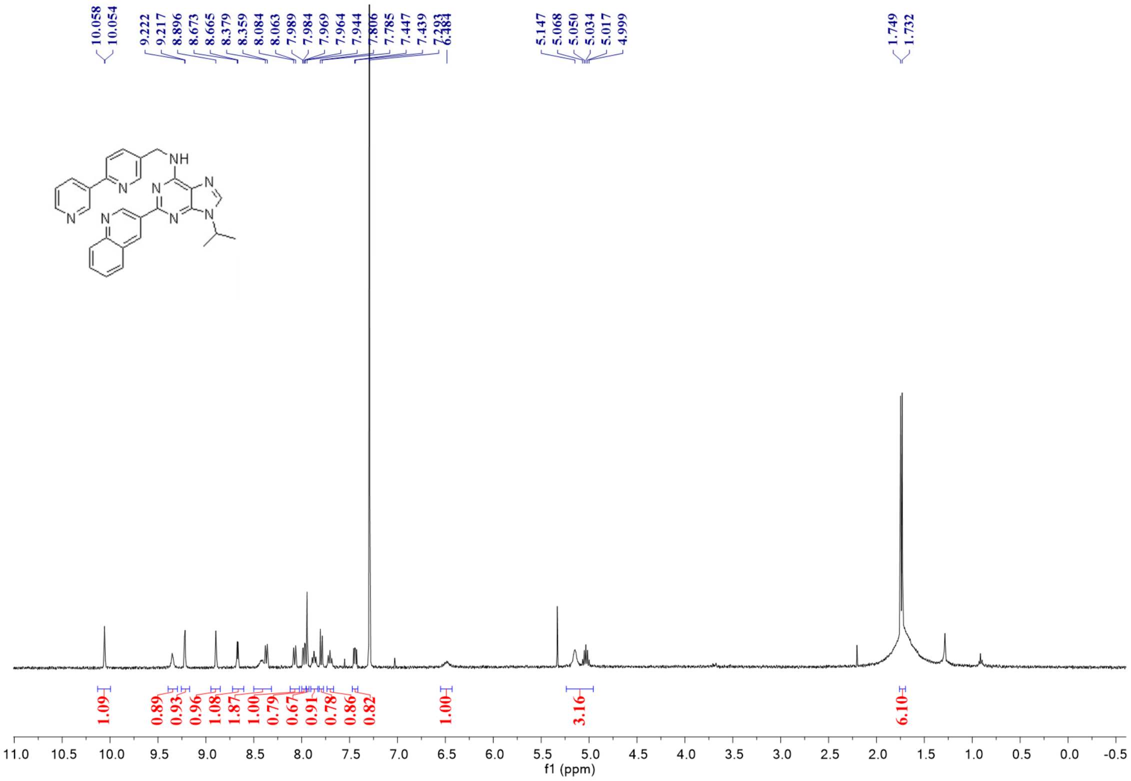

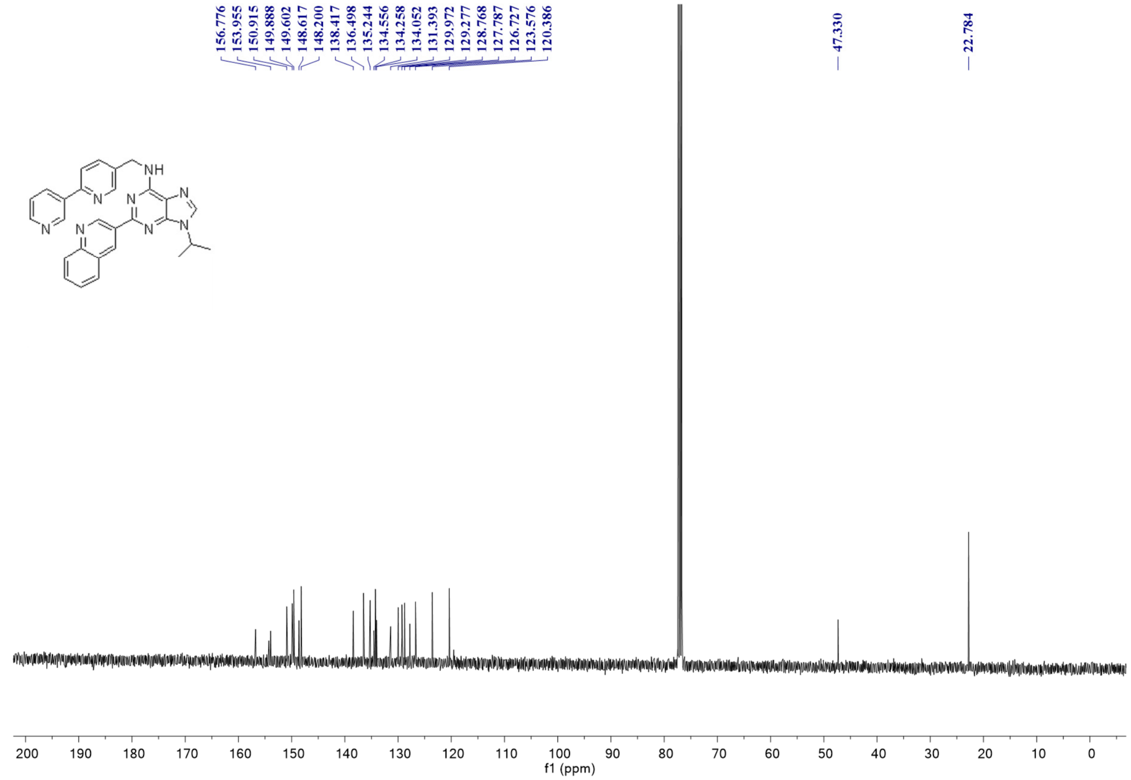

18h

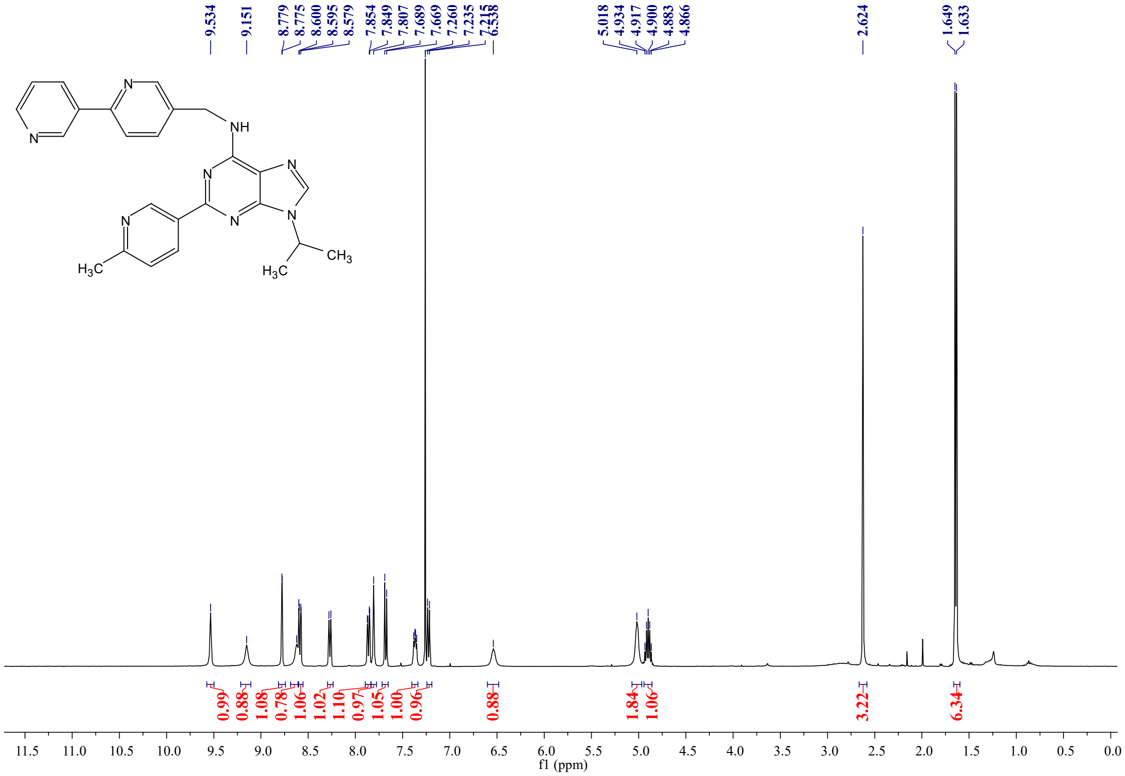

9.534
9.151
8.779
8.775
8.600
8.595
8.579
7.854
7.849
7.807
7.689
7.669
7.260
7.235
6.538
5.018
4.934
4.917
4.900
4.883
4.866
2.624
1.649
1.633
NH
N
H3C
CH3
0.99
0.88
1.08
0.78
1.06
1.02
1.10
0.97
1.05
1.00
0.96
0.88
1.84
1.06
3.22
6.34
f1 (ppm)

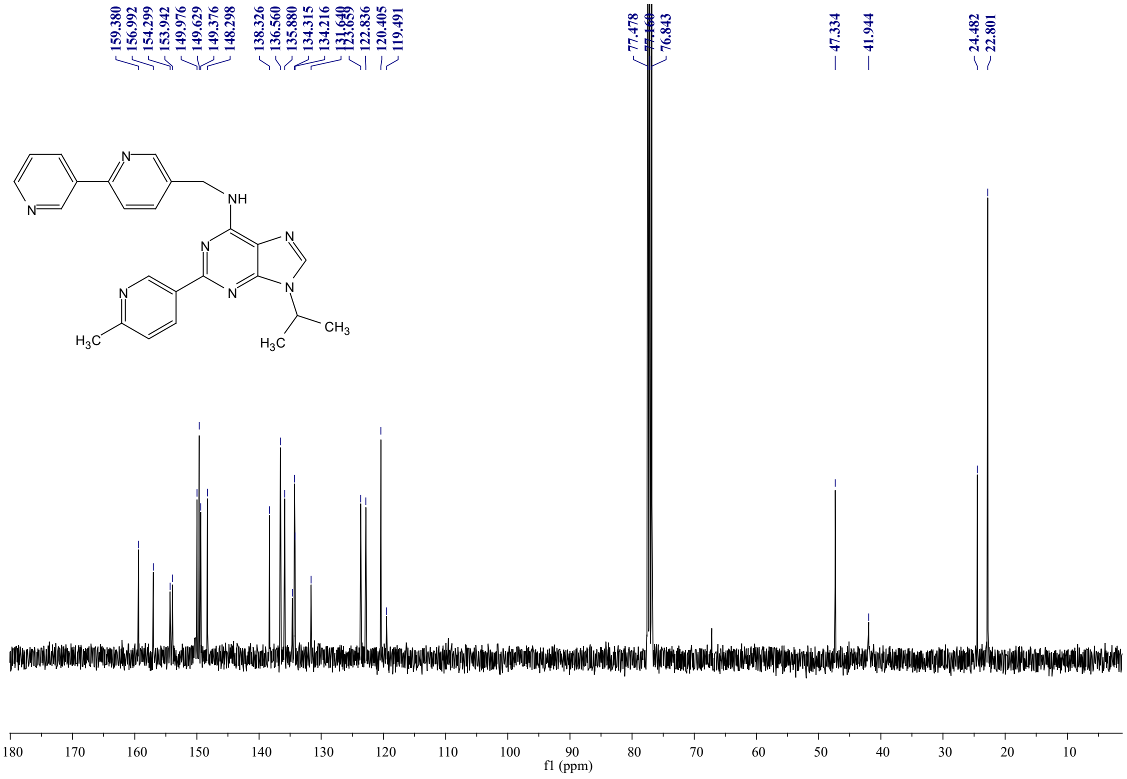

159.380
156.992
154.299
153.942
149.976
149.629
149.376
148.298
138.326
136.560
135.880
134.315
134.216
122.836
120.405
119.491
77.478
76.843
47.334
41.944
24.482
22.801
NH
N
H3C
CH3
f1 (ppm)

18i

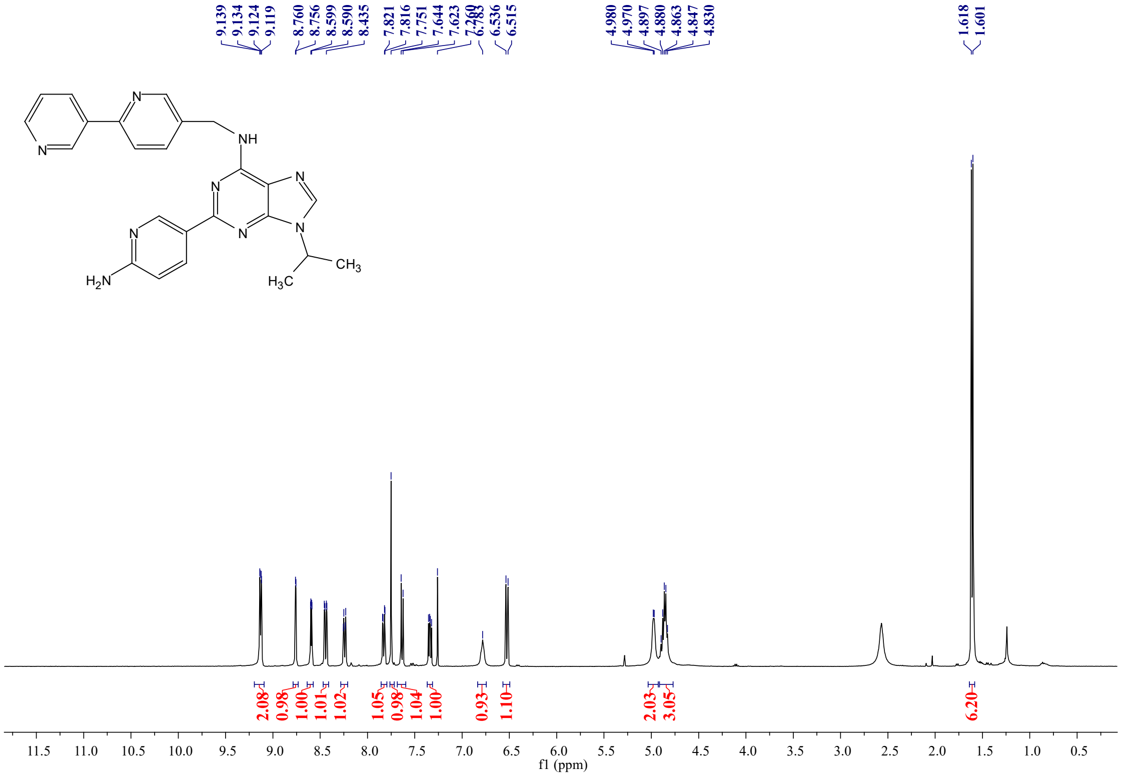

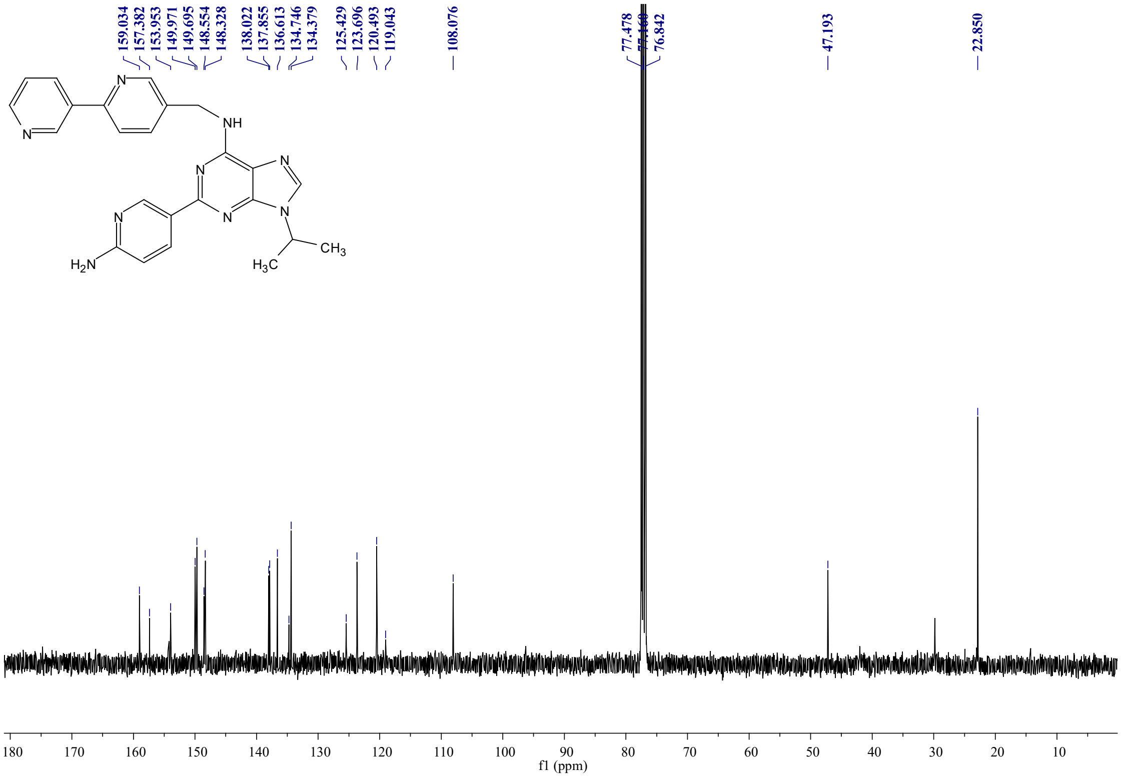

18j

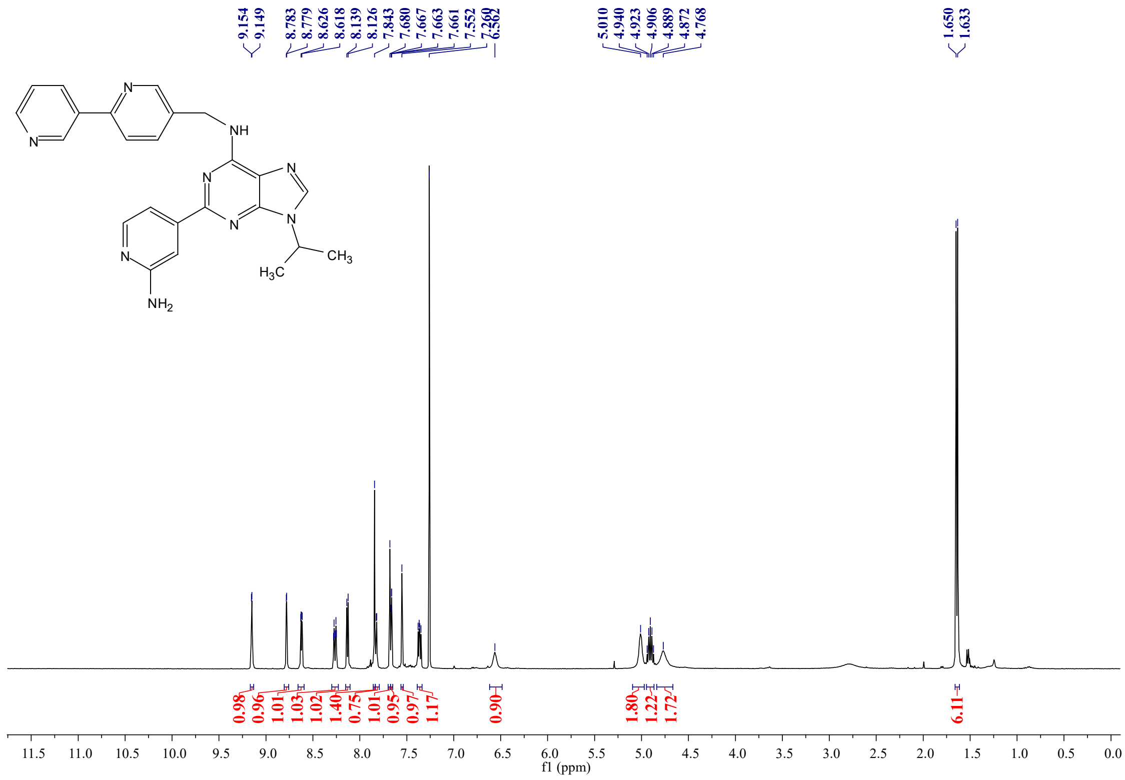
9.154
9.149
8.783
8.779
8.626
8.618
8.139
8.126
7.843
7.680
7.667
7.663
7.661
7.552
7.260
6.562
5.010
4.940
4.923
4.906
4.889
4.872
4.768
1.650
1.633
NH
N
N
N
N
N
N
N
CH3
H3C
NH2
0.98
0.96
1.01
1.03
1.02
1.40
0.75
1.01
0.95
0.97
1.17
0.90
1.80
1.22
1.72
6.11
11.5 11.0 10.5 10.0 9.5 9.0 8.5 8.0 7.5 7.0 6.5 6.0 5.5 5.0 4.5 4.0 3.5 3.0 2.5 2.0 1.5 1.0 0.5 0.0
f1 (ppm)

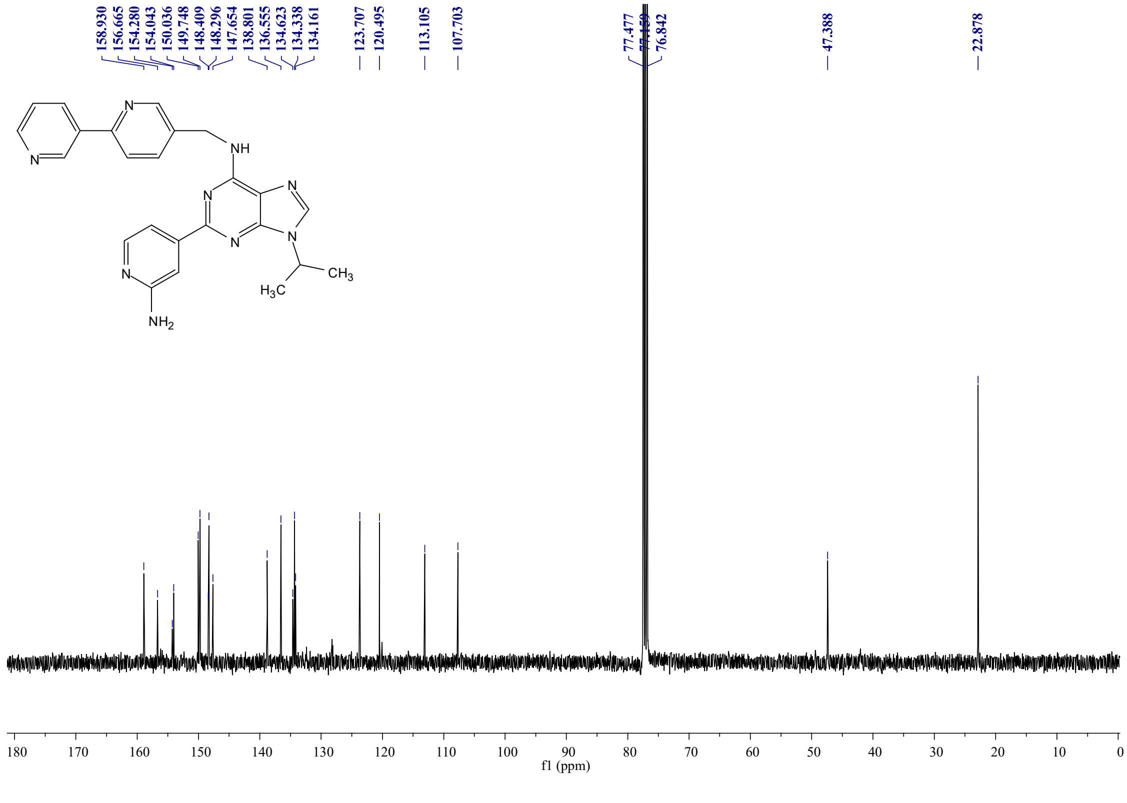
158.930
156.665
154.280
154.043
150.036
149.748
148.409
148.296
147.654
138.801
136.555
134.623
134.338
134.161
123.707
120.495
113.105
107.703
77.477
77.159
76.842
47.388
22.878
NH
N
N
N
N
N
N
N
CH3
H3C
NH2
180 170 160 150 140 130 120 110 100 90 80 70 60 50 40 30 20 10 0
f1 (ppm)

18k

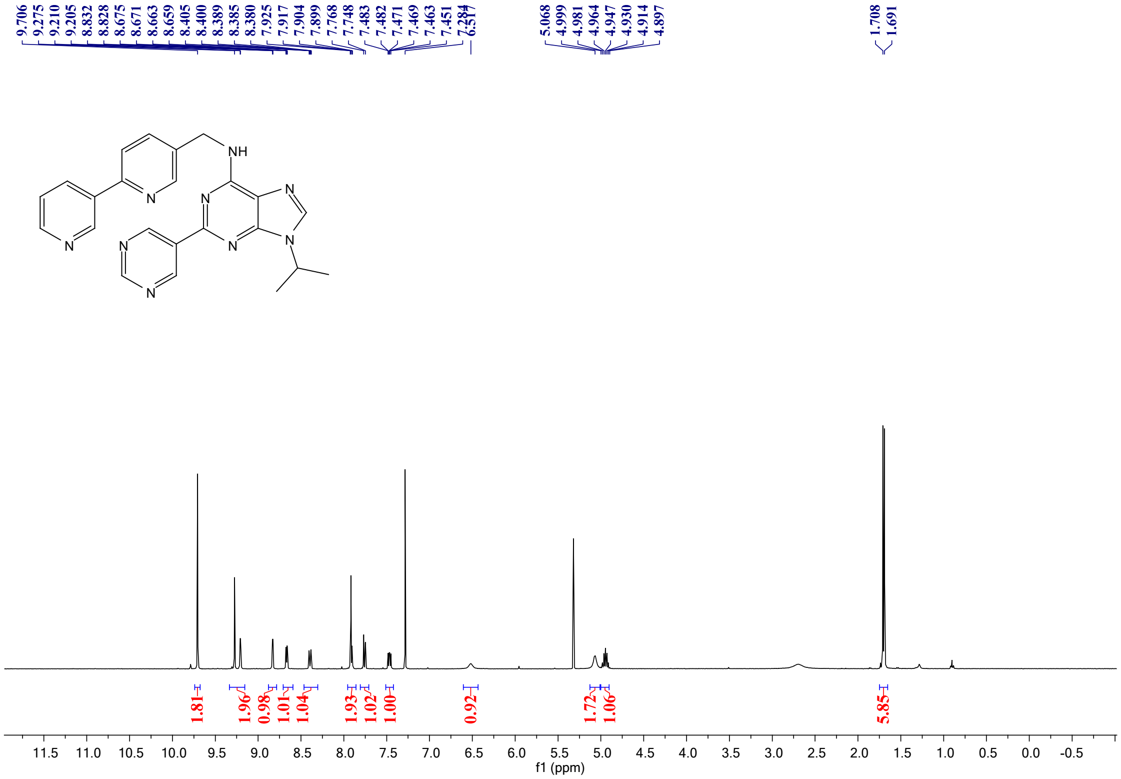
9.706
9.275
9.210
9.205
8.832
8.828
8.675
8.671
8.663
8.659
8.405
8.400
8.389
8.385
8.380
7.925
7.917
7.904
7.899
7.768
7.748
7.483
7.482
7.471
7.469
7.463
7.451
7.284
6.517
5.068
4.999
4.981
4.964
4.947
4.930
4.914
4.897
1.708
1.691
NH
N
N
N
N
N
N
N
N
1.81
1.96
0.98
1.01
1.04
1.93
1.02
1.00
0.92
1.72
1.06
5.85
f1 (ppm)

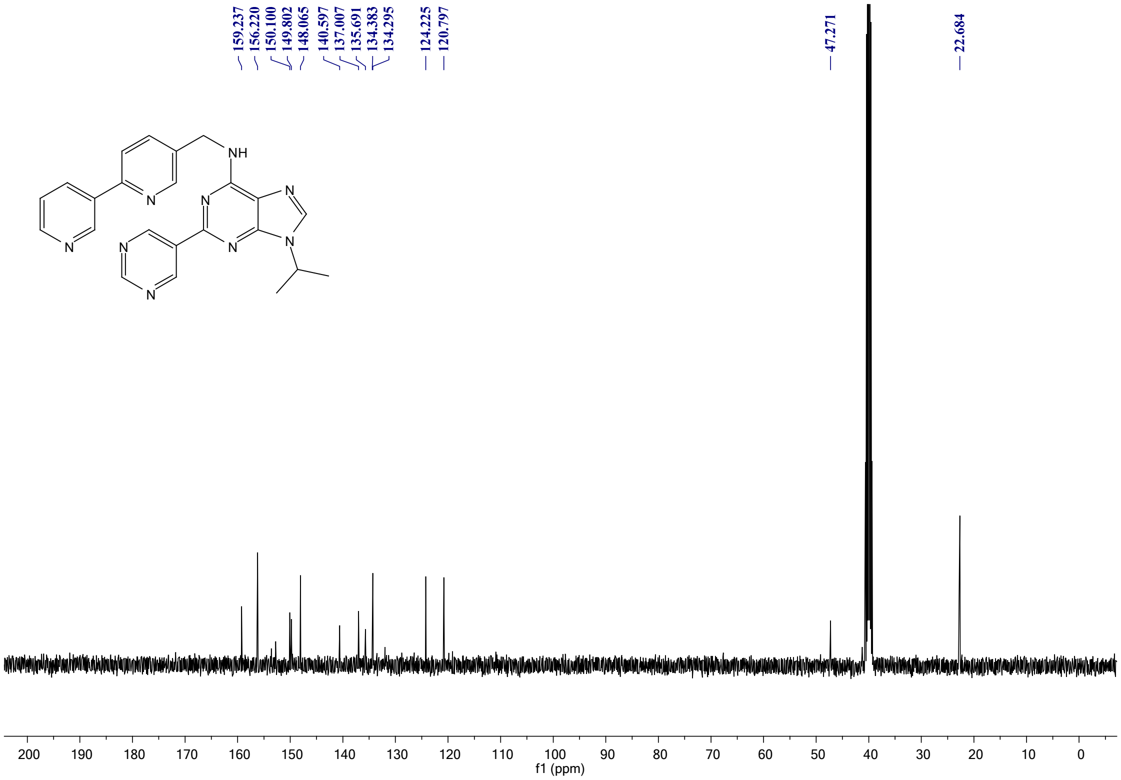
159.237
156.220
150.100
149.802
148.065
140.597
137.007
135.691
134.383
134.295
124.225
120.797
47.271
22.684
NH
N
N
N
N
N
N
N
N
f1 (ppm)

18l

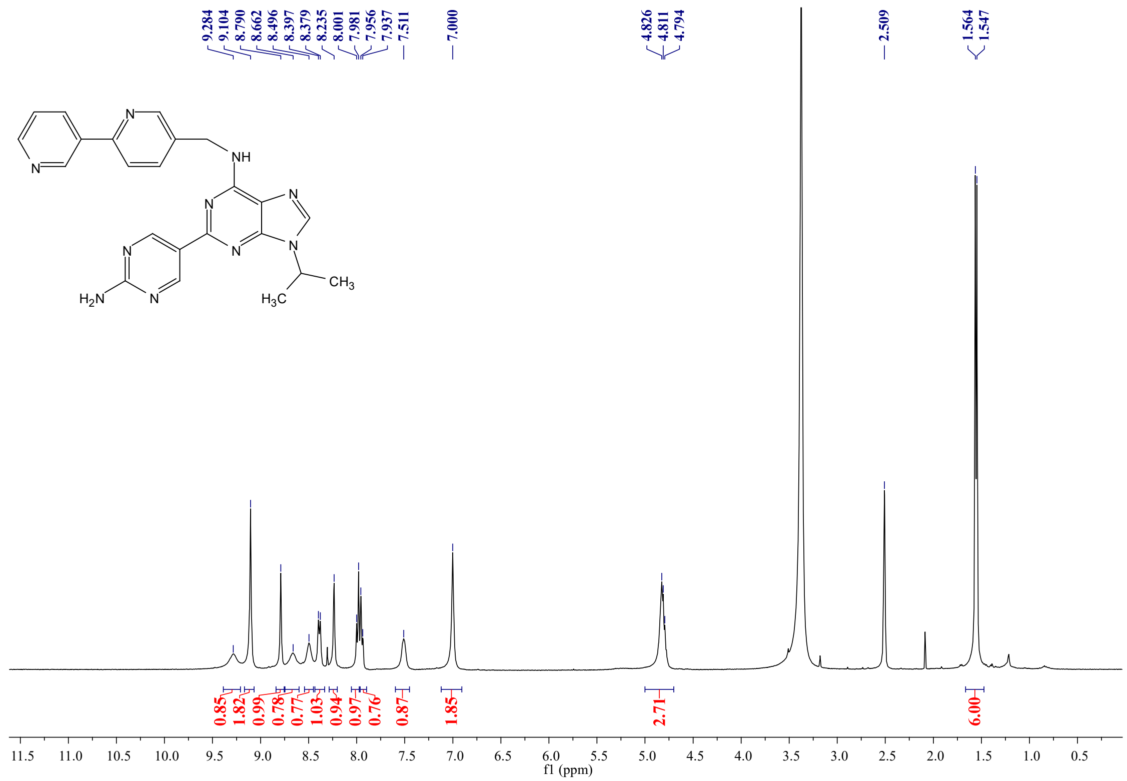
9.284
9.104
8.790
8.662
8.496
8.397
8.379
8.235
8.001
7.981
7.956
7.937
7.511
7.000
4.826
4.811
4.794
2.509
1.564
1.547
0.85
1.82
0.99
0.78
0.77
1.03
0.94
0.97
0.76
0.87
1.85
2.71
6.00
f1 (ppm)

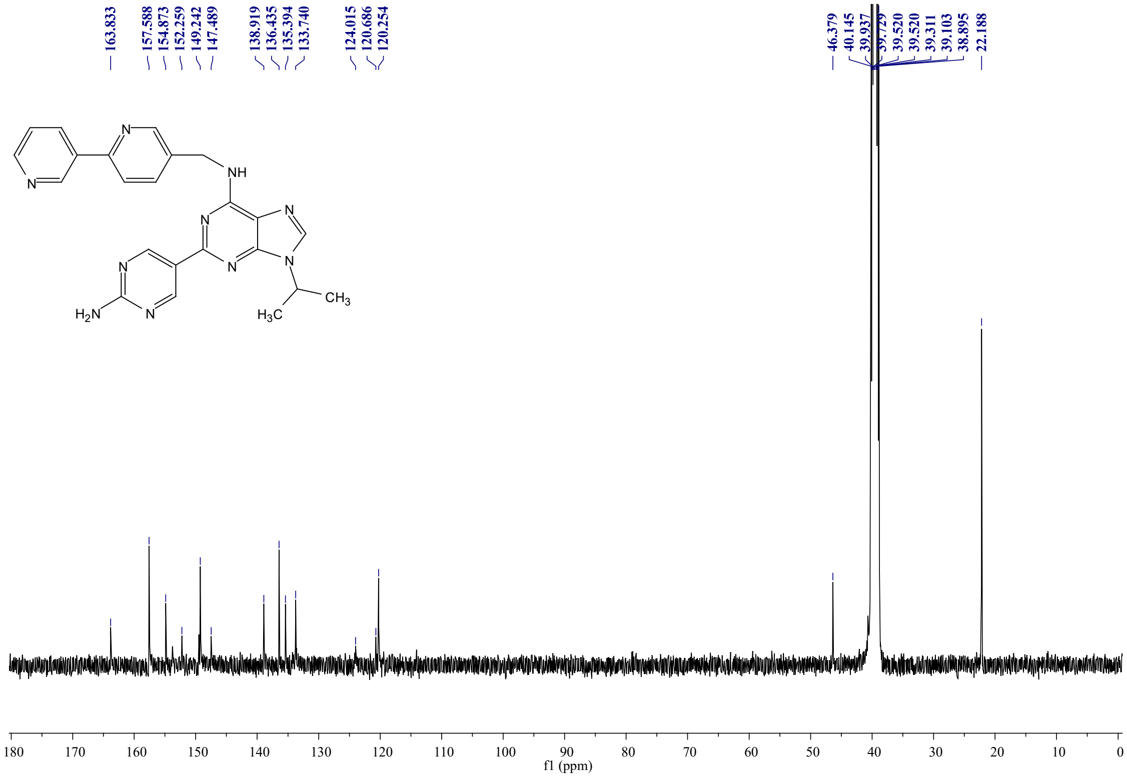
163.833
157.588
154.873
152.259
149.242
147.489
138.919
136.435
135.394
133.740
124.015
120.686
120.254
46.379
40.145
39.937
39.729
39.520
39.520
39.311
39.103
38.895
22.188
f1 (ppm)

18m

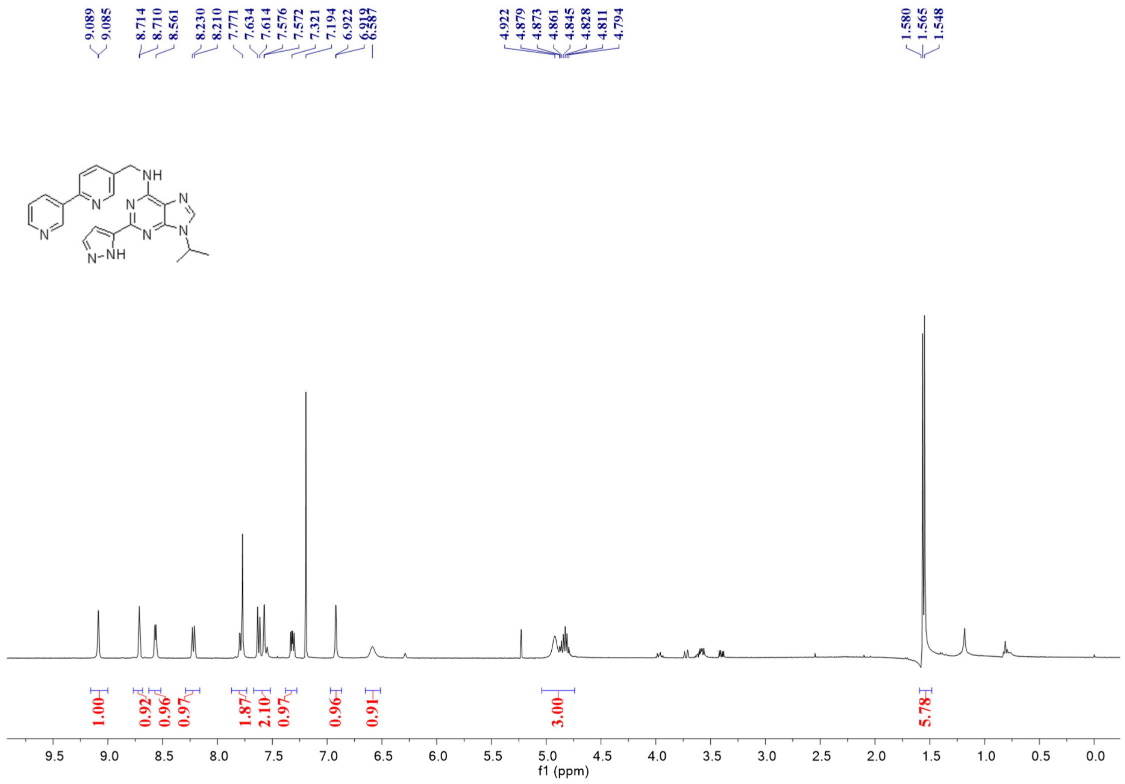

9.089
9.085
8.714
8.710
8.561
8.230
8.210
7.771
7.634
7.614
7.576
7.572
7.321
7.194
6.922
6.919
6.587
4.922
4.879
4.873
4.861
4.845
4.828
4.811
4.794
1.580
1.565
1.548
NH
N
N
N
N
N
N
N-NH
1.00
0.92
0.96
0.97
1.87
2.10
0.97
0.96
0.91
3.00
5.78
9.5
9.0
8.5
8.0
7.5
7.0
6.5
6.0
5.5
5.0
4.5
4.0
3.5
3.0
2.5
2.0
1.5
1.0
0.5
0.0
f1 (ppm)

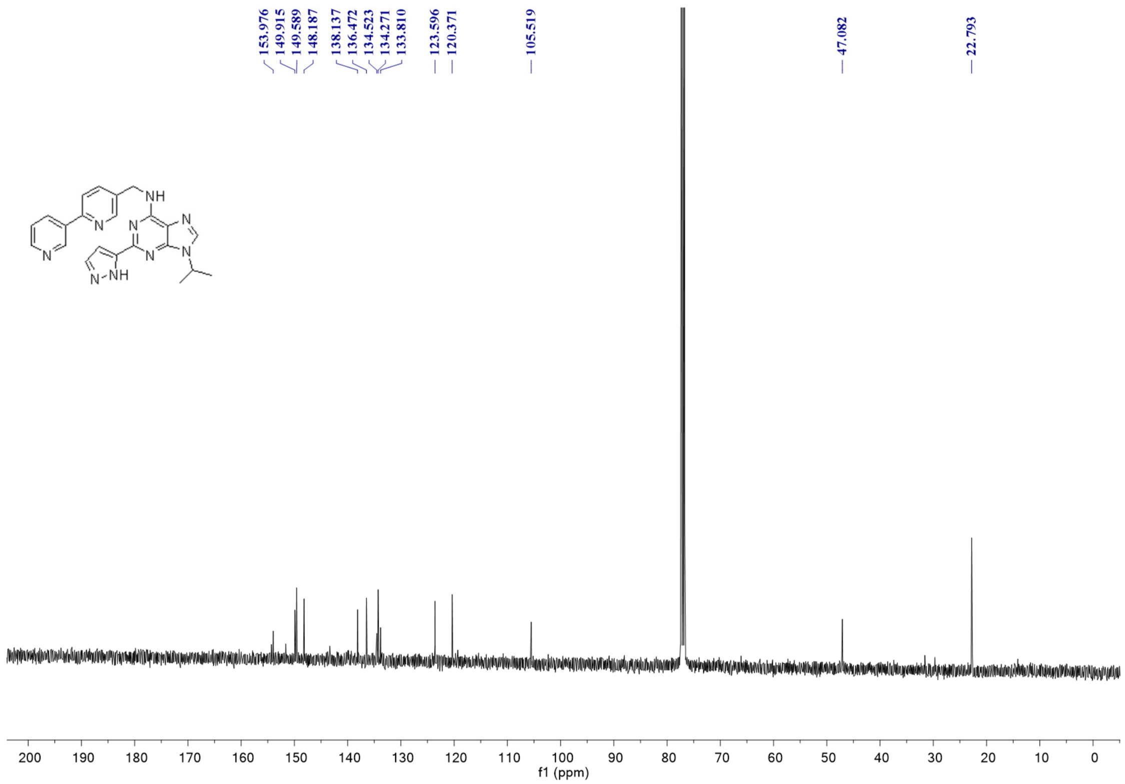

153.976
149.915
149.589
148.187
138.137
136.472
134.523
134.271
133.810
123.596
120.371
105.519
47.082
22.793
NH
N
N
N
N
N
N
N-NH
200
190
180
170
160
150
140
130
120
110
100
90
80
70
60
50
40
30
20
10
0
f1 (ppm)

18n

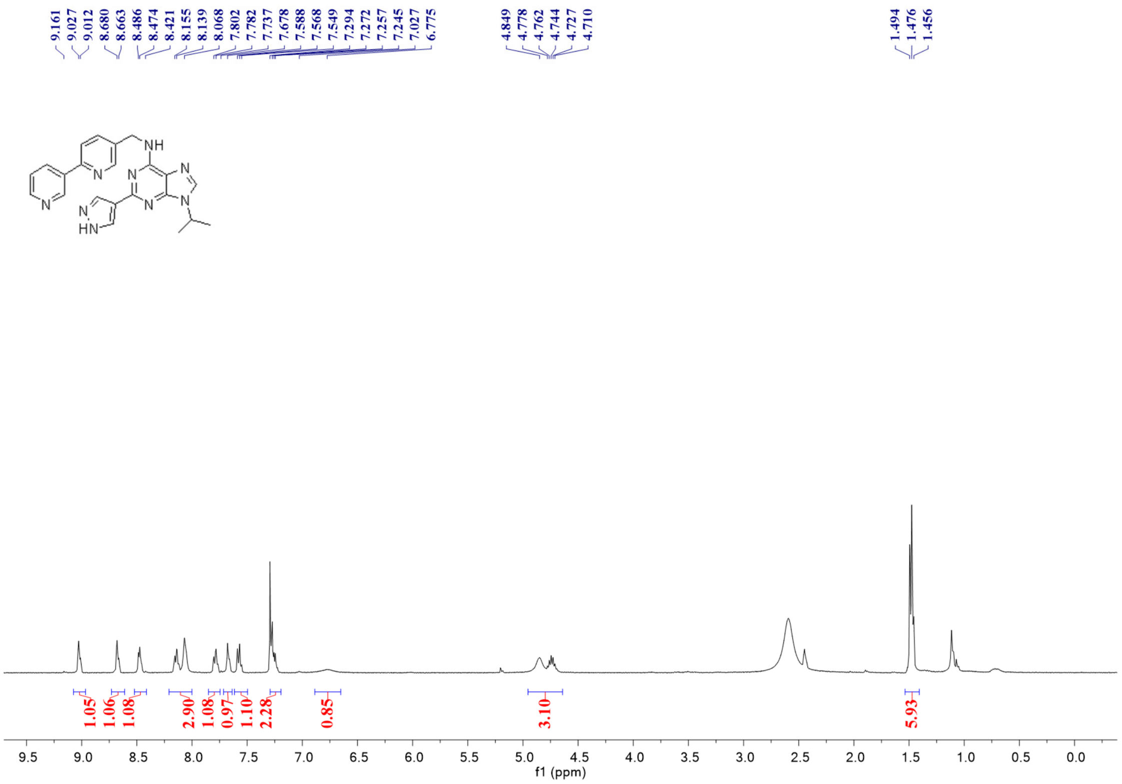

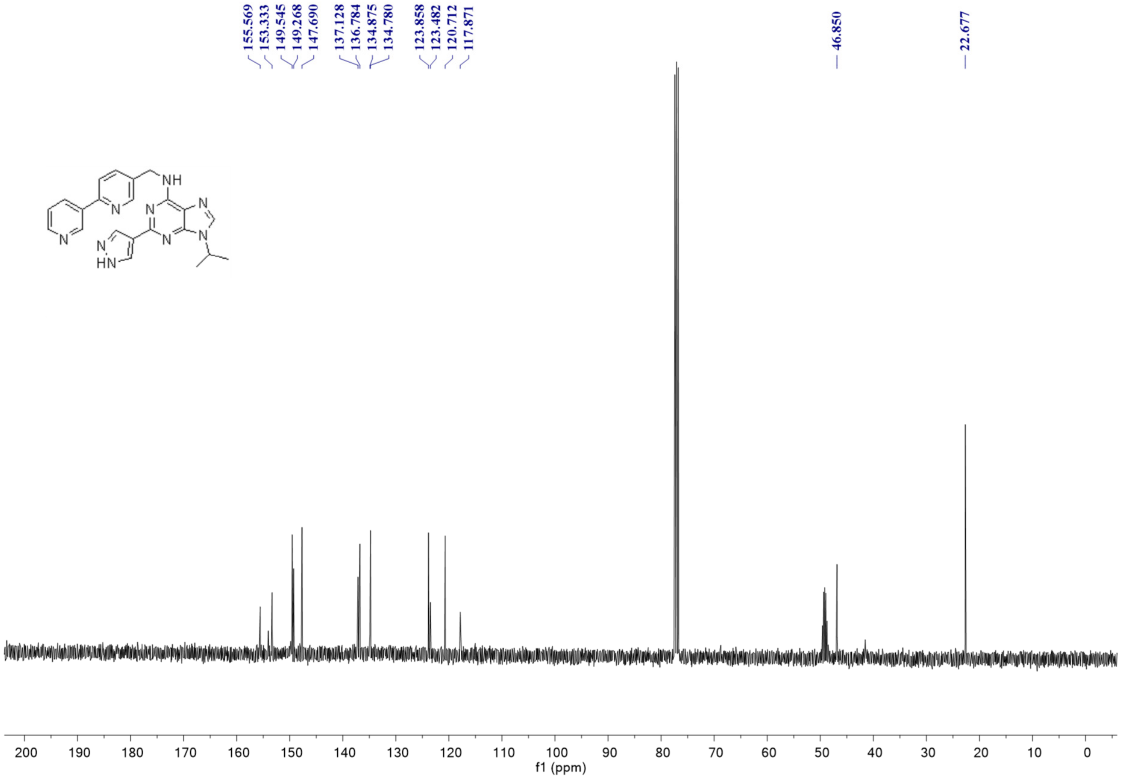

19a

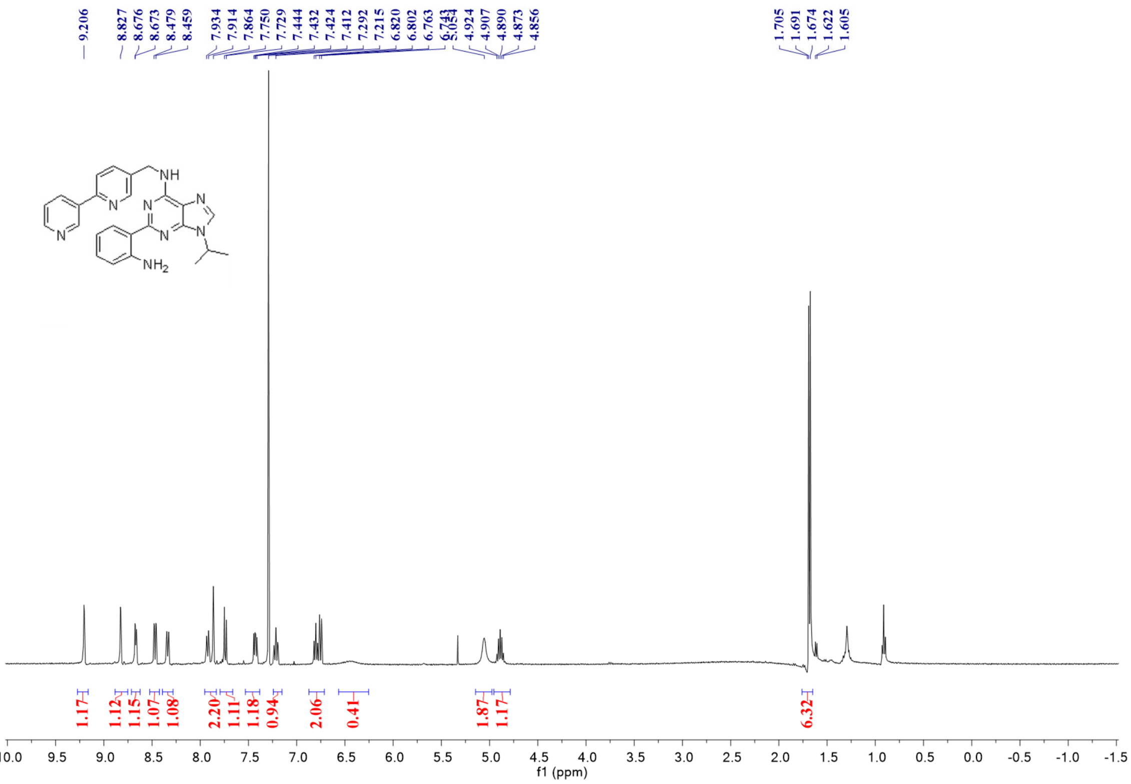
9.206
8.827
8.676
8.673
8.479
8.459
7.934
7.914
7.864
7.750
7.729
7.444
7.432
7.424
7.412
7.292
7.215
6.820
6.802
6.763
4.924
4.907
4.890
4.873
4.856
1.705
1.691
1.674
1.622
1.605
NH
N
N
N
N
N
N
NH_2
1.17
1.12
1.15
1.07
1.08
2.20
1.11
1.18
0.94
2.06
0.41
1.87
1.17
6.32
10.0 9.5 9.0 8.5 8.0 7.5 7.0 6.5 6.0 5.5 5.0 4.5 4.0 3.5 3.0 2.5 2.0 1.5 1.0 0.5 0.0 -0.5 -1.0 -1.5
f1 (ppm)

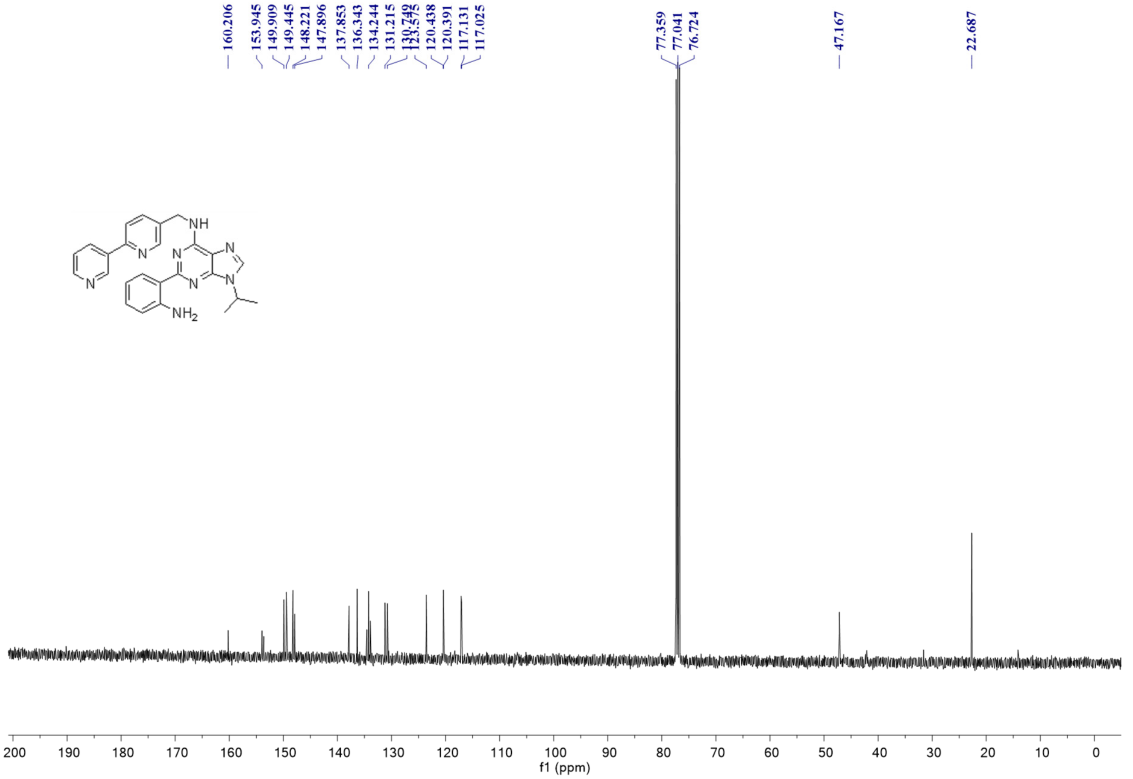
160.206
153.945
149.909
149.445
148.221
147.896
137.853
136.343
134.244
131.215
120.438
120.391
117.131
117.025
77.359
77.041
76.724
47.167
22.687
NH
N
N
N
N
N
N
NH_2
200 190 180 170 160 150 140 130 120 110 100 90 80 70 60 50 40 30 20 10 0
f1 (ppm)

19b

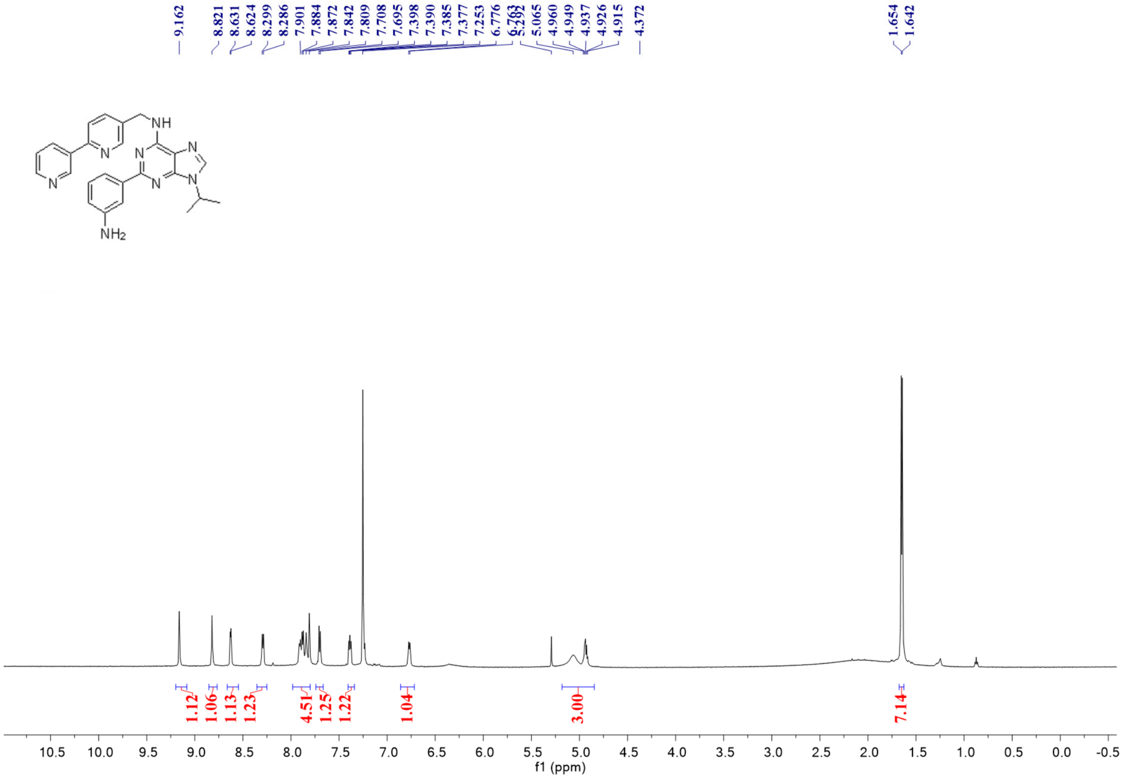
9.162
8.821
8.631
8.624
8.299
8.286
7.901
7.884
7.872
7.842
7.809
7.708
7.695
7.398
7.390
7.385
7.377
7.253
6.776
5.065
4.960
4.949
4.937
4.926
4.915
4.372
1.654
1.642
NH
N
N
N
N
N
N
NH2
1.12
1.06
1.13
1.23
4.51
1.25
1.22
1.04
3.00
7.14
10.5 10.0 9.5 9.0 8.5 8.0 7.5 7.0 6.5 6.0 5.5 5.0 4.5 4.0 3.5 3.0 2.5 2.0 1.5 1.0 0.5 0.0 -0.5
f1 (ppm)

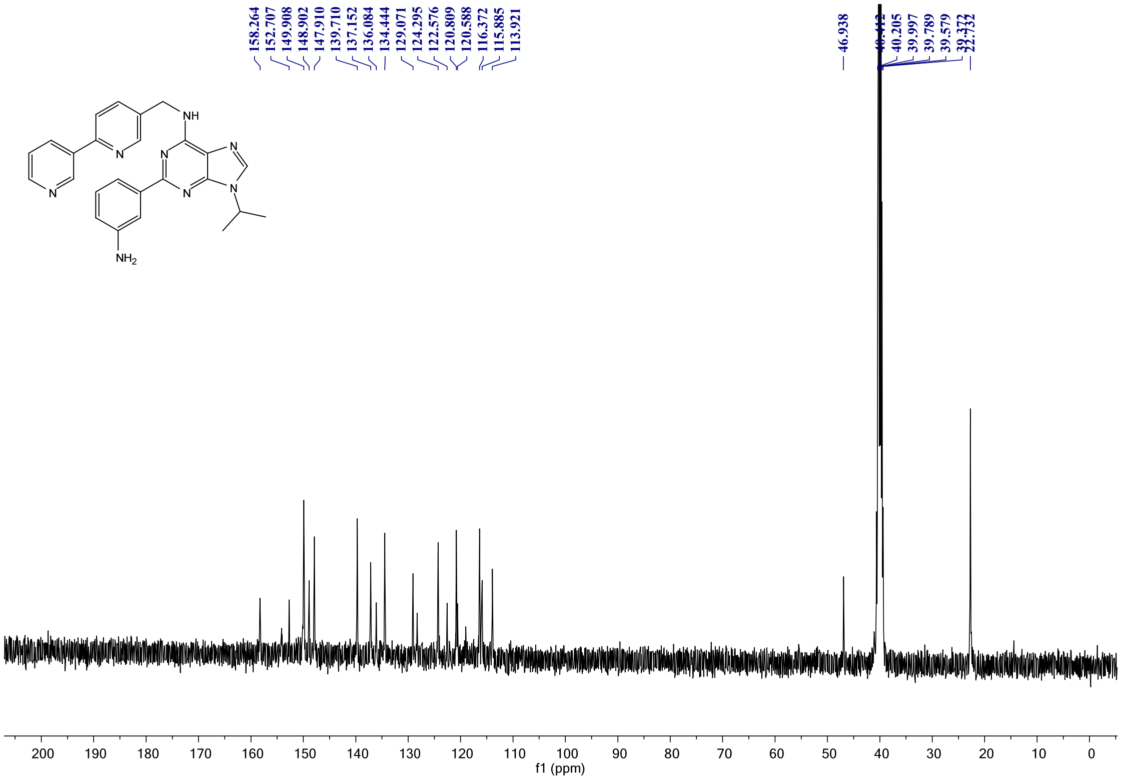
158.264
152.707
149.908
148.902
147.910
139.710
137.152
136.084
134.444
129.071
124.295
122.576
120.809
120.588
116.372
115.885
113.921
46.938
40.205
39.997
39.789
39.579
NH
N
N
N
N
N
N
NH2
200 190 180 170 160 150 140 130 120 110 100 90 80 70 60 50 40 30 20 10 0
f1 (ppm)

19c

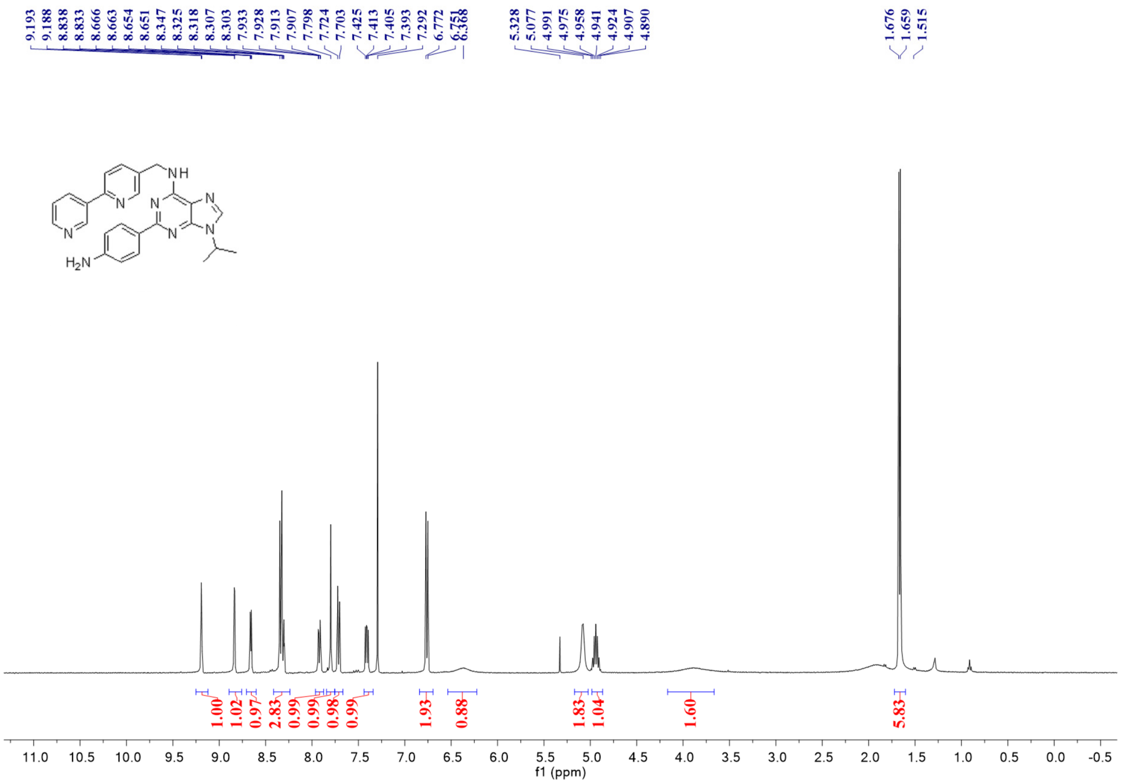
9.193
9.188
8.838
8.833
8.666
8.663
8.654
8.651
8.347
8.325
8.318
8.307
8.303
7.933
7.928
7.913
7.907
7.798
7.724
7.703
7.425
7.413
7.405
7.393
7.292
6.772
6.751
6.368
5.328
5.077
4.991
4.975
4.958
4.941
4.924
4.907
4.890
1.676
1.659
1.515
NH
N
N
N
N
N
N
H2N
1.00
1.02
0.97
2.83
0.99
0.99
0.98
0.99
1.93
0.88
1.83
1.04
1.60
5.83
11.0 10.5 10.0 9.5 9.0 8.5 8.0 7.5 7.0 6.5 6.0 5.5 5.0 4.5 4.0 3.5 3.0 2.5 2.0 1.5 1.0 0.5 0.0 -0.5
f1 (ppm)

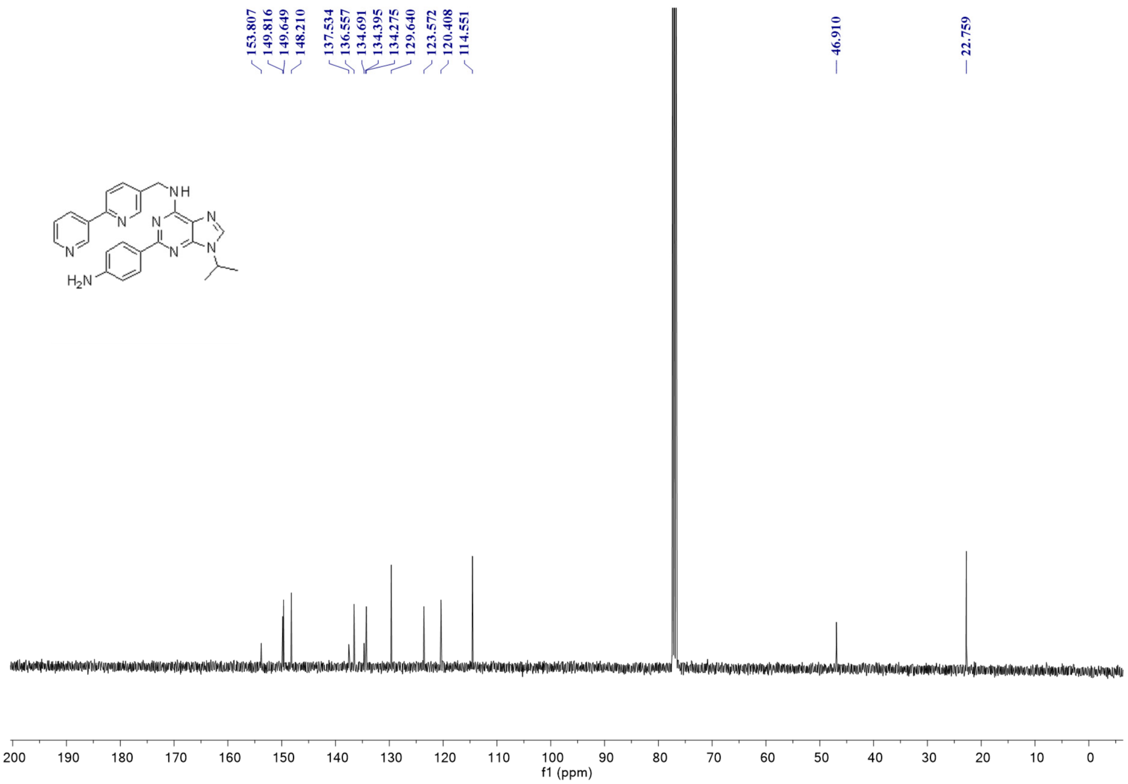
153.807
149.816
149.649
148.210
137.534
136.557
134.691
134.395
134.275
129.640
123.572
120.408
114.551
46.910
22.759
NH
N
N
N
N
N
N
H2N
200 190 180 170 160 150 140 130 120 110 100 90 80 70 60 50 40 30 20 10 0
f1 (ppm)

20a

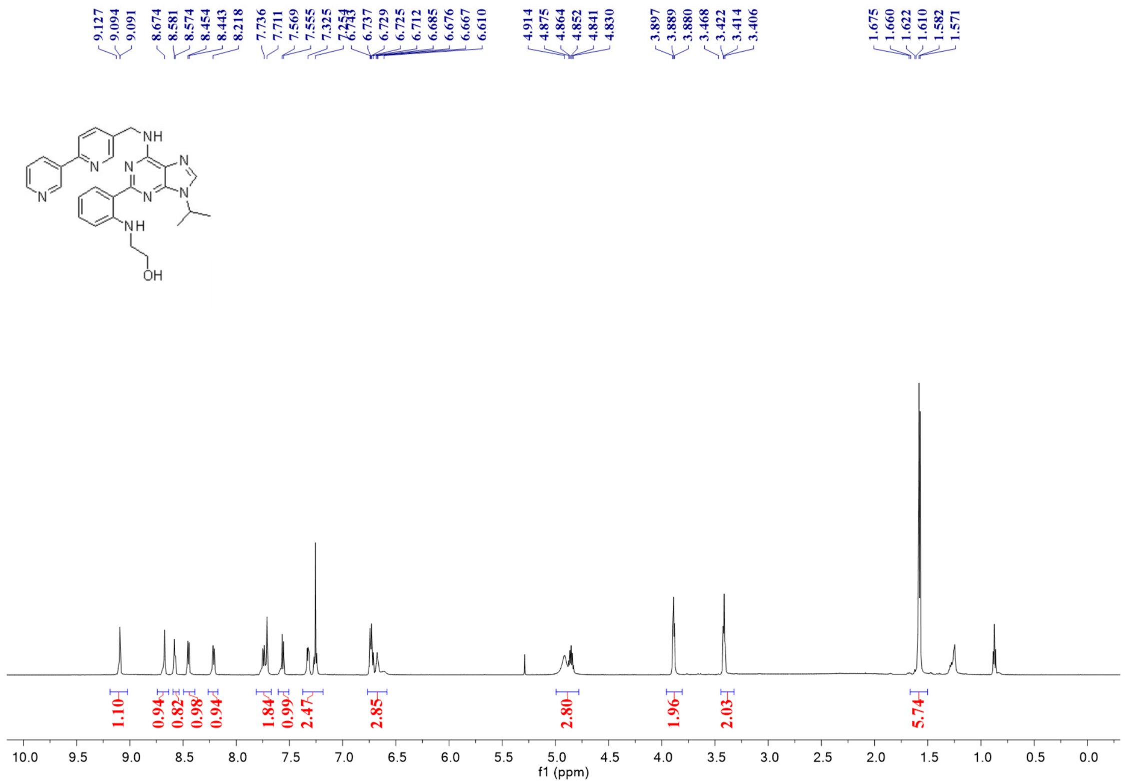

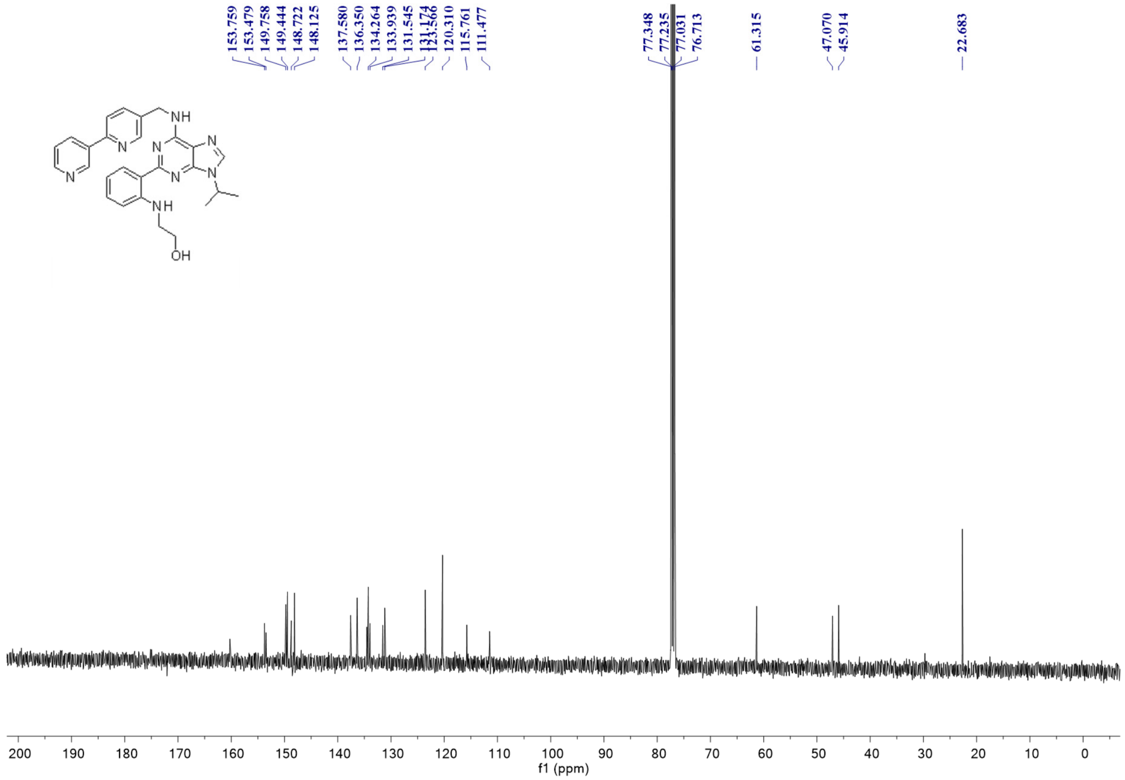

20b

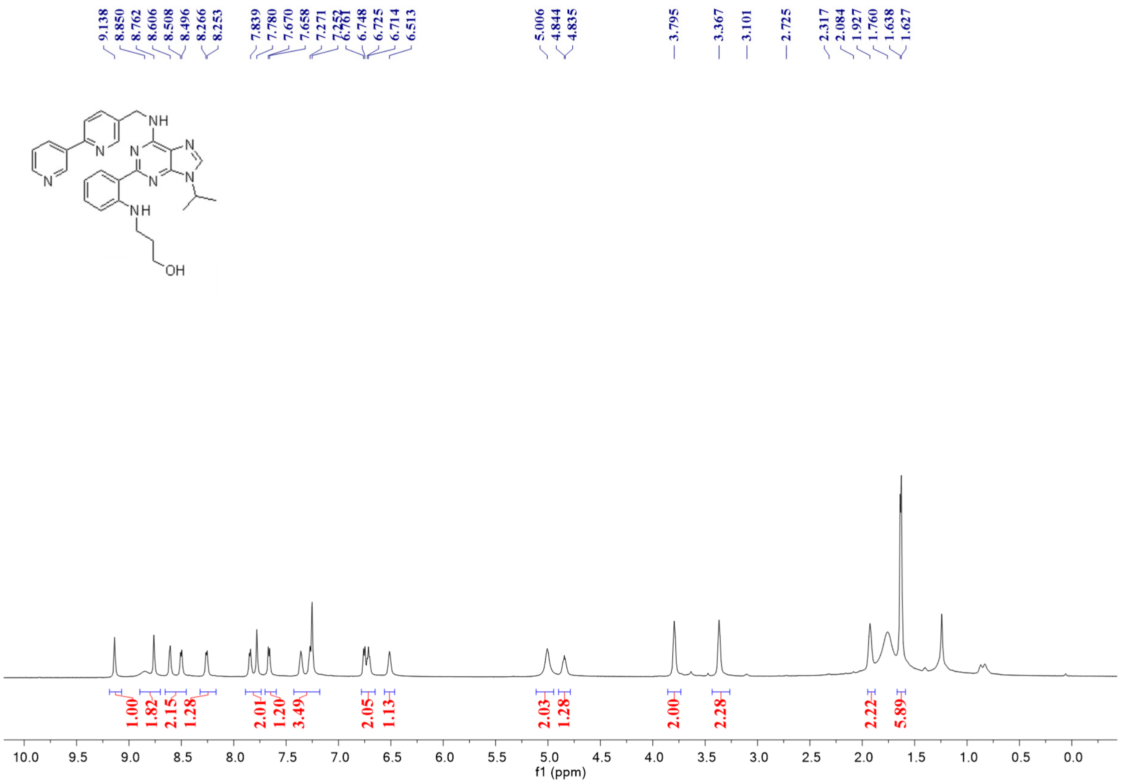

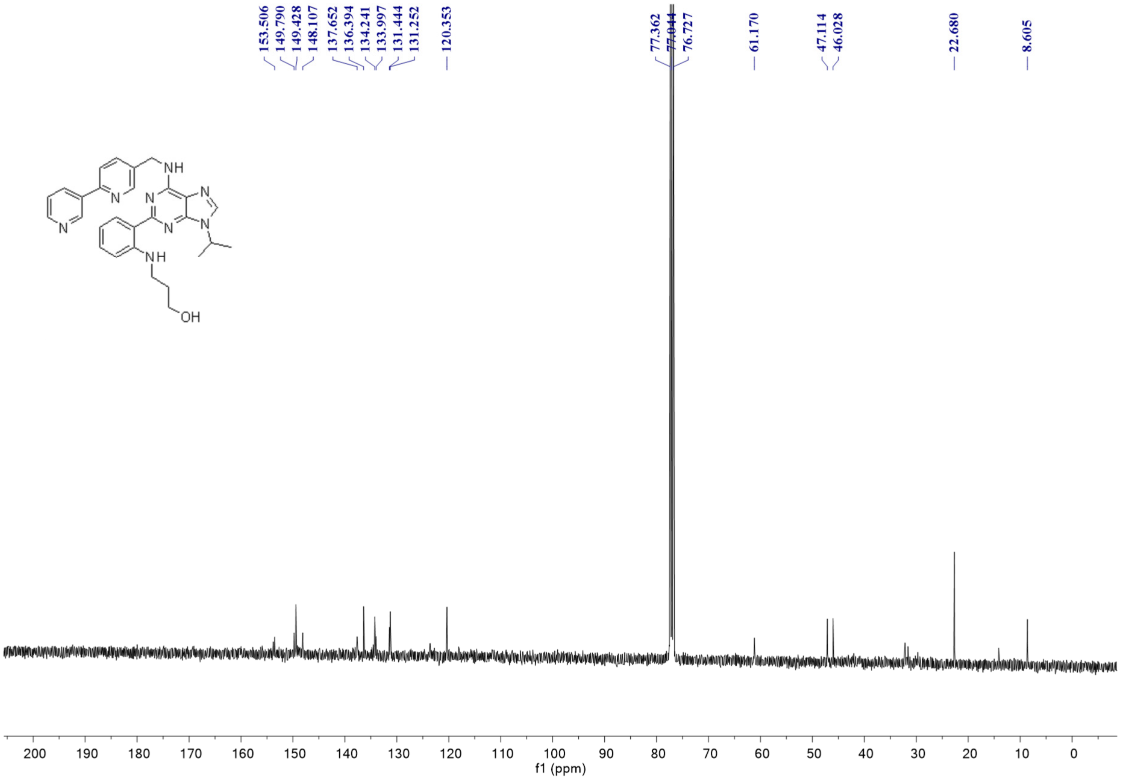

20c

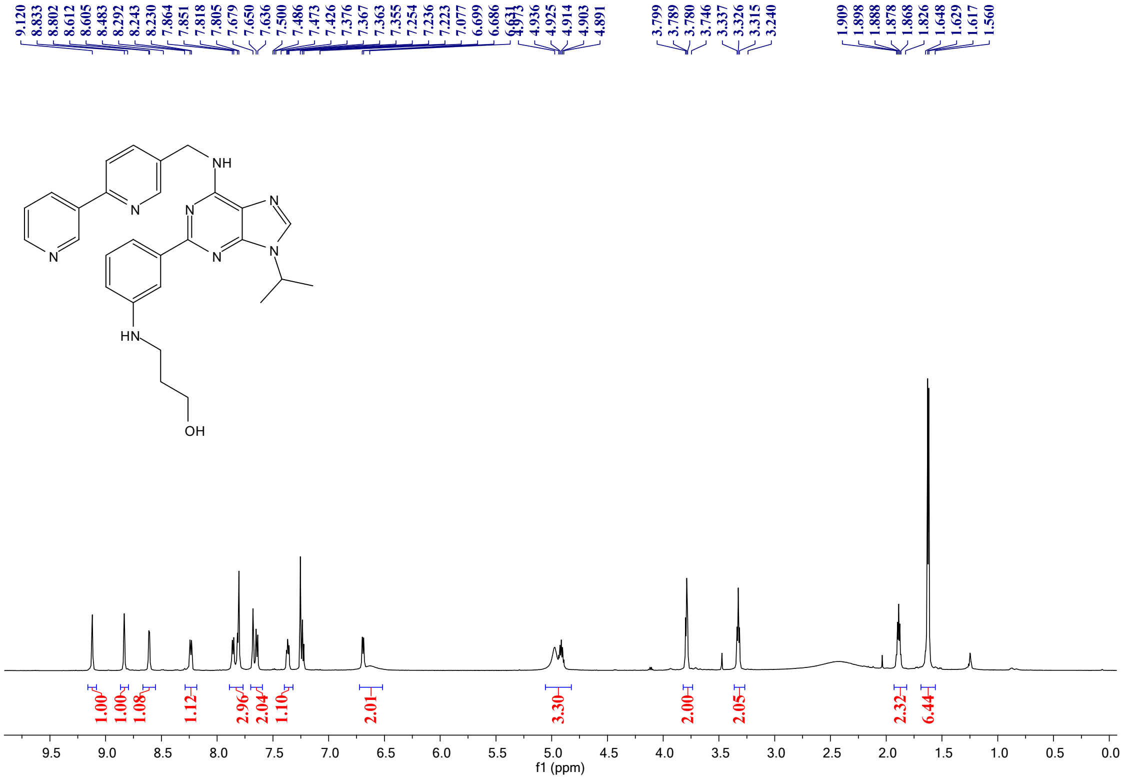
9.120
8.833
8.802
8.612
8.605
8.483
8.292
8.243
8.230
7.864
7.851
7.818
7.805
7.679
7.650
7.636
7.500
7.486
7.473
7.426
7.376
7.367
7.363
7.355
7.254
7.236
7.223
7.077
6.699
6.686
4.973
4.936
4.925
4.914
4.903
4.891
3.799
3.789
3.780
3.746
3.337
3.326
3.315
3.240
1.909
1.898
1.888
1.878
1.868
1.826
1.648
1.629
1.617
1.560
NH
N
N
N
N
N
N
HN
OH
1.00
1.00
1.08
1.12
2.96
2.04
1.10
2.01
3.30
2.00
2.05
2.32
6.44
f1 (ppm)

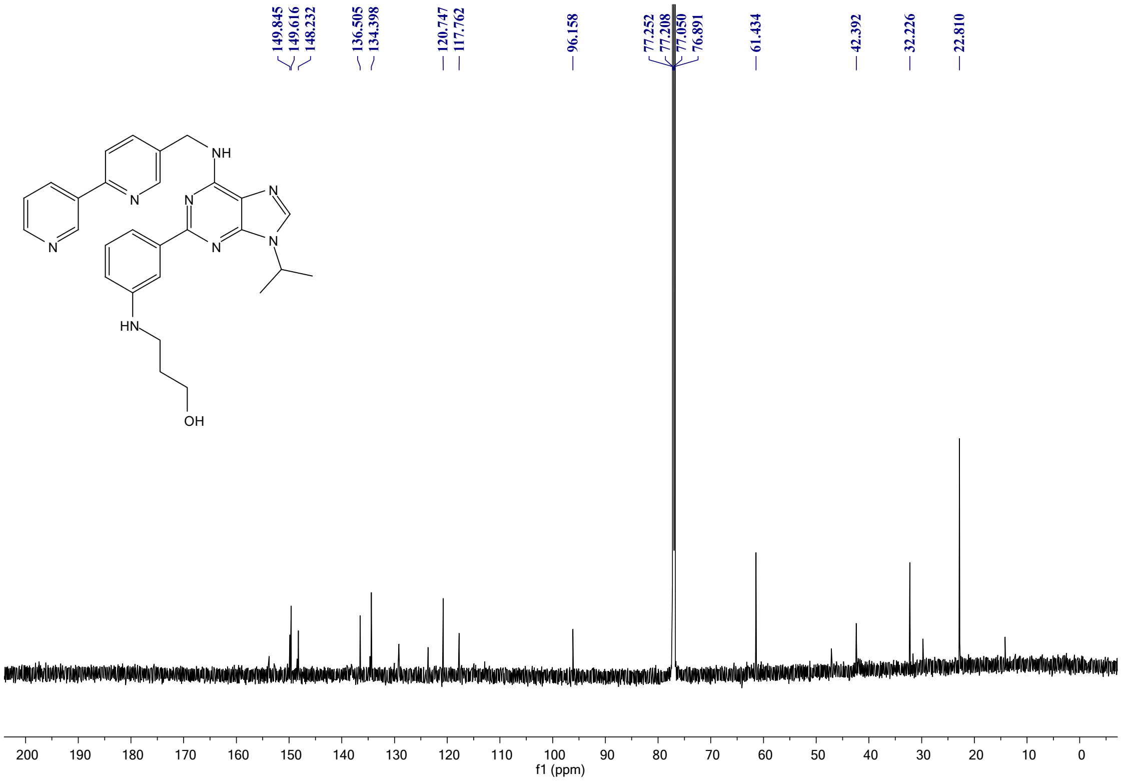
149.845
149.616
148.232
136.505
134.398
120.747
117.762
96.158
77.252
77.208
77.050
76.891
61.434
42.392
32.226
22.810
NH
N
N
N
N
N
N
HN
OH
f1 (ppm)

20d

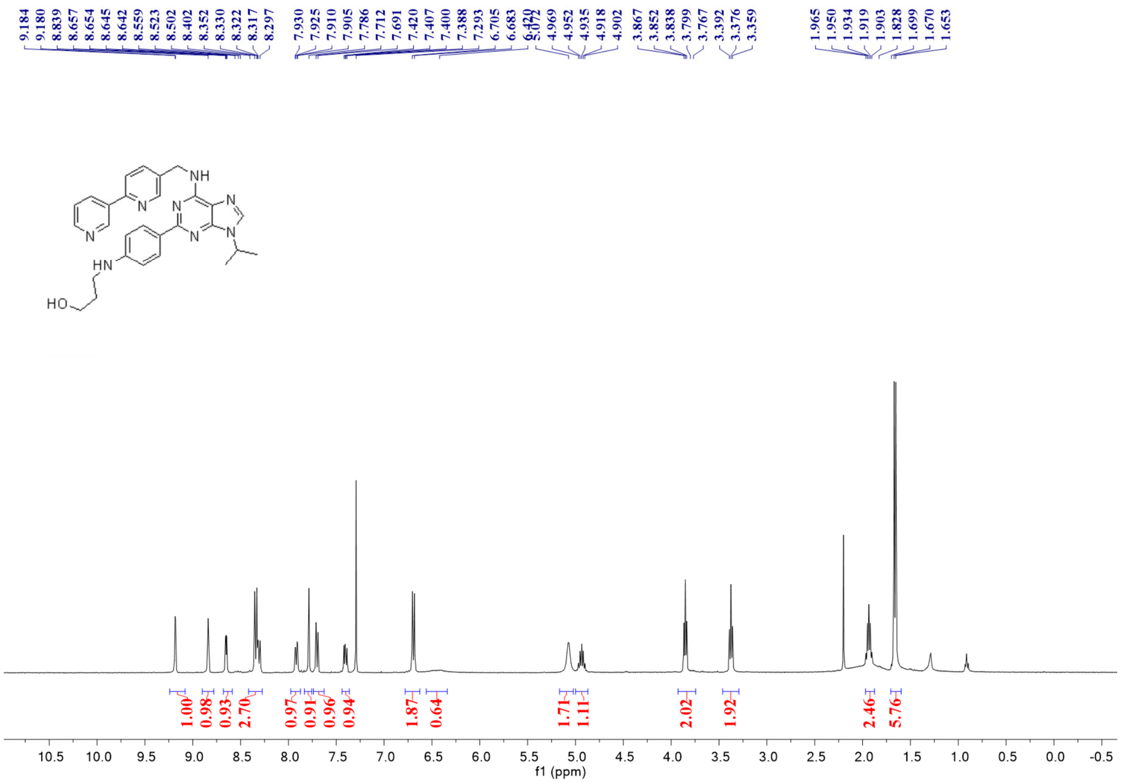
9.184 9.180 8.839 8.657 8.654 8.645 8.642 8.559 8.523 8.502 8.402 8.352 8.330 8.322 8.317 8.297 7.930 7.925 7.910 7.905 7.786 7.712 7.691 7.420 7.407 7.400 7.388 7.293 6.705 6.683 6.420 5.072 4.969 4.952 4.935 4.918 4.902 3.867 3.852 3.838 3.799 3.767 3.392 3.376 3.359 1.965 1.950 1.934 1.919 1.903 1.828 1.699 1.670 1.653
NH
N
HN
HO
1.00 0.98 0.93 2.70 0.97 0.91 0.96 0.94 1.87 0.64 1.71 1.11 2.02 1.92 2.46 5.76
f1 (ppm)

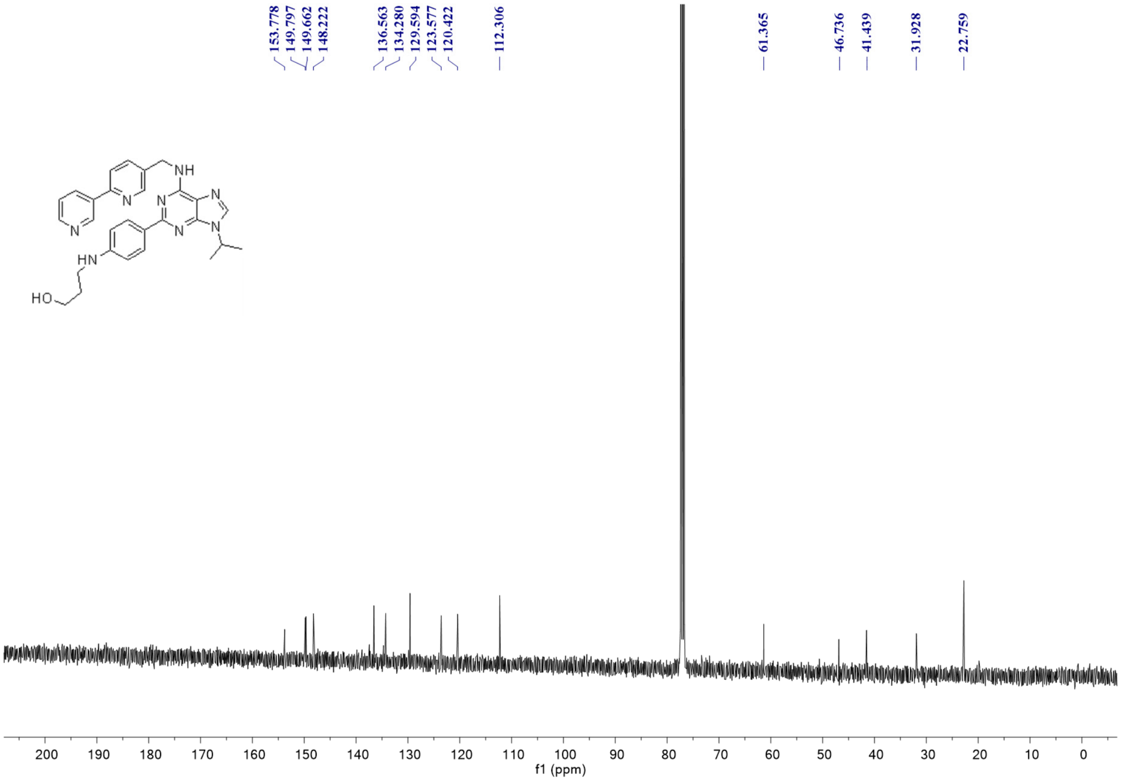
153.778 149.797 149.662 148.222 136.563 134.280 129.594 123.577 120.422 112.306 61.365 46.736 41.439 31.928 22.759
NH
N
HN
HO
f1 (ppm)

21a

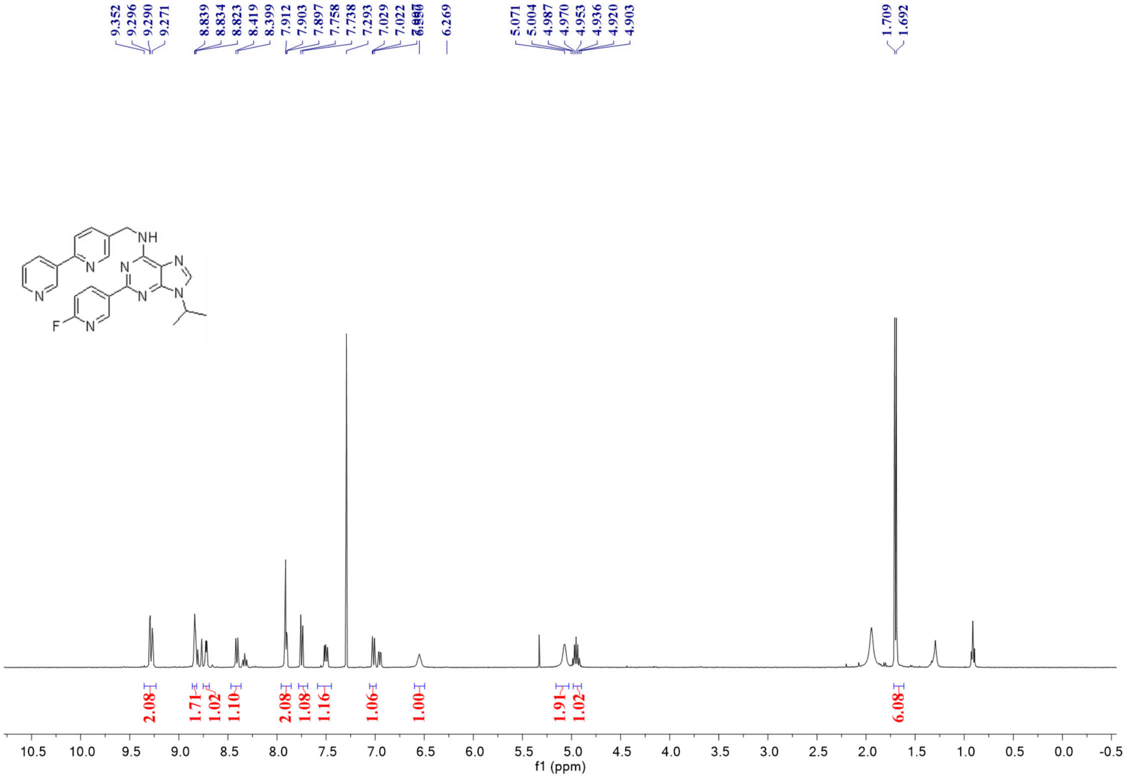

9.352
9.296
9.290
9.271
8.839
8.834
8.823
8.419
8.399
7.912
7.903
7.897
7.758
7.738
7.293
7.029
7.022
6.269
5.071
5.004
4.987
4.970
4.953
4.936
4.920
4.903
1.709
1.692
NH
N
F
2.08
1.71
1.02
1.10
2.08
1.08
1.16
1.06
1.00
1.91
1.02
6.08
f1 (ppm)

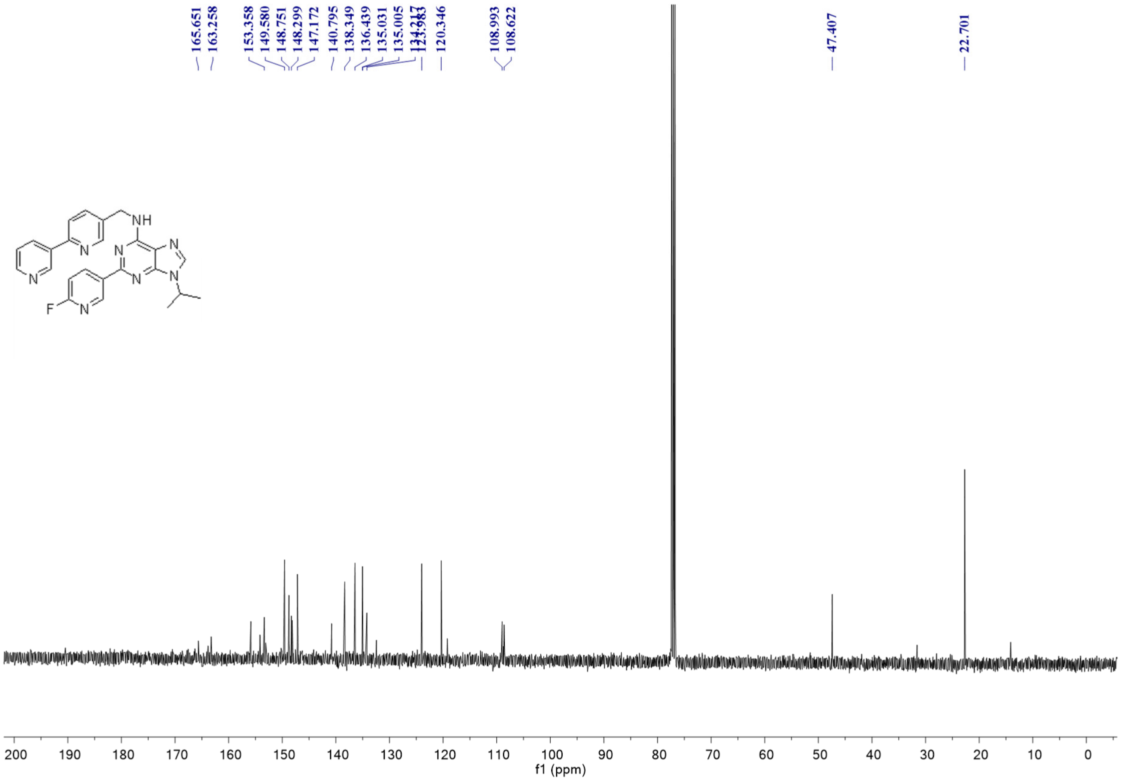

165.651
163.258
153.358
149.580
148.751
148.299
147.172
140.795
138.349
136.439
135.031
135.005
120.346
108.993
108.622
47.407
22.701
NH
N
F
f1 (ppm)

21b

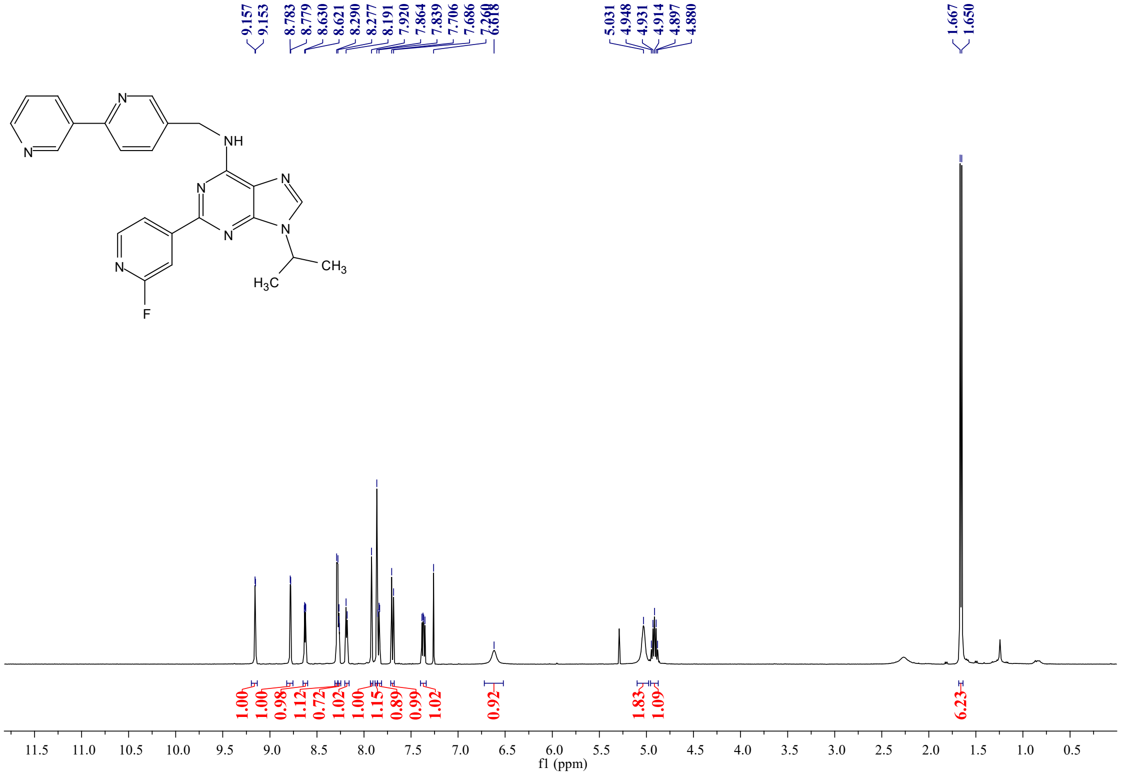
9.157
9.153
8.783
8.779
8.630
8.621
8.290
8.277
8.191
7.920
7.864
7.839
7.706
7.686
7.260
6.618
5.031
4.948
4.931
4.914
4.897
4.880
1.667
1.650
NH
N
F
H_3C
CH_3
1.00
1.00
0.98
1.12
0.72
1.02
1.00
1.15
0.89
0.99
1.02
0.92
1.83
1.09
6.23
f1 (ppm)

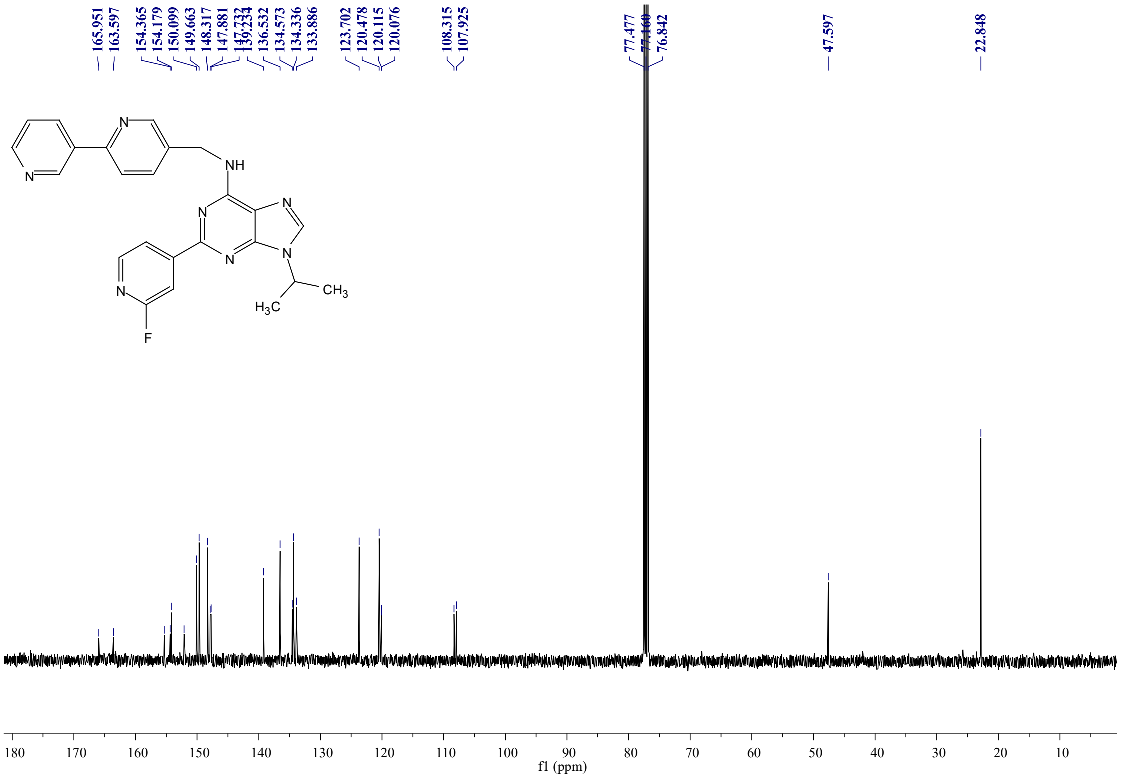
165.951
163.597
154.365
154.179
150.099
149.663
148.317
147.881
147.732
139.234
136.532
134.573
134.336
133.886
123.702
120.478
120.115
120.076
108.315
107.925
77.477
77.160
76.842
47.597
22.848
NH
N
F
H_3C
CH_3
f1 (ppm)

22a

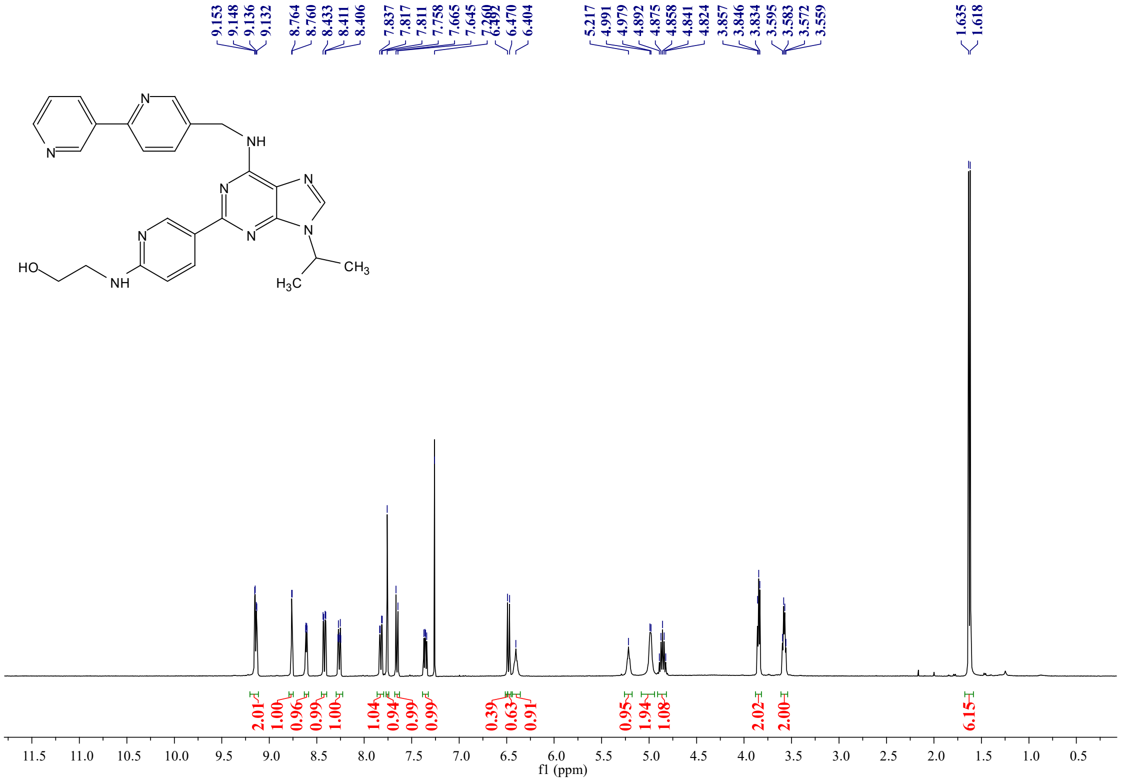
9.153
9.148
9.136
9.132
8.764
8.760
8.433
8.411
8.406
7.837
7.817
7.811
7.758
7.665
7.645
6.470
6.404
5.217
4.991
4.979
4.892
4.875
4.858
4.841
4.824
3.857
3.846
3.834
3.595
3.583
3.572
3.559
1.635
1.618
N
NH
N
N
N
N
N
HO
NH
CH_3
H_3C
2.01
1.00
0.96
0.99
1.00
1.04
0.94
0.99
0.99
0.39
0.63
0.91
0.95
1.94
1.08
2.02
2.00
6.15
11.5 11.0 10.5 10.0 9.5 9.0 8.5 8.0 7.5 7.0 6.5 6.0 5.5 5.0 4.5 4.0 3.5 3.0 2.5 2.0 1.5 1.0 0.5
f1 (ppm)

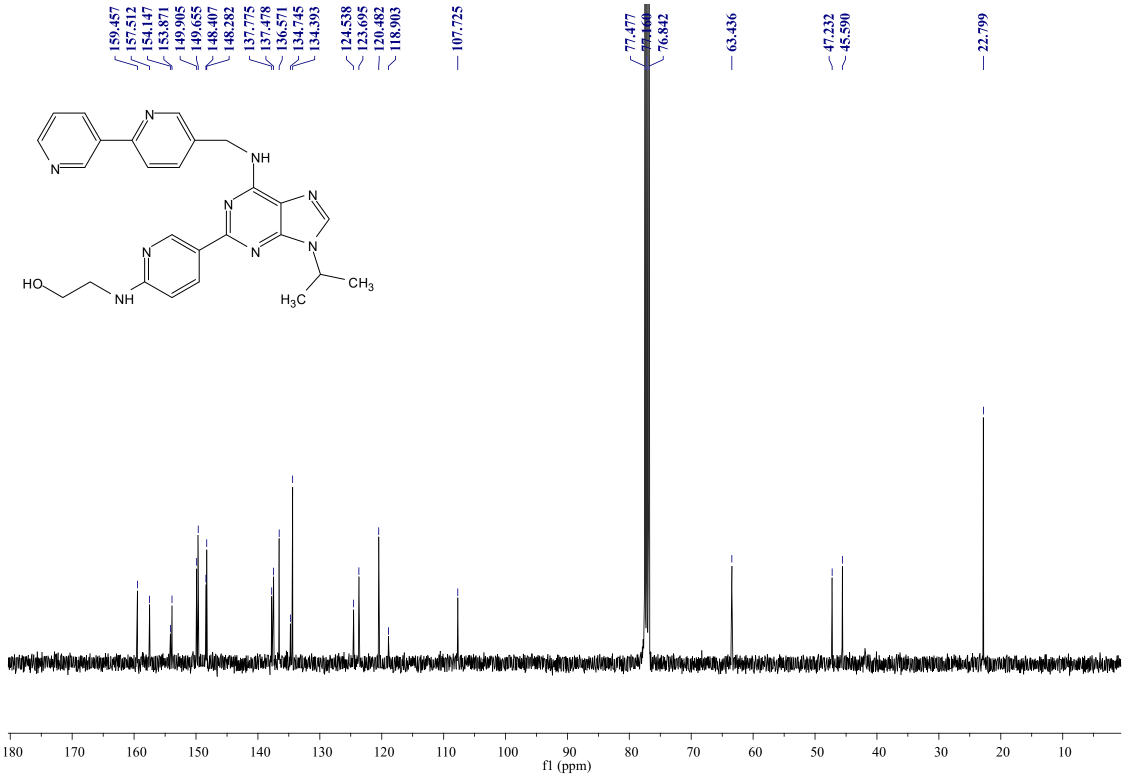
159.457
157.512
154.147
153.871
149.905
149.655
148.407
148.282
137.775
137.478
136.571
134.745
134.393
124.538
123.695
120.482
118.903
107.725
77.477
76.842
63.436
47.232
45.590
22.799
N
NH
N
N
N
N
N
HO
NH
CH_3
H_3C
180 170 160 150 140 130 120 110 100 90 80 70 60 50 40 30 20 10
f1 (ppm)

22b

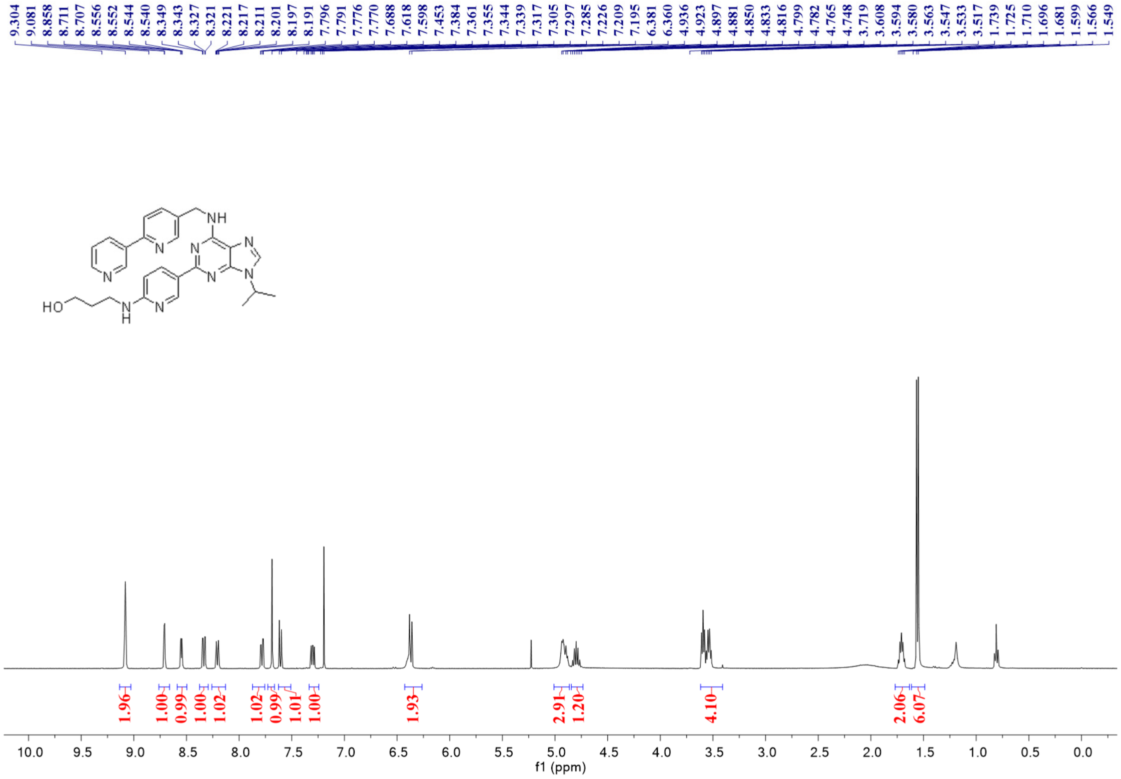

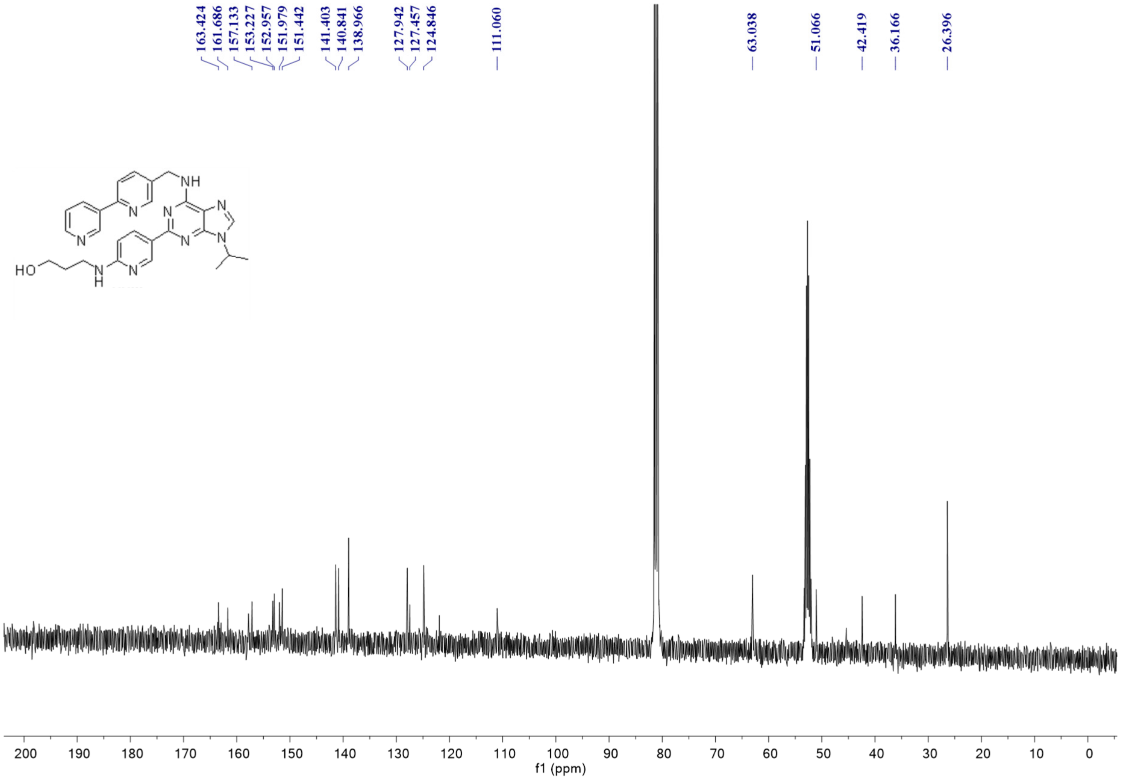

22c

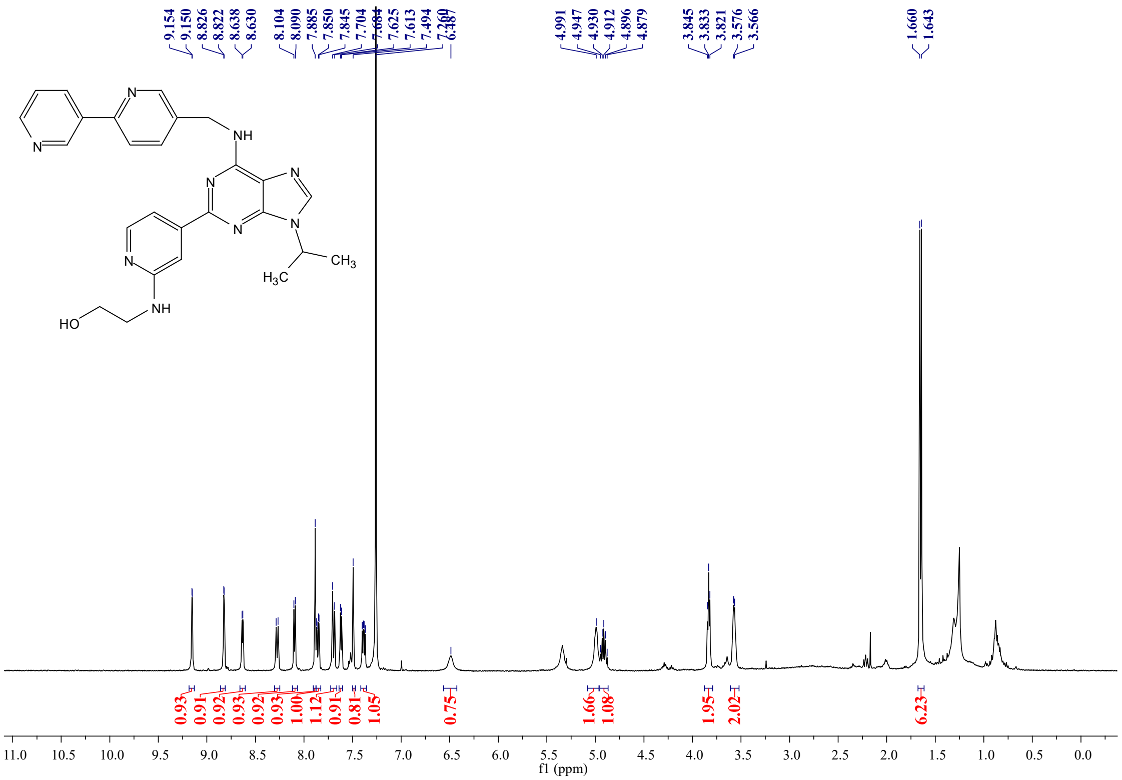

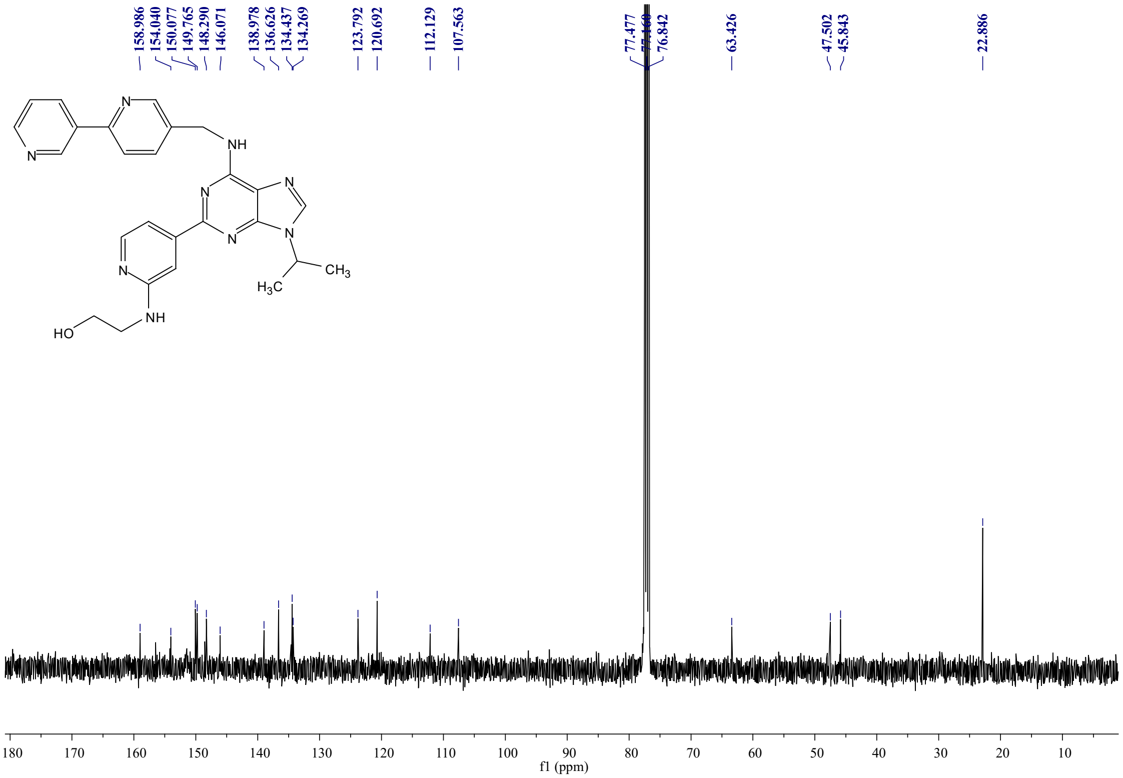

22d

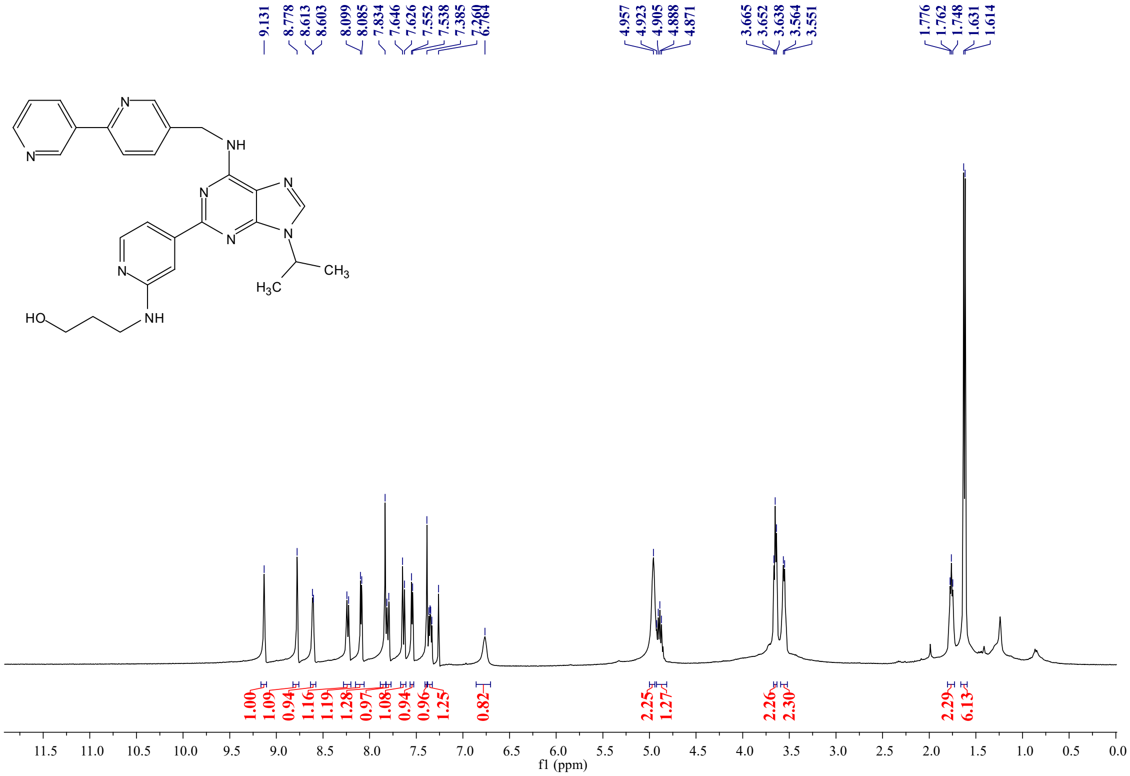
9.131
8.778
8.613
8.603
8.099
8.085
7.834
7.646
7.626
7.552
7.538
7.385
7.260
6.784
4.957
4.923
4.905
4.888
4.871
3.665
3.652
3.638
3.564
3.551
1.776
1.762
1.748
1.631
1.614
NH
N
N
N
N
N
N
N
CH3
H3C
HO
NH
1.00
1.09
0.94
1.16
1.19
1.28
0.97
1.08
0.94
0.96
1.25
0.82
2.25
1.27
2.26
2.30
2.29
6.13
11.5 11.0 10.5 10.0 9.5 9.0 8.5 8.0 7.5 7.0 6.5 6.0 5.5 5.0 4.5 4.0 3.5 3.0 2.5 2.0 1.5 1.0 0.5 0.0
f1 (ppm)

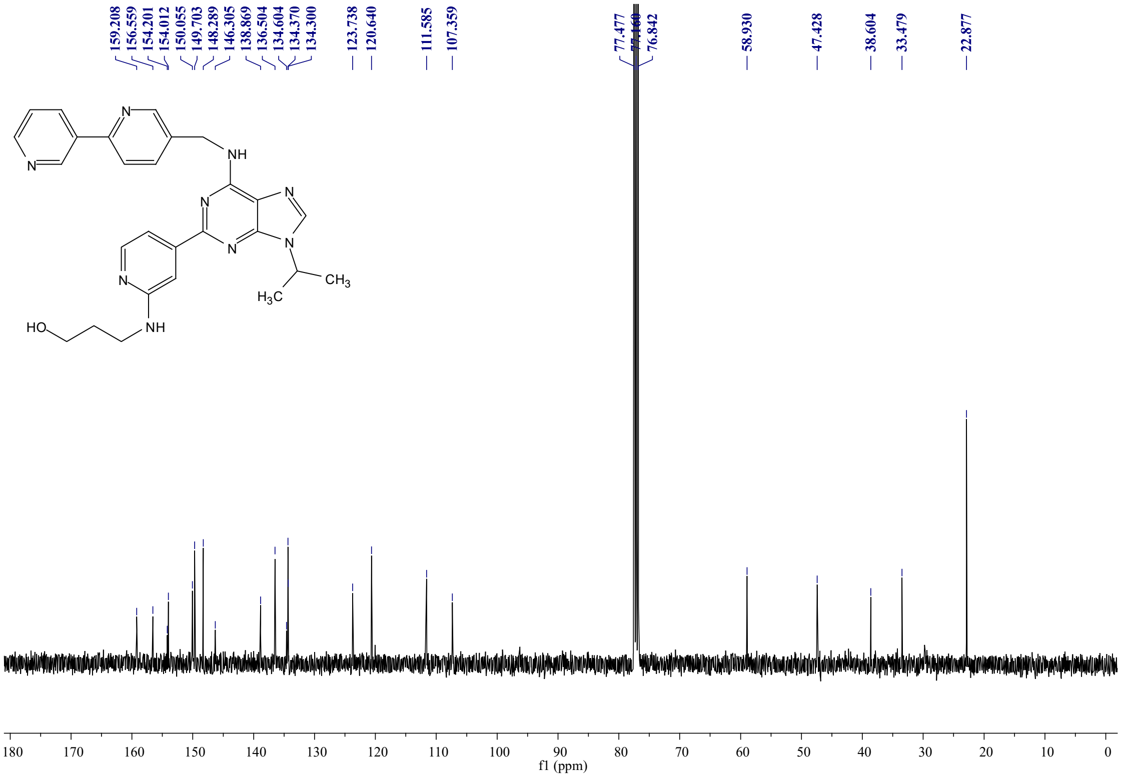
159.208
156.559
154.201
154.012
150.055
149.703
148.289
146.305
138.869
136.504
134.604
134.370
134.300
123.738
120.640
111.585
107.359
77.477
76.842
58.930
47.428
38.604
33.479
22.877
NH
N
N
N
N
N
N
N
CH3
H3C
HO
NH
180 170 160 150 140 130 120 110 100 90 80 70 60 50 40 30 20 10 0
f1 (ppm)

24

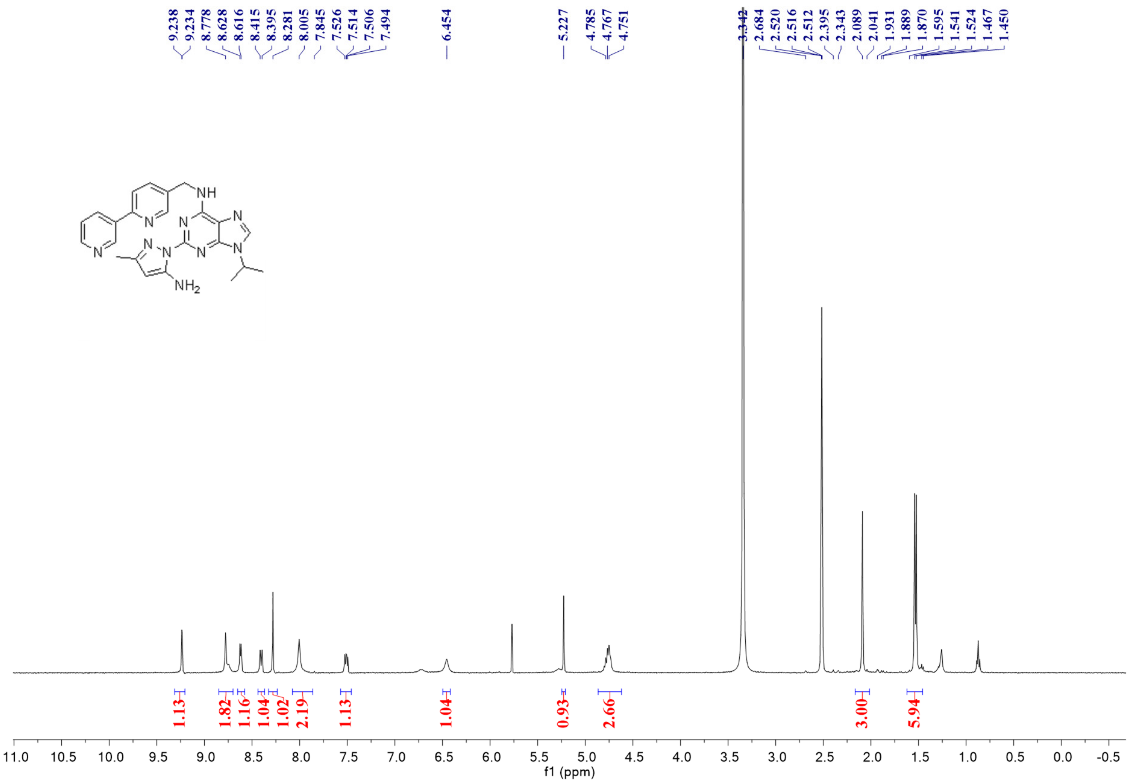

9.238
9.234
8.778
8.628
8.616
8.415
8.395
8.281
8.005
7.845
7.526
7.514
7.506
7.494
6.454
5.227
4.785
4.767
4.751
3.342
2.684
2.520
2.516
2.512
2.395
2.343
2.089
2.041
1.931
1.889
1.870
1.595
1.541
1.524
1.467
1.450
NH
N
N
N
N
N
N
N
N
NH2
1.13
1.82
1.16
1.04
1.02
2.19
1.13
1.04
0.93
2.66
3.00
5.94
11.0 10.5 10.0 9.5 9.0 8.5 8.0 7.5 7.0 6.5 6.0 5.5 5.0 4.5 4.0 3.5 3.0 2.5 2.0 1.5 1.0 0.5 0.0 -0.5
f1 (ppm)

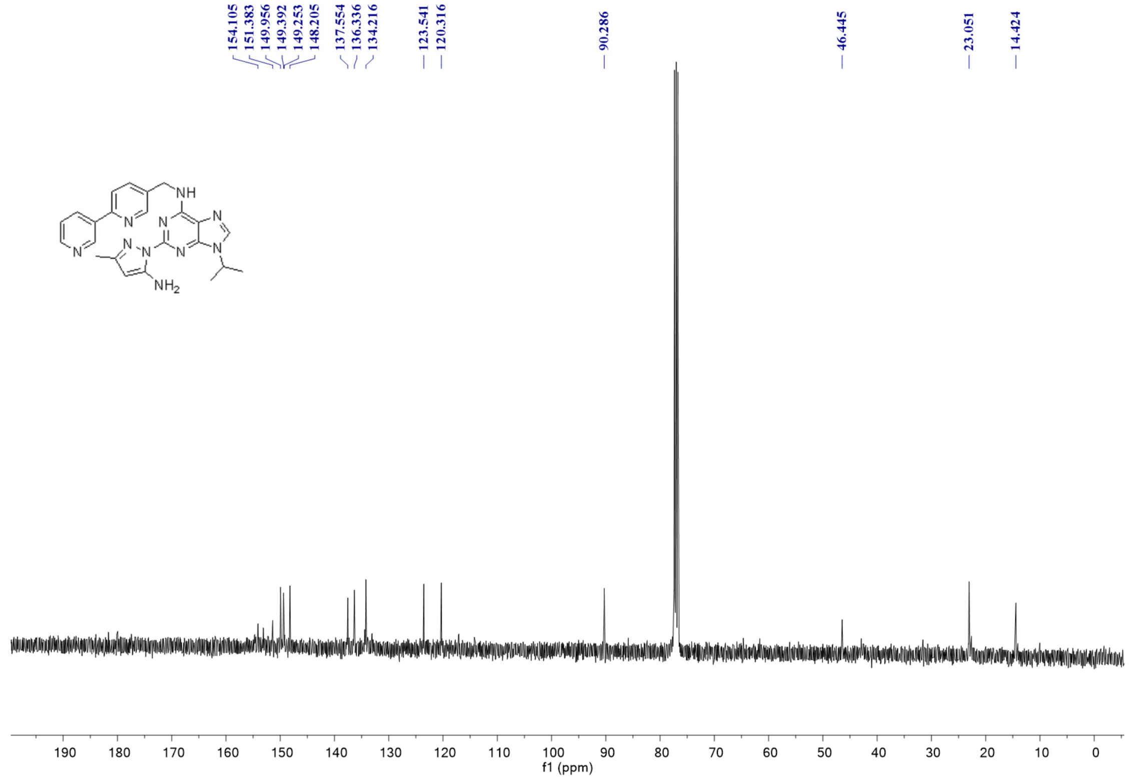

154.105
151.383
149.956
149.392
149.253
148.205
137.554
136.336
134.216
123.541
120.316
90.286
46.445
23.051
14.424
NH
N
N
N
N
N
N
N
N
NH2
190 180 170 160 150 140 130 120 110 100 90 80 70 60 50 40 30 20 10 0
f1 (ppm)

26a

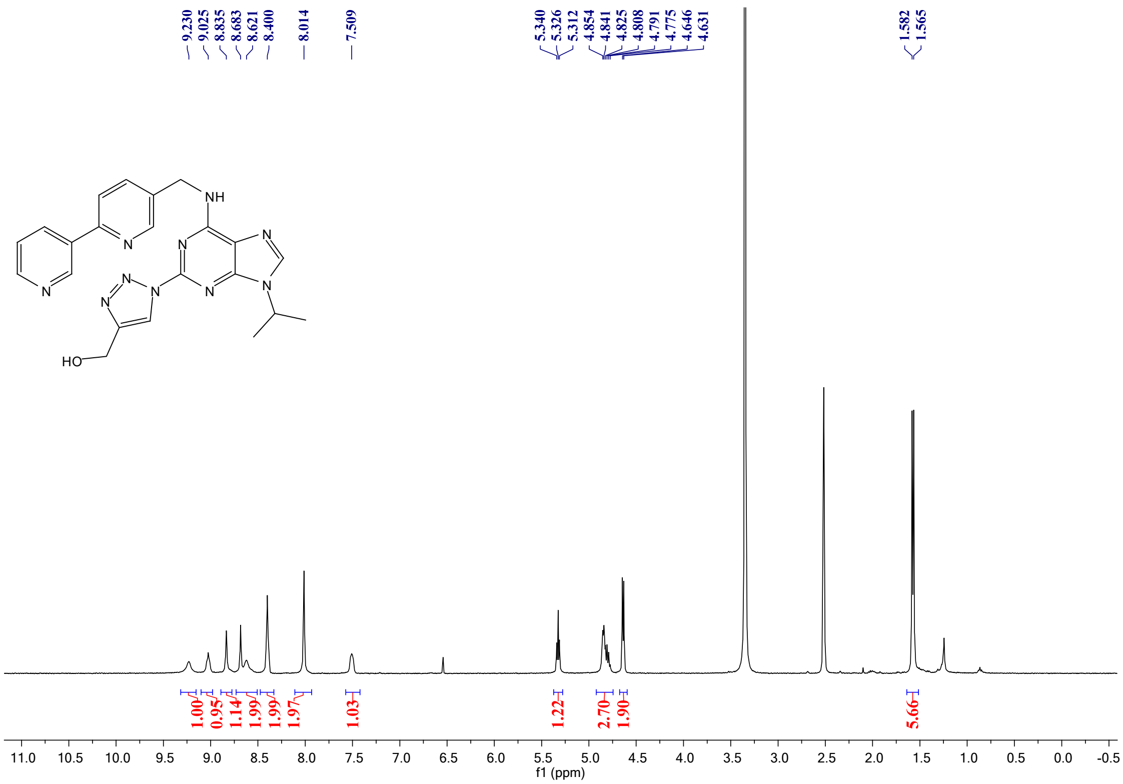
9.230
9.025
8.835
8.683
8.621
8.400
8.014
7.509
5.340
5.326
5.312
4.854
4.841
4.825
4.808
4.791
4.775
4.646
4.631
1.582
1.565
NH
N
N
N
N
N
N
N
N
N
HO
1.00
0.95
1.14
1.99
1.99
1.97
1.03
1.22
2.70
1.90
5.66
11.0 10.5 10.0 9.5 9.0 8.5 8.0 7.5 7.0 6.5 6.0 5.5 5.0 4.5 4.0 3.5 3.0 2.5 2.0 1.5 1.0 0.5 0.0 -0.5
f1 (ppm)

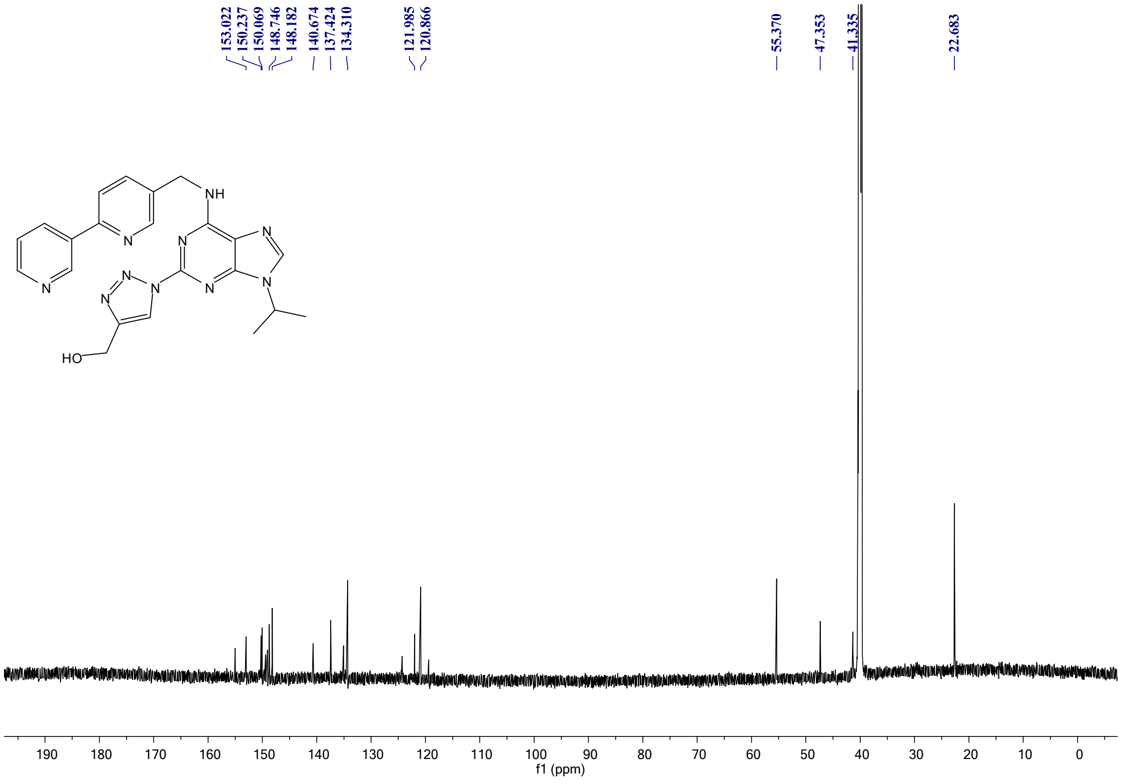
153.022
150.237
150.069
148.746
148.182
140.674
137.424
134.310
121.985
120.866
55.370
47.353
41.335
22.683
NH
N
N
N
N
N
N
N
N
N
HO
190 180 170 160 150 140 130 120 110 100 90 80 70 60 50 40 30 20 10 0
f1 (ppm)

26b

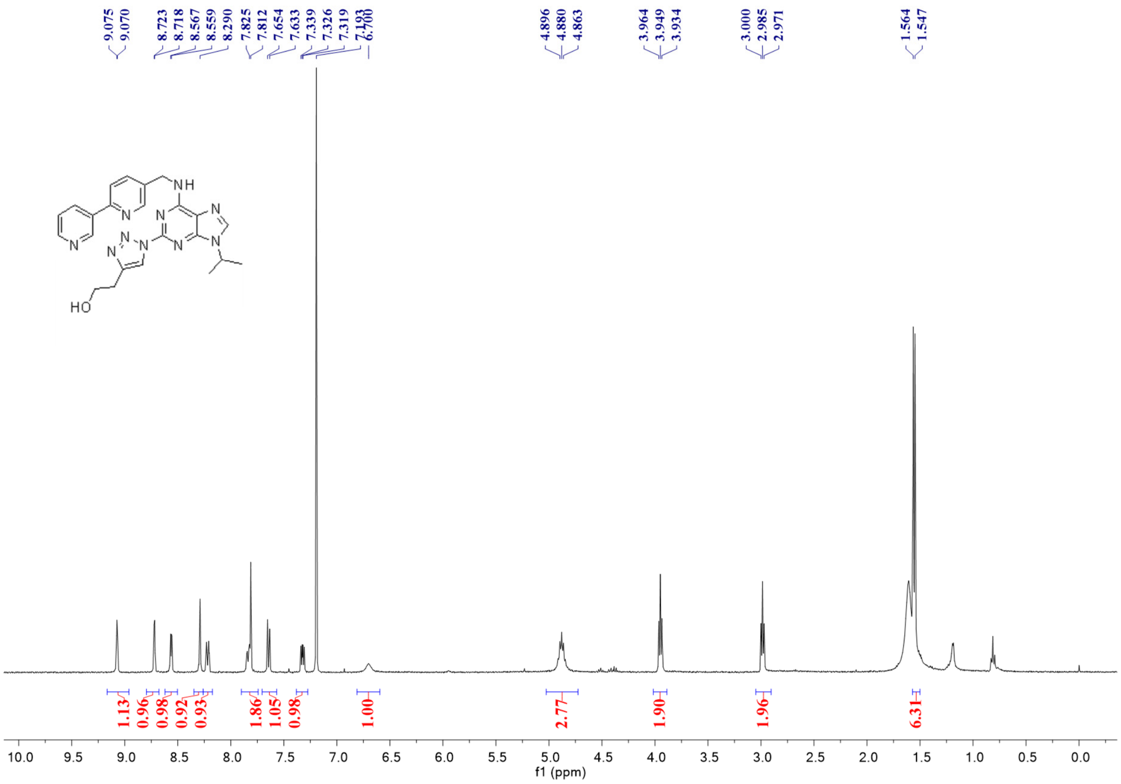
9.075
9.070
8.723
8.718
8.567
8.559
8.290
7.825
7.812
7.654
7.633
7.339
7.326
7.319
7.193
6.700
4.896
4.880
4.863
3.964
3.949
3.934
3.000
2.985
2.971
1.564
1.547
1.13
0.96
0.98
0.92
0.93
1.86
1.05
0.98
1.00
2.77
1.90
1.96
6.31
f1 (ppm)

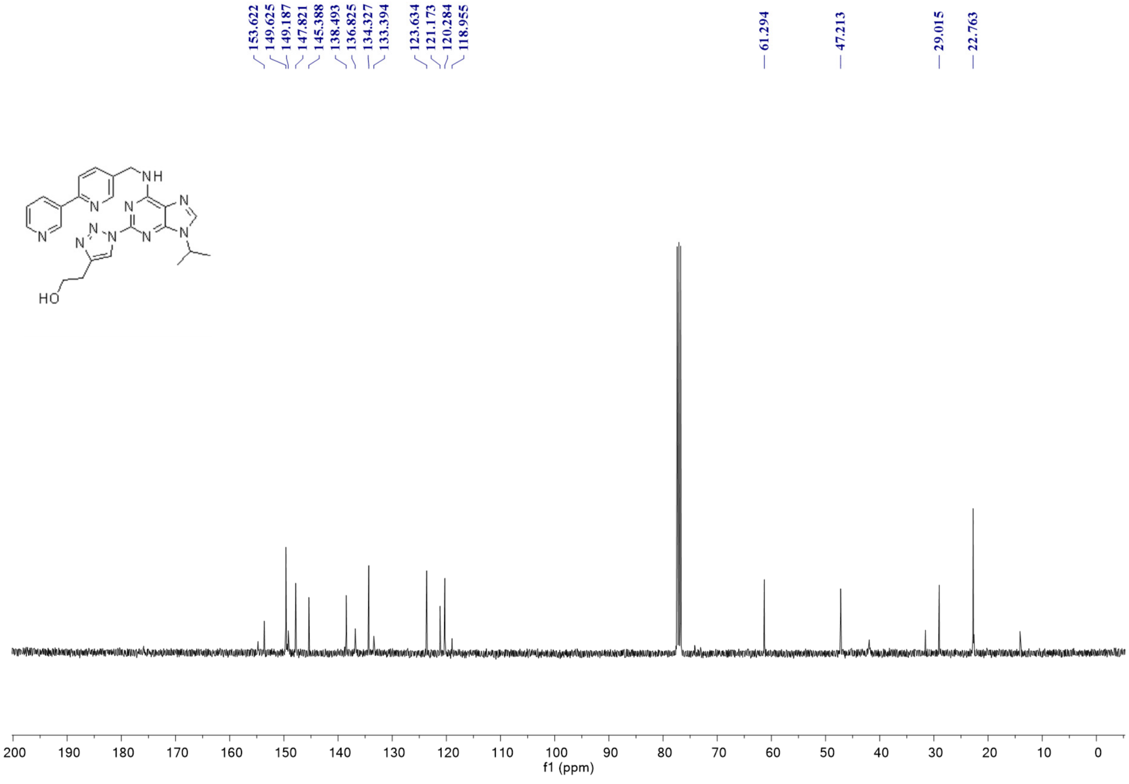
153.622
149.625
149.187
147.821
145.388
138.493
136.825
134.327
133.394
123.634
121.173
120.284
118.955
61.294
47.213
29.015
22.763
f1 (ppm)

28a

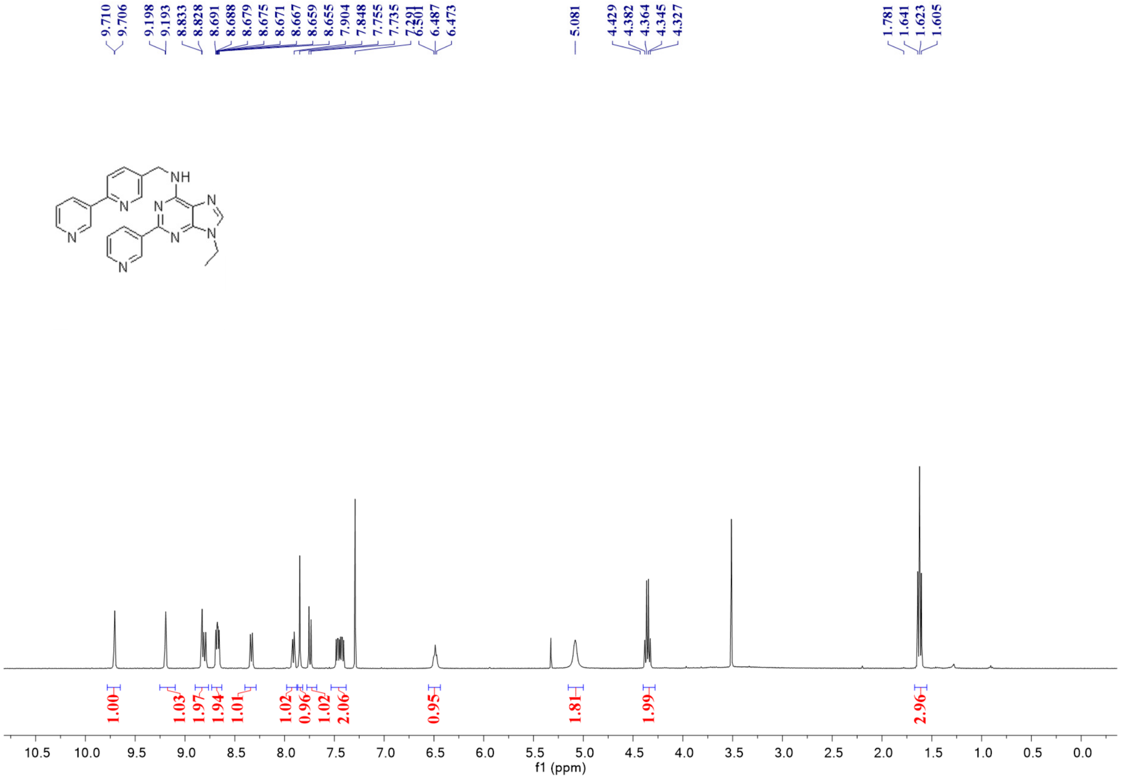

9.710
9.706
9.198
9.193
8.833
8.828
8.691
8.688
8.679
8.675
8.671
8.667
8.659
8.655
7.904
7.848
7.755
7.735
6.487
6.473
5.081
4.429
4.382
4.364
4.345
4.327
1.781
1.641
1.623
1.605
NH
N
1.00
1.03
1.97
1.94
1.01
1.02
0.96
1.02
2.06
0.95
1.81
1.99
2.96
f1 (ppm)

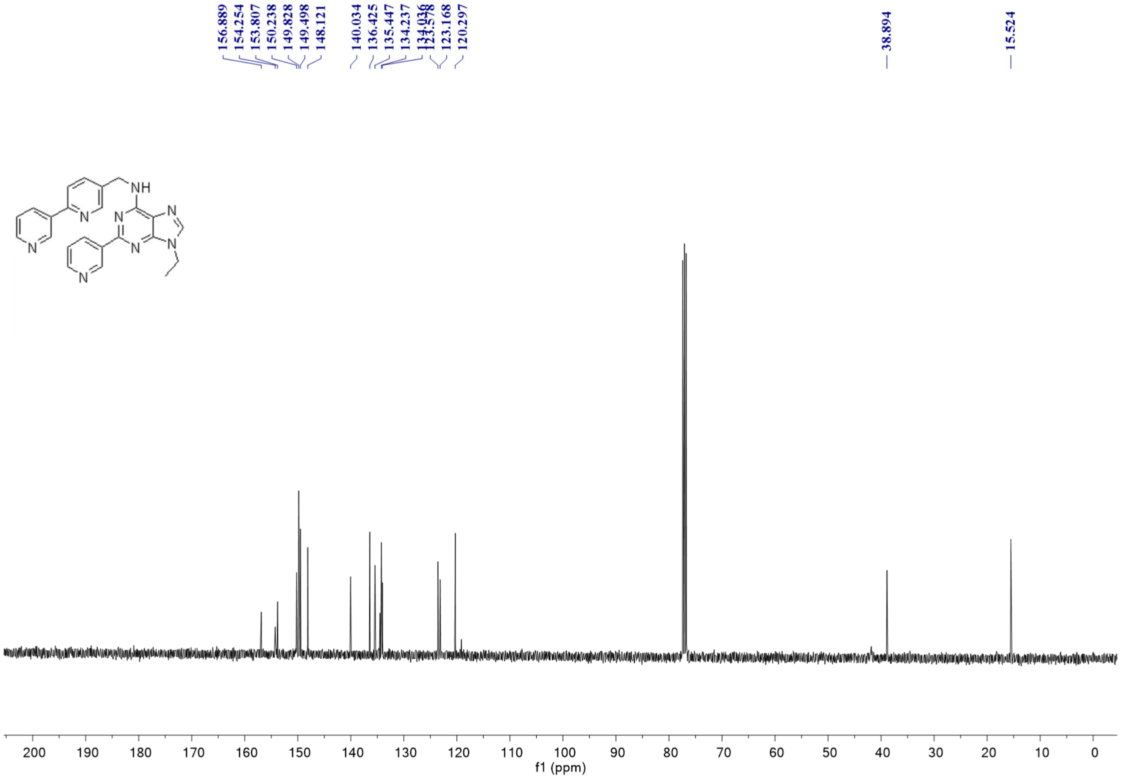

156.889
154.254
153.807
150.238
149.828
149.498
148.121
140.034
136.425
135.447
134.237
123.168
120.297
38.894
15.524
NH
N
f1 (ppm)

28b

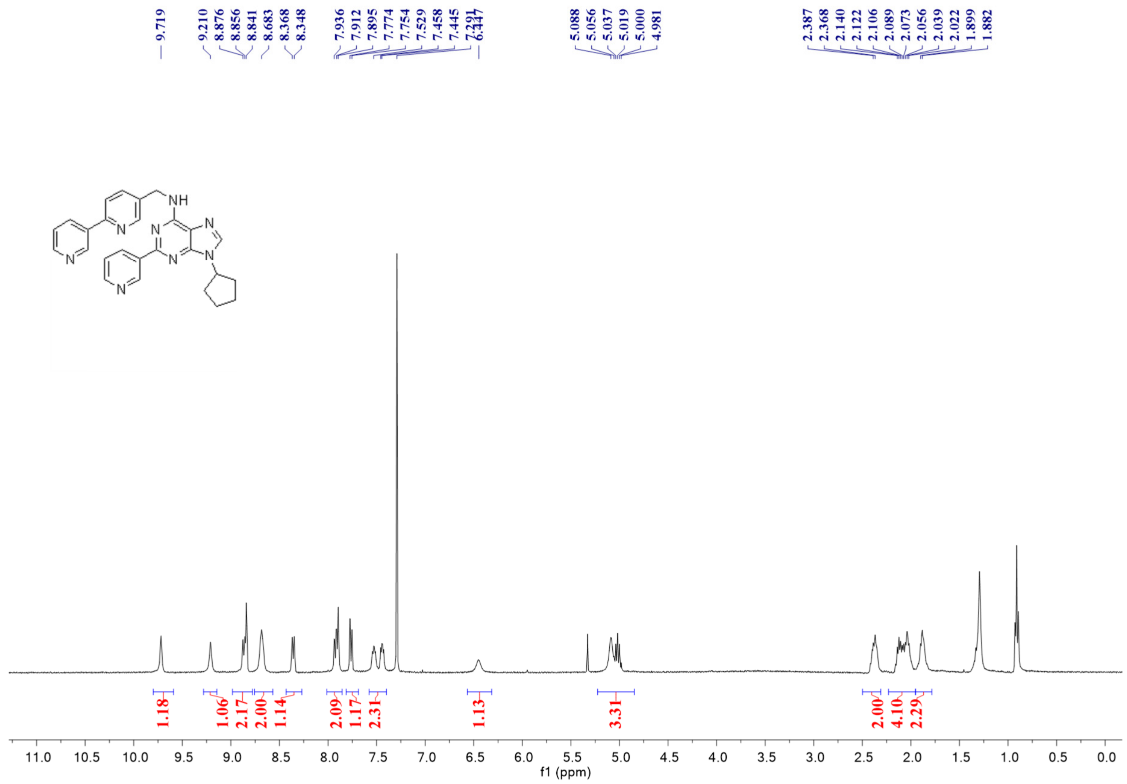

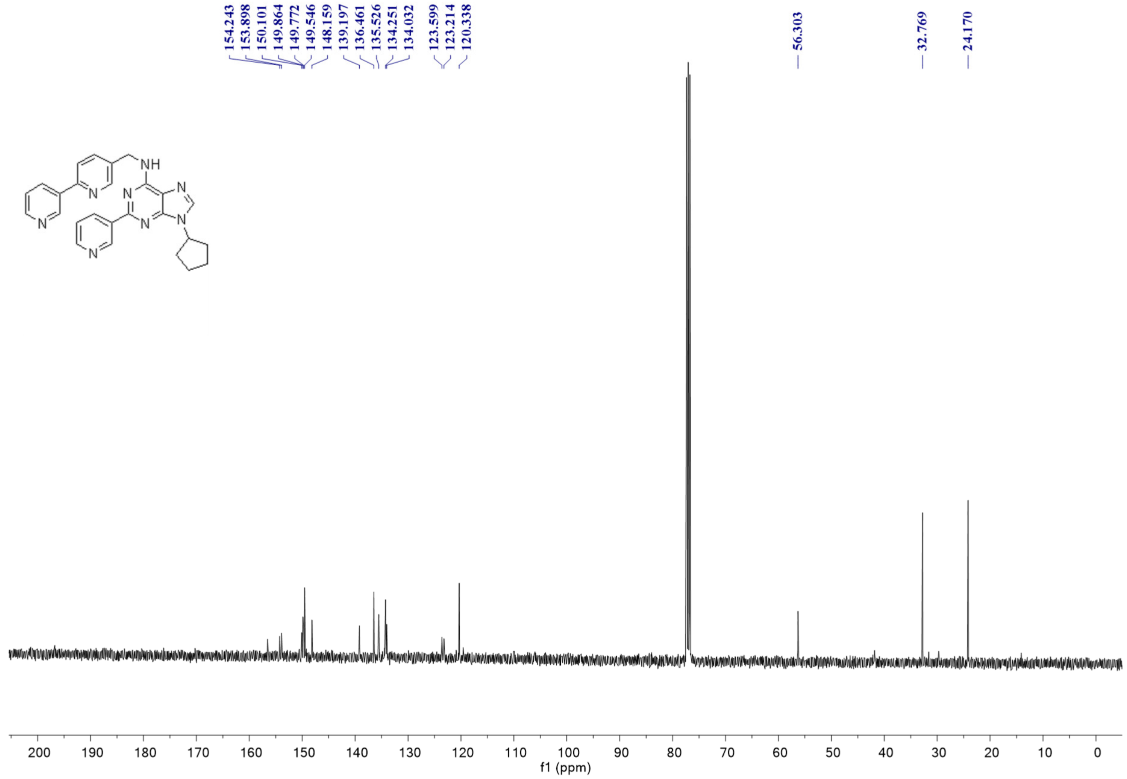

28c

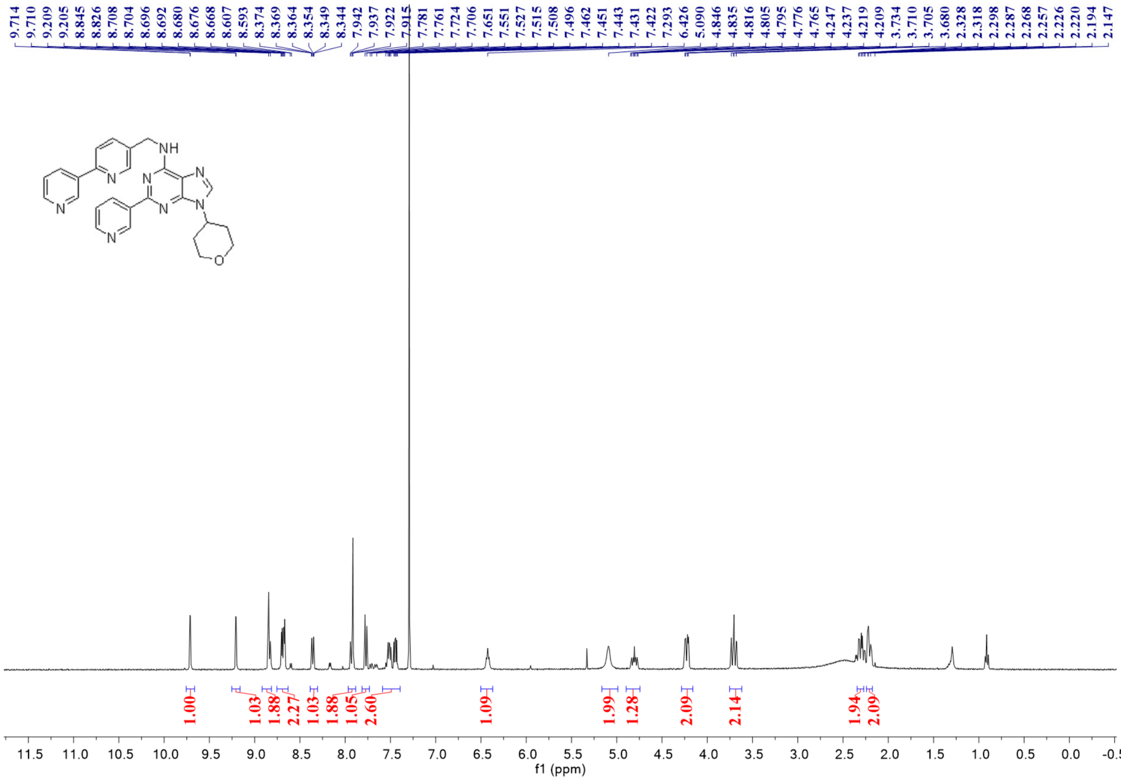
9.714
9.710
9.209
9.205
8.845
8.826
8.708
8.704
8.696
8.692
8.680
8.676
8.668
8.607
8.593
8.374
8.369
8.364
8.354
8.349
8.344
7.942
7.937
7.922
7.915
7.781
7.761
7.724
7.706
7.651
7.551
7.527
7.515
7.508
7.496
7.462
7.451
7.443
7.431
7.422
7.293
6.426
5.090
4.846
4.835
4.816
4.805
4.795
4.776
4.765
4.247
4.237
4.219
4.209
3.734
3.710
3.705
3.680
2.328
2.318
2.298
2.287
2.268
2.257
2.226
2.220
2.194
2.147
NH
N
O
1.00
1.03
1.88
2.27
1.03
1.88
1.05
2.60
1.09
1.99
1.28
2.09
2.14
1.94
2.09
f1 (ppm)

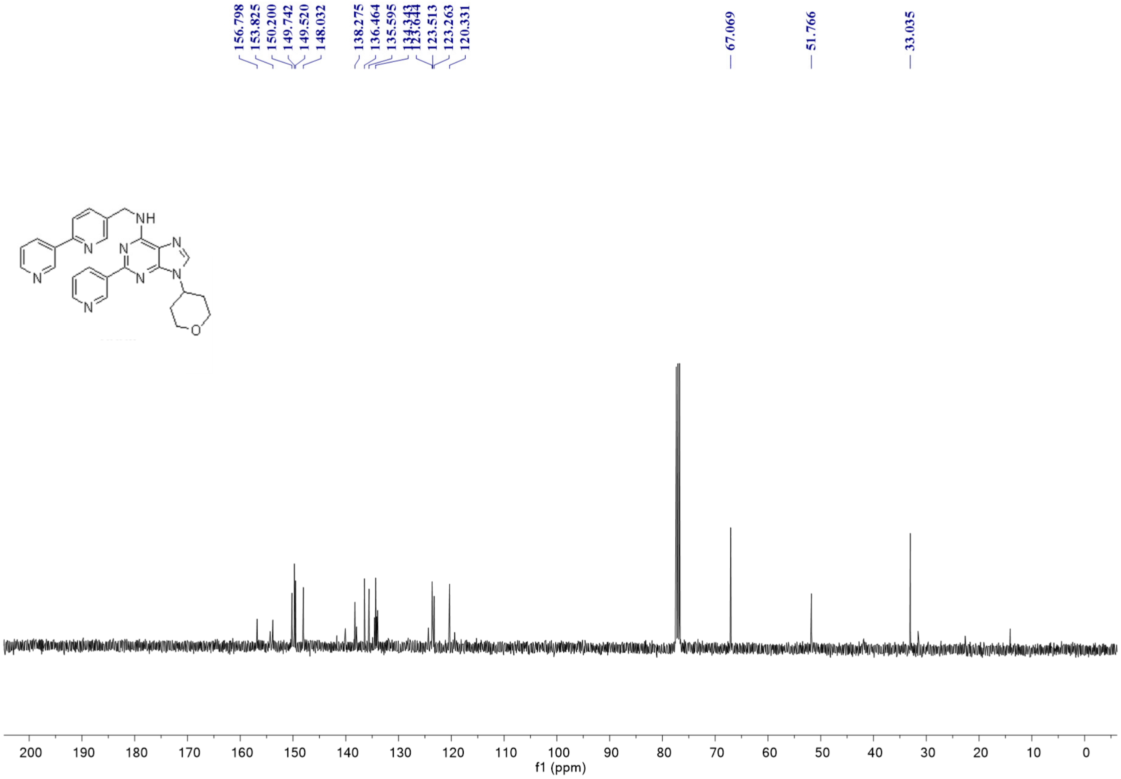
156.798
153.825
150.200
149.742
149.520
148.032
138.275
136.464
135.595
123.513
123.263
120.331
67.069
51.766
33.035
NH
N
O
f1 (ppm)

30a

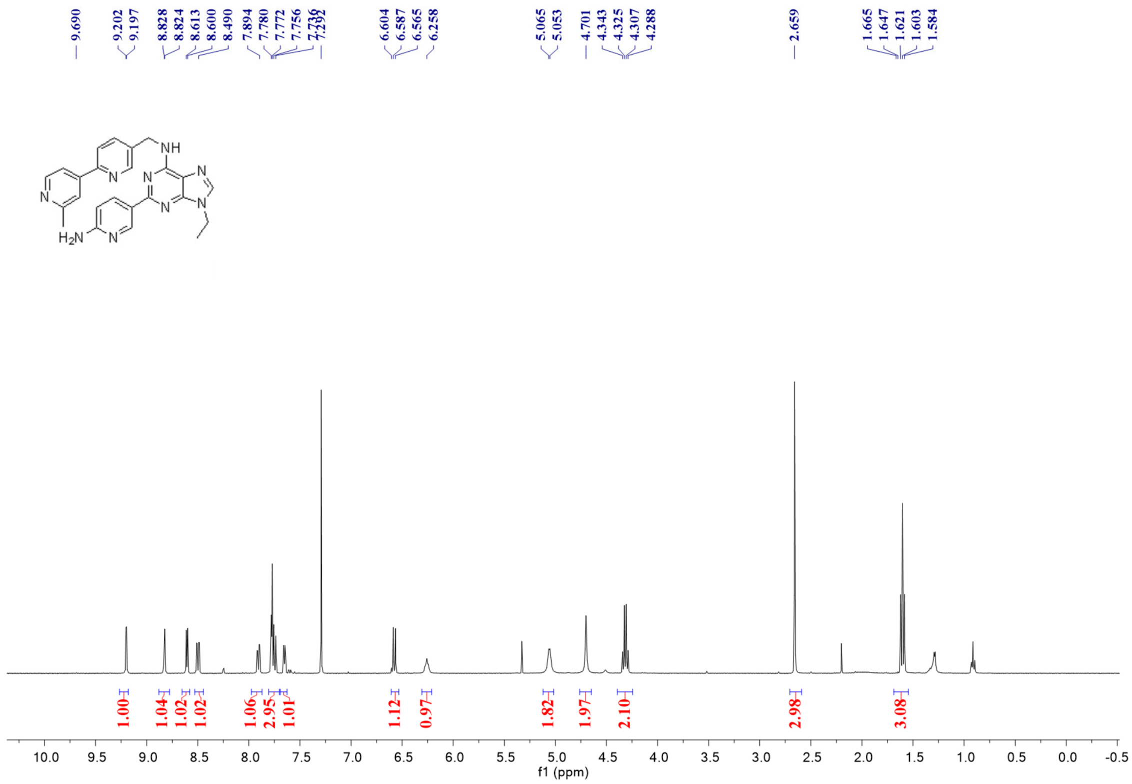
9.690
9.202
9.197
8.828
8.824
8.613
8.600
8.490
7.894
7.780
7.772
7.756
7.736
7.292
6.604
6.587
6.565
6.258
5.065
5.053
4.701
4.343
4.325
4.307
4.288
2.659
1.665
1.647
1.621
1.603
1.584
NH
N
N
N
N
N
N
N
H2N
1.00
1.04
1.02
1.02
1.06
2.95
1.01
1.12
0.97
1.82
1.97
2.10
2.98
3.08
10.0
9.5
9.0
8.5
8.0
7.5
7.0
6.5
6.0
5.5
5.0
4.5
4.0
3.5
3.0
2.5
2.0
1.5
1.0
0.5
0.0
-0.5
f1 (ppm)

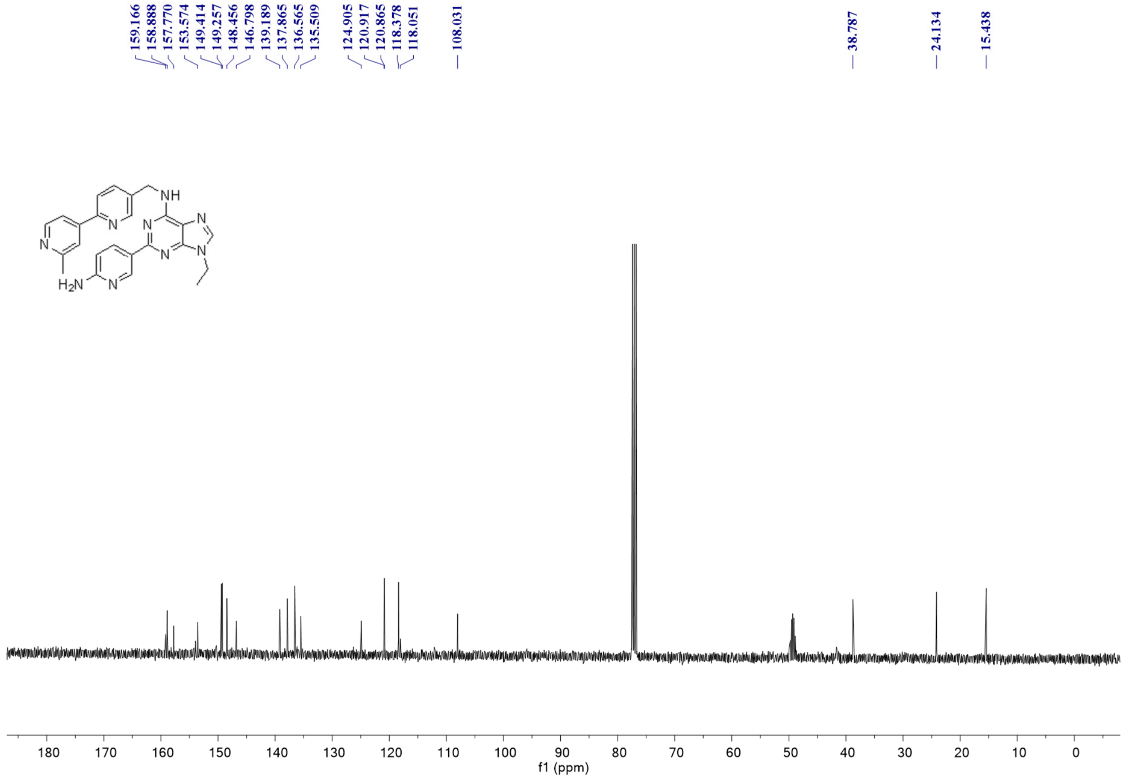
159.166
158.888
157.770
153.574
149.414
149.257
148.456
146.798
139.189
137.865
136.565
135.509
124.905
120.917
120.865
118.378
118.051
108.031
38.787
24.134
15.438
NH
N
N
N
N
N
N
N
H2N
180
170
160
150
140
130
120
110
100
90
80
70
60
50
40
30
20
10
0
f1 (ppm)

30b

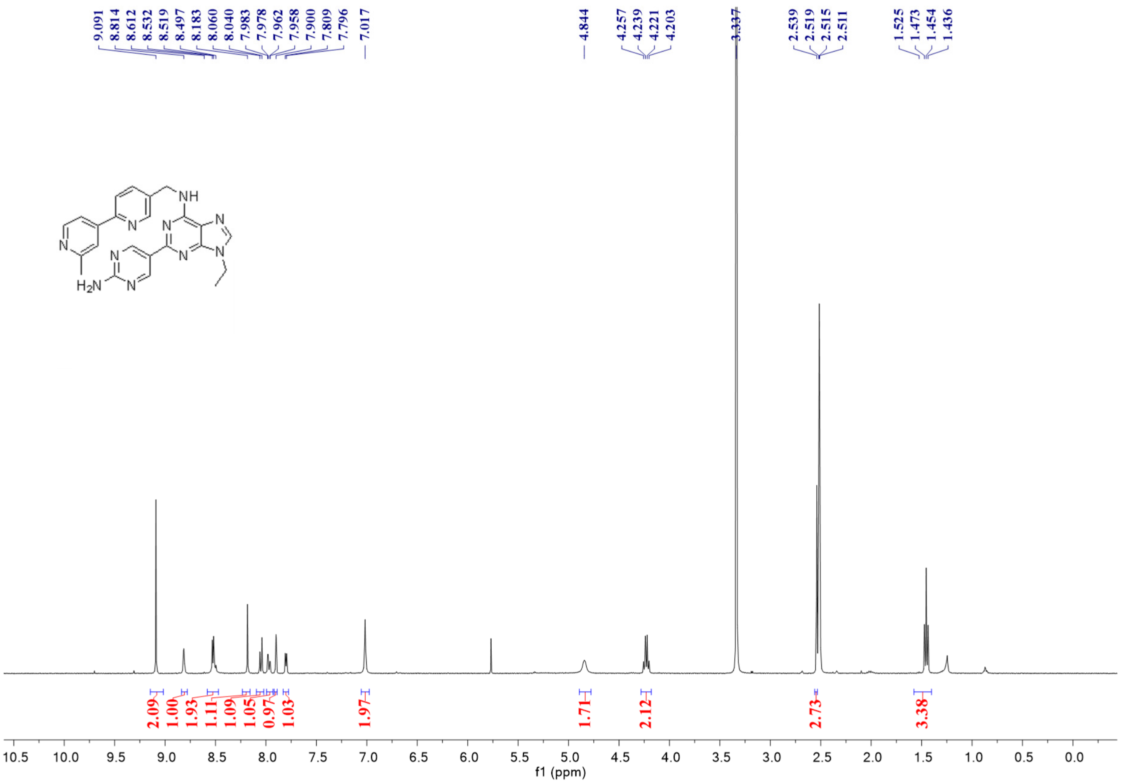
9.091
8.814
8.612
8.532
8.519
8.497
8.183
8.060
8.040
7.983
7.978
7.962
7.958
7.900
7.809
7.796
7.017
4.844
4.257
4.239
4.221
4.203
3.337
2.539
2.519
2.515
2.511
1.525
1.473
1.454
1.436
NH
N
N
N
N
N
N
N
H2N
N
2.09
1.00
1.93
1.11
1.09
1.05
0.97
1.03
1.97
1.71
2.12
2.73
3.38
f1 (ppm)

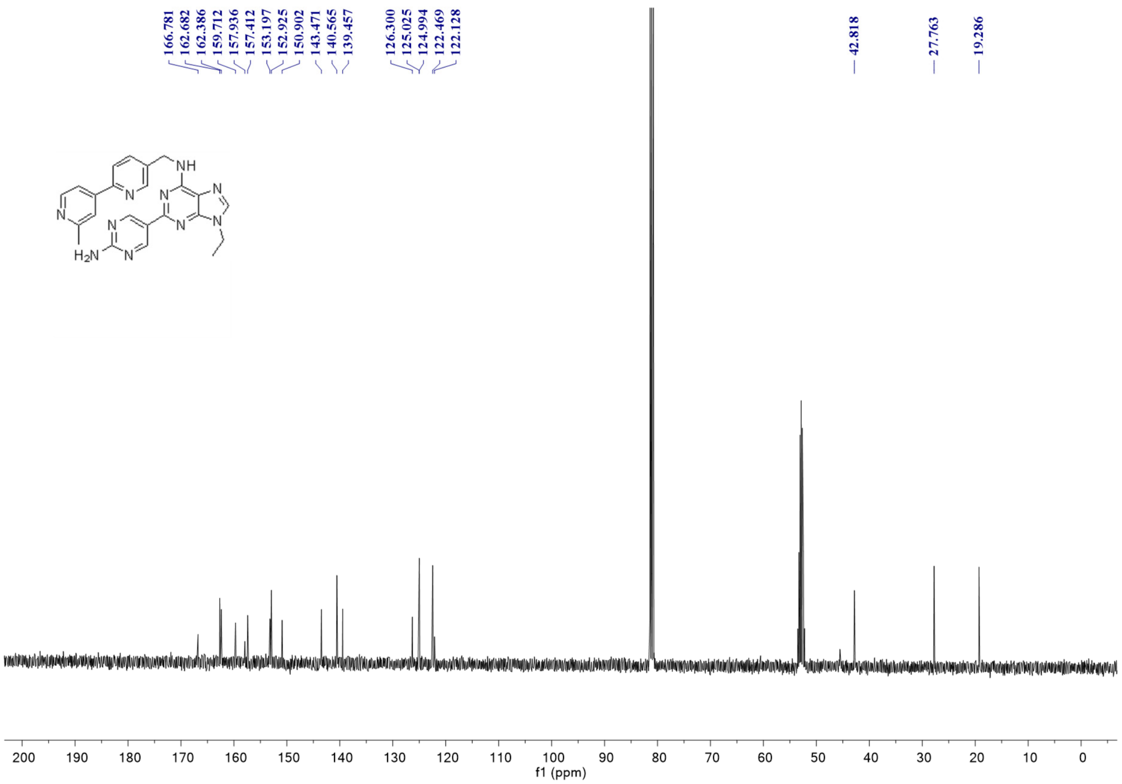
166.781
162.682
162.386
159.712
157.936
157.412
153.197
152.925
150.902
143.471
140.565
139.457
126.300
125.025
124.994
122.469
122.128
42.818
27.763
19.286
NH
N
N
N
N
N
N
N
H2N
N
f1 (ppm)

30c

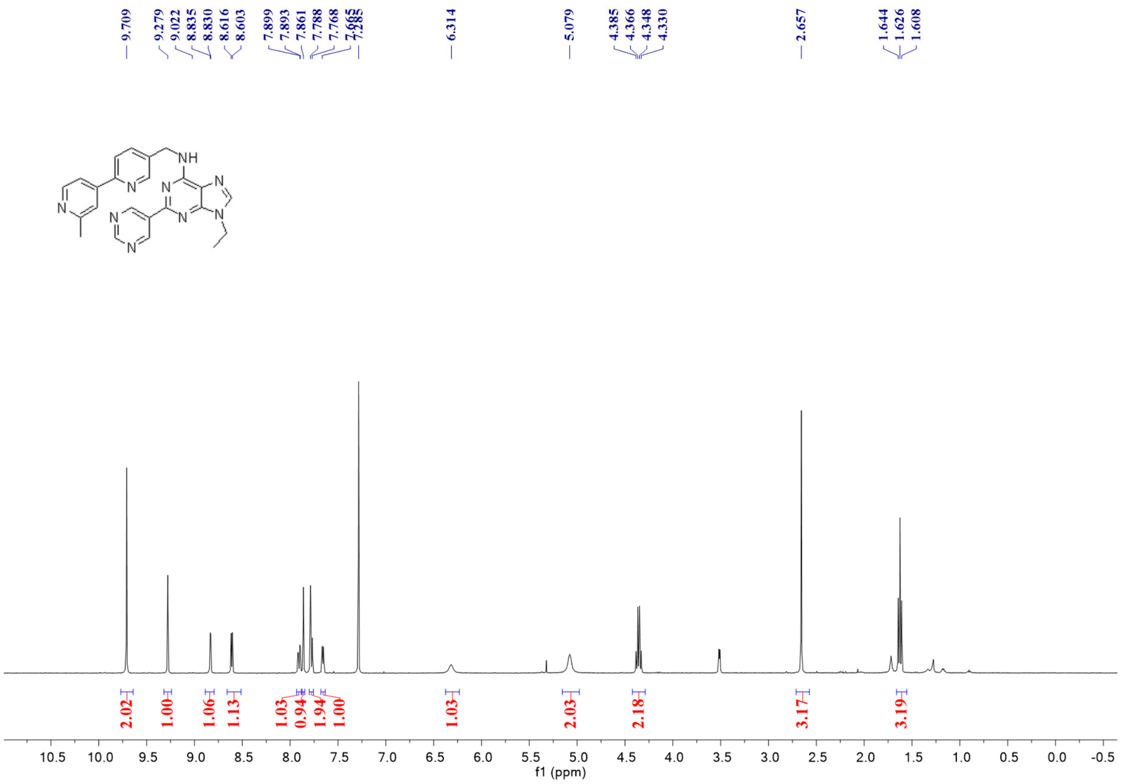
9.709 9.279 9.022 8.835 8.830 8.616 8.603 7.899 7.893 7.861 7.788 7.768 7.665 7.285 6.314 5.079 4.385 4.366 4.348 4.330 2.657 1.644 1.626 1.608
2.02 1.00 1.06 1.13 1.03 0.94 1.94 1.00 1.03 2.03 2.18 3.17 3.19
f1 (ppm)

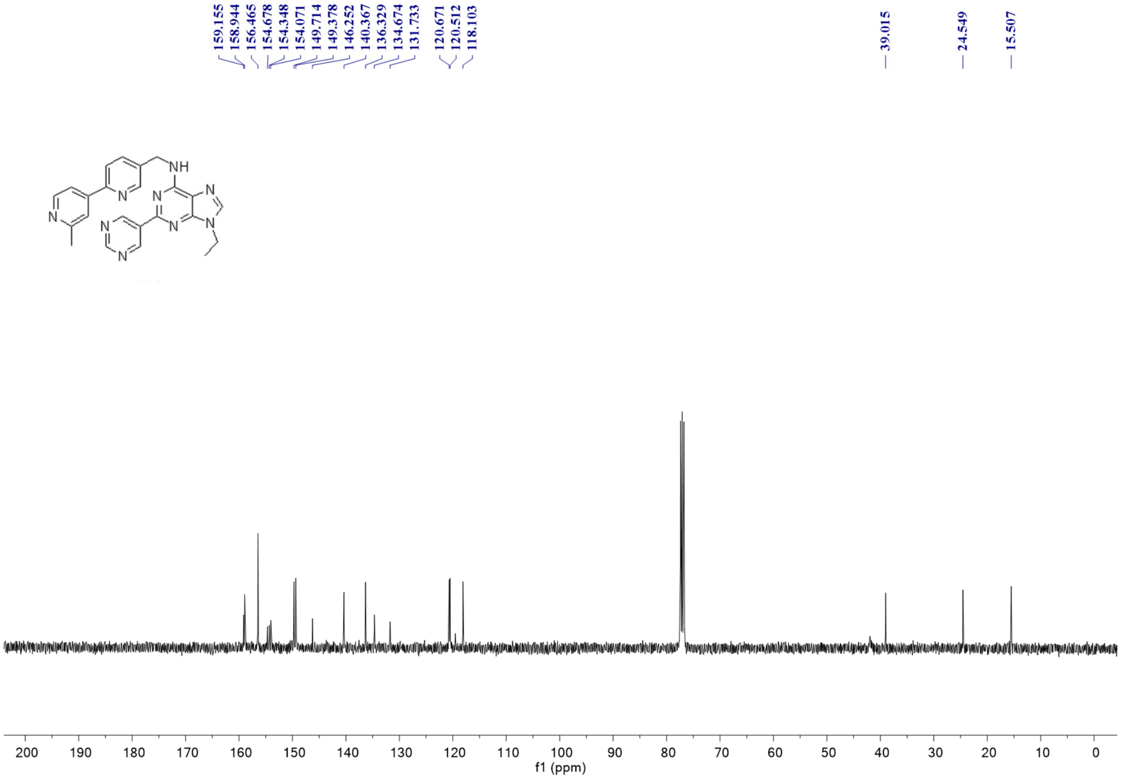
159.155 158.944 156.465 154.678 154.348 154.071 149.714 149.378 146.252 140.367 136.329 134.674 131.733 120.671 120.512 118.103 39.015 24.549 15.507
f1 (ppm)

30d

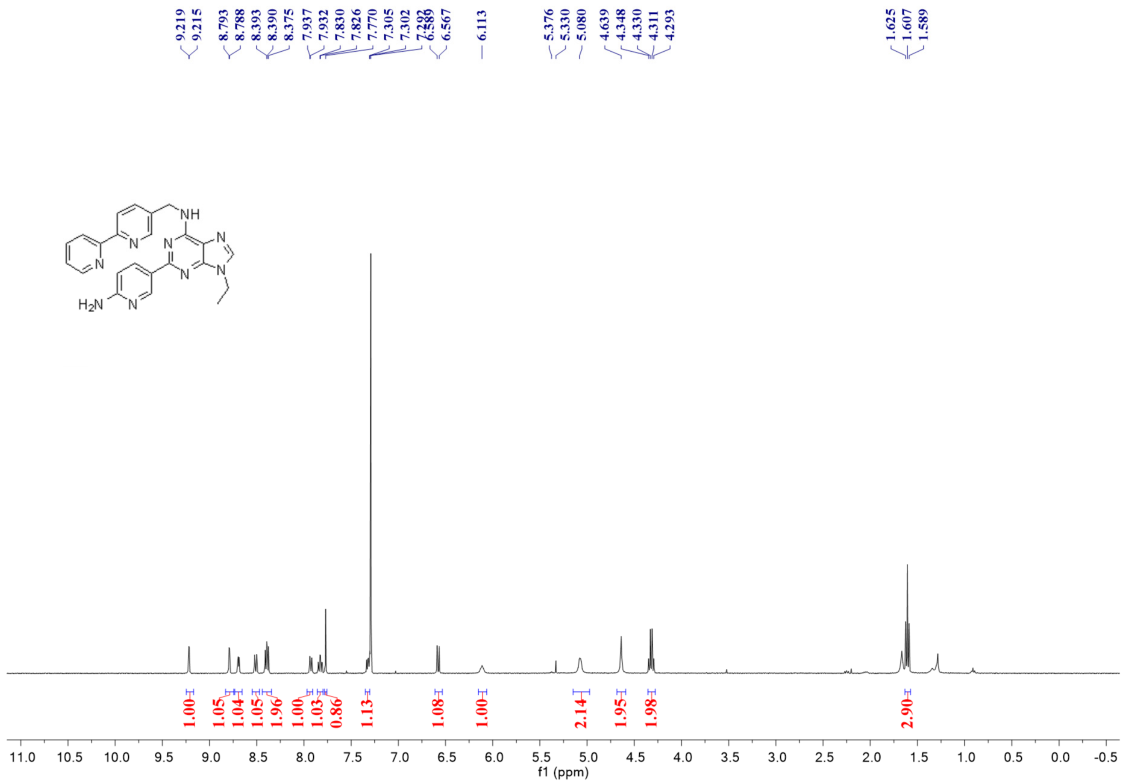
9.219
9.215
8.793
8.788
8.393
8.390
8.375
7.937
7.932
7.830
7.826
7.770
7.305
7.302
6.567
6.113
5.376
5.330
5.080
4.639
4.348
4.330
4.311
4.293
1.625
1.607
1.589
NH
N
H2N
1.00
1.05
1.04
1.05
1.96
1.00
1.03
0.86
1.13
1.08
1.00
2.14
1.95
1.98
2.90
f1 (ppm)

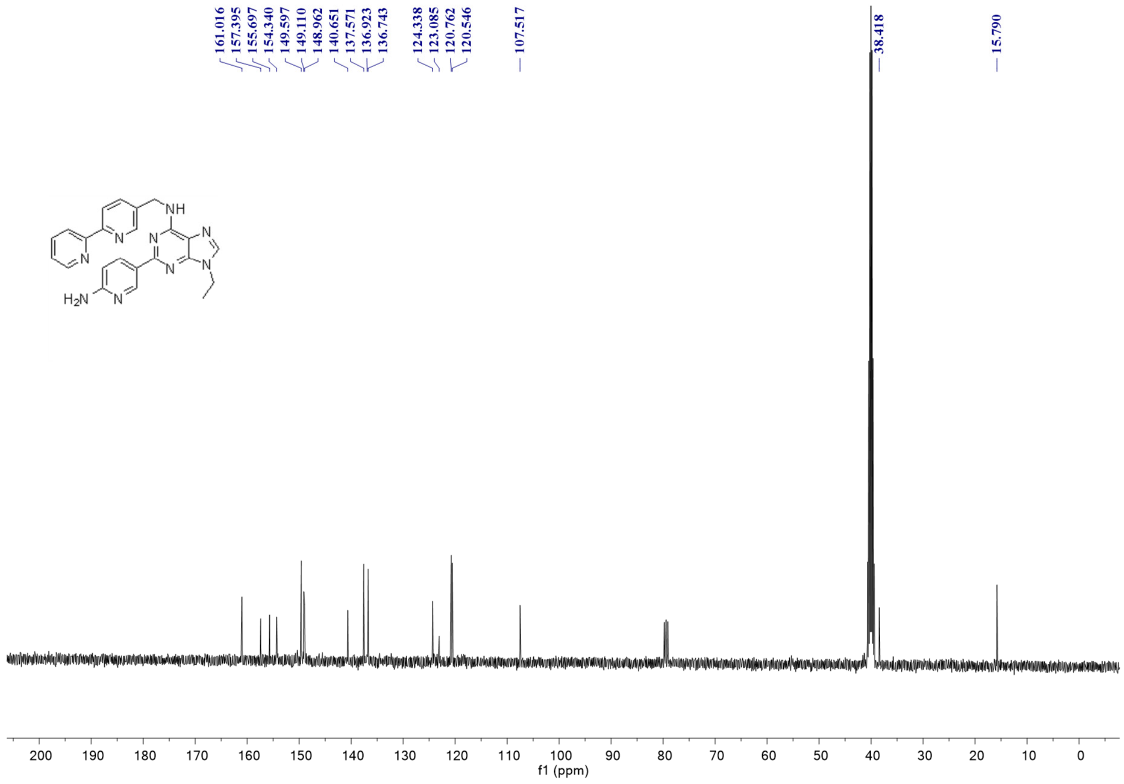
161.016
157.395
155.697
154.340
149.597
149.110
148.962
140.651
137.571
136.923
136.743
124.338
123.085
120.762
120.546
107.517
38.418
15.790
NH
N
H2N
f1 (ppm)

30e

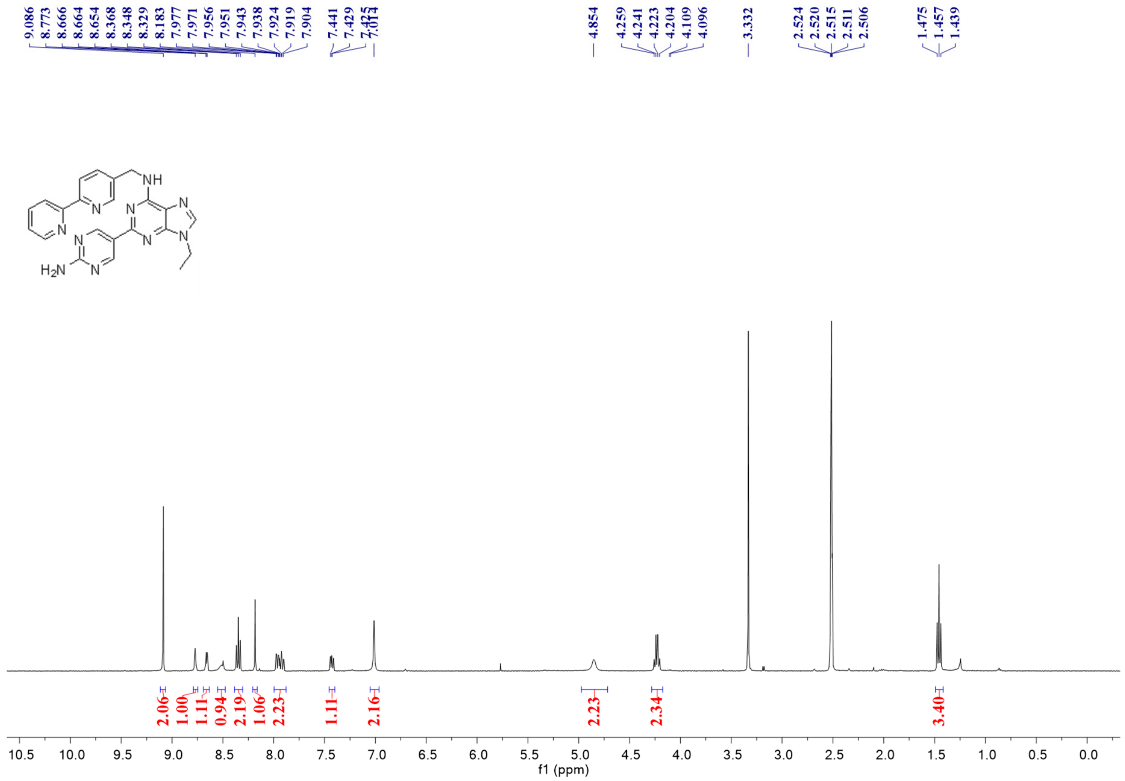
9.086
8.773
8.666
8.664
8.654
8.368
8.348
8.329
8.183
7.977
7.971
7.956
7.951
7.943
7.938
7.924
7.919
7.904
7.441
7.429
7.425
7.014
4.854
4.259
4.241
4.223
4.204
4.109
4.096
3.332
2.524
2.520
2.515
2.511
2.506
1.475
1.457
1.439
NH
N
N
N
N
N
N
N
H_2N
2.06
1.00
1.11
0.94
2.19
1.06
2.23
1.11
2.16
2.23
2.34
3.40
f1 (ppm)

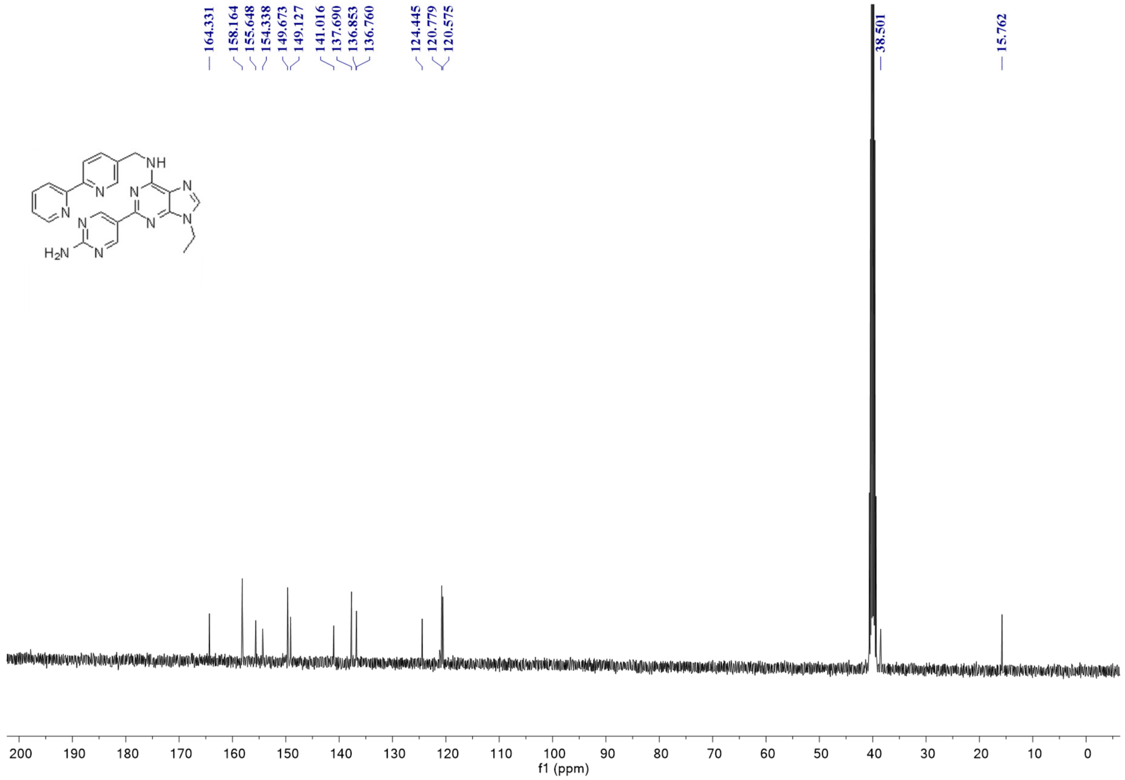
164.331
158.164
155.648
154.338
149.673
149.127
141.016
137.690
136.853
136.760
124.445
120.779
120.575
38.501
15.762
NH
N
N
N
N
N
N
N
H_2N
f1 (ppm)
